# Workflow for E3 Ligase Ligand Validation for PROTAC Development

**DOI:** 10.1021/acschembio.4c00812

**Published:** 2025-02-11

**Authors:** Nebojša Miletić, Janik Weckesser, Thorsten Mosler, Rajeshwari Rathore, Marina E. Hoffmann, Paul Gehrtz, Sarah Schlesiger, Ingo V. Hartung, Nicola Berner, Stephanie Wilhelm, Juliane Müller, Bikash Adhikari, Václav Němec, Saran Aswathaman Sivashanmugam, Lewis Elson, Hanna Holzmann, Martin P. Schwalm, Lasse Hoffmann, Kamal Rayees Abdul Azeez, Susanne Müller, Bernhard Kuster, Elmar Wolf, Ivan Đikić, Stefan Knapp

**Affiliations:** † Institute of Pharmaceutical Chemistry, Goethe University, Max-von-Laue-Str. 9, 60438 Frankfurt am Main, Germany; ‡ Structural Genomics Consortium (SGC), Buchmann Institute for Life Sciences, Max-von-Laue-Str. 15, 60438 Frankfurt am Main, Germany; § Institute of Biochemistry II, School of Medicine, Goethe University Frankfurt, Frankfurt am Main 60590, Germany; ∥ Medicinal Chemistry, Global Research & Development, Merck Healthcare KGaA, 64293 Darmstadt, Germany; ⊥ Chair of Proteomics and Bioanalytics, Technical University of Munich, Emil-Erlenmeyer-Forum 5, 85354 Freising, Germany; # German Cancer Consortium (DKTK), partner site Munich, a partnership between DKFZ and University Center Technical University of Munich, Frankfurt am Main 60590, Germany; ∇ Institute of Biochemistry, University of Kiel, Rudolf-Höber-Str. 1, 24118 Kiel, Germany; ○ German Cancer Consortium (DKTK) site Frankfurt/Mainz, Frankfurt am Main 60590, Germany

## Abstract

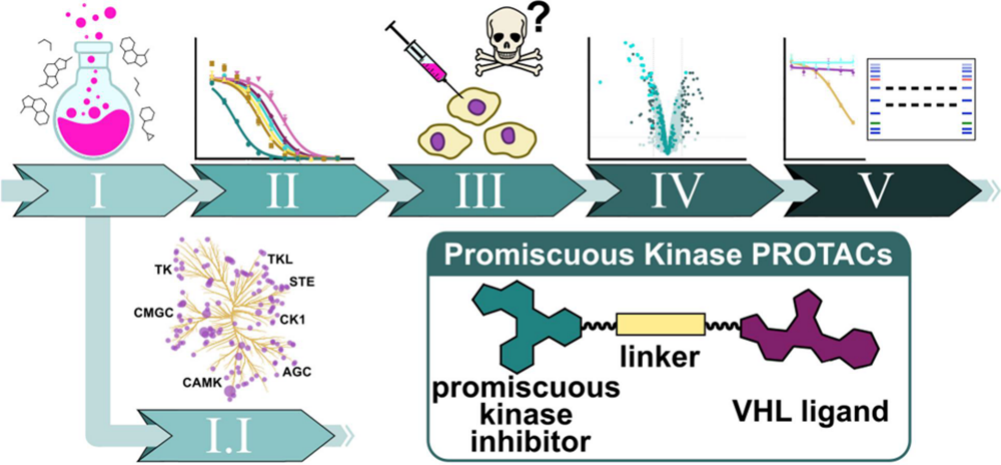

Proteolysis targeting chimeras (PROTACs) have gained considerable attention as a new modality in drug discovery. The development of PROTACs has been mainly focused on using CRBN (Cereblon) and VHL (Von Hippel-Lindau ligase) E3 ligase ligands. However, the considerable size of the human E3 ligase family, newly developed E3 ligase ligands, and the favorable druggability of some E3 ligase families hold the promise that novel degraders with unique pharmacological properties will be designed in the future using this large E3 ligase space. Here, we developed a workflow aiming to improve and streamline the evaluation of E3 ligase ligand efficiency for PROTAC development and the assessment of the corresponding “degradable” target space using broad-spectrum kinase inhibitors and the well-established VHL ligand VH032 as a validation system. Our study revealed VH032 linker attachment points that are highly efficient for kinase degradation as well as some of the pitfalls when using protein degradation as a readout. For instance, cytotoxicity was identified as a major mechanism leading to PROTAC- and VHL-independent kinase degradation. The combination of E3 ligase ligand negative controls, competition by kinase parent compounds, and neddylation and proteasome inhibitors was essential to distinguish between VHL-dependent and -independent kinase degradation events. We share here the findings and limitations of our study and hope that this study will provide guidance for future evaluations of new E3 ligase ligand systems for degrader development.

## Introduction

Proteolysis targeting chimeras (PROTACs) first emerged more than two decades ago, in 2001, as an alternative approach to classical inhibitors in the treatment of diseases such as cancer.^1^ Since then, they have gained considerable attention with several PROTACs having entered clinical trials to date.^2−7^ PROTACs are heterobifunctional molecules containing a ligand that binds to the protein of interest (POI) and a ligand that binds to a ubiquitin ligase (E3), connected by a linker moiety. Simultaneous recruitment of both proteins by the PROTAC, bringing them into proximity, can induce ubiquitin transfer and subsequent proteasomal degradation of the POI via the endogenous ubiquitin-proteasome (UPS) machinery.^1^ As one PROTAC molecule can induce the degradation of many POIs, this catalytic, event-driven mode of action (MoA) offers significant advantages over classical occupancy-driven pharmacology requiring high inhibitor concentrations.^8−10^ The reduced dependency on high and continuous drug exposure due to their catalytic MoA as well as the ablation of all protein functions has been considered a major advantage over conventional small molecule inhibitors. Furthermore, the ability of PROTACs to target any domain of the POI, not just the functionally important domain, has greatly expanded the possibilities of modulating protein function with small molecules.^11^ The challenges in developing PROTACs involve harnessing a complex, multifaceted degradation pathway requiring a bespoke assay panel for the evaluation of rate-limiting steps, in addition to pharmacokinetic challenges related to their relatively high molecular weight,^12^ often violating drug design guidelines such as the Lipinski “rule of five”^13^ and sometimes resulting in poor physicochemical properties.^14−16^ As a consequence, designing PROTACs remains mostly empirical,^17−19^ rendering the development of PROTACs tedious.^20^

To date, only a few E3 ligases and their ligands have been established for PROTAC development, with the majority of ligands targeting the cullin-RING ligases CRBN^21−23^ (Cereblon) or VHL^24−26^ (Von Hippel-Lindau tumor suppressor) and to a lesser extent the IAP/XIAP (inhibitor of apoptosis/X-linked inhibitor of apoptosis) family.^27−29^ For cullin-dependent ligases, compounds have been developed that inhibit key upstream activation events such as neddylation, providing important tools for mechanistic studies.^30^ However, the considerable size of the human E3 ligase family^31,32^ suggests that there are numerous additional druggable domains, offering a large unused area for PROTAC development.

Although VHL-based PROTACs will likely not lead to oral drug candidates due to the peptidic nature of the VHL ligands employed to date, the VHL system provides significant value due to several advantages over the CRBN system. VHL-based PROTACs are much less prone to molecular glue-like behavior, making it more straightforward to identify highly selective PROTAC degraders for target validation studies. In addition, the chiral prolinol pharmacophore of VHL ligands provides an opportunity to use the opposite stereoisomer as a matched nondegrading control. Finally, in contrast to CRBN-based PROTACs, the higher essentiality of VHL reduces the potential for fast resistance development. Therefore, extending the understanding of the VHL-PROTAC opportunity is highly desirable.^33^

The potential of harnessing E3 ligases more broadly would be significant. For example, there are many E3 ligases that are expressed in specific or diseased tissues such as cancer cells.^31,34^ Targeting these E3 ligases may pave the way for tissue-specific POI degradation.^35^ In addition, a larger E3 ligase toolbox^36^ would help to overcome the recently observed resistances that develop after treatment with PROTACs, which often manifest themselves in mutations within the recruited E3 ligase rendering the PROTAC inactive while retaining cell survival.^37−39^ Recently, E3 ligase ligands targeting KEAP1,^40−42^ GID4,^43^ TRIM24,^44,45^ or members of the DCAF family^19,36,46^ have expanded the E3 ligase toolbox, but these ligands have not been used for PROTAC development or, as in the case of DCAF1 ligands, have been used only for the development of very few PROTACs. Covalent ligands have also been developed that can permanently reprogram an E3 ligase to degrade a POI during its cellular life cycle.^47,48^ However, we postulate that the reactivity of the electrophiles currently used to form covalent bonds is likely to cause adverse toxicity in cellular environments.

Despite the advances made, the validation of novel E3 ligases and in particular the evaluation of their degradation efficacy remains difficult. The most common approach demonstrating the utility of an E3 ligase ligand for PROTAC development is the use of POIs known to be readily degraded by the UPS, such as the target protein BRD4 (bromodomain containing 4).^42,48,49^

The highly cell-penetrating BET/BRD4 ligand JQ1,^50^ which contains a well-established exit vector with an easy synthetic route for linker attachment, has become a widely used model system. This is a pragmatic but also a very double-edged approach, as BET/BRD4 inhibition causes significant changes in gene expression, often leading to toxicity at low and substoichiometric inhibitor concentrations.^51,52^ This means that PROTAC-induced degradation is often indistinguishable from degradation caused by general toxicity. Furthermore, this approach does not provide insight into the general utility of the developed E3 ligase ligand for the degradation of other POIs.

Here, we established a workflow that utilizes nonselective ligands targeting protein kinases, a major drug development target family. Promiscuous ligands have been previously employed for evaluating the “degradability” of kinases using mainly CRBN ligands.^53,54^ We therefore used VHL-based PROTACs exhibiting highly promiscuous kinase inhibitors and analyzed their kinome-wide degradation potential by mass spectrometry (MS)-based quantitative proteomics. This strategy drastically reduced the synthetic effort by addressing a large target space and revealed kinase targets that can be degraded by VHL-based PROTACs.

## Results and Discussion

**Figure 1:**
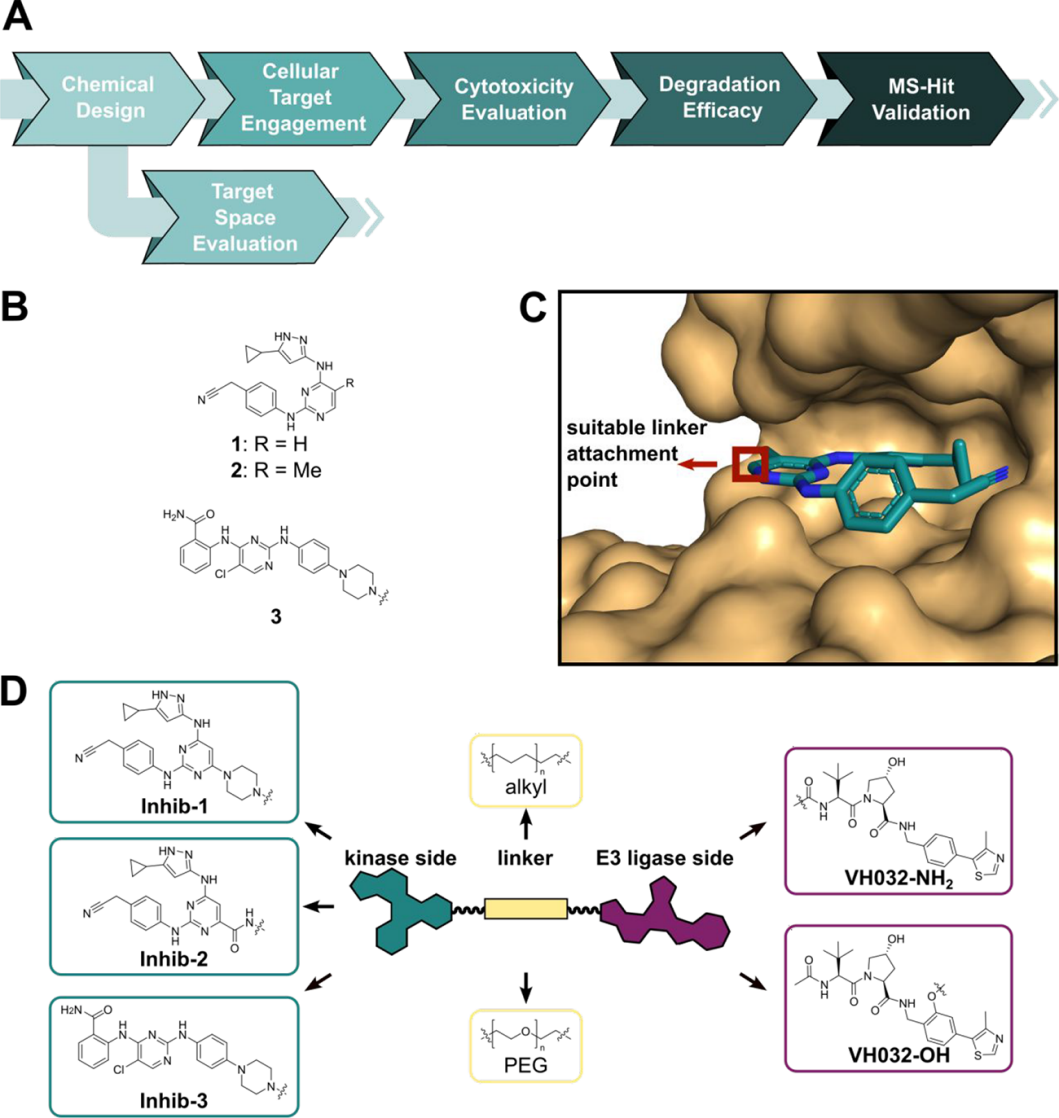
Workflow for validating E3 ligase for PROTAC design using promiscuous kinase inhibitors as POI ligands. (A) Schematic representation of the developed workflow. (B) Structures of the kinase inhibitors **1** (and methylated derivative **2**)^59^ and **3**.^60^ (C) Cocrystal structure of **2** with STK17B (PDB ID: 3LM0) reveals the solvent exposed pyrimidine moiety as a suitable linker attachment point (red box). (D) General chemical structure of PROTACs used highlighting the promiscuous kinase ligand (green), the linker (beige), and the VHL ligand (purple). Two structurally diverse kinase ligands were used based on **1**(59) and **3**(60) (left). Kinase ligands based on **1** were coupled via either a piperazine spacer (**Inhib-1**) or a carboxylic acid (**Inhib-2**) attached to the central pyrimidine moiety, enabling varying linker coupling chemistry. **Inhib-3** was coupled via its terminal piperazine moiety (left bottom). Kinase ligands were coupled to the commonly used VHL ligand VH032-NH_2_ (right top) or VH032-OH (right bottom) using alkyl (mid top) or PEG (mid bottom) linkers.

To establish a workflow for E3 ligase ligand validation, we used the well-studied E3 ligase VHL. The workflow included six evaluation steps (Figure 1A) starting with the chemical design and synthesis; followed by mapping of the target space of the promiscuous kinase inhibitors bearing linker attachment points using DSF (differential scanning fluorimetry^55^) and Kinobeads technology;^56,57^ cell penetration and cellular target engagement evaluation using the NanoBRET (bioluminescence resonance energy transfer) assay^58^ on selected model kinases; assessment of compound cytotoxicity by CellTiterGlo and assessment of the degradation efficacy by MS-based proteomics. The workflow was concluded by validation assays of selected POIs using Western blotting and HiBiT split-luciferase-based assays, including control compounds of the VHL degradation pathway.

Two highly promiscuous kinase inhibitors **1**(59) and **3**(60) (Figure 1B) were selected to serve as POI ligands in this study. Both ligands exhibited a high degree of promiscuity, targeting a wide range of kinases across the human kinome. In addition, the cocrystal structure of **2** with STK17B (PDB ID: 3LM0, Figure 1C) revealed a solvent-exposed moiety on the central pyrimidine ring, suggesting this ring system as a suitable linker attachment point. Either a piperazine (**4**) or a carboxylic acid group (**5**) was introduced at the central pyrimidine ring, allowing for different linker attachment chemistries. While no cocrystal structure of **3** in complex with a kinase was available, the use of this ligand in a broad spectrum NanoBRET tracer^60^ suggested the terminal piperazine as a suitable linker attachment point (**6**). PEG or alkyl linkers were used to couple either one of the three parent kinase ligands to VH032-NH_2_ or VH032-OH, yielding diverse promiscuous kinase PROTACs with two alternative exit vectors located on the E3 ligase side (Figure 1D).

### Kinase Inhibitor Conjugates **4**–**6** Have Broad Activity across the Kinome

**Figure 2:**
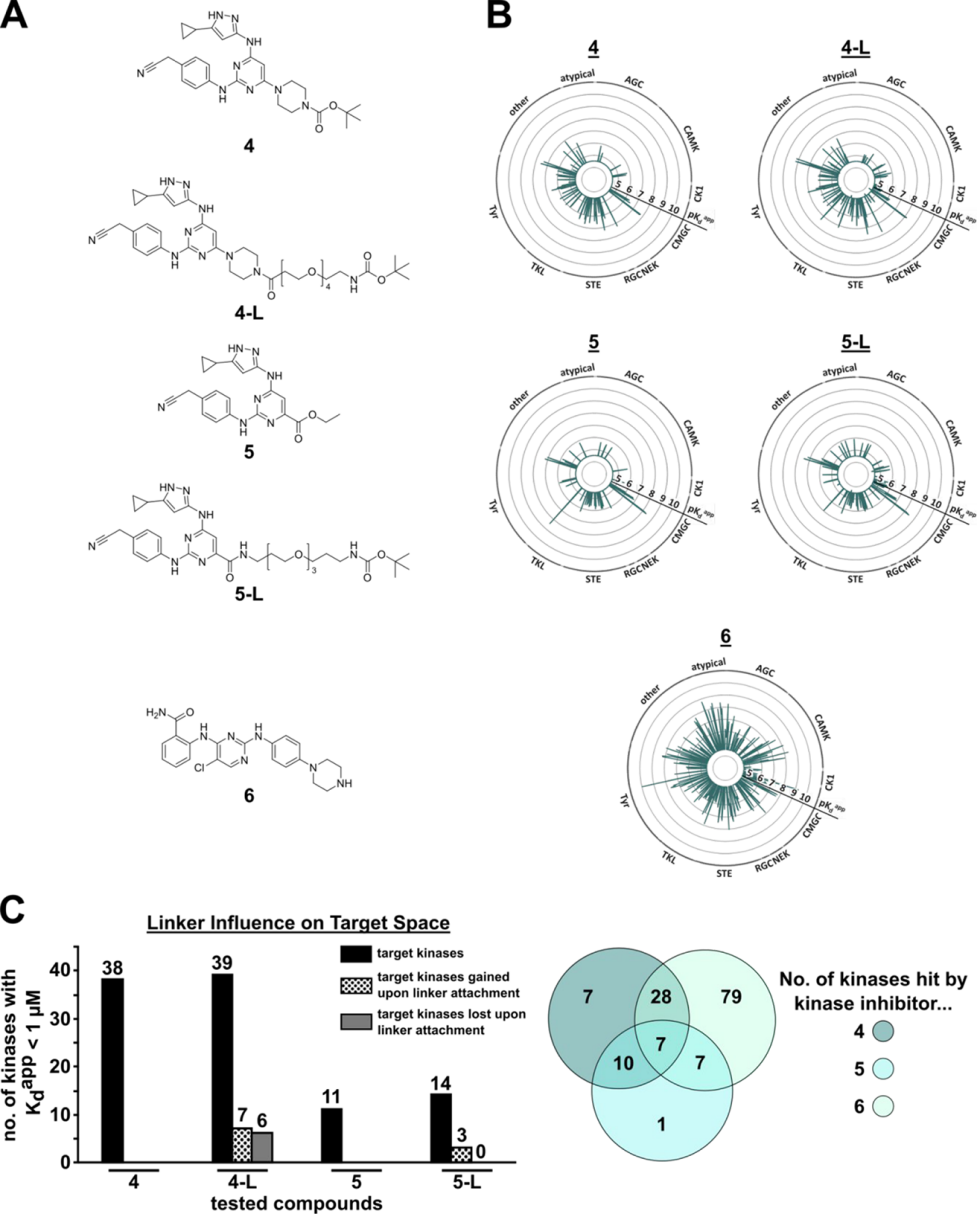
Mapping of the kinome target space. (A) Chemical structures of all kinase parent ligands **4**–**6** and linker conjugates **4-L** and **5-L**. (B) Kinobeads data of kinase parent ligands **4**–**6** and the linker conjugates **4-L** and **5-L** visualized as radar plots (p*K*_d_^app^). (C) Bar chart (left panel) representing the number of kinases engaged with a *K*_d_^app^ below 1 μM threshold in a Kinobeads assay (black), number of target kinases for which *K*_d_^app^ fell below 1 μM threshold upon linker attachment (dotted), and number of kinases for which *K*_d_^app^ increased above 1 μM threshold upon linker attachment (gray) of kinase parent inhibitors **4** and **5** and their corresponding linker conjugates **4-L** and **5-L**. Venn diagram (right panel) showing the number of kinases engaged with a *K*_d_^app^ below 1 μM by each individual kinase parent ligand and target overlaps.

To ensure a broad target coverage across the entire human kinome, the target spaces of the kinase parent ligands bearing the introduced linking moieties **4**–**6** and the linker conjugates **4-L** and **5-L** were evaluated (Figure 2). Screening of the linker conjugates was particularly important, as the multiple hydrogen bond donors and acceptors may offer different binding modes to the kinase ATP binding sites of individual kinases. The introduced bulky moieties likely restrained the binding mode, thus requiring validation of the selected exit vectors through a comprehensive selectivity screen. The corresponding linker conjugate of **6**, but not the final PROTACs, was found to be prone to decomposition under standard storage conditions and was therefore excluded. However, we assumed that the introduction of the piperazine as an exit vector protruded far from the ATP binding sites so that the introduction of longer linker moieties was unlikely to significantly change the selectivity profile of this ligand.

Initial selectivity screening was performed using DSF. The kinase parent ligands **4**–**6** and the linker conjugates **4-L** and **5-L** were evaluated in an in-house panel of 100 representative kinases.^61,62^ Of the 100 kinases screened, 80 kinases showed significant melting point shifts (Δ*T*_m_) induced by at least one of the three kinase parent ligands, with Δ*T*_m_ > 10 °C for some kinases, indicating nanomolar affinities for these targets.

The DSF data revealed that ligand **6** engaged most kinases. Inhibitors **4** and **5** showed only minor differences in their selectivity profiles across the panel, indicating that the introduced piperazine spacer (**4**) and carboxylic acid (**5**) were equally well tolerated (Figure S1). Similarly, linker conjugates **4-L** and **5-L** showed only minor differences in the thermal stabilization of kinases when compared to their respective kinase parent ligands. Thus, the effect of linker attachment on the interaction with the human kinome appeared to be negligible, which validated the selected exit vectors.

Next, the interacting endogenous full-length kinases were assessed using the MS-based Kinobeads assay.^56,57^ Approximately half of the kinome^63^ was detected in the Kinobeads experiments using a Jurkat cell lysate. Again, all three kinase parent inhibitors demonstrated target engagement across various kinase families covering a broad target space (Figure 2B). Consistent with our DSF data, inhibitor **6** interacted with most kinases (79 kinases unique to **6** and 28 shared with **4** with a *K*_d_^app^ < 1 μM). Collectively, the kinase parent ligands engaged with over 120 different kinases with a *K*_d_^app^ < 1 μM. This represented approximately 50% of all kinases detected by this assay format. Only seven kinases were shared by all three ligands (Figure 2C). Interestingly, inhibitor **5** engaged a significantly lower number of kinases compared to **4**, despite their shared core scaffold and near-identical DSF profiles. This suggested that the introduced carboxylic acid moiety was less tolerated than the piperazine spacer moiety and likely hindered interaction with some full-length kinases. Nevertheless, inhibitor **5** was included in the design of the promiscuous kinase PROTACs, as it would facilitate a broader chemical space for linker attachment. The **4-L** and **5-L** linker conjugates exhibited comparable numbers of target kinases to their respective kinase parents, consistent with the results observed in the DSF experiments (Figure 2C). The slight increase in target numbers observed for **5-L** in comparison to that for **5** may originate from additional stabilizing target interactions enabled by the amide moiety. It is noteworthy that despite the nearly unchanged net number of target kinases bound by **4-L**, the target space was altered as seven kinases fell below while six kinases rose above the threshold of 1 μM compared to ligand **4**. In summary, the screening data validated the selected exit vectors for linker attachment and identified approximately 120 kinases that potently interacted with the selected kinase parent inhibitors.

### PROTAC Design

**Figure 3:**
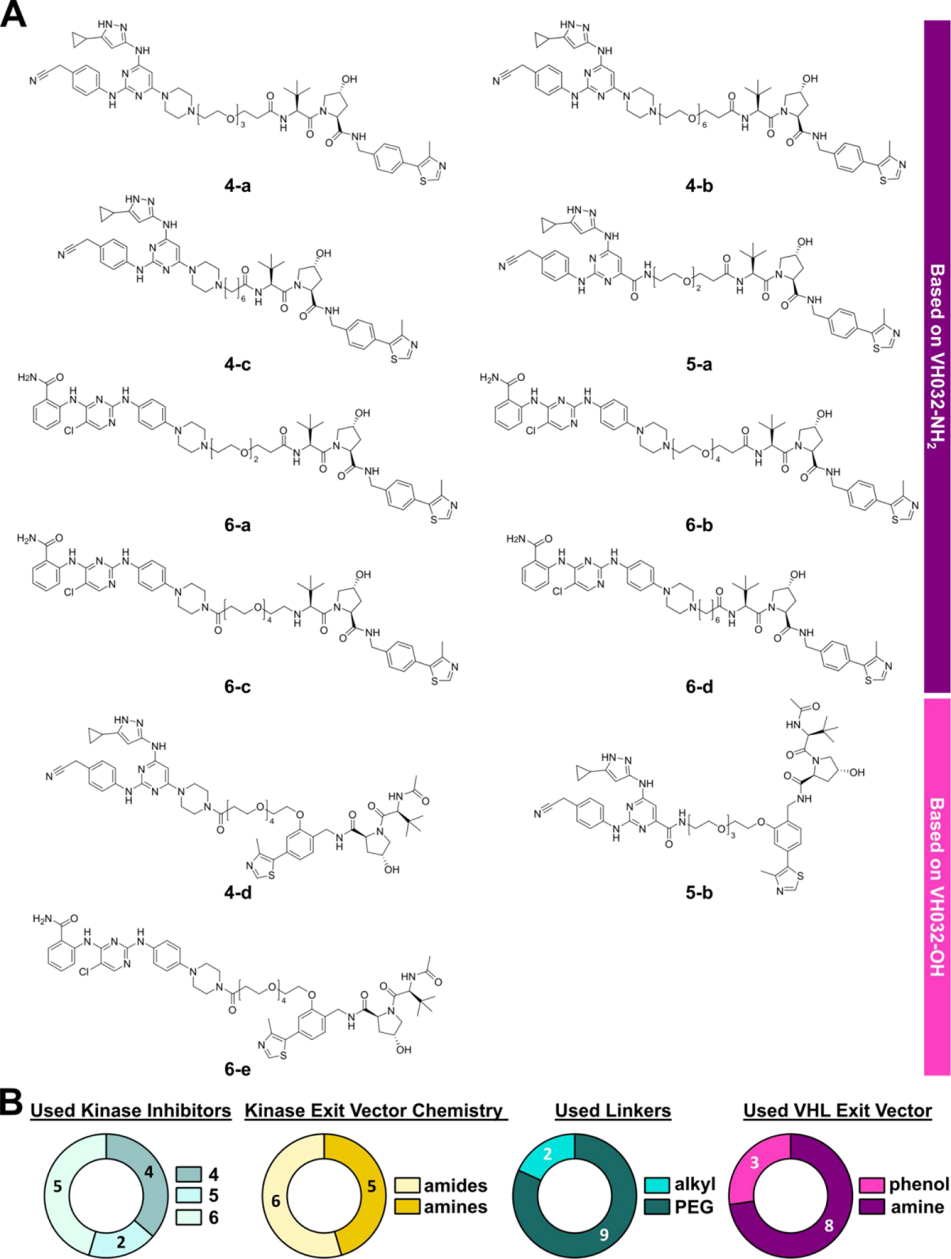
Overview of all synthesized PROTACs. (A) Chemical structures of all synthesized PROTACs. (B) Pie charts summarizing and highlighting the structural characteristics across the PROTACs set: (I) distribution of kinase parent inhibitors **4**–**6**; (II) distribution of the employed linker coupling chemistry on the kinase ligand; (III) distribution of used alkyl and PEG linkers; and (IV) distribution of utilized VHL ligand exit vectors.

After validating the exit vectors of kinase parent inhibitors **4–6** and assessing the target kinome, we proceeded with the synthesis of the promiscuous kinase PROTACs. We aimed to implement a high degree of structural diversity in our PROTAC design by combining different structural features in a modular fashion. By coupling the three kinase parent inhibitors (**4**–**6**) to the commercially available VHL ligand VH032-NH_2_ or VH032-OH using PEG or alkyl linkers of various lengths, a set of 11 structurally diverse PROTACs was synthesized (Figure 3). The synthetic routes are provided in the Supporting Information. Due to the limited target space covered by inhibitor **5**, inhibitors **4** and **6** were preferably employed. Structural flexibility on the kinase ligand coupling site and the resulting ability to accommodate favorable conformations in PROTAC-induced ternary complexes were leveraged by attaching the linkers via either amide bonds or tertiary amines. Tertiary amines were expected to facilitate the formation of ternary complexes due to their greater structural flexibility and adaptability. Amides, which are more structurally rigid, may further stabilize select ternary complexes by fixing favored complex geometries, thereby facilitating their efficacy. Since the incorporation of PEG linkers typically increases the solubility of the PROTAC in water,^64^ this linker type was prioritized and complemented with few alkyl linkers. In addition, we limited the study to flexible rather than constrained linkers, as these structural modifications are usually introduced at a later stage during PROTAC optimization. The resulting linker conjugates were coupled to VH032-NH_2_ exclusively via amide chemistry. The resulting terminal amide moiety has been reported to be important for the interaction with VHL.^65,66^ However, we included PROTAC **6-c** which intentionally lacked the terminal amide moiety to explicitly test its relevance to PROTAC degradation efficacy. A series of linker conjugates were also centrally coupled to VH032-OH, to test an alternative exit vector on VHL. VH032-OH has been previously used in functional PROTACs reported in the literature,^67−69^ which piqued our interest in comparing the degradation efficacy resulting from this alternative coupling site.

### Designed PROTACs Showed Good Cell Permeability and Cellular Target Engagement

Next, we evaluated the cell penetration of all synthesized PROTACs and characterized them in cellular target engagement experiments using NanoBRET assays. To estimate cell penetration, we performed NanoBRET assays in both intact and permeabilized cells and used the resulting IC_50, per._/IC_50, int._ ratios as an approximation of cell permeability. We selected AAK1 and AURKA as representative targets, as all kinase parent ligands (**4**–**6**) potently bound to these kinases in Kinobeads as well as DSF assays and NanoBRET assays showed an excellent signal-to-noise ratio for these targets in intact as well as permeabilized cells.

**Figure 4:**
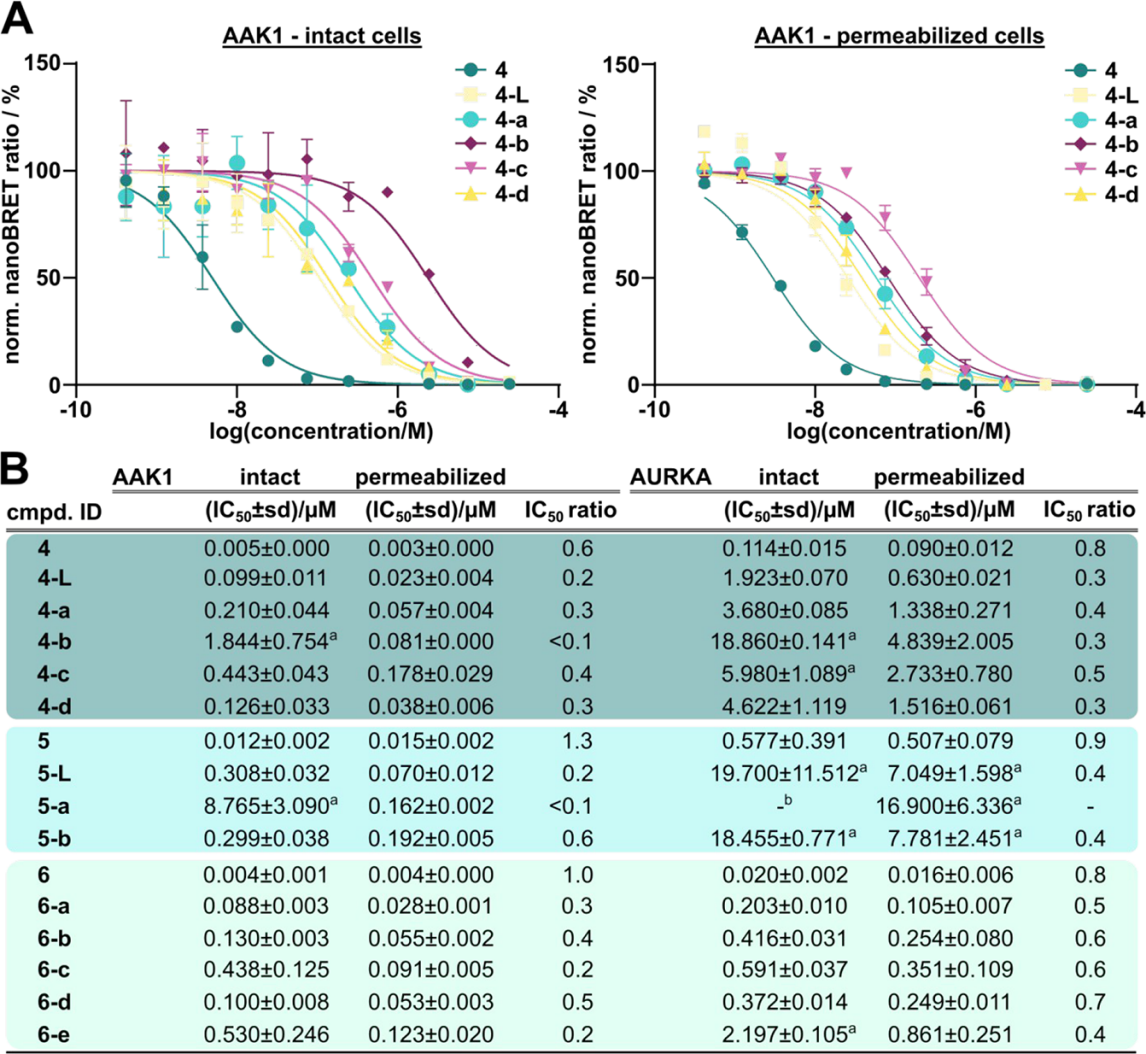
Kinase parent inhibitors, the respective linker conjugates, and the resulting promiscuous kinase PROTACs show cellular target engagement of model kinases. (A) Exemplary NanoBRET dose–response curves of the parent inhibitor **4**, the linker conjugate **4-L**, and the PROTACs **4-a**–**4-d** measured using nanoLUC full-length AAK1 in intact (left) and permeabilized (right) cells. (B) IC_50_ values were calculated as the means of duplicates of a 10-point dose–response curve with errors calculated using the standard deviation. Compound sets based on the same kinase parent inhibitor are clustered in colored boxes (**4** represented in dark green, **5** represented in light blue, and **6** represented in light green). ^a^Estimated IC_50_ values are based on extrapolation. ^b^IC_50_ values were outside the assay window.

Parent inhibitors **4**–**6**, the corresponding linker conjugates **4-L** and **5-L** as well as the resulting PROTACs were all tested in NanoBRET experiments (see Figures S2–S8 for dose–response curves). In general, the greatest decrease in cellular binding affinity was caused by the attachment of the linkers onto the parent ligands, while the addition of the E3 ligase ligand resulted in a comparably smaller decrease for both AAK1 and AURKA (Figure 4). Part of this loss of cellular potency was due to weaker cell penetration as indicated by the IC_50, per._/IC_50, int._ ratios, in particular for the PROTACs (Figure 4B). It is noteworthy that the ratios for some of the compounds markedly differed between AAK1 and AURKA, highlighting how such ratios can only provide a qualitative but nevertheless valuable approximation of compound cell permeability. Nevertheless, the majority of PROTACs engaged AAK1 with submicromolar binding affinities in cellulo, with the exceptions of PROTACs **4-b** and **5-a,** which showed single-digit micromolar IC_50_ values. The high potency of inhibitor **6** for AURKA was also observed for the corresponding PROTACs which displayed submicromolar binding affinities, except for **6-e**. The PROTACs based on inhibitor **5** showed an overall weak AURKA target engagement.

The majority of PROTACs showed comparable VHL binding to the VHL parent ligand VH032 with low single-digit micromolar binding affinities in permeabilized cells (Table S1) but significantly lower affinities in intact cells (not shown). Similar trends were observed for VHL-based PROTACs in a recent publication from our group.^12^ PROTACs **4-d**, **5-b**, and **6-e**, which all shared the VH032-OH exit vector, displayed similar VHL engagement compared to VH032-NH_2_-based PROTACs in permeabilized cells, indicating that the phenolic VH032-OH exit vector was equally tolerated. Consistent with the literature, PROTAC **6-c**, which deliberately lacked the terminal amide moiety at the linker attachment point on the VHL ligand, did not bind strongly to VHL. The NanoBRET study demonstrated that all PROTACs, except **4-b** and **5-a**, had significant binding affinity for both selected kinase targets as well as for the VHL E3 ligase.

### Cytotoxicity Correlated with Compound Promiscuity

**Figure 5:**
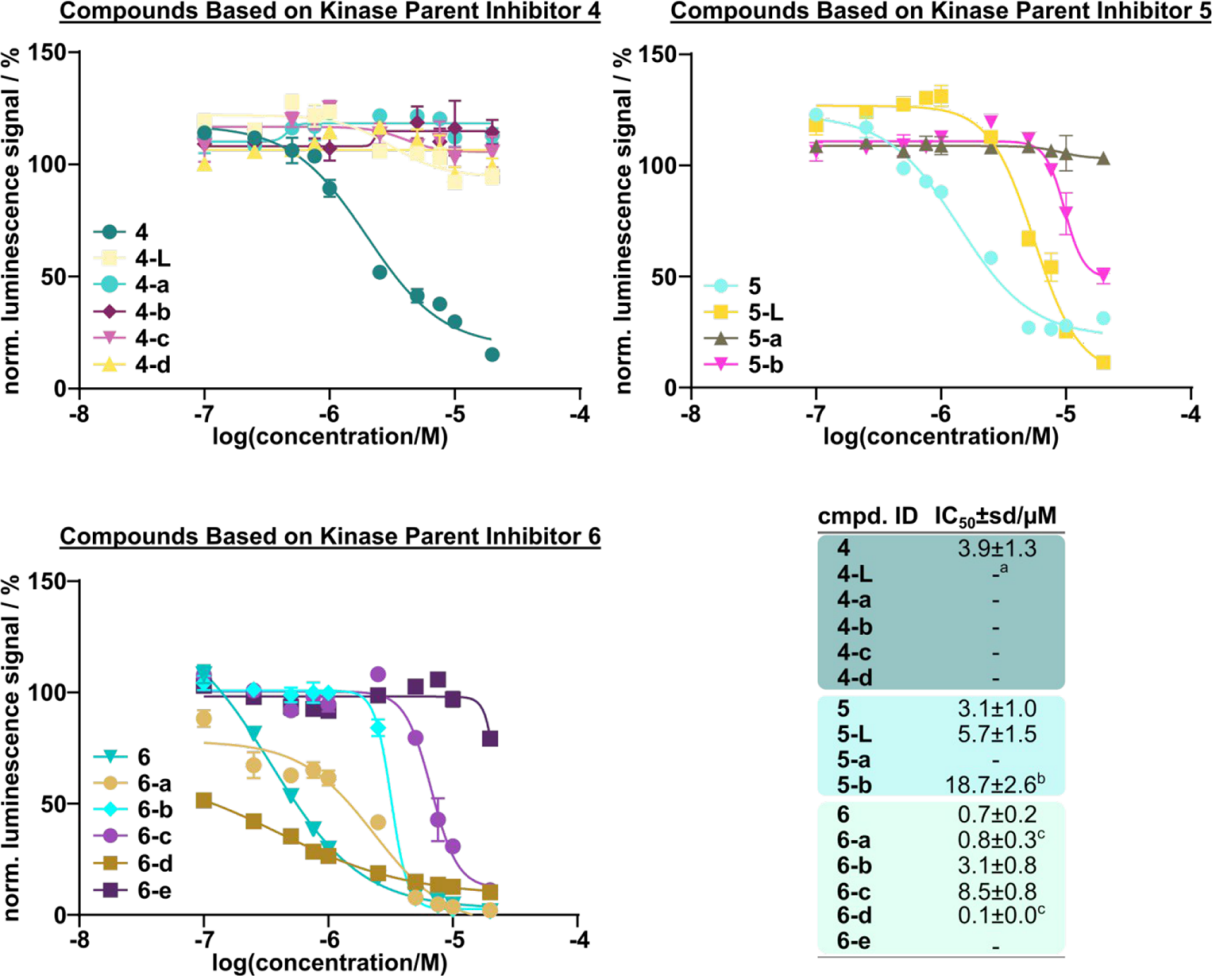
Level of promiscuity correlates with compound cytotoxicity. Exemplary CellTiterGlo dose–response curves of Jurkat cells treated with kinase parent inhibitors **4**–**6**, linker conjugates **4-L** and **5-L**, and the resulting promiscuous kinase PROTACs after 24 h of incubation. Compounds are shown according to the used kinase parent inhibitor. Bottom right: calculated IC_50_ values are shown as the mean of triplicate measurements in μM. Errors were calculated using the standard deviation. Compounds were grouped based on their parent ligand as shown in the figure caption. ^a^IC_50_ values were outside the assay window. ^b^Estimated IC_50_ values are based on extrapolation in GraphPad. ^c^Estimated IC_50_ values as corresponding dose–response curves were not sigmoidal.

Toxicity has a profound effect on transcription, translation, and other cellular functions that can lead to a decrease of POI expression levels, which could be misinterpreted as PROTAC-mediated degradation. Inhibition of multiple essential kinases can also lead to cytotoxic effects and, consequently, to protein degradation by the ubiquitin system. To distinguish between kinase degradation due to cytotoxic effects and degradation induced by the promiscuous PROTACs, we evaluated the cytotoxicity profiles of all synthesized PROTACs, the respective kinase parent inhibitors (**4–6**), and the linker conjugates **4-L** and **5-L** in Jurkat cells using the CellTiterGlo assay (Figure 5). Cells were treated for 24 h to ensure a detectable and reliable cell viability read-out. As expected, the most promiscuous inhibitor, compound **6**, showed the most pronounced effect on cell viability with a submicromolar IC_50_ value, further confirming on-target activity across various essential kinases as already observed in previous Kinobeads experiments. While being generally weaker, inhibitors **4** and **5** showed comparable effects on cell viability with single-digit micromolar IC_50_ values. The difference in cell viability among the parent kinase inhibitors clearly demonstrated a strong correlation between promiscuity and the resulting cytotoxicity of these compounds. The linker conjugates **4-L** and **5-L** and the PROTACs displayed weaker effects on cell viability compared to the respective kinase parent inhibitors, in line with their weaker cell permeability. Similar to the kinase parent inhibitors, PROTACs based on inhibitor **6** showed a much more pronounced effect on cell viability than PROTACs derived from inhibitors **4** and **5**.

PROTAC **6-e** was the sole exception from the inhibitor **6**-based PROTAC series for which no cytotoxicity was observed. Interestingly, PROTACs **6-a** and **6-d** showed particularly pronounced effects on cell viability in comparison to the other PROTACs of this series. It is noteworthy that the compounds based on inhibitor **5** demonstrated significantly stronger effects on cell viability in comparison to the inhibitor **4**-derived series despite exhibiting a lower degree of inhibitor promiscuity.

### PROTACs **4-a** and **6-b** Induced Strong UPS-Dependent Degradation of Kinases

To identify which kinases were degraded by the synthesized PROTACs, we performed quantitative proteomic analyses. Cells were treated with 1 μM of PROTACs for 6 h to minimize the impact of cytotoxicity on protein degradation. Initially, the kinase parent inhibitors (**4**–**6**) were profiled to investigate the effect of the inhibitory activity on kinase expression levels. The inhibitors were tested at 0.25 μM to account for their more favorable physicochemical properties and cell penetration. Compound **5** reduced protein levels of PLK1 and BUB1, and **6** reduced PIK3C3 levels, while no significantly downregulated kinases were observed for inhibitor **4** (Figure S9, left panels). These data demonstrated that kinase inhibition by the kinase parent inhibitors did not cause significant kinase degradation/downregulation in Jurkat cells after 6 h of treatment. The inhibitory activity of the used kinase inhibitors hence had a negligible effect on the obtained degradation profiles at a concentration of 0.25 μM. The parent kinase inhibitors were additionally tested at a 10-fold higher concentration (2.5 μM) to investigate any effects on kinase levels caused by inhibition of kinase catalytic activity or cytotoxicity. Surprisingly, the proteomic data revealed that no additional kinases showed lower protein levels for inhibitors **4** and **5**. However, treatment with inhibitor **6** revealed lower protein levels of additional kinases, probably due to its cytotoxicity (Figure S9, right panels). All three kinase parent inhibitors (**4**–**6**) induced significant changes in protein expression levels of several nonkinase proteins at 2.5 μM. To investigate potential cell line-dependent differences, the effects of **4** and **5** on kinase expression levels were also profiled in MCF-7 cells using the same treatment conditions (Figure S10). Both inhibitors showed comparable downregulation profiles in MCF-7 cells when treated at 0.25 μM, compared with experiments performed in Jurkat cells.

Next, we evaluated the degradation efficacy of the PROTACs. At the chosen treatment time (6 h) and concentration (1 μM), PROTACs **4-a**, **4-c**, **6-a, 6-b**, and **6-d** showed significant degradation of several kinases, whereas treatment with PROTACs **4-b**, **4-d**, **5-a**, **5-b**, **6-c**, and **6-e** did not result in significant degradation of any kinase detected by our proteomic analysis in MCF-7 cells (Figures S11 and S12). It is likely that PROTACs **5-a** and **5-b** were inactive, as they did not bind many kinases in our Kinobeads selectivity study and would require extensive linker optimization to gain activity on any of the targets with which they interacted. In addition, **5-a** showed poor cell permeability.

The inactivity of **6-c** was expected as the absence of the terminal amide at the linker attachment point prevented efficient binding to VHL. Inefficient cell penetration, as shown by the NanoBRET data, was also likely the reason for the lack of degradation activity of PROTAC **4-b.**

Interestingly, none of the PROTACs based on the VH032-OH exit vector, i.e., **4-d**, **5-b**, and **6-e**, induced degradation of kinases, suggesting that the central position on the VHL032 ligand is not efficient for PROTAC design for the targeted kinases. PROTAC **6-e** was additionally profiled at 0.25 and 2.5 μM (Figure S13), respectively, to rule out an early onset of hook effects or too low PROTAC concentrations of this particular PROTAC, but no significantly degraded kinases were detected.

PROTACs **6-a** and **6-d**, which strongly induced cytotoxicity at concentrations below 1 μM, were used to elucidate how cytotoxicity triggered downregulation of targets and can be mistakenly attributed to PROTAC-induced kinase degradation. Indeed, both PROTACs induced the degradation of a significant number of kinases, and we speculated that at least some of this degrader activity was caused by cytotoxicity. This hypothesis was further supported by the observed lower protein levels of numerous nonkinase proteins. Nevertheless, PROTACs **6-a** and **6-d** likely also mediated strong VHL-dependent kinase degradation.

**Figure 6:**
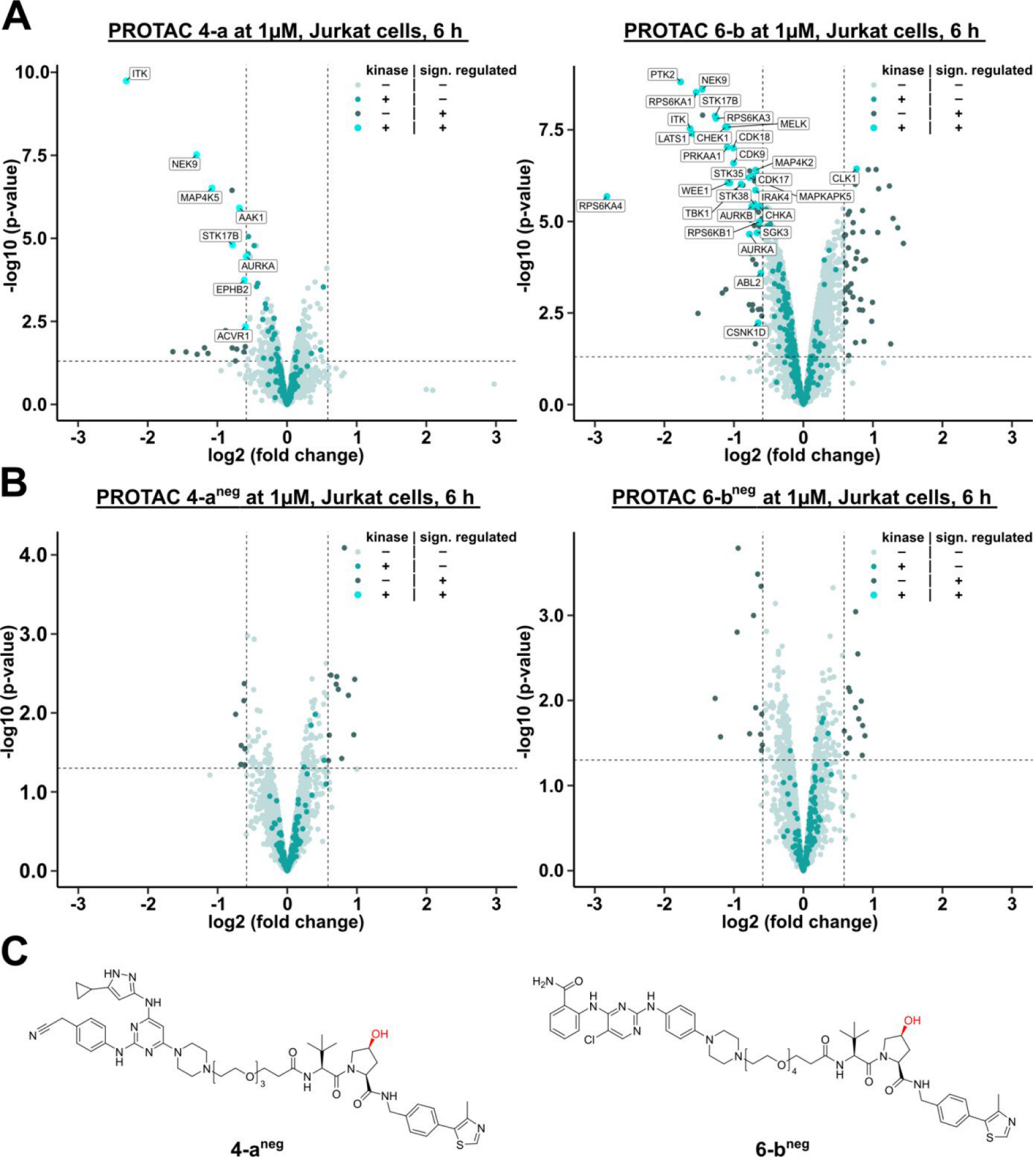
PROTACs **4-a** and **6-b** induce VHL-dependent degradation of multiple kinases across the entire kinome. Jurkat cells were treated with 1 μM of PROTAC for 6 h. Nonkinase proteins that showed no significant changes in expression levels are represented by light-blue dots; kinases that displayed no significant expression level alterations are represented by green dots; nonkinase proteins that exhibited significant changes in expression level are represented by dark gray dots; and kinases that were significantly up- or downregulated are represented by enlarged cyan dots and are labeled. (A) Volcano plots of PROTACs **4-a** and **6-b**. (B) Volcano plots of the negative controls **4-a**^**neg**^ and **6-b**^**neg**^. (C) Chemical structures of the negative controls **4-a**^**neg**^ and **6-b**^**neg**^ harboring the inactive hydroxyproline epimer of VH032-NH_2_ precluding interaction with VHL.

We selected PROTACs **4-a** and **6-b** to further investigate their VHL-dependent, kinome-wide degradation efficiencies in a dose-dependent manner by quantitative proteomics. Few kinases were degraded at 0.25 μM (Figure S14). Optimal activity was observed at 1 μM, whereas at a high concentration (2.5 μM), the number of degraded kinases was again lower, suggesting the onset of a hook effect. Cell line-specific effects were investigated by including the human T-lymphocyte-derived Jurkat cell line in our proteomic analysis, which is known to express a large number of kinases.^56^ Indeed, more kinases were degraded when treated with the two PROTACs compared with MCF-7 cells (Figure S11). This was particularly pronounced for PROTAC **6-b** (Figure 6A).

The observed differences could only be partially explained by cell type-specific kinase expression, but the most significantly degraded kinase in Jurkat cells, ITK, was indeed a T-cell-specific kinase (Table S2). The difference in VHL expression levels between Jurkat and MCF-7 cells (32.9 nTPM and 36.9 nTPM, respectively; extracted from https://www.proteinatlas.org(70)) was marginal and did not provide an explanation for the observed cell line-specific degradation efficacies.

In addition, efficient target engagement, as determined by Kinobeads assays, was not a strong predictor of degradation efficacy, suggesting that structural features of the linker region leading to efficient ternary complex formation and the degradability of the target are more important than on-target affinity (Table S3).

In order to confirm the VHL dependency of the observed kinase degradation, the corresponding negative controls **4-a**^**neg**^ and **6-b**^**neg**^ were synthesized, carrying the inactive hydroxyproline epimer at the VHL ligand (Figure 6C). Following the same evaluation workflow as for the other PROTACs (Figures S17–S21; Tables S4 and S5), **4-a**^**neg**^ and **6-b**^**neg**^ were analogously profiled in Jurkat cells. Gratifyingly, both negative controls were found to be indeed inactive confirming VHL-dependent degradation of these two PROTACs (Figure 6B).

After confirming that PROTACs **4-a** and **6-b** induced kinase degradation in a VHL-dependent manner, we tested both PROTACs in a subsequent series of orthogonal Western blot experiments to validate the data obtained from our proteomics analyses. We additionally evaluated the PROTACs in HiBiT assays to quantitatively determine target degradation by establishing stable HiBiT cell lines expressing tagged ITK^HiBiT^ and AURKA^HiBiT^, respectively, by using lentiviral transduction. These studies also included additional control experiments to further demonstrate a UPS dependence of the observed degradation.

**Figure 7:**
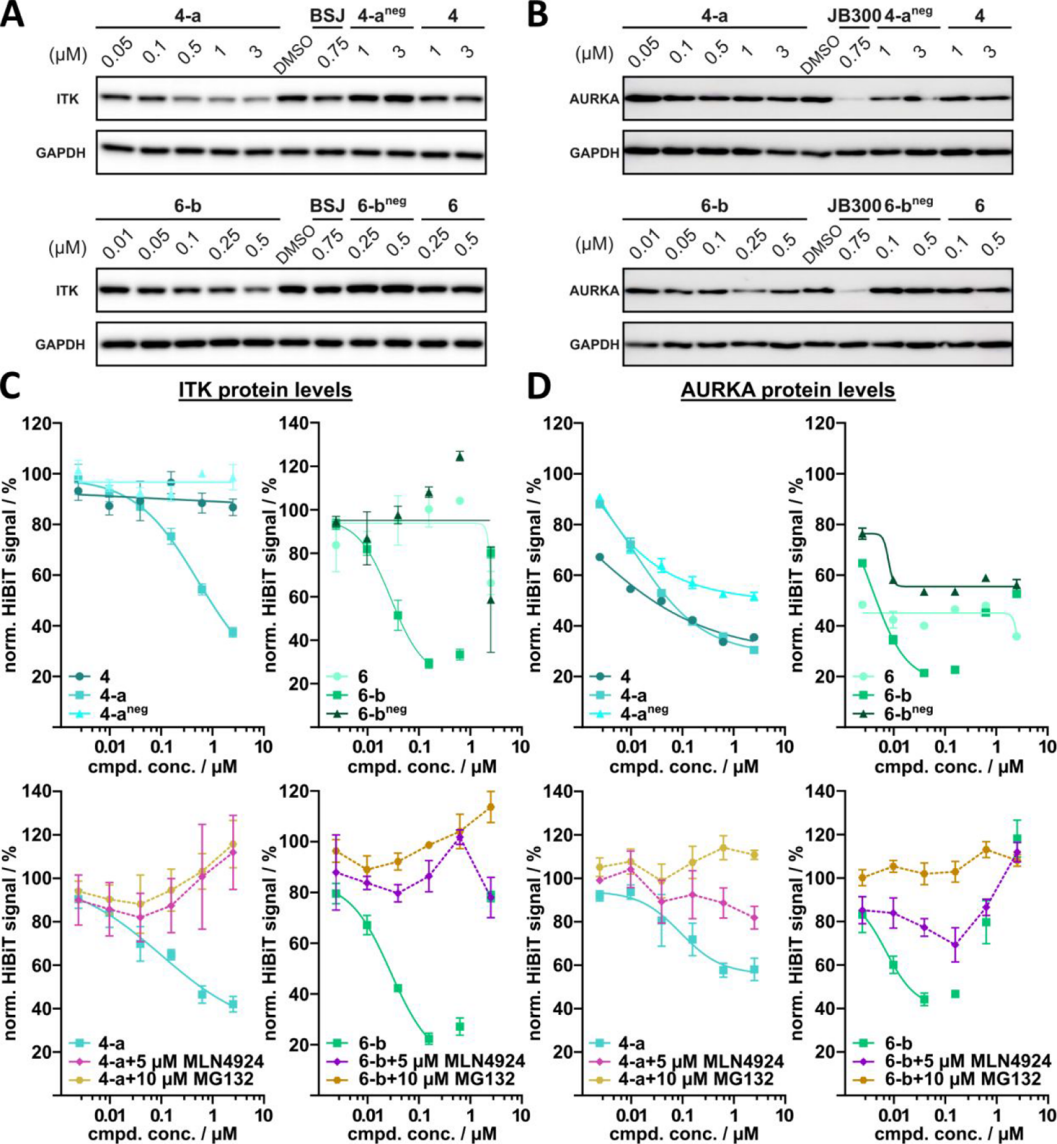
Validation of proteomic hits by Western blots and HiBiT experiments. (A,B) Western blots of Jurkat cells treated with different concentrations of the specified compounds for 6 h. ITK (A) and AURKA protein levels (B) were compared with Jurkat cells treated with DMSO. GAPDH was used as a loading control. (C,D) ITK (C) and AURKA (D) protein levels were based on luciferase measurements. MV4–11 cells, expressing tagged AURKA^HiBiT^ protein, and Jurkat cells, expressing tagged ITK^HiBiT^, were treated with different concentrations of the specified compounds for 6 h. Following cell lysis, the resulting lysates were complemented with the largeBiT fragment, and luciferase activity was measured. Top panel: the activity of PROTACs **4-a** and **6-b** was compared to that of their respective kinase parent inhibitors (**4** and **6**) and their corresponding negative controls (**4-a**^**neg**^ and **6-b**^**neg**^). Bottom panel: cotreatment experiments of PROTACs **4-a** and **6-b** were conducted in the presence of either the neddylation inhibitor MLN4924 or the proteasome inhibitor MG132, respectively.

We selected significantly depleted ITK and marginally depleted AURKA as representative kinases to confirm the degradation observed in our proteomic experiments in Jurkat cells and to justify the set cutoff levels (log2(fold change) ≥ 0.60). The recently published PROTACs BSJ-05–037^23^ and JB300^21^ were used as positive controls for ITK and AURKA, respectively. However, BSJ-05–037, which has not been investigated in Jurkat cells in the literature, demonstrated only weak degradation of ITK under the used treatment conditions (6 h) (Figure 7A,B).

Profiling in Western blot assays revealed a dose-dependent degradation of ITK induced by both PROTACs, **4-a** and **6-b**, in Jurkat cells after 6 h of treatment (Figure 7A). Consistent with the proteomic data, the corresponding negative controls **4-a**^**neg**^ and **6-b**^**neg**^ as well as the respective kinase parent inhibitors **4** and **6** did not cause any degradation of ITK. Determining the degradation levels in HiBiT experiments revealed that PROTACs **4-a** and **6-b** showed a DC_50_ of 937 nM and 42 nM, respectively, but did not degrade ITK beyond a *D*_max_ of 71% (Figure 7C, upper panel). The ectopic and stable expression of ITK^HiBiT^ in Jurkat cells shifted apparent DC_50_ and *D*_max_ values to high potency probably due to differences in expression levels compared to endogenous ITK and potentially also inefficient membrane integration of ITK^HiBiT^ rendering it more prone to degradation. In addition, a shallow dose response for PROTAC **4-a** was revealed, not reaching *D*_max_ at the highest concentration tested (2.5 μM). In agreement with our concentration-dependent proteomic experiments, a hook effect was observed for PROTAC **6-b** at >1 μM but not for **4-a**.

As expected, rescue experiments using cotreatment with neddylation (MLN4924) and proteasome (MG132) inhibitors rescued the observed ITK degradation confirming UPS-dependent and PROTAC-mediated target degradation (Figure 7C, lower panel).

PROTACs **4-a** and **6-b** both induced degradation of AURKA in proteomic experiments in Jurkat cells, with degradation levels close to the set cutoff (log2(fold change) ≥ 0.60). As expected, analogous profiling in Western blot assays showed only a weak reduction in AURKA levels by either PROTAC (Figure 7B). In contrast, the established AURKA degrader JB300, used as a positive control, showed a strong degradation. The negative controls **4-a**^**neg**^ and **6-b**^**neg**^ as well as the kinase parent inhibitors **4** and **6** had no effect on AURKA levels. Despite the apparent low activity of PROTACs **4-a** and **6-b** in Western blot assays, both PROTACs surprisingly induced dose-dependent degradation of AURKA in HiBiT experiments which also revealed a hook effect for PROTAC **6-b** at high concentrations (Figure 7D, upper panel). Surprisingly, the HiBiT experiments further showed a weak reduction of AURKA levels induced by both the negative controls **4-a**^**neg**^ and **6-b**^**neg**^ as well as the kinase parent inhibitors **4** and **6**, albeit to a lesser extent. Rescue experiments using cotreatment with the proteasome inhibitor (MG132) showed complete rescue of **4-a**- and **6-b**-mediated AURKA degradation. However, analogous experiments using the neddylation inhibitor (MLN4924) yielded only partial rescue of AURKA levels, suggesting that a secondary, neddylation-independent mechanism was responsible for the observed AURKA degradation. AURKA levels are strongly controlled by the cell cycle, and centrosome-associated AURKA has been shown to be resistant to PROTAC-mediated degradation.^71^ Small discrepancies in the degradation efficacy between the ectopically expressed AURKA^HiBiT^ and endogenous AURKA levels may therefore be the result of an altered cellular localization, resulting in enhanced accessibility to the PROTAC-mediated degradation of AURKA^HiBiT^. In addition, the strong cell cycle dependency of AURKA levels combined with its ectopic overexpression may present AURKA^HiBiT^ as a sensitive reporter of early-stage cytotoxic effects. The degradation efficiency for a specific kinase may also be influenced by the promiscuous nature of the presented PROTACs as a result of competition of multiple kinases for degradation, potentially reducing the amount of available-PROTAC-to-POI.

## Concluding Remarks

VHL and CRBN are the major E3 ligases currently being targeted for the development of target-specific degraders. The established toolbox of high-affinity ligands targeting these E3 ligases as well as chemical tools that are used to demonstrate VHL- and CRBN-dependent degradation make these two systems an ideal choice for PROTAC development, with the VHL system being especially attractive to generate degrader chemical probes for target validation. Here, we established a workflow for the validation of E3 ligase ligands using VHL-based promiscuous kinase PROTACs together with MS-based proteomics in a proof-of-principle study. This approach has the advantage that a single promiscuous kinase PROTAC can simultaneously target close to 100 kinases, drastically reducing the overall synthetic effort and enabling a comprehensive evaluation of an E3 ligase across a large target space. The study provides insight into the most suitable exit vectors on the VHL and POI ligands for target degradation. However, while also providing invaluable insight into the required compatibility between the interrogated E3 ligase and respective targets, functional linker/E3 ligase combinations for a given kinase target evaluated by a promiscuous kinase PROTAC will require additional optimization when transferred to a target-selective PROTAC. We envision that this process can be streamlined through automation of synthetic efforts as well as “direct-to-cell” strategies where PROTACs are synthesized in parallel and tested on cellular sensors such as HiBiT without prior time-consuming purification.^17,72,73^ The predictive value of a promiscuous targeting approach has been demonstrated by a recent study by the Fischer lab that mapped the degradable kinome using mainly CRBN targeting ligands.^54^ Indeed, selective PROTACs have been developed for many of the highly degraded kinases identified in that study.^74−78^

Kinome-wide screening data showed that the promiscuous kinase parent inhibitors we selected for this study potently inhibited more than 120 kinases, validating their linker exit vectors. Using the well-established E3 ligand VH032 and its derivative VH032-OH, we synthesized a set of structurally diverse PROTACs by combining distinct structural features in a modular fashion. Although target affinity did not seem to correlate well with PROTAC degradation, all PROTACs that showed poor cell penetration in our NanoBRET model were found to be inactive in proteomic analyses. By thoroughly evaluating cell viability effects, we identified cytotoxicity as the main cause of apparent PROTAC-independent POI degradation. Furthermore, PROTACs that were inactive under nontoxic conditions induced degradation at higher PROTAC concentrations. We also showed recently that commonly used control compounds, such as proteasome and neddylation inhibitors, can induce PROTAC-independent degradation, highlighting the importance of considering general toxicity in the evaluation workflow.^30^ It is interesting to note that many PROTACs based on new E3 ligase ligands developed in the literature have been used at high (>10 μM) PROTAC concentrations, frequently employing the panBET inhibitor JQ1, which causes toxicity in most cell lines at low concentrations. Typically, neither negative controls that are inactive for the targeted E3 ligase nor tool compounds preventing E3 ligase activation are available for these E3 ligases. In our study, we used the parent inhibitors as controls, in addition to VHL-inactive stereoisomers, demonstrating that the promiscuous kinase parent inhibitors had only marginal effects on kinase expression levels when used at low (nontoxic) concentrations and short (6 h) treatment times. We also demonstrated how the degradation efficacy can be cell line-dependent and highlighted the necessity and relevance of orthogonal hit validation assays.

Employing a well-validated E3 ligase ligand in this study may present a potential limitation of our workflow, as many E3 ligases that could be amenable to ligand design are functionally poorly characterized. This lack of data may signify that the activation mechanism remains unknown or that the ubiquitination reactions catalyzed by the targeted E3 ligases do not result in protein degradation but serve a different function within the ubiquitin system. Thus, if using our proposed strategy of engaging many targets through promiscuous POI ligands yields only inactive PROTACs, meticulous follow-up studies on E3 ligase function would need to be integrated into the workflow. However, we hope that the data presented in this study will lead to a streamlined approach to assessing the potential of E3 ligase ligands for degrader development, which includes the proposed evaluation steps as well as a thorough investigation of degrader toxicity.

## Data Availability

Label-free quantitative proteomics (only performed for kinase parent inhibitors **4**–**6** in Jurkat cells): the mass spectrometry proteomics data and complete MaxQuant search results have been deposited to the ProteomeXchange Consortium (http://www.proteomexchange.org/) via the MassIVE partner repository with the data set identifier MSV000095896. TMT-labeled quantitative proteomics: the mass spectrometry proteomics data have been deposited to the ProteomeXchange Consortium via the PRIDE partner repository with the data set identifier PXD057431.

## Supporting Information Available

Additional assay data including a DSF heatmap, NanoBRET dose–response data, volcano plots of all MS experiments, HiBiT data as well as additional experimental procedures, and full compound characterization data containing spectra (^1^H and ^13^C NMR, LCMS, and HRMS) (PDF)
DSF shift values (XLSX)
Kinobeads profiling (XLSX)
MS proteomics data for parent inhibitors in Jurkat cells (XLSX)
MS proteomics data (XLSX)
