# Supplementary material for: Workflow for E3 Ligase Ligand Validation for PROTAC Development
Source: ACS Chem Biol. 2025 Feb 11;20(2):507–21. doi: 10.1021/acschembio.4c00812 (PMC11851430; doi:10.1021/acschembio.4c00812)
Supplement: Supplementary file 1 — cb4c00812_si_001.pdf [file cb4c00812_si_001.pdf]

# - Supporting Information -

A workflow for E3 ligase ligand validation for PROTAC development

Nebojša Miletić[1,2,#], Janik Weckesser[1,2,#], Thorsten Mosler[3], Rajeshwari Rathore[3], Marina E. Hoffmann[3], Paul Gehrtz[4], Sarah Schlesiger[4], Ingo V. Hartung[4], Nicola Berner[5,6], Stephanie Wilhelm[5], Juliane Müller[7], Bikash Adhikari[7], Václav Němec[1,2], Saran Aswathaman Sivashanmugam[1,2], Lewis Elson[1,2], Hanna Holzmann[1,2], Martin P. Schwalm[1,2], Lasse Hoffmann[1,2], Kamal Rayees Abdul Azeez[1,2], Susanne Müller[1,2], Bernhard Kuster[5,6], Elmar Wolf[7], Ivan Đikić[3], Stefan Knapp[1,2,8,*]

[1]Institute of Pharmaceutical Chemistry, Goethe University, Max-von-Laue-Str. 9, 60438 Frankfurt am Main, Germany

[2]Structural Genomics Consortium (SGC), Buchmann Institute for Life Sciences, Max-von-Laue-Str. 15, 60438 Frankfurt am Main, Germany

[3]Institute of Biochemistry II, School of Medicine, Goethe University Frankfurt, Frankfurt am Main 60590, Germany

[4]Medicinal Chemistry, Global Research & Development, Merck Healthcare KGaA, 64293 Darmstadt, Germany

[5]Chair of Proteomics and Bioanalytics, Technical University of Munich, Emil-Erlenmeyer-Forum 5, 85354 Freising, Germany

[6]German Cancer Consortium (DKTK), partner site Munich, a partnership between DKFZ and University Center Technical University of Munich, Germany

[7]Institute of Biochemistry, University of Kiel, Rudolf-Höber-Str. 1, 24118 Kiel, Germany[8]German Cancer Consortium (DKTK) site Frankfurt/Mainz

*N.M. and J.W. contributed equally to this study

*Correspondence: knapp@pharmchem.uni-frankfurt.de

Methods

DSF Assay

Recombinant protein kinase domains at a concentration of 2 µM were mixed with a 20 µM compound solution in DMSO, 20 mM HEPES, pH 7.5, and 500 mM NaCl. SYPRO Orange (5000×, Invitrogen) was added as a fluorescence probe (1 µl per mL). Subsequently, temperature-dependent protein unfolding profiles were measured using the QuantStudio™ 5 realtime PCR machine (Thermo Fisher, Waltham, MA, USA). Excitation and emission filters were set to 465 and 590 nm. The temperature was raised with a step rate of 3 °C per minute. Data points were analyzed with the internal software (Thermo Fisher, Protein Thermal Shift™ Software v1.4) using the Boltzmann equation to determine the inflection point of the transition curve. Differences in melting temperature are given as ΔT_m values in °C. Measurements were performed in duplicates.

Kinobeads selectivity profiling of PROTACs

Cell culture. Human Jurkat cells (Clone E6.1, ATCC TIB-152) were cultured in RPMI-1640 containing 10% Fetal Bovine Serum (FBS) at 37 °C and 5% CO_2. Culture medium was refreshed every 2 – 3 days and cells were kept at densities between 0.5 – $2{\cdot}10^6$ cell/mL until lysis.

Cell line authentication was accomplished by single nucleotide polymorphism (SNP) profiling (Multiplexion, Heidelberg, Germany).

Kinobeads pulldowns. Kinobeads pulldown experiments were performed as previously described.[1–3] Briefly, cells were lysed in 0.8% NP40, 50 mM Tris-HCl pH 7.5, 5% glycerol, 1.5 mM $MgCl_2$, 150 mM NaCl, 1 mM Na_3VO_4, 25 mM NaF, 1 mM DTT, protease inhibitors (SigmaFast, Sigma) and phosphatase inhibitors (prepared in-house according to Phosphatase inhibitor cocktail 1, 2 and 3 from SigmaAldrich). The cell lysate was ultracentrifuged and diluted with 50 mM Tris-HCl pH 7.5, 5% glycerol, 1.5 mM $MgCl_2$, 150 mM NaCl, 1 mM Na_3VO_4, 25 mM NaF, 1 mM DTT, protease inhibitors and phosphatase inhibitors to a final concentration of 5 mg/ml as determined by Bradford assay. For selectivity profiling of compounds the cell lysate (2.5 mg of total proteins per pulldown) was pre-incubated with increasing compound concentrations (DMSO vehicle, 3 nM, 10 nM, 30 nM, 100 nM, 300 nM, 1000 nM, 3 µM and 30 µM) for 45 min at 4 °C in an end-over-end shaker. Subsequently, lysates were incubated with Kinobeads epsilon (17 µL settled beads) for 30 min at 4 °C. To assess the degree of protein depletion from the lysates by Kinobeads, the flow-through of the DMSO control was recovered

for a pulldown of pulldown experiment where the lysate was incubated a second time with fresh Kinobeads. Beads were washed in 3 steps with buffer containing 0.4%, 0.2% and no NP40. Bound proteins were reduced with 50 mM DTT in 8 M Urea, 40 mM Tris-HCl pH 7.4 for 30 min at room temperature. After alkylation with 55 mM chloroacetamide (CAA), the Urea concentration was reduced to 1 – 2 M and proteins were digested using trypsin. Acidified peptides were desalted and concentrated using SepPak tC18 µEluation plates (Waters). Samples were frozen, dried by vacuum centrifugation and stored at -20 °C.

LC-MS^2 analysis. Peptides were measured on a Dionex Ultimate3000 nano HPLC coupled online to an Orbitrap Fusion Lumos (Thermo Fisher Scientific) mass spectrometer.

Peptides were dissolved in 0.1% formic acid and delivered to a trap column (ReproSil-pur C18-AQ, 5 µm, Dr. Maisch, 20 mm × 75 µm, self-packed) at a flow rate of 5 µL/min in LC solvent A (0.1 % formic acid).[4] After 10 min of loading, peptides were transferred to an analytical column (ReproSil Gold C18-AQ, 3 µm, Dr. Maisch, 400 mm × 75 µm, self-packed) and separated using a 70 min linear gradient from 4% to 32% LC solvent B (0.1% formic acid, 3 % DMSO in acetonitrile) in LC solvent A.

The Orbitrap Fusion Lumos was operated as follows: positive polarity; spray voltage 2.1 kV, capillary temperature 275 °C.

A full-scan (MS1) was recorded from 360 to 1300 m/z with a resolution of 60,000 in the Orbitrap in profile mode. The MS1 AGC target was set to 4e5 and the maxIT was set to 50 ms, RF lens value was set to 50%. Based on the full scans, precursors were targeted for the MS/MS scans (MS^2) if the isotope envelope was peptidic (MIPS), the charge was between 2 and 6 and the intensity exceeded 2.5e4. The dynamic exclusion was set to 30 sec and exclude isotopes was switched on. The MS2 quadrupole isolation window was set to 1.7 m/z. Peptide fragmentation occurred in the ion routing multipole (IRM) by HCD with a fixed collision energy mode, the collision energy normalized to the precursor m/z and charge with a collision energy of 30%. The MS2 scan was acquired in the Orbitrap at a resolution of 15,000 in centroid mode and a defined first mass of 100 m/z. The AGC value was set to 2e5 at a maximum IT of 75 ms.

Peptide and protein identification and quantification. Peptide and protein identification and quantification was performed using MaxQuant[5] (version 1.6.12.0) by searching the MS^2 data against all canonical protein sequences as annotated in the UniProt reference database (human proteins only, downloaded 24.08.2020) using the search engine Andromeda.[6] Carbamidomethylated cysteine was set as fixed modification and phosphorylation of serine, threonine and tyrosine, oxidation of methionine and N-terminal protein acetylation as variable

modifications. Trypsin/P was specified as the proteolytic enzyme and up to two missed cleavage sites were allowed. Label-free quantification[7] and match between runs were enabled within MaxQuant.

Data analysis. For Kinobeads competition binding assays, relative binding was calculated based on the LFQ intensity ratio to the DMSO control for every single inhibitor concentration. EC_{50} values were derived from a four-parameter log-logistic regression using an internal pipeline that utilizes the 'drc' package in R. Multiplying the EC_{50} values with a correction factor (cf) results in an apparent binding constant (K_d^{app}). The correction factor for a protein is defined as the ratio of the amount of protein captured from two consecutive pulldowns of the same DMSO control lysate.[2] Targets of the inhibitors were annotated manually. A protein was considered a target if the resulting binding curve showed a sigmoidal curve shape with a dose dependent decrease of binding to the beads. Additionally, the number of unique peptides and MSMS counts per condition were taken into account. Protein intensity in the DMSO control sample was also included in the target annotation process. A protein was considered as a direct binder if annotated in Uniprot.org as protein kinase, lipid kinase, nucleotide binder, helicase, ATPase and GTPase as well as FAD and heme containing proteins.

NanoBRET® Assay

Genes encoding full-length AAK1 (Promega, NV1001), Aurora A (Promega: NV1041) and VHL (Promega: N2741) proteins respectively cloned in frame with the NanoLuc fusion tag were transfected into HEK293T cells using FuGENE HD (Promega: E2312) following the manufacturer's protocol and proteins were allowed to express for 20 hours at 37 °C and 5% CO_2. After trypsinization and resuspending in Opti-MEM (Life Technologies: 31985070), 10 µL of the transfected cells were pipetted into each well of a 384-well plate (Greiner: 781207) at a cell density of $2 \cdot 10^5$ cells/mL. Compounds were pipetted at various concentrations using an Echo acoustic dispenser (Labcyte), immediately followed by Tracer K10 (Promega, Tracer DB ID: T000008) for AAK1 and Aurora A and VHL Tracer (Promega, Tracer DB ID: T000019) for VHL, at an optimum K_D concentration chosen from TracerDB (tracerdb.org), respectively. The system was allowed to equilibrate for 2 hours at 37 °C and 5% CO_2 prior to BRET measurements. To measure the BRET signal, the NanoBRET® NanoGlo Substrate (Promega: N1573) was added as per the manufacturer's protocol, and filtered luminescence was measured on a PHERAstar plate reader (BMG Labtech) equipped with a luminescence filter pair (450 nm BP filter (donor) and 610 nm LP filter (acceptor)). For NanoBRET® measurement in permeabilized cells, 25 nL of digitonin (0.05 µg/µL) was pipetted into each well using an

Echo acoustic dispenser (Labcyte) prior to incubating the plate for 5 minutes at 37 °C and 5% CO_2. After incubation, the BRET signals were measured by a PHERAstar plate reader (BMG Labtech) following the same procedure as intact NanoBRET® measurement. Competitive displacement data were then normalized to controls and were plotted using GraphPad Prism 9 software employing a normalized 3-parameter curve fit with the following equation: $Y = 100/(1 + 10^{X - \log IC50})$.

Cell Titre Glow® Assay

The effects of the compounds on cell viability was determined using the CellTiter-Glo® 2.0 Cell Viability Assay (Promega: G9241) following manufacturer's protocol. Ten µL of Jurkat cells at a cell density of $2 \cdot 10^5$ cells/mL were seeded into individual wells of a white 384-well plate (Greiner: 781207) and the cells were allowed to equilibrate for 1 hour at 37 °C and 5% CO_2. After equilibration, PROTACs were titrated at various concentrations using an Echo acoustic dispenser (Labcyte) and the cells were incubated for 24 hours at 37 °C and 5% CO_2. Equal volume of CellTiter-Glo® 2.0 reagent (10 µL) was added to each well and cells were incubated for 10 minutes at room temperature. Filtered luminescence was measured on a PHERAstar plate reader (BMG Labtech) and data was evaluated using GraphPad Prism 9 software employing a normalized curve it with the following equation: $Y=Bottom + (Top-Bottom)/(1+10^{(LogEC50-X) *HillSlope})$.

Label-free Quantitative Proteomics (Only Performed for Kinase Parent Inhibitors 4 – 6 in Jurkat Cells)

Sample preparation. Jurkat cells (ATCC© HTB-22) were cultured in RPMI (Thermo: 12633012) supplemented with 10% Foetal Bovine Serum (Thermo: 26140079) and 1% Penicillin-Streptomycin (Thermo: 15140122). Cells were seeded in 6 well plates (Greiner: 657160) at a cell density of $1 \cdot 10^6$ cells/well and left at 37 °C and 5% CO_2 overnight. The following day cells were treated with kinase parent inhibitors **4** – **6** at respective concentration for 6 h. Following treatment, cells were washed thrice with 1X DPBS (Thermo: 14190144) and 50 µl of SDS Lysis Buffer (2% SDS, 50 mM TRIS pH 8.5, 10 mM TCEP, 40 mM CAA) was added to each sample. Cells were transferred into low binding reaction tubes (Eppendorf: 0030108116) and samples were boiled for 15 minutes at 95 °C and sonicated for 2 minutes. In order to reduce viscosity, samples were sonicated using a sonication water bath (10 cycles, 15 sec sonication, 15 sec pause on ice), boiled at 95 °C for 10 min and trifluoroacetic acid was added to a final concentration of 1%. To neutralize the sample (final pH 7.6 – 8.0), 300 mM *N*-methylmorpholin was added to a final concentration of 2 %. Protein yield was determined by

Thermo Pierce BCA (bicinchoninic acid) protein assays. All steps were performed according to the manufacture's protocol.

Prior to tryptic digest, detergent was removed by SP3 clean-up, following the protocol first described by Hughes *et al.*[8] adapted to a BRAVO Agilent liquid handling platform. In short, lysate containing 200 µg of protein was mixed with 1 mg SP3 beads (50:50 mixture of Sera-Mag carboxylate-modified magnetic bead types A and B (Cytiva Europe)) in a 96-deep-well plate and proteins were precipitated onto the beads in 70% ethanol in ddH_2O (double distilled water). The beads were washed three times with 80% ethanol in ddH_2O and once with 100% acetonitrile. Disulfide bonds were reduced with 10 mM DTT (Dithiothreitol) for 45 min at 37 °C, followed by alkylation of cysteines with 55 mM CAA (2-chloroacetamide) for 30 min at room temperature in 100 µl of digestion buffer (2 mM $CaCl_2$ in 40 mM Tris-HCl, pH 7.8). Trypsin (1:50 (wt/wt) enzyme-to-protein ratio) was added and proteins were digested off the beads at 37 °C and 1200 rpm overnight. For peptide recovery, the beads were settled on magnets and the supernatant was transferred to a new 96-well plate. Beads were washed by addition of 100 µl 2% formic acid in ddH_2O and the supernatant was transferred to the collection plate. Subsequently, the samples were desalted as described below. Prior to LC-MS/MS analysis, samples were desalted using hydrophilic-lipophilic balanced (HLB; 10 mg *N*-Vinylpyrrolidon-Divinylbenzol porous particles 30 µm, MACHEREY-NAGEL) 96-well plates using centrifugation at 200 rpm for 1. For this, HLB material was primed with 500 µL of isopropanol, acetonitrile, and solvent B (0.1% formic acid in 70% acetonitrile in ddH_2O) and equilibrated with 1,000 µL of solvent A (0.1% formic acid in ddH_2O) prior to sample loading (by gravitation, 5 min). The sample flow-through was re-applied to the plate and bound peptides were washed with 1,000 µL of solvent A. Peptides were eluted with 250 µL of solvent B (3 min, 200 rpm; 1 min, 1,000 rpm). Samples were frozen at -80 °C, dried by vacuum centrifugation and stored at -20 °C until LC-MS/MS measurement.

LC-MS^2 analysis. A micro-flow LC-MSMS setup with a Q Exactive HF-X mass spectrometer (Thermo Fisher Scientific) was used as described in detail in previous publications.[9,10]

50 µg of peptides dissolved in 0.1% formic acid were directly injected onto the microflow LC system. Online chromatography was performed using a commercially available Thermo Fisher Scientific Acclaim PepMap 100 C18 LC column (2 µm particle size, 1 mm ID × 150 mm; catalog number: 164711). Column temperature was maintained at 55 °C using the integrated column oven. Peptides were delivered at a flow rate of 50 µl/min and separated using a two-step linear gradient (120 min) ranging from 1 – 24% (105 min) and 24 – 35% (15 min) of LC solvent B (0.1% formic acid, 3% DMSO in acetonitrile) in LC solvent A (0.1% formic acid, 3% DMSO).[4] The Q Exactive HF-X was operated as follows: positive polarity; spray voltage 4 kV, capillary

temperature 320 °C; vaporizer temperature 200 °C. The flow rates of sheath gas, aux gas and sweep gas were set to 40, 3, and 0, respectively. TopN was set to 50. Full MS was readout in the orbitrap, resolution was set to 120,000 and the mass range was set to 360 – 1300. Full MS AGC target value was 3E6 with a maximum IT of 100 ms and RF lens value was set to 40. Peptide match was set to preferred and default charge state was set to 2. The dynamic exclusion duration was set to 40 s and exclude isotopes was switched on. For readout of MS^2 spectra, orbitrap resolution was set to 15,000 and the mass range was set to 200 – 2000. The isolation width was set to 1.3 m/z, the first mass was fixed at 100 m/z, NCE was 28. The AGC target value was set to 1E5 at a maximum IT of 22 ms.

Peptide and protein identification and quantification. Protein and peptide identification and quantification was performed using MaxQuant[5] (version 1.6.17.0) by searching the MS^2 data against all canonical protein sequences as annotated in the UniProt reference database (human proteins only, downloaded 24.08.2020) using the search engine Andromeda[6]. Carbamidomethylated cysteine was set as fixed modification; oxidation of methionine and N-terminal protein acetylation were set as variable modification. Trypsin/P was specified as proteolytic enzyme and up to two missed cleavage sites were allowed. The minimum peptide length was set to seven and all data were adjusted to 1% peptide-spectrum-match (PSM) and 1% protein false discovery rate (FDR). LFQ based quantification was enabled including the match between runs option.

Proteome data analysis. Data analysis was performed using Perseus (v 2.0.3.0) and RStudio (R version 3.6.3 (2020-02-29)) on identified and quantified protein groups as provided in the proteinGroups.txt file. Proteingroups.txt was filtered for contaminants and reverse hits, and median centric normalization and log2 transformation were performed. Entries were filtered for less than two missing values in each condition. A two-sided students T-test was performed for all proteins. P-values were corrected for multiple hypothesis testing using the FDR approach (fdr 0.05, 250 randomization, s0 0.01).

TMT-labelled Quantitative Proteomics

Cell Treatments and Sample preparations for MS

Lysis, Reduction and Alkylation. MCF-7 cells (ATCC© HTB-22) were cultured in DMEM (Thermo: 11965084) supplemented with 10% Foetal Bovine Serum (Thermo: 26140079) and 1% Penicillin-Streptomycin (Thermo: 15140122). Jurkat cells (ATCC© CRL-2899) were

cultured in RPMI (Thermo: 11875093) supplemented as mentioned above. Cells were seeded in 6 well plates (Greiner: 657160) at a cell density of $1 \cdot 10^6$ cells/well and left at 37 °C and 5% CO_2 overnight. The following day cells were treated with PROTACs at according concentration for 6 h. Following treatment, cells were washed thrice with 1X DPBS (Thermo: 14190144) and 50 µl of SDS Lysis Buffer (2% SDS, 50 mM TRIS pH 8.5, 10 mM TCEP, 40 mM CAA) was added to each sample. Cells were transferred into low binding reaction tubes (Eppendorf: 0030108116) and samples were boiled for 15 minutes at 95 °C and sonicated for 2 minutes.

Methanol/Chloroform Precipitation. 200 µl of methanol and 50 µl of chloroform were added to each sample reaction tube and mixed via vortex. 150 µl of milliQ-H_2O were added and samples were vortexed further. Samples were centrifuged for 5 minutes at 15000 g and the aqueous layer above the interphase was removed. 150 µl of methanol was added, samples were vortexed and centrifuged for 10 minutes at 15000 g to precipitate the pellet. The aforementioned wash procedure was repeated twice before transferring the pellet to a new low bind reaction tube in which it was allowed to dry for 5 minutes.

Dissolving the Protein Pellet and Protein Quantification. 50 µl of digestion buffer (8 M Urea, 50 mM TRIS pH 8) was added to each sample before vortexing and shaking at 750 rpm at room temperature for 10 minutes to solubilize the pellet. Protein quantification via Pierce™ BCA Protein Assay Kit (Thermo: 23225) was carried out according to manufacturer's protocol. Digestion buffer was added to reach a final concentration of 1.5 mg/ml.

Protein Digestion. Samples were diluted 1:2 with 50 mM TRIS pH 8.5 to reach a final concentration of 4 M Urea. Lys-C (Promega: VA1170) was added at a 1:100 enzyme/substrate ratio. Samples were agitated at 550 rpm and 37 °C for 3 h. Samples were further diluted with 50 mM TRIS pH 8.5 to a final concentration of 1 M Urea and Trypsin (Promega: V5280) was added at a 1:100 enzyme/substrate ratio. Samples were agitated overnight at 550 rpm and 37°C. The digestion was stopped the following day via addition of TFA to a final concentration of 1%.

Solid Phase Extraction via C18 Cartridges. Sep-Pak C18 Cartridges (Waters: WAT054955) were used for solid phase extraction of digested peptides. For preconditioning of C18 cartridges, 1 mL of methanol was added to each column followed by 1 mL of solution B (0.1% formic acid, 80% acetonitrile) and 1 mL of solution A (0.1% formic acid, ddH_2O) twice. Samples

were loaded to their respective columns and once samples had passed, columns were washed twice with solution A to remove unwanted components from the loading step. Elution of samples was carried out via introduction of 40% and 60% acetonitrile steps. Samples were dried and snap frozen in liquid nitrogen.

TMT-Labeling. Desalted peptides were dried and resuspended in TMT-labeling buffer (200 mM EPPS pH 8.2, 20% acetonitrile). 10 µg of peptides per condition were subjected to TMT labeling with 1:2.5 peptide TMT ratio (w/w) for 1 h at room temperature. The labeling reaction was quenched by addition of hydroxylamine to a final concentration of 0.5% and incubation at room temperature for 15 minutes. Successful TMT labeling was verified by mixing equimolar ratios of peptides and subjecting the mix to single shot LC-MS/MS analysis. For high pH reversed phase fractionation on a Dionex analytical HPLC, 50 µg of pooled and purified TMT labelled samples were resuspended in 10 mM ammonium-bicarbonate (ABC), 5% acetonitrile, and separated on a 250 mm long C18 column (Aeris Peptide XB-C18, 4.6 mm ID, 2.6 µm particle size; Phenomenex) using a multistep gradient from 100% solvent A (5% acetonitrile, 10 mM ABC in water) to 60% solvent B (90% acetonitrile, 10 mM ABC in water) over 70 minutes. Eluting peptides were collected every 45 seconds into a total of 96 fractions, which were cross-concatenated into 24 fractions and dried in a vacuum concentrator and resuspended in 3% acetonitrile with 0.1% TFA for LC-MS analysis.

Mass spectrometry data acquisition. Tryptic peptides were analyzed on an Orbitrap Lumos coupled to an easy nLC 1200 (ThermoFisher Scientific) using a 35 cm long, 75 µm ID fused-silica column packed in house with 1.9 µm C18 particles (Reprosil pur, Dr. Maisch), and kept at 50 °C using an integrated column oven (Sonation). HPLC solvents consisted of 0.1% formic acid in water (buffer A) and 0.1% formic acid and 80% acetonitrile in water (buffer B). Assuming equal amounts in each fraction, 500 ng of peptides were eluted by a non-linear gradient from 7% to 40% of buffer B over 90 minutes followed by a step-wise increase to 90% buffer B in 6 minutes which was held for another 9 minutes. A synchronous precursor selection (SPS) multi-notch MS3 method was used in order to minimize ratio compression as previously described.[11] Full scan MS spectra (350-1400 m/z) were acquired with a resolution of 120,000 at m/z 200, maximum injection time of 100 ms and AGC target value of $4 \cdot 10^5$. The most intense precursors with a charge state between 2 and 6 per full scan were selected for fragmentation ("Top Speed" with a cycle time of 1.5 seconds) and isolated with a quadrupole isolation window of 0.7 Th. MS2 scans were performed in the Ion trap (Turbo) using a maximum injection time of 50 ms, AGC target value of $1.5 \cdot 10^4$ and fragmented using CID with a normalized collision energy

(NCE) of 35%. SPS-MS3 scans for quantification were performed on the 10 most intense MS2 fragment ions with an isolation window of 0.7 Th (MS) and 2 m/z (MS2). Ions were fragmented using HCD with an NCE of 50% and analyzed in the Orbitrap with a resolution of 50000 at m/z 200, scan range of 100 – 500 m/z, AGC target value of $1.5 \cdot 10^5$ and a maximum injection time of 86 ms. Repeated sequencing of already acquired precursors was limited by setting a dynamic exclusion of 60 seconds and 7 ppm and advanced peak determination was deactivated. All spectra were acquired in centroid mode.

Mass spectrometry data analysis. MS raw data were analyzed using MaxQuant (2.4.2.0).[5] Acquired spectra were searched against a database containing 20,606 unique human protein sequences (Taxonomy ID 9606) downloaded from UniProt (released 02-2023) and a collection of common contaminants using the Andromeda search engine integrated in MaxQuant.[6] Identifications were filtered to obtain false discovery rates (FDR) below 1% for both - peptide spectrum matches (PSM; minimum length of 7 amino acids) and proteins - using a target-decoy strategy.[12] Spectra were searched with a mass tolerance of 6 ppm in MS mode, 20 ppm in HCD MS2 mode, strict trypsin specificity, and allowing up to 2 miscleavages. Carbamidomethylated cysteine was set as a fixed modification and oxidation of methionine and *N*-terminal protein acetylation as variable modifications with allowing up to 5 modifications per peptide. Obtained data was further processed using the R Studio environment. Only proteins quantified in all replicates after standard filtering were used for statistical analysis. TMT channels were normalized using quantile normalization from the Limma package. Proteins were deemed significantly regulated using the moderated Limma t-test.[13]

Western-Blot Assay

Two mL of Jurkat cells were seeded into 6 well plates (Greiner) at a density of $5 \cdot 10^5$ cells/mL. The following day, cells were treated with compounds at according concentrations or DMSO for 6 hours and then lysed with 6 M Urea at 4 °C for 20 minutes. The lysates were centrifuged for 15 minutes at 16000 g and the supernatant was collected to perform the Western-Blot. Protein quantification was performed using nanodrop One. The SDS samples were prepared by adding 7 µL of 2.5x Laemmli buffer to 30 µg of protein from each cell lysate and then heated at 95 °C for 5 minutes. The samples were separated by a BIS-TRIS polyacrylamide gel (AURKA: 12% BIS-TRIS polyacrylamide gel, 200 V for 50 min; ITK: 10% BIS-TRIS polyacrylamide gel, 100 V for 1:45) followed by transfer to PVDF membranes (Millipore) at 350 mA for 140 minutes. The membranes were blocked using 5%wt/vol BSA (in TBS-T, 20 mM Tris-HCl, pH 7.5, 150 mM NaCl and 0.1% (vol/vol) Tween 20) at room temperature for 1 hour.

The blots were incubated with respective primary antibodies at 1:1000 dilution (Cell Signaling technologies: Aurora A (D3E4Q), ITK (2F12) and GAPDH (D4C6R)) at 4 °C overnight. The blots were washed with TBS-T thrice and incubated with 1:5000 dilution of horseradish peroxidase (HRP)-labelled respective secondary antibodies (Cell Signaling technologies, Anti-rabbit (7074S) and Anti-Mouse (7076S)) for 1 hour. The blots were washed with TBS-T thrice and then incubated with chemiluminescent HRP substrate (Bio-Rad) for 2 minutes. The blots were imaged and the signals were detected using the Amersham ImageQuant 800 (Cytiva).

HiBiT Assay

Cell culture. Human MV4-11 (male) and human Jurkat (male) cells were cultured in RPMI-1640 medium whereas human HEK293 (female) cells were cultured in DMEM medium at 37 °C in 5% CO_2. Both media were supplemented with 10% FBS and 1% penicillin/streptomycin.

Cloning. HiBiT-ITK construct was cloned by PCR amplification of vector containing full-length ITK. The PCR product was inserted into pRRL-PGK-HiBiT entry vector using AgeI/MluI sites. The following primers were used for cloning HiBiT-ITK; forward: CGCACCGGTATGAACAACTTTATCCTCCTGG, reverse: CGCGACGCGTTTAAAGTCCTGATTCTGCAATTTCAGC.

Cell line generation. The stable cell line MV4-11$^{AURORA-HiBiT}$ was generated as previously described.[14] Lentiviral infection was used to generate the stable Jurkat HiBiT cell line. Lentivirus was produced using plasmids psPAX2, pMD2.G, and HiBiT-ITK plasmid in HEK293 cells. Jurkat cells were infected with filtered virus supernatant and selected after 48 h of infection for the generation of the stable Jurkat$^{ITK-HiBiT}$ cell line.

HiBiT Measurements. HiBiT assay was performed as described previously.[14] Briefly, MV4-11 and Jurkat HiBiT cells were seeded and treated with compounds for the indicated time. Nano-Glo HiBiT Lytic Detection System (Promega) was used for the assay and luminescence was measured on a Tecan Spark Multiplate reader (Tecan). DC_{50}s were calculated using the dose-response (four parameters) equation in Prism (GraphPad).

Assay Data

I. DSF Data

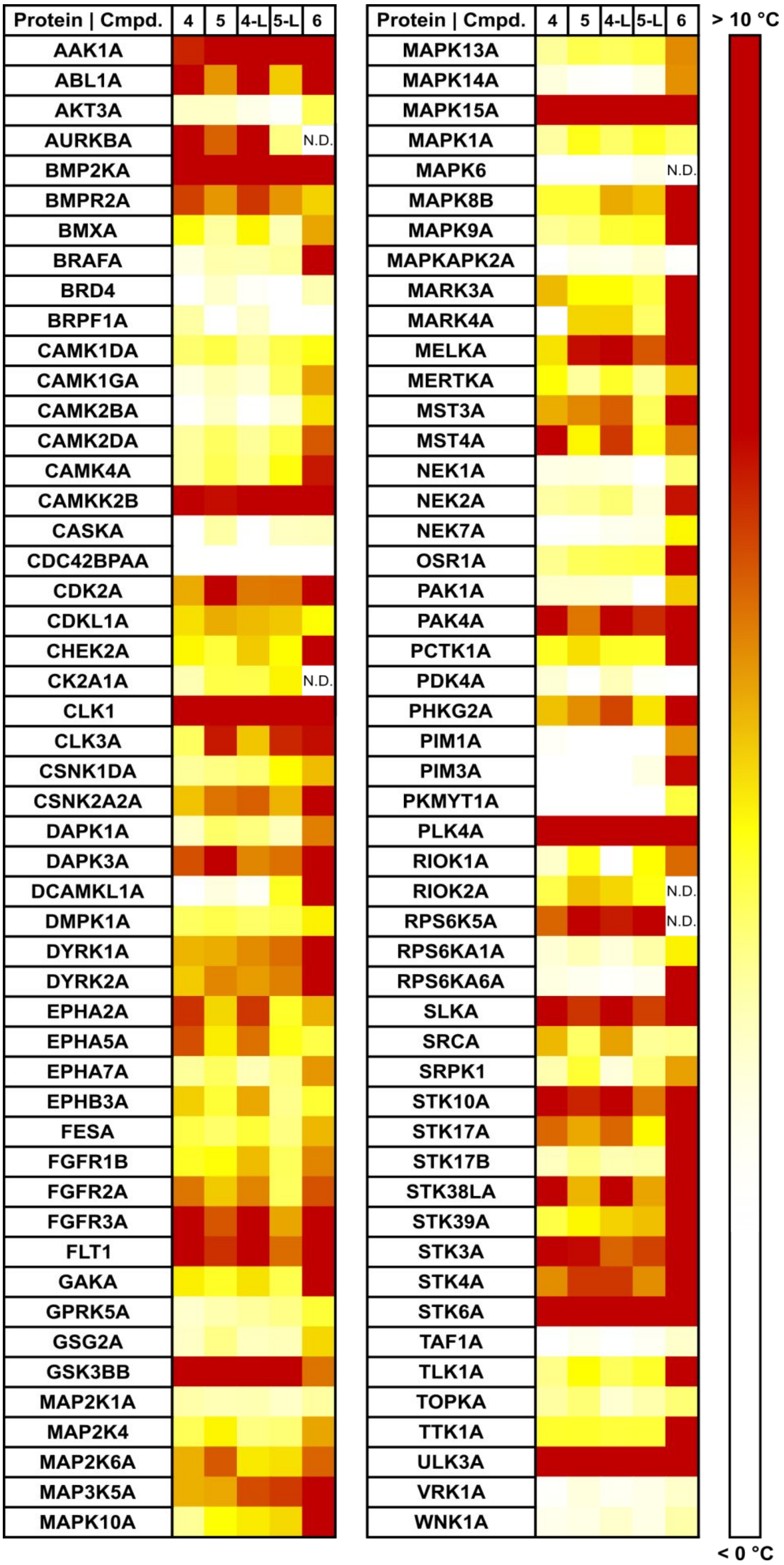

Figure S1: Selectivity Profiles of Kinase Parent Inhibitors and Linker Conjugates. Kinase parent inhibitors 4 – 6 and linker conjugates 4-L and 5-L were screened in a differential scanning fluorimetry (DSF) assay against a panel of 100 representative kinases.[15–17] The results are represented as a heat map ranging from no stabilization of the kinase (white) to highest stabilization of the kinase by a compound (red). The exact values of the Tm-shifts and used control compounds are listed in the supplementary file "Supplementary_Table_DSF_Shift_Values". N.D. = not determined

II. NanoBRET® Data

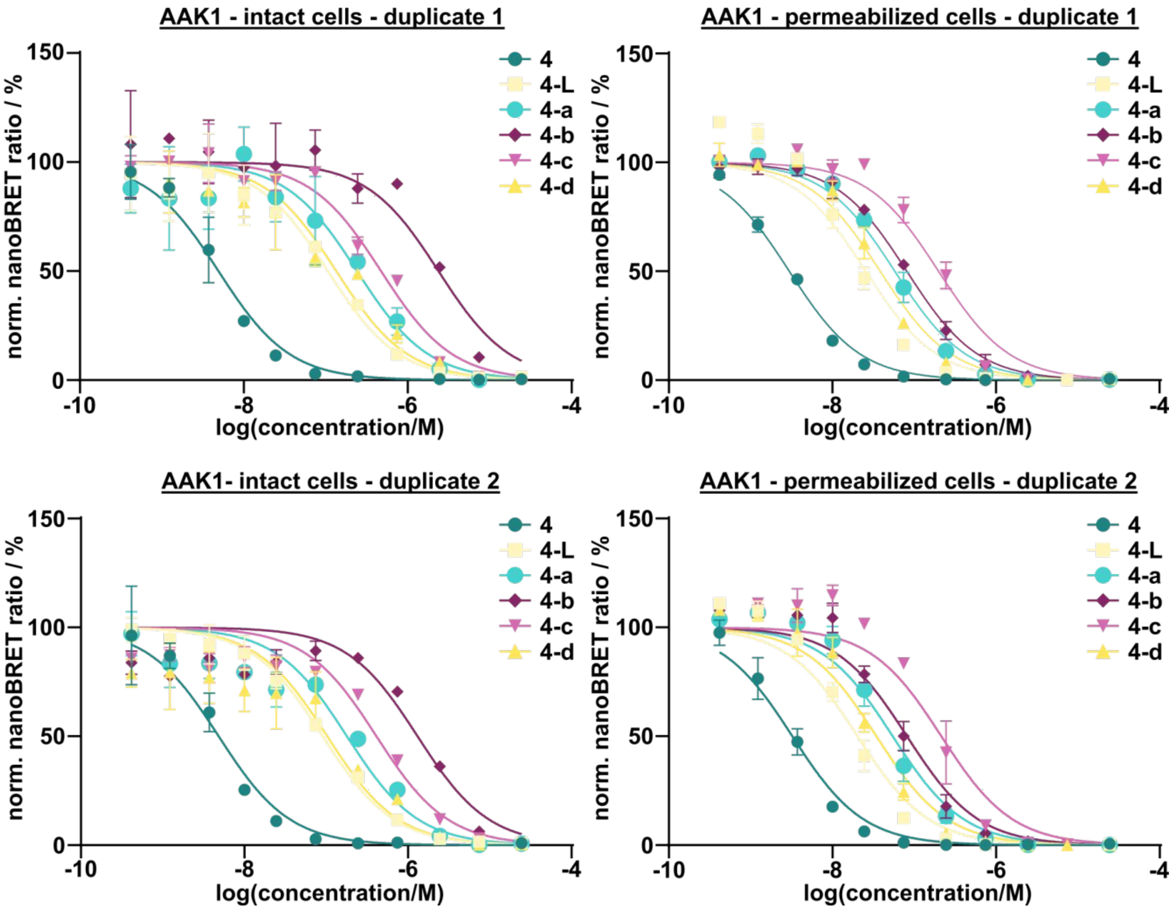

Figure S2: AAK1 engagement data of all compounds based on kinase parent inhibitor 4. NanoBRET® dose response curves of kinase parent inhibitor **4**, respective linker conjugate **4-L** and resulting promiscuous kinase PROTACs **4-d** measured in duplicates (top row: duplicate 1; bottom row: duplicate 2) against AAK1 in intact (left) and permeabilized (right) cells.

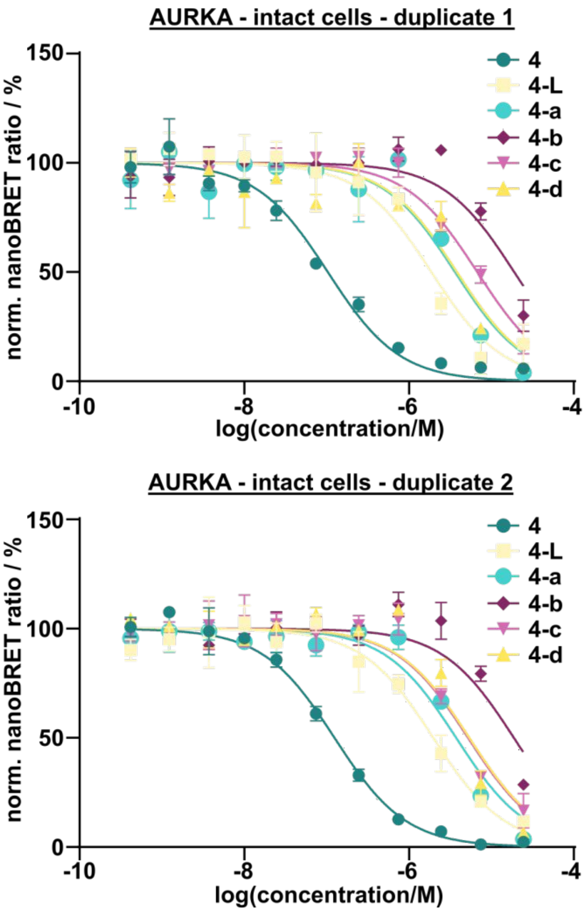

Figure S3: AURKA engagement data of all compounds based on kinase parent inhibitor 4. NanoBRET® dose response curves of kinase parent inhibitor **4**, respective linker conjugate **4-L** and resulting promiscuous kinase PROTACs **4-a** – **4-d** measured in duplicates (top row: duplicate 1; bottom row: duplicate 2) against AURKA in intact (left) and permeabilized (right) cells.

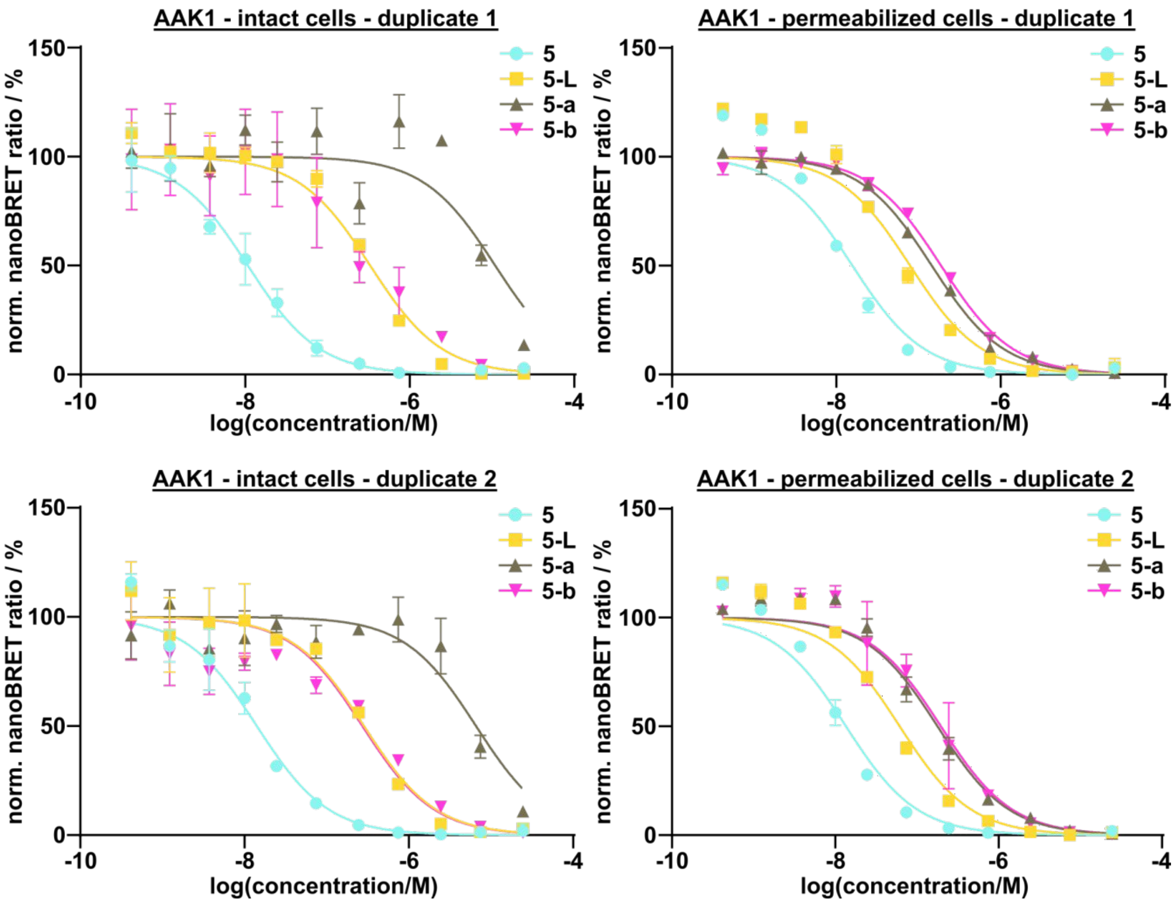

Figure S4: AAK1 engagement data of all compounds based on kinase parent inhibitor 5. NanoBRET® dose response curves of kinase parent inhibitor **5**, respective linker conjugate **5-L** and resulting promiscuous kinase PROTACs **5-a** and **5-b** measured in duplicates (top row: duplicate 1; bottom row: duplicate 2) against AAK1 in intact (left) and permeabilized (right) cells.

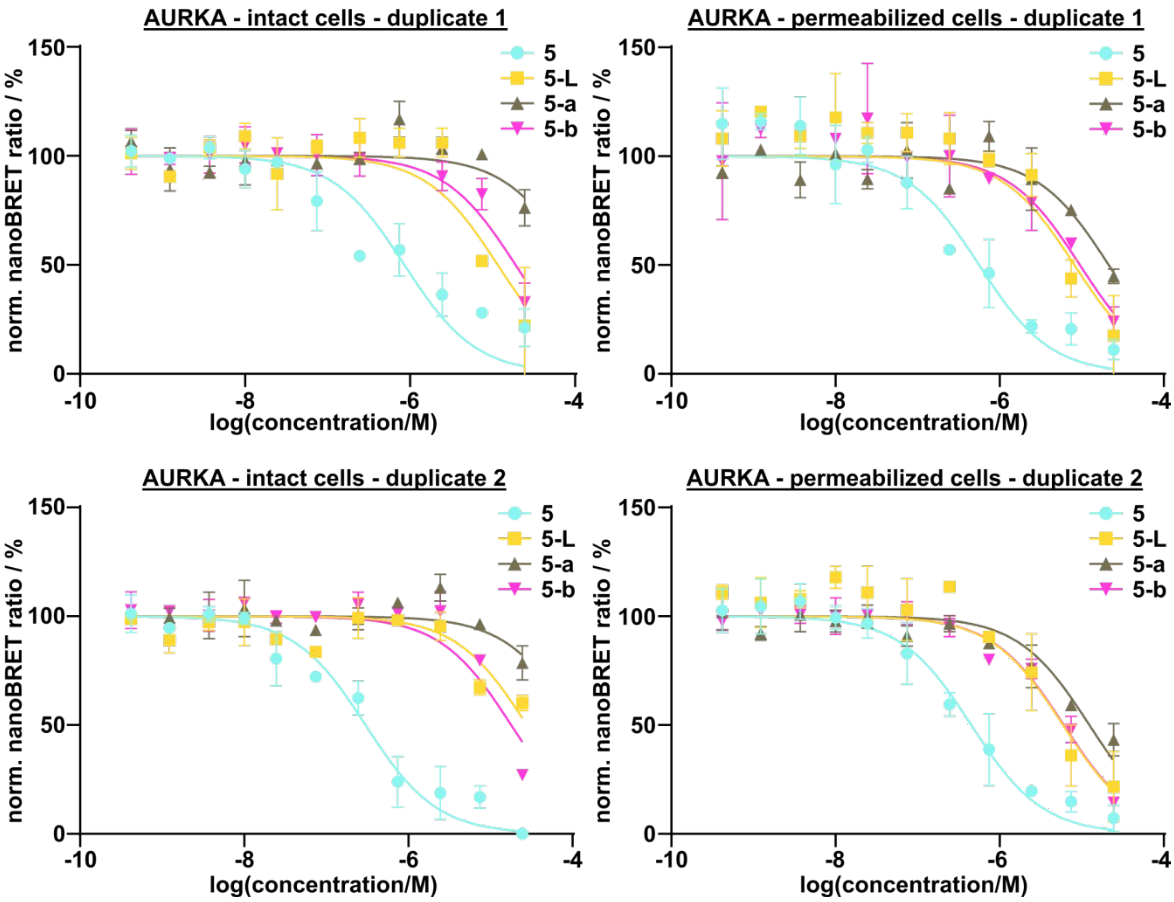

Figure S5: AURKA engagement data of all compounds based on kinase parent inhibitor 5. NanoBRET® dose response curves of kinase parent inhibitor **5**, respective linker conjugate **5-L** and resulting promiscuous kinase PROTACs **5-a** and **5-b** measured in duplicates (top row: duplicate 1; bottom row: duplicate 2) against AURKA in intact (left) and permeabilized (right) cells.

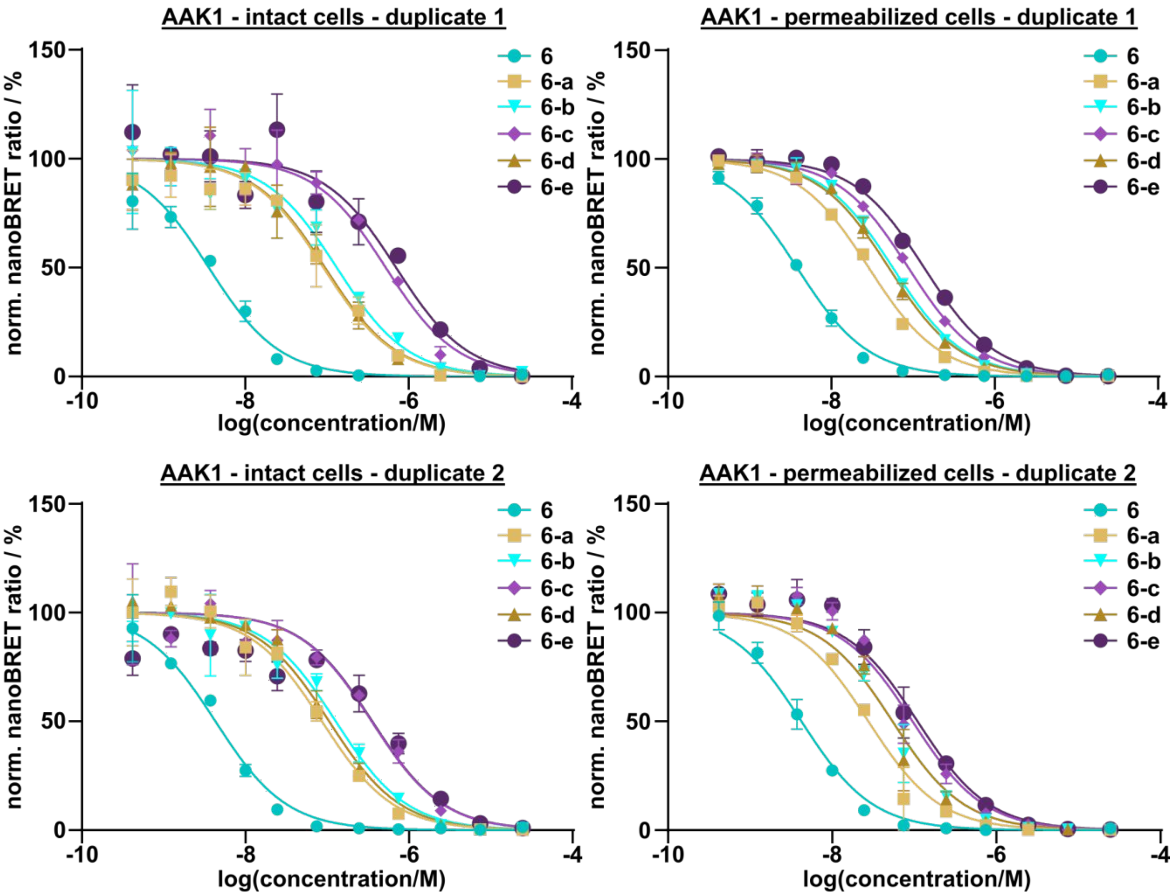

Figure S6: AAK1 engagement data of all compounds based on kinase parent inhibitor 6. NanoBRET® dose response curves of kinase parent inhibitor **6** and resulting promiscuous kinase PROTACs **6-a** – **6-e** measured in duplicates (top row: duplicate 1; bottom row: duplicate 2) against AAK1 in intact (left) and permeabilized (right) cells.

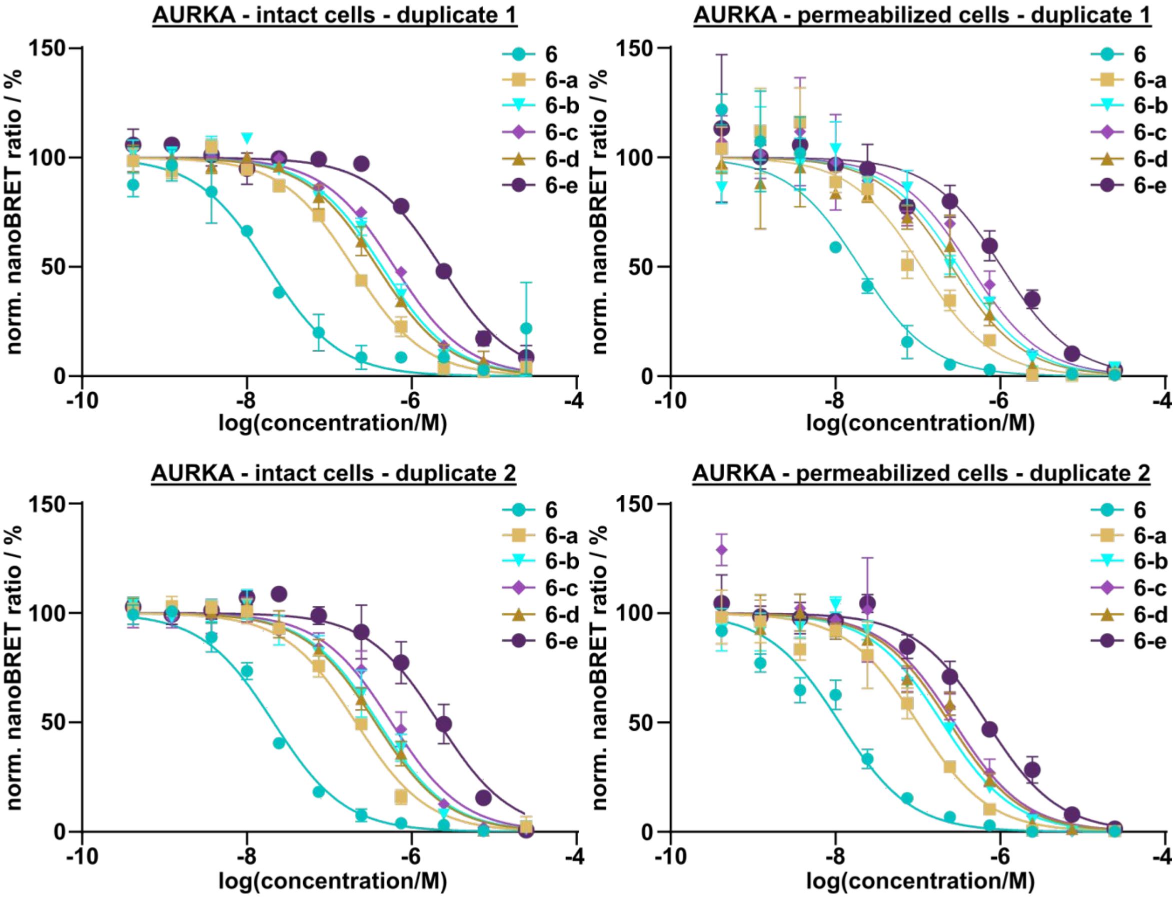

Figure S7: AURKA engagement data of all compounds based on kinase parent inhibitor 6. NanoBRET® dose response curves of kinase parent inhibitor **6** and resulting promiscuous kinase PROTACs **6-a** – **6-e** measured in duplicates (top row: duplicate 1; bottom row: duplicate 2) against AURKA in intact (left) and permeabilized (right) cells.

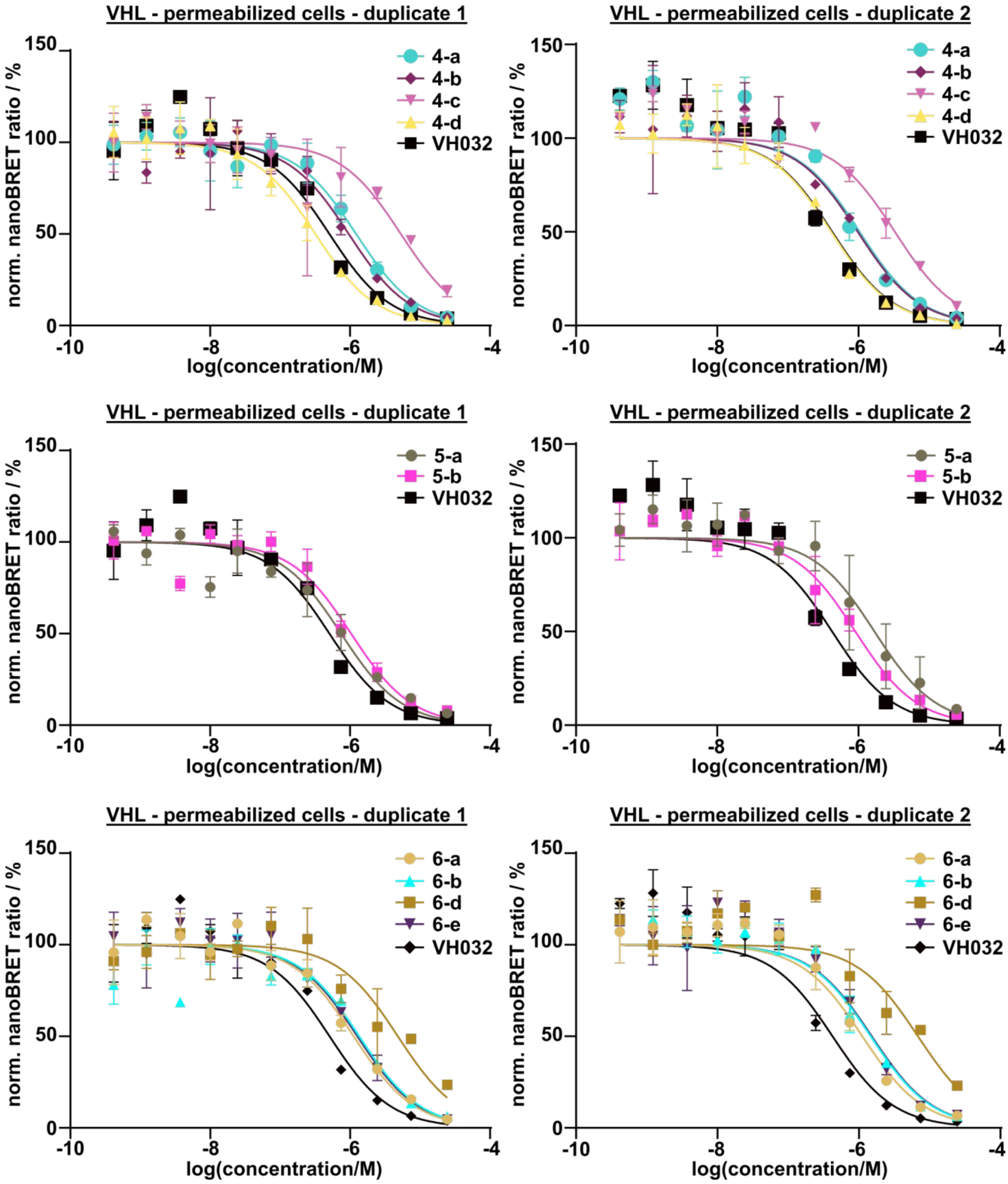

Figure S8: VHL engagement data of all promiscuous kinase PROTACs. NanoBRET® dose response curves of all promiscuous kinase PROTACs measured in duplicates (left column: duplicates 1; right column: duplicates 2) against VHL in permeabilized cells using commercially available VH032 as a positive control. PROTACs are shown according to the used kinase parent inhibitor (top row: PROTACs based on kinase parent inhibitor **4**; mid row: PROTACs based on kinase parent inhibitor **5**; bottom row: PROTACs based on kinase parent inhibitor **6**).

Table S1: Summarized VHL engagement data of all promiscuous kinase PROTACs. IC_{50} values are given as the means of duplicate measurements (shown in Figure S8) with errors indicating standard deviation. Commercially available VH032 was used as a positive control. PROTACs based on the same kinase parent inhibitor are clustered in colored boxes (inhibitor **4** represented in dark green, inhibitor **5** represented in light blue and inhibitor **6** represented in light green). [a]IC_{50} value is outside of the assay window.

cmpd. ID	VHL permeabilized (IC_{50}±sd)/μM
**4-a**	1.133±0.132
**4-b**	0.970±0.013
**4-c**	4.317±1.462
**4-d**	0.366±0.064
**5-a**	1.217±0.665
**5-b**	0.972±0.094
**6-a**	1.123±0.005
**6-b**	1.410±0.083
**6-c**	-[a]
**6-d**	5.831±1.428
**6-e**	1.396±0.174
**VH032**	0.466±0.060

III. Proteomics Data

III.a Kinase Parent Inhibitors 4 – 6

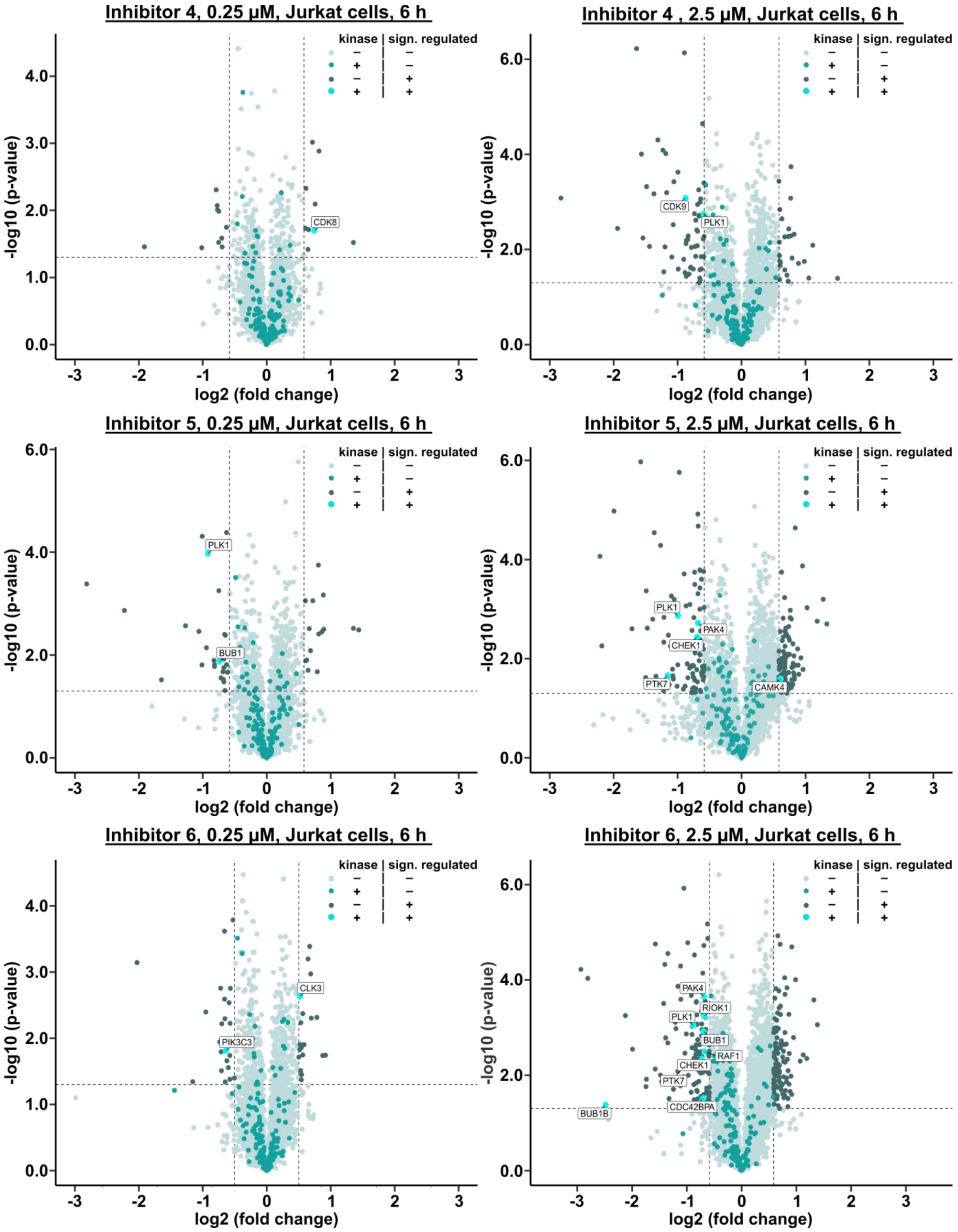

Figure S9: Kinase parent inhibitors 4 – 6 have a marginal effect on kinase expression levels. Jurkat cells were treated with 0.25 µM (left column) and 2.5 µM (right column) of inhibitors **4** – **6** for six hours, respectively. The cells were then lysed, and the resulting lysates were analyzed via quantitative proteomics. Non-kinase proteins that exhibited no significant changes in expression levels are represented by light-blue dots; kinases that displayed no significant expression level alterations are represented by green dots; non-kinase proteins that exhibited significant

changes in expression level are represented by dark grey dots; and kinases that were significantly up- or downregulated are represented by enlarged cyan dots and labelled accordingly.

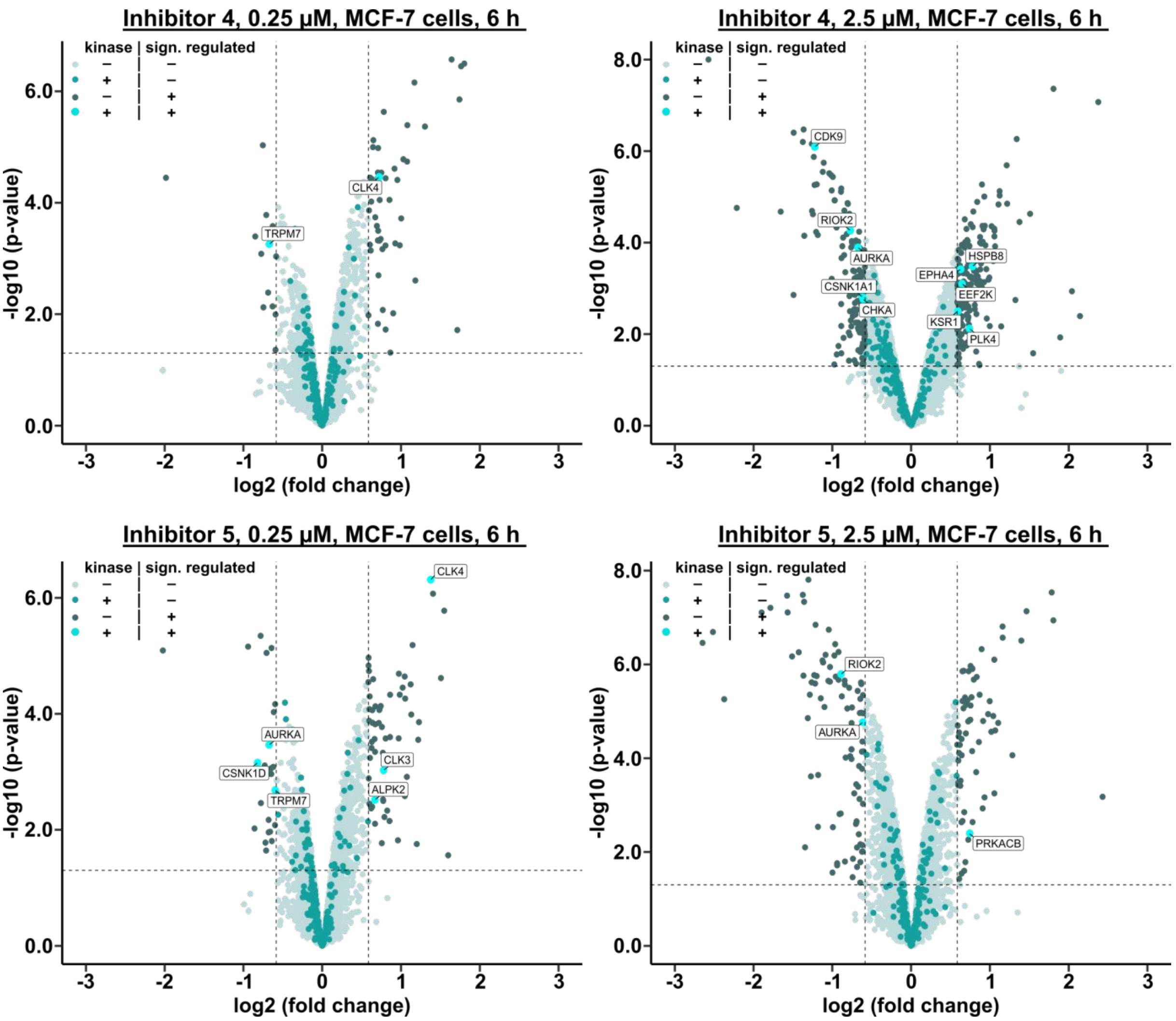

Figure S10: Dysregulation of kinases is cell line specific at higher concentrations. MCF-7 cells were treated with 0.25 µM (left column) and 2.5 µM (right column) of inhibitors **4** and **5** for six hours, respectively. The cells were then lysed, and the resulting lysates were analyzed via quantitative proteomics. Non-kinase proteins that exhibited no significant changes in expression levels are represented by light-blue dots; kinases that displayed no significant expression level alterations are represented by green dots; non-kinase proteins that exhibited significant changes in expression level are represented by dark grey dots; and kinases that were significantly up- or downregulated are represented by enlarged cyan dots and labelled accordingly.

III.b Promiscuous Kinase PROTACs

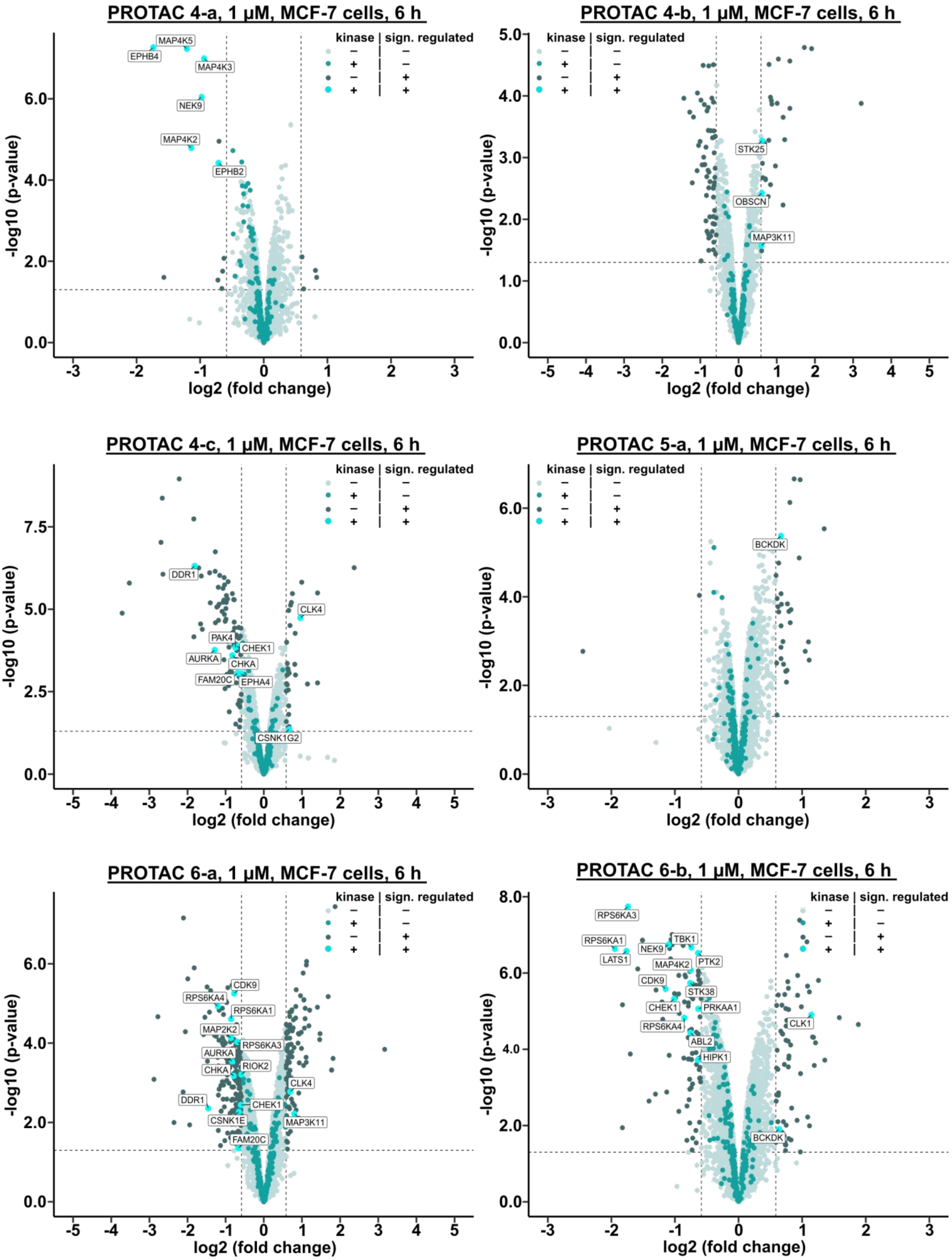

Figure S11: Promiscuous kinase PROTACs show vastly different degradation profiles. MCF-7 cells were treated with 1 µM of PROTAC for six hours. The cells were then lysed, and the resulting lysates were analyzed via quantitative proteomics. Non-kinase proteins that exhibited no significant changes in expression levels are represented by light-blue dots; kinases that displayed no significant expression level alterations are represented by green dots; non-kinase proteins that exhibited significant changes in expression level are represented by dark grey

dots; and kinases that were significantly up- or downregulated are represented by enlarged cyan dots and labelled accordingly.

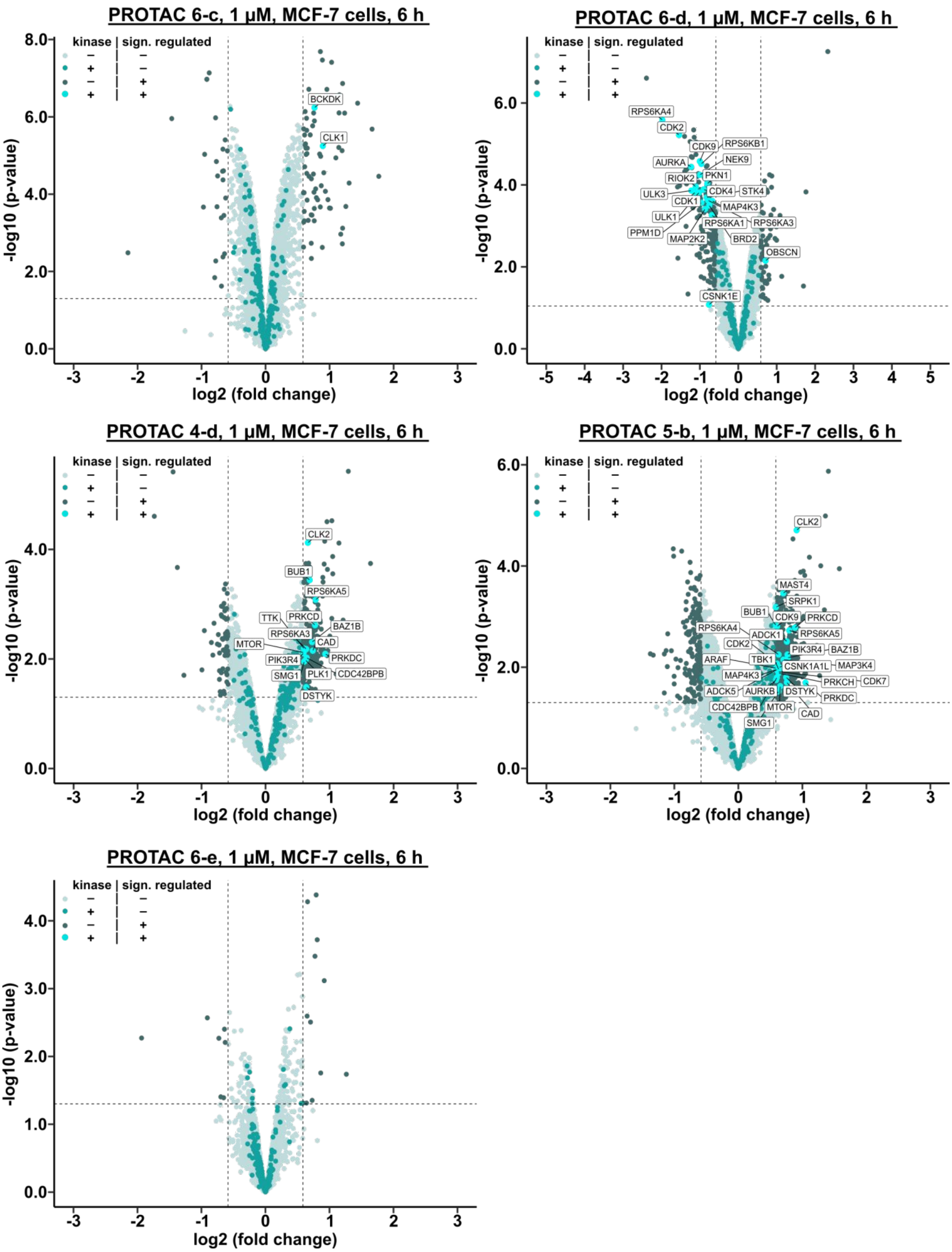

Figure S12: Promiscuous kinase PROTACs show vastly different degradation profiles. MCF-7 cells were treated with 1 µM of PROTAC for six hours. The cells were then lysed, and the resulting lysates were analyzed via quantitative proteomics. Non-kinase proteins that exhibited no significant changes in expression levels are represented by light-blue dots; kinases that displayed no significant expression level alterations are represented by

green dots; non-kinase proteins that exhibited significant changes in expression level are represented by dark grey dots; and kinases that were significantly up- or downregulated are represented by enlarged cyan dots and labelled accordingly.

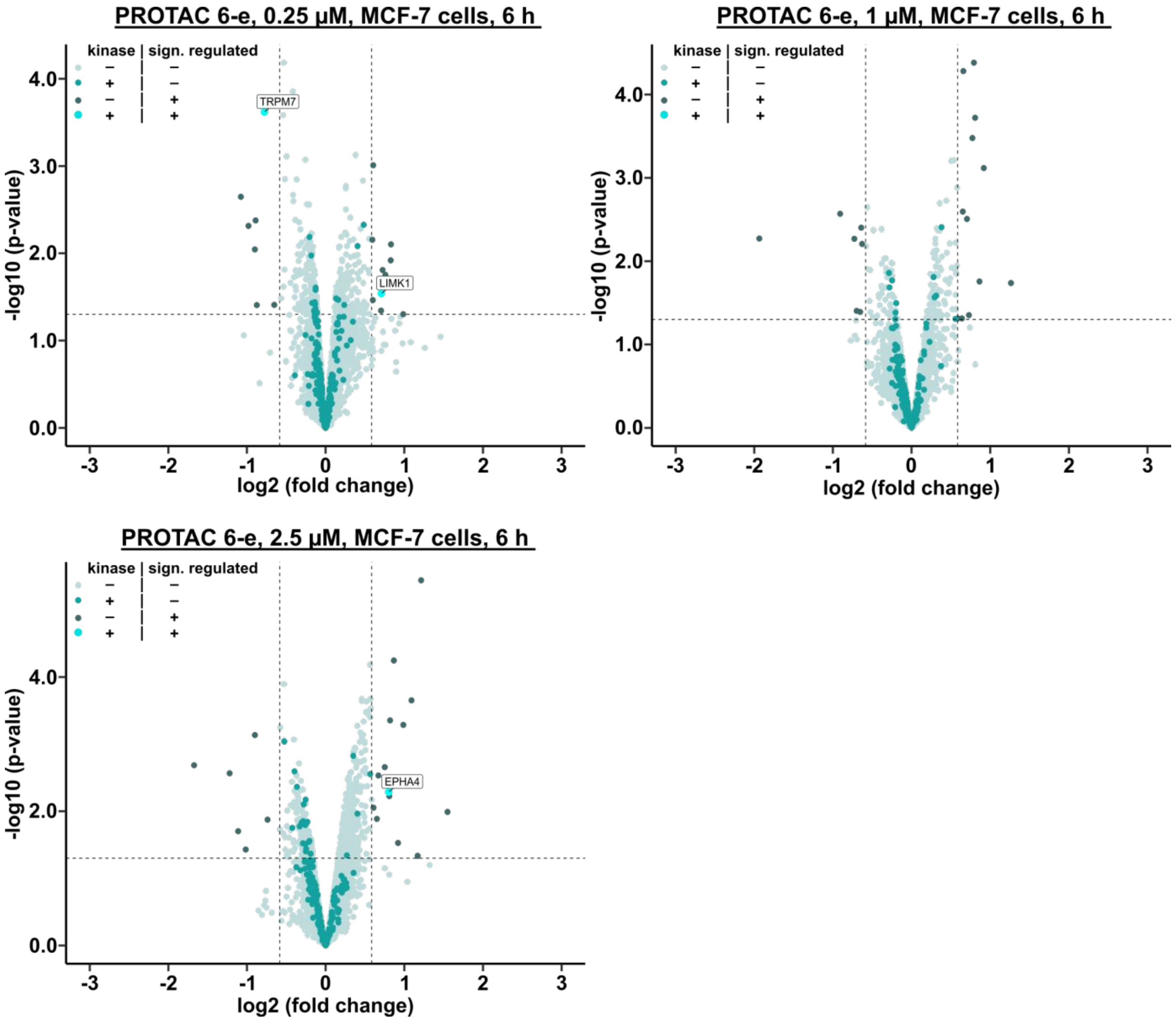

Figure S13: The phenolic VHL exit vector may be unable to form functional ternary complexes in this promiscuous set-up. MCF-7 cells were treated with 0.25 µM, 1 µM and 2.5 µM of PROTAC **6-e** for six hours, respectively. The cells were then lysed, and the resulting lysates were analyzed via quantitative proteomics. Non-kinase proteins that exhibited no significant changes in expression levels are represented by light-blue dots; kinases that displayed no significant expression level alterations are represented by green dots; non-kinase proteins that exhibited significant changes in expression level are represented by dark grey dots; and kinases that were significantly up- or downregulated are represented by enlarged cyan dots and labelled accordingly.

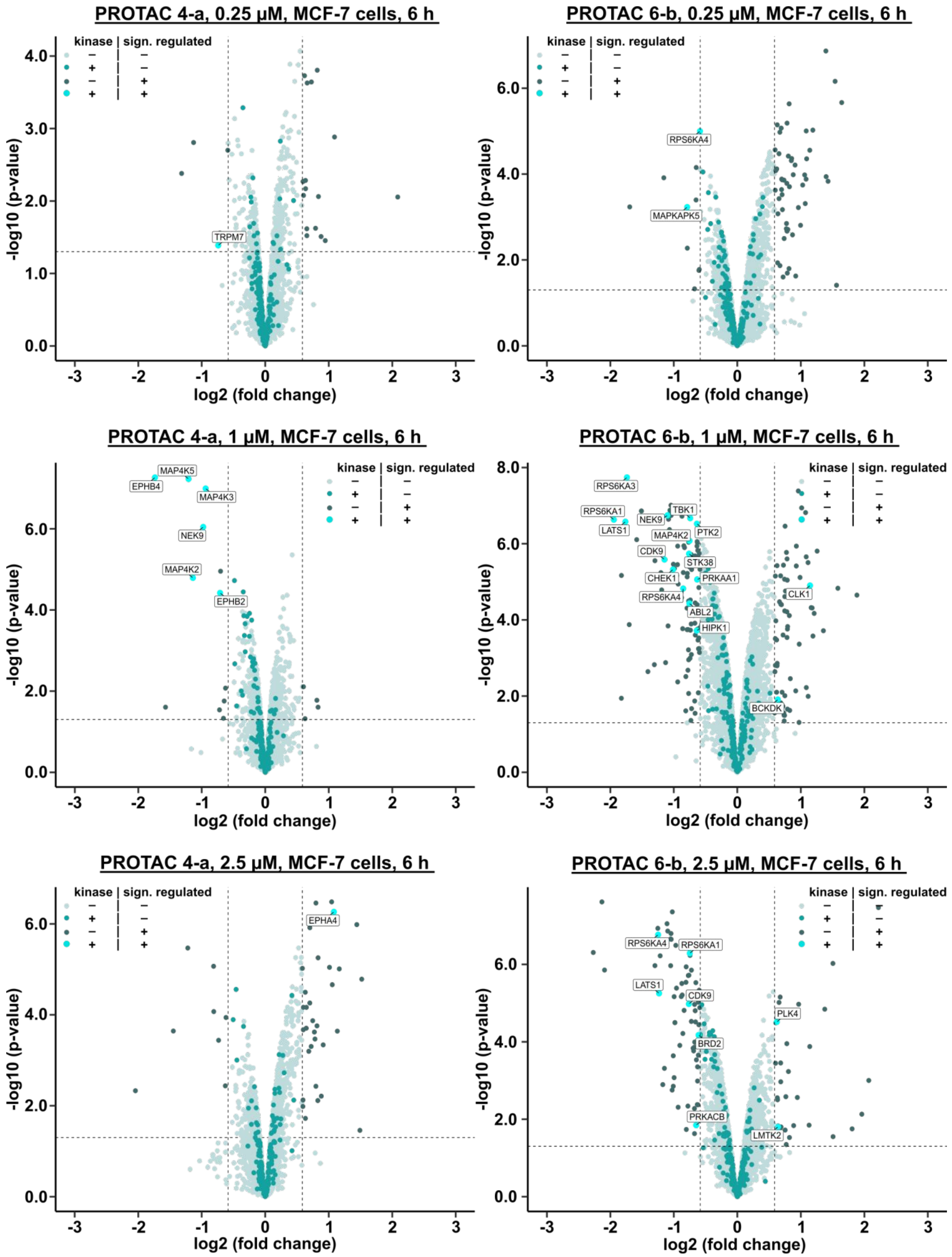

Figure S14: Promiscuous kinase PROTACs 4-a and 6-b exhibit a narrow activity window. MCF-7 cells were treated with 0.25 µM, 1 µM and 2.5 µM of PROTACs **4-a** and **6-b** for six hours, respectively. The cells were then lysed, and the resulting lysates were analyzed via quantitative proteomics. Non-kinase proteins that exhibited no significant changes in expression levels are represented by light-blue dots; kinases that displayed no significant expression level alterations are represented by green dots; non-kinase proteins that exhibited significant changes in expression level are represented by dark grey dots; and kinases that were significantly up- or downregulated are represented by enlarged cyan dots and labelled accordingly.

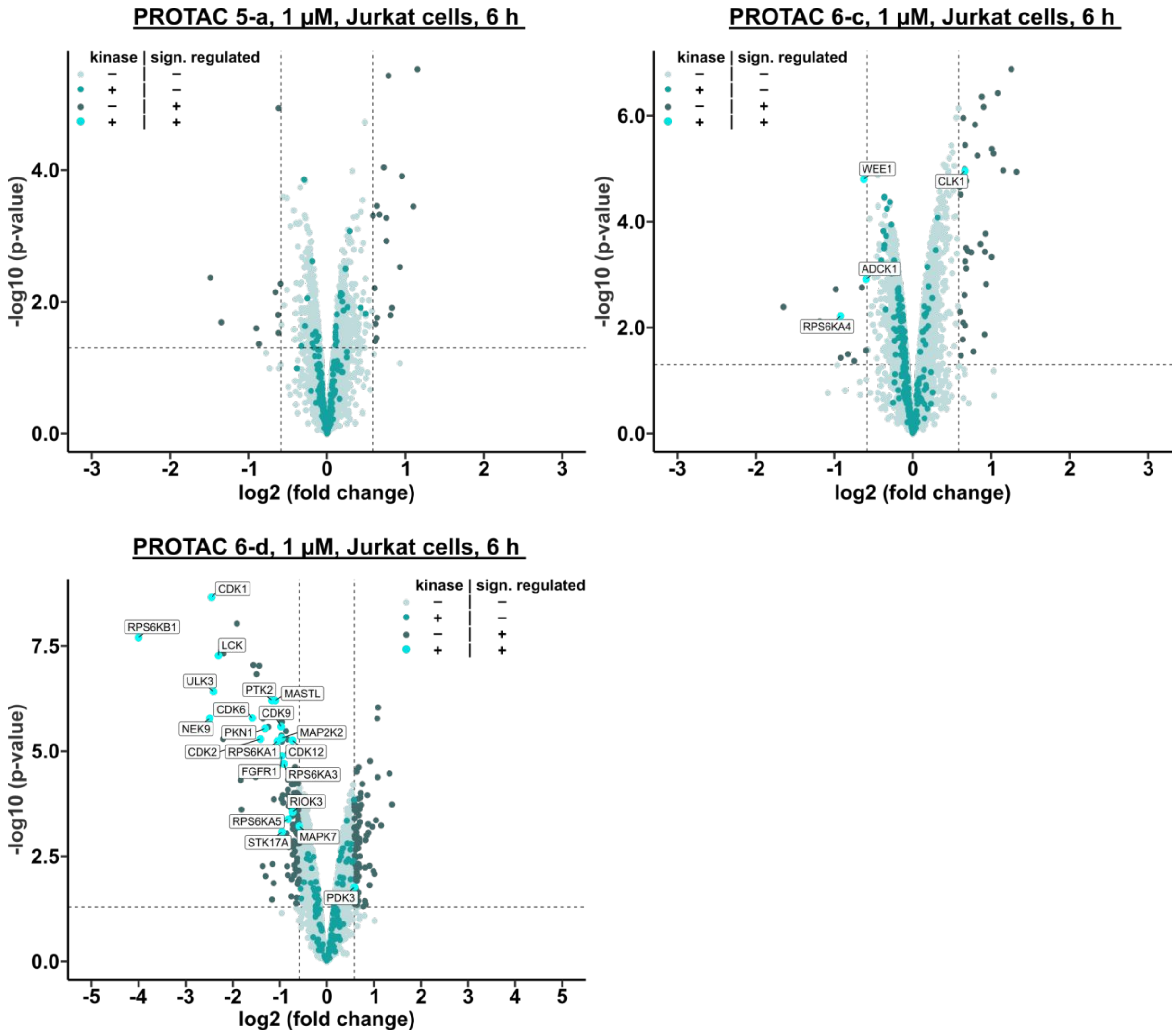

Figure S15: Cell line selection impacts PROTAC degradation profiles and efficacies. Jurkat cells were treated with 1 µM of PROTAC for six hours. The cells were then lysed, and the resulting lysates were analyzed via quantitative proteomics. Non-kinase proteins that exhibited no significant changes in expression levels are represented by light-blue dots; kinases that displayed no significant expression level alterations are represented by green dots; non-kinase proteins that exhibited significant changes in expression level are represented by dark grey dots; and kinases that were significantly up- or downregulated are represented by enlarged cyan dots and labelled accordingly.

Table S2: Cell line restricted protein expression levels do not correlate with cell line exclusive kinase downregulation. Lists of kinases that were exclusively downregulated in either MCF-7 or Jurkat cells after six hours treatment with 1 µM of the respective PROTAC (on the left: PROTAC **4-a**; on the right: PROTAC **6-b**). The cell line exclusive downregulations of kinases are indicated by yellow boxes. Correlations between higher cell line restricted expression levels and cell line exclusive downregulation are highlighted with a star symbol. The respective kinase expression levels were extracted from: https://www.proteinatlas.org.[18] [a]The kinase was not detected in the according proteomic experiment.

PROTAC 4-a	expression levels / nTPM	
**kinase**	**MCF-7 cells**	**Jurkat cells**
**AAK1**	5.6	**9.1 ★**
**ACVR1**	9.2[a]	5.3
**AURKA**	179.0	82.0
**EPHB4**	**65.7 ★**	2.2[a]
**ITK**	0.0[a]	**50.6 ★**
**MAP4K2**	7.5	24.1
**MAP4K3**	**17.1 ★**	4.2[a]
**STK17B**	6.1[a]	**20.3 ★**

PROTAC 6-b	expression levels / nTPM	
**kinase**	**MCF-7 cells**	**Jurkat cells**
**AURKA**	179.0	82.0
**AURKB**	102.2[a]	**162.1 ★**
**CDK17**	10.7	**19.7 ★**
**CDK18**	14.2	**20.7 ★**
**CHKA**	18.6	11.4
**CSNK1D**	69.5	42.7
**HIPK1**	**140.0 ★**	56.3
**IRAK4**	4.2	**8.5 ★**
**ITK**	0.0[a]	**50.6 ★**
**MAPKAPK5**	39.3[a]	**40.1 ★**
**MELK**	39.0[a]	**60.9 ★**
**RPS6KB1**	398.9	22.3
**SGK3**	13.2[a]	6.3
**STK17B**	6.1[a]	**20.3 ★**
**STK35**	10.1[a]	**15.6 ★**
**WEE1**	12.5[a]	**34.3 ★**

exclusively downregulated in stated cell

★ = cell line restricted downregulation correlates with higher expression level in given cell

Table S3: Target engagement by kinase parent inhibitors does not correlate with PROTAC induced downregulation. List of kinases that were downregulated in MCF-7 and Jurkat cells after six hours treatment with 1 µM of the respective PROTAC (on the left: PROTAC **4-a**; on the right: PROTAC **6-b**). The kinases that were found to be downregulated by the respective PROTAC are indicated with a green check mark for the corresponding cell line. The kinases that were not downregulated by the respective PROTAC are indicated with a red cross for the corresponding cell line. Downregulated kinases to which the corresponding kinase parent inhibitors had shown potent binding below 1 µM in kinobead assays are highlighted with a black cross symbol. [a]Kinase was not detected in the kinobead assay.

PROTAC 4-a	induced downregulation		
kinase	MCF-7 cells	Jurkat cells	
AAK1	✗	✓	✦
ACVR1	✗	✓	✦
AURKA	✗	✓	✦
EPHB2	✓	✓	✦
EPHB4[a]	✓	✗	
ITK	✗	✓	✦
MAP4K2	✓	✗	
MAP4K3	✓	✗	
MAP4K5	✓	✓	✦
NEK9	✓	✓	
STK17B	✗	✓	✦

✓ = Kinase was downregulated

✗ = Kinase was not downregulated

✦ = Kinase was hit < 1 µM by kinase parent inhibitor in kinobead experiments

PROTAC 6-b	induced downregulation		
kinase	MCF-7 cells	Jurkat cells	
ABL2	✓	✓	✦
AURKA	✗	✓	✦
AURKB	✗	✓	✦
CDK17	✗	✓	✦
CDK18	✗	✓	✦
CDK9	✓	✓	
CHEK1	✓	✓	✦
CHKA[a]	✗	✓	
CSNK1D	✗	✓	
HIPK1[a]	✓	✗	
IRAK4	✗	✓	✦
ITK	✗	✓	✦
LATS1	✓	✓	✦
MAP4K2	✓	✓	
MAPKAPK5[a]	✗	✓	
MELK	✗	✓	✦
NEK9	✓	✓	✦
PRKAA1	✓	✓	✦
PTK2	✓	✓	✦
RPS6KA1	✓	✓	✦
RPS6KA3	✓	✓	✦
RPS6KA4	✓	✓	✦
RPS6KB1	✗	✓	
SGK3[a]	✗	✓	
STK17B	✗	✓	
STK35[a]	✗	✓	
STK38[a]	✓	✓	
TBK1	✓	✓	✦
WEE1	✗	✓	✦

IV. HiBiT® Data

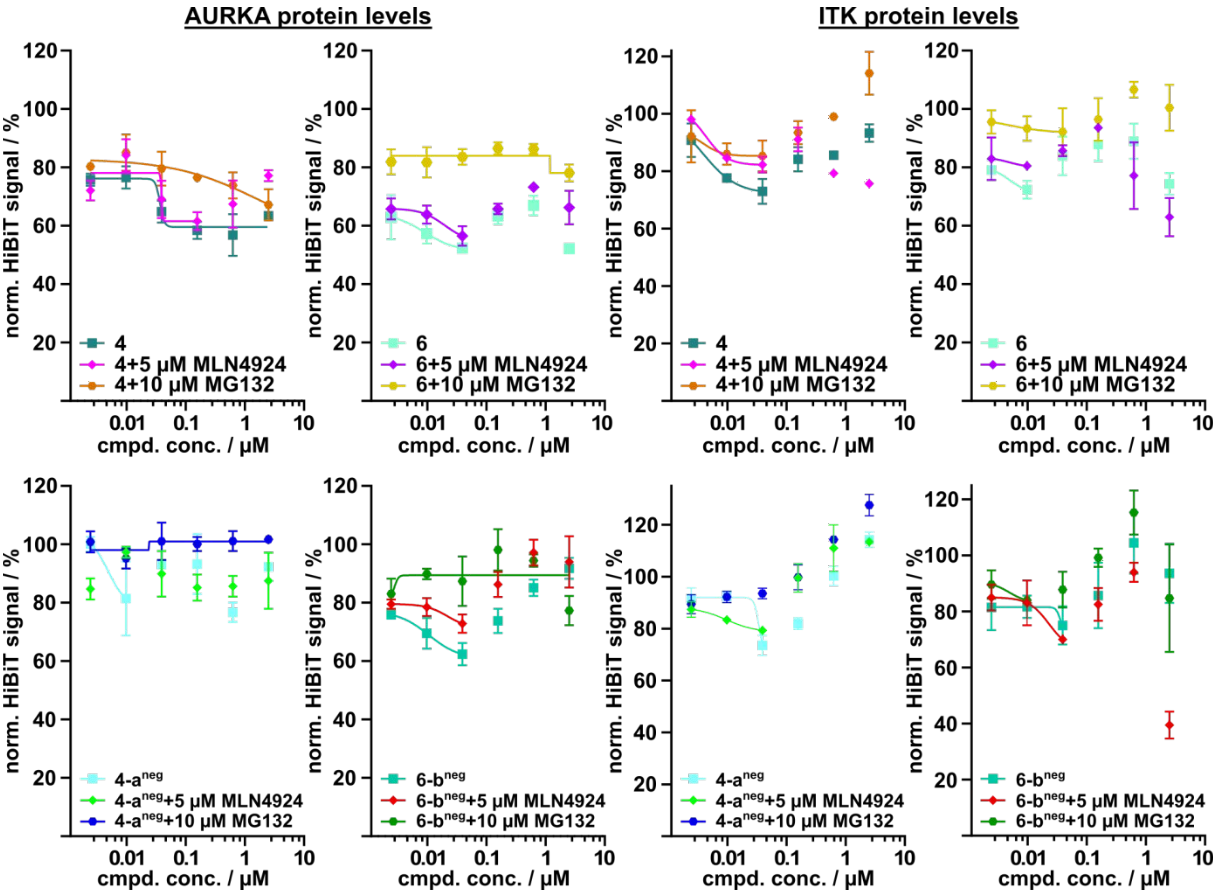

Figure S16: Heightened sensitivity towards early-stage cytotoxic effects induced by promiscuous compounds apparently induces physiological degradation of tagged AURKA in tested HiBiT cell line. AURKA (on the left) and ITK (on the right) protein levels based on luciferase measurements. MV4-11 cells, expressing HiBiT tagged AURKA protein, and Jurkat cells, expressing HiBiT tagged ITK, were co-treated with different concentrations of the specified compounds in presence of either the neddylation inhibitor MLN4924 or the proteasome inhibitor MG132 for 6 hours. Following cell lysis, the resulting lysates were complemented with the largeBiT fragment, and luciferase activity was measured.

V. Assay Data of Negative Controls 4-a^{neg} and 6-b^{neg}

V.a Target Engagement Data

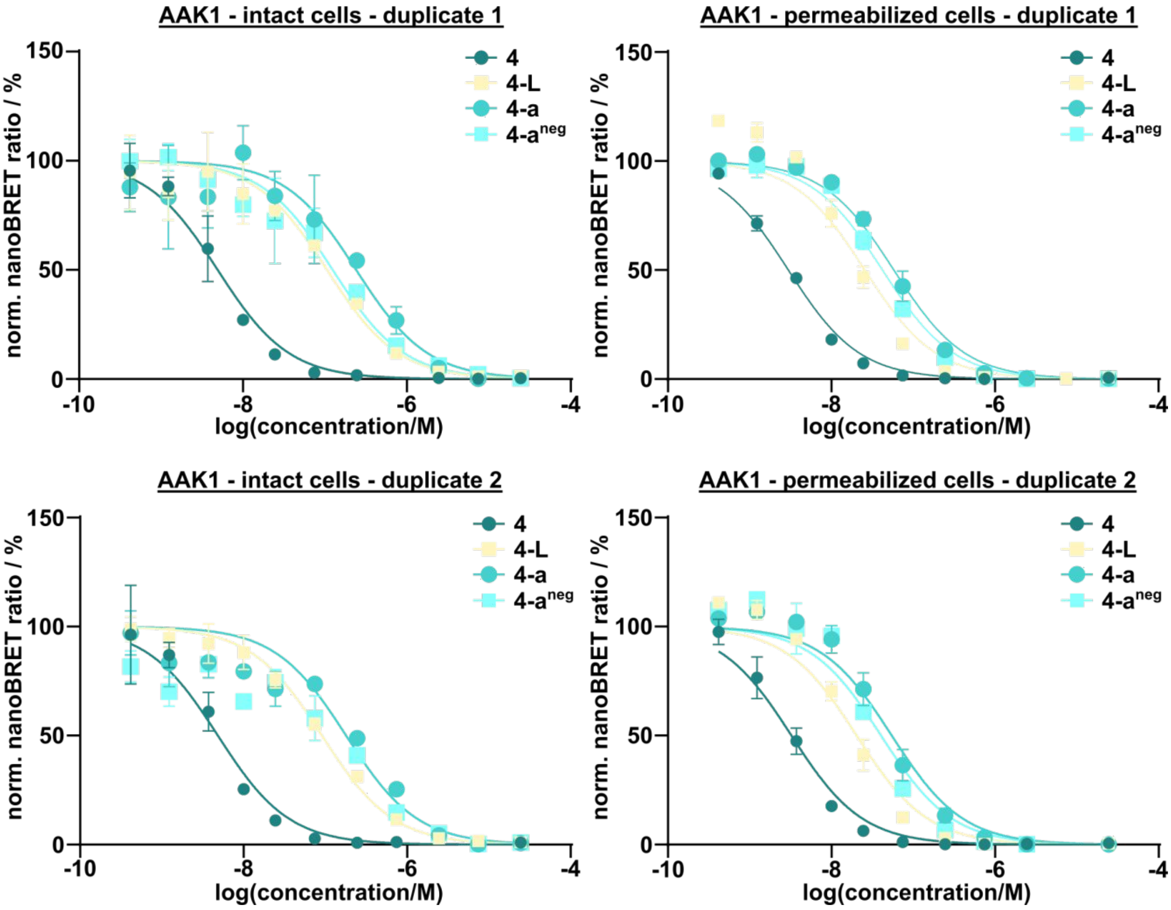

Figure S17: AAK1 engagement data of compounds based on kinase parent inhibitor 4. NanoBRET® dose response curves of kinase parent inhibitor **4**, respective linker conjugate **4-L**, resulting promiscuous kinase PROTAC **4-a** and corresponding negative control **4-a^{neg}** measured in duplicates (top row: duplicate 1; bottom row: duplicate 2) against AAK1 in intact (left) and permeabilized (right) cells.

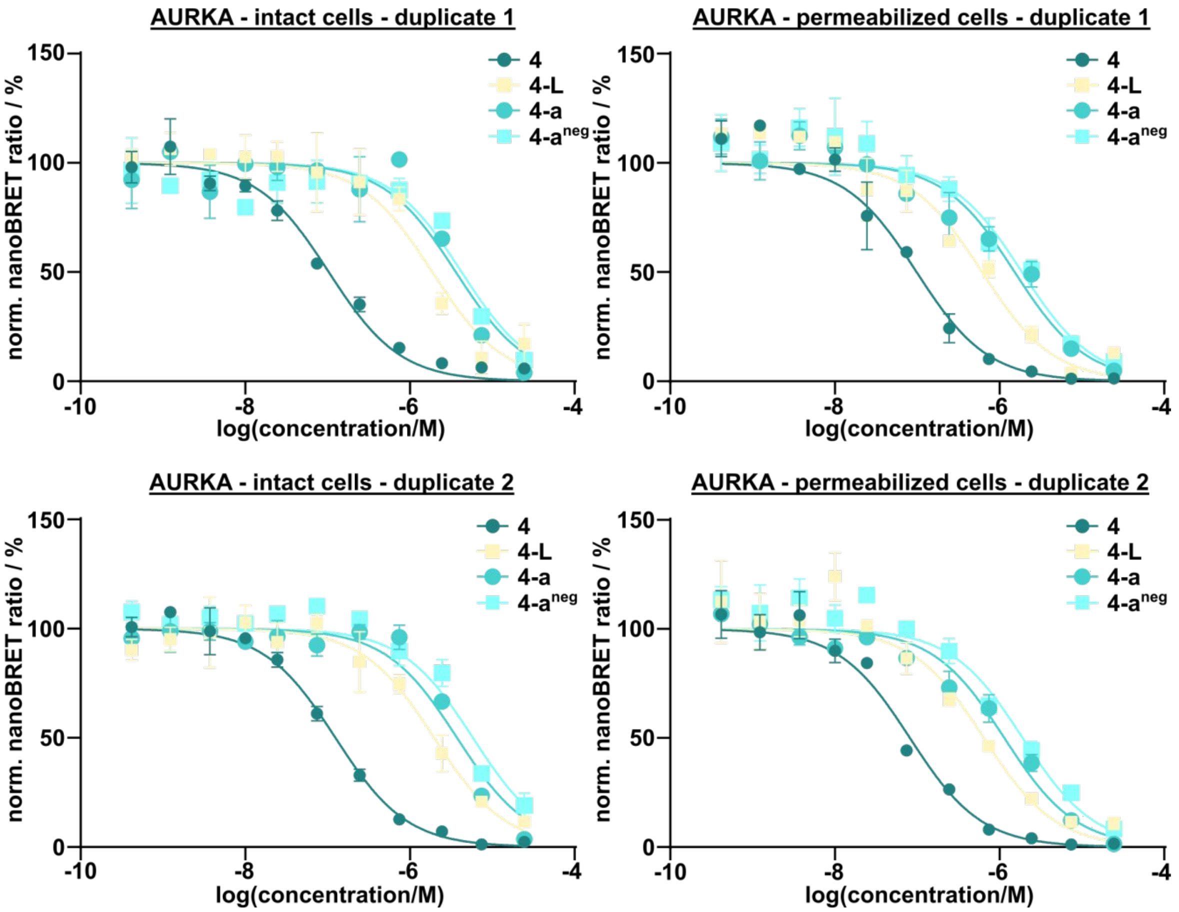

Figure S18: AURKA engagement data of compounds based on kinase parent inhibitor 4. NanoBRET® dose response curves of kinase parent inhibitor **4**, respective linker conjugate **4-L**, resulting promiscuous kinase PROTAC **4-a** and corresponding negative control **4-a^{neg}** measured in duplicates (top row: duplicate 1; bottom row: duplicate 2) against AURKA in intact (left) and permeabilized (right) cells.

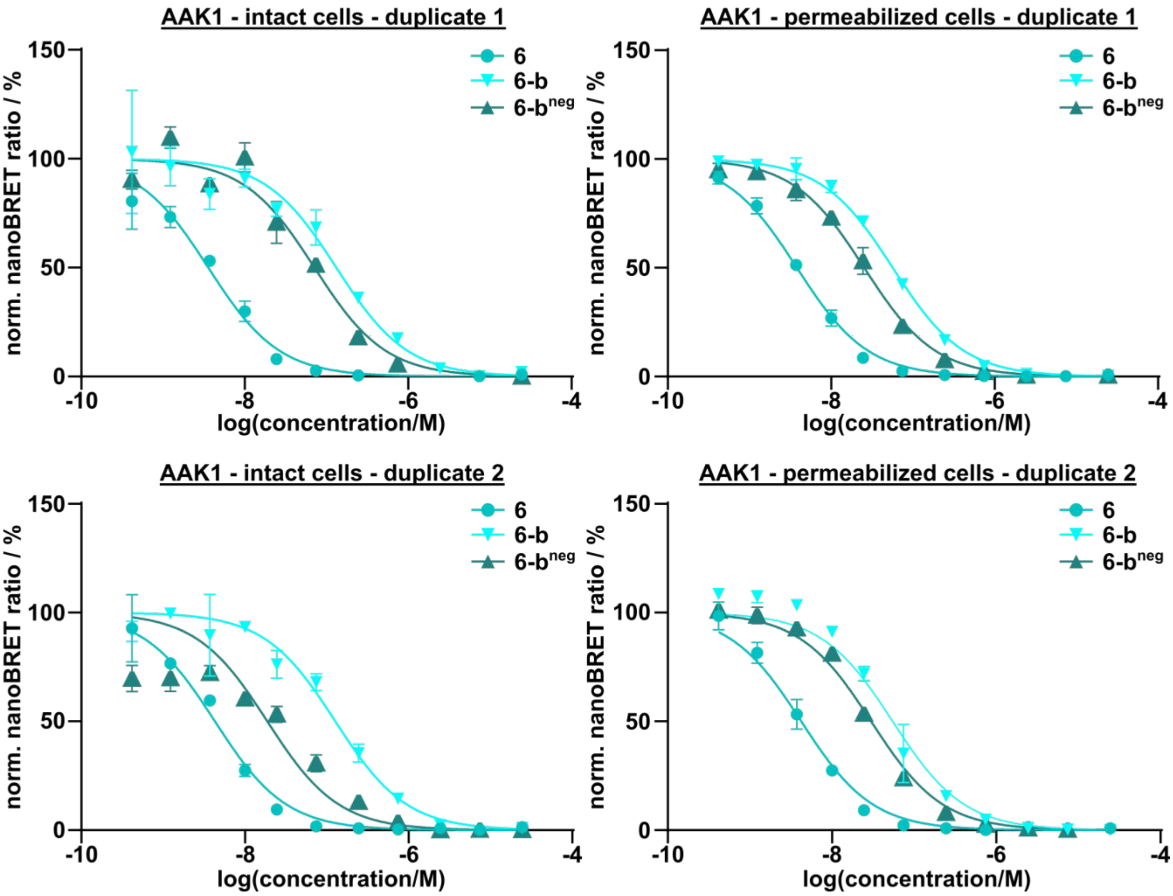

Figure S19: AAK1 engagement data of compounds based on kinase parent inhibitor 6. NanoBRET® dose response curves of kinase parent inhibitor **6**, resulting promiscuous kinase PROTAC **6-a** and corresponding negative control **6-b^{neg}** measured in duplicates (top row: duplicate 1; bottom row: duplicate 2) against AAK1 in intact (left) and permeabilized (right) cells.

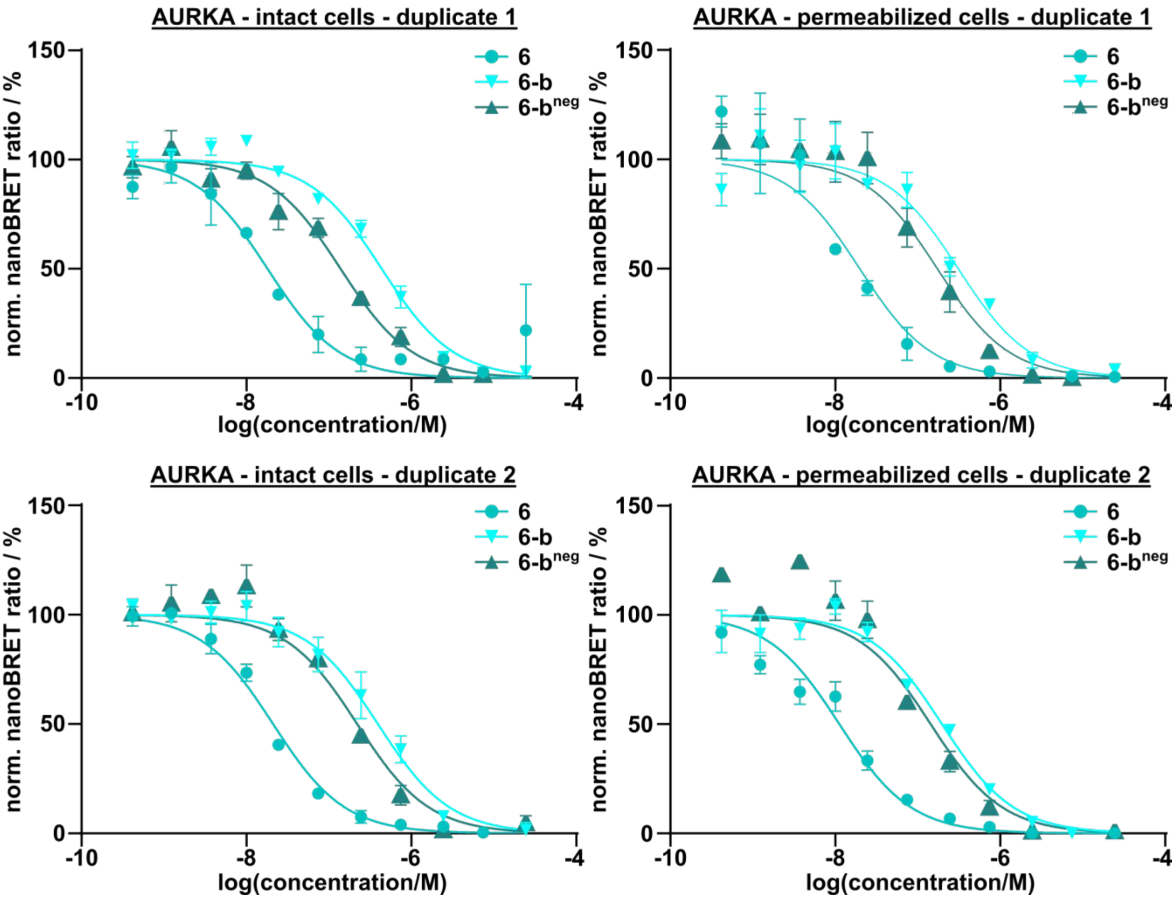

Figure S20: AURKA engagement data of compounds based on kinase parent inhibitor 6. NanoBRET® dose response curves of kinase parent inhibitor **6**, resulting promiscuous kinase PROTAC **6-a** and corresponding negative control **6-b^{neg}** measured in duplicates (top row: duplicate 1; bottom row: duplicate 2) against AURKA in intact (left) and permeabilized (right) cells.

Table S4: Summarized target engagement data of negative controls 4-a^{neg} and 6-b^{neg}. IC_{50} values are given as the means of duplicate measurements with errors indicating standard deviation. PROTACs based on the same kinase parent inhibitor are clustered in colored boxes (inhibitor **4** represented in dark green and inhibitor **6** represented in light green).

	AAK1 intact	permeabilized		AURKA intact	permeabilized	
cmpd. ID	(IC_{50}±sd)/μM	(IC_{50}±sd)/μM	IC_{50} ratio	(IC_{50}±sd)/μM	(IC_{50}±sd)/μM	IC_{50} ratio
4	0.005±0.000	0.003±0.000	0.6	0.114±0.015	0.090±20.01	0.8
4-L	0.099±0.011	0.023±0.004	0.2	1.923±0.070	0.630±0.021	0.3
4-a	0.210±0.044	0.057±0.004	0.3	3.680±0.085	1.338±0.271	0.4
4-a^{neg}	0.108±0.042	0.040±0.001	0.4	5.043±1.115	1.893±0.025	0.4
6	0.004±0.001	0.004±0.000	1.0	0.020±0.002	0.016±0.006	0.8
6-b	0.130±0.003	0.055±0.002	0.4	0.416±0.031	0.254±0.080	0.6
6-b^{neg}	0.045±0.037	0.027±0.003	0.6	0.180±0.058	0.158±0.021	0.9

Table S5: Summarized VHL engagement data of negative controls 4-a[neg] and 6-b[neg]. IC_{50} values are given as the means of duplicate measurements with errors indicating standard deviation. PROTACs based on the same kinase parent inhibitor are clustered in colored boxes (inhibitor **4** represented in dark green and inhibitor **6** represented in light green). [a]IC_{50} value was outside of the assay window.

cmpd. ID	VHL permeabilized (IC_{50}±sd)/µM
**4-a**	1.133±0.132
**4-a[neg]**	-[a]
**6-b**	1.410±0.083
**6-b[neg]**	-[a]
**VH032**	0.466±0.060

V.b Cytotoxicity Data

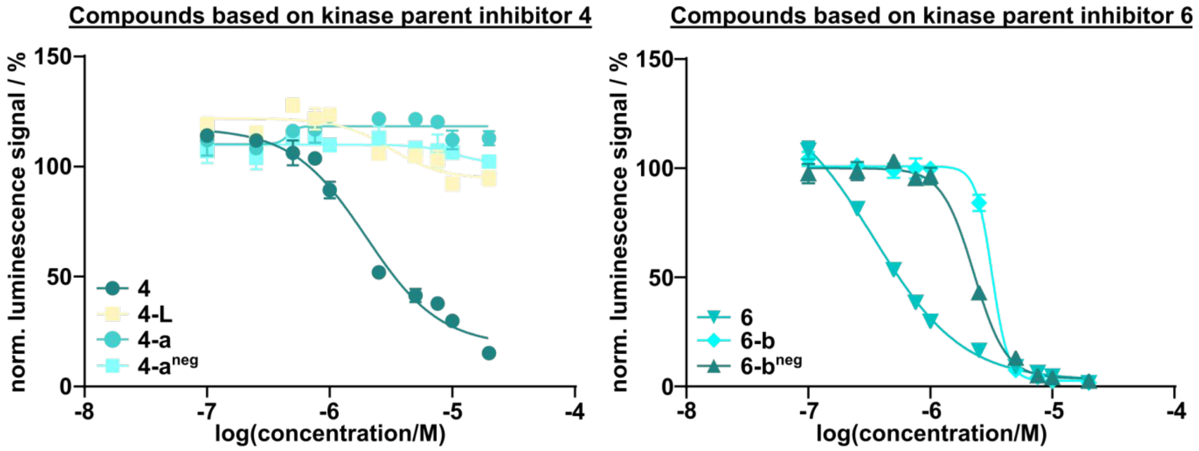

cmpd. ID	IC_{50}±sd/µM
**4**	3.9±1.3
**4-L**	-[a]
**4-a**	-
**4-a[neg]**	-
**6**	0.7±0.2
**6-b**	3.1±0.8
**6-b[neg]**	3.6±1.6

Figure S21: Cytotoxicity Assessment of Negative Controls 4-a[neg] and 6-b[neg]. Top row: CellTiterGLO® dose response curves of Jurkat cells treated with kinase parent inhibitors **4** and **6**, linker conjugates **4-L**, PROTACs **4-a** and **6-b**, and the corresponding negative controls **4-a[neg]** and **6-b[neg]** after 24 hours incubation time. Compounds are shown according to the used kinase parent inhibitor. Bottom row: Summarized IC_{50} values given as the mean of triplicate measurements in µM. Error bard indicate standard deviation. Table entries are clustered according to the used kinase parent inhibitors (**4** represented in dark green and **6** represented in light green). [a] IC_{50} values were outside of the assay window.

Chemistry

Materials & Methods

All commercial chemicals and solvents were used without further purification. All reactions were performed under inert atmosphere (Ar). Reactions were monitored by thin layer chromatography using silica-coated ALUGRAM® Xtra SIL G UV254 plates from Macherey Nagel. Product purification was performed on a PuriFlash Flash Column Chromatography System from Interchim using prepacked silica columns (PF-30SIHP-JP (30 µm), PF-50SIHP-JP (50 µm), PF-15C18HP (15 µm), PF-30C18HP (30 µm) or PF-15NH2 (15 µm)) or by preparative HPLC which was carried out on an Agilent 1260 Infinity II device using an Eclipse XDB-C18 (Agilent, 21.2 x 250 mm, 7 µm) reversed phase column. A suitable gradient (flow rate 21 mL/min.) was used, with 0.1% TFA in water (A) and 0.1% TFA in acetonitrile (B), as a mobile phase. The synthesized compounds were characterized by ^{1}H-NMR and ^{13}C-NMR spectroscopy. NMR spectra were measured in DMSO-d_6, $CDCl_3$ or DCM-d_2 on a Bruker AV500 or DPX600 spectrometer. Chemical shifts (δ) are reported in parts per million (ppm). Determination of the compound purity and/or mass by LCMS was carried out on an Agilent 1260 Infinity II device (methods A-F) with a 1260 DAD HS detector (G7117C; 310 nm or 320 nm) and a LC/MSD device (G6125B, ESI pos. 100-1000). The compounds were analyzed on a Poroshell 120 EC-C18 (Agilent, 3 x 150 mm, 2.7 µm) reversed phase column using 0.1% formic acid in water (A) and 0.1% formic acid in acetonitrile (B) as a mobile phase or on an Agilent 1260 Infinity II device (method G), with a 1260 MWD detector (G7165A; 320 nm). The compounds were analyzed on an Eclipse XDB-C18 (Agilent, 4.6 x 250 mm, 5 µm) reversed phase column using 0.1% TFA in water (A) and 0.1% TFA in acetonitrile (B) as a mobile phase. The following gradients were used:

Method A: 0 min. 5% B - 2 min. 80% B - 5 min. 95% B - 7 min. 95% B (flow rate of 0.6 mL/min.).

Method B: 0 min. 5% B – 0.3 min. 5% B - 5 min. 95% B – 7 min. 95% B (flow rate of 0.6 mL/min.).

Method C: 0 min. 5% B – 0.6 min. 5% B – 4.2 min. 55% B – 7.4 min. 90% B – 9 min. 100% B – 11 min. 100% B (flow rate of 0.6 mL/min.).

Method D: 0 min. 5% B – 2 min. 5% B - 8 min. 98% B – 10 min. 98% B (flow rate of 0.6 mL/min.).

Method E: 0 min. 5% B – 2.8 min. 75% B – 7.2 min. 100% B – 7 min. 100% B (flow rate of 0.6 mL/min.).

Method F: 0 min. 5% B – 0.4 min. 5% B – 8 min. 100% B – 10 min. 100% B (flow rate of 0.6 mL/min.).

UV-detection was performed at 310 nm or 320 nm and all compounds used for further biological characterizations showed > 95% purity if not stated otherwise.

TLC-ESI spectra were measured directly from TLC using TLC-MS interface 2 from Camag. High resolution (Orbitrap) measurements (method 1) were executed on a MALDI LTQ XL Orbitrap spectrometer from Thermo Scientific or on a Exploris 480 Thermo (method 2; Bremen, Germany) mass spectrometer equipped with a heated electrospray source (HESI) and coupled to a liquid chromatography system Vanquish VF-P10-A binary pump, VF-A10-A auto sampler which was set to 10 °C and which was equipped with a 25 µL injection syringe and a 100 µL sample loop. Instead of a column a 0.18 mm, 600 mm length capillary was installed within the column compartment VH-C10-A. For automated direct infusion 2.0 µL sample was injected using the flow gradient in Table S6 with 90% pure acetonitrile and 10% water with 0.1% formic acid. The flow was switched according to Table S6 from waste to the MS and back to the waste, to prevent source contamination. For monitoring two full scan modes were selected with the following parameters. Polarity: positive; scan range: 100 to 1500 *m*/*z*; resolution: 480,000; AGC target: “Standard”; maximum IT: “Auto”. General settings: sheath gas flow rate: 20; auxiliary gas flow rate 5; sweep gas flow rate: 1; spray voltage: 3.5 kV; capillary temperature: 325 °C; S-lens RF level: 50; auxiliary gas heater temperature: 125 °C. For negative mode, all values were kept instead of the spray voltage which was set to 2.5 kV.

Table S6: HRMS flow and MS acquisition.

time	Flow	MS Acquisition	Waste valve
0	0.1	off	waste
0.5	0.1	on	waste → MS
0.6	0.02	on	MS
4	0.02	on	MS → waste
4.1	1	off	waste
5	1	off	waste
5.1	0.2	off	waste
5.5	0.2	off	waste → MS
5.8	0.2	off	MS → waste
6	0.2	off	waste

Experimental Procedures

I. Kinase Parent Inhibitors and Linker Conjugates

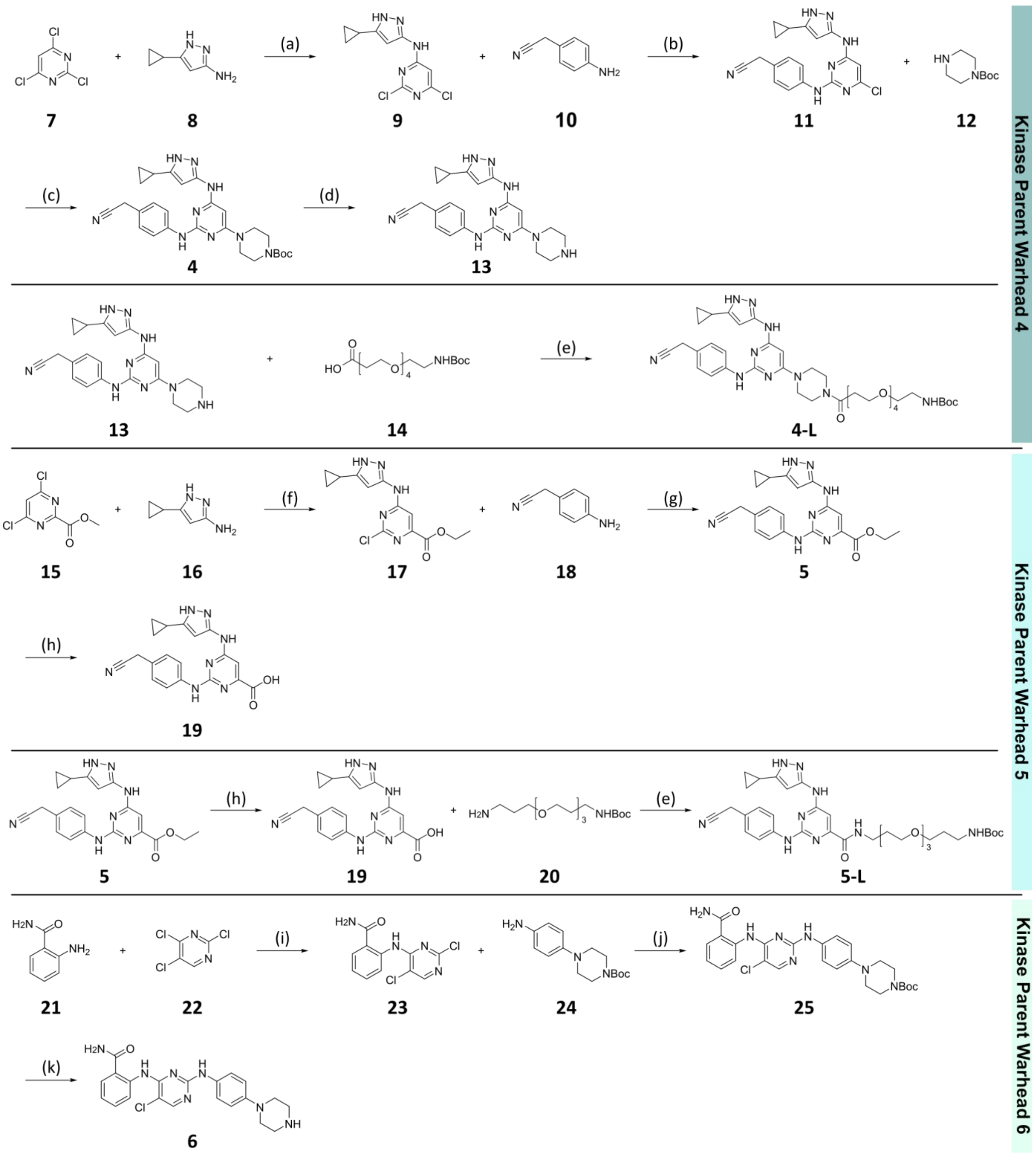

Scheme S1: Synthesis routes for kinase parent inhibitors 4 – 6. Reagents and conditions: (a) DIPEA, *n*-BuOH, 80 °C, 1 h; (b) conc. HCl, *n*-BuOH, 100 °C, overnight; (c) DIPEA, 1,4-dioxane, 125 °C, overnight; (d) 25vol% TFA/DCM, r.t., 30 min - 1 h; (e) HATU, DIPEA, DMF, r.t., overnight; (f) DIPEA, EtOH, 80 °C, overnight; (g) conc. HCl, EtOH, 85 °C, 72 h; (h) LiOH·H_2O, 4:1 MeOH/H_2O, r.t., 1 h; (i) DIPEA, *i*-PrOH, 100 °C, overnight; (j) DMF, 100 °C, overnight; (k) 2 M HCl in 1,4-dioxane, r.t., 6 h.

2,6-dichloro-N-(5-cyclopropyl-1H-pyrazol-3-yl)pyrimidin-4-amine (9)

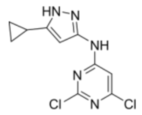

2,4,6-trichloropyrimidine (**7**) (1.88 mL, 16.4 mmol, 1.00 eq.) and 5-cyclopropyl-1H-pyrazol-3-amine (**8**) (2.01 g, 16.4 mmol, 1.00 eq.) were dissolved in 16 mL *n*-butanol. DIPEA (5.70 mL, 32.7 mmol, 2.00 eq.) were added and the resulting reaction mixture was stirred at 80 °C for 1 h. After cooling the reaction mixture to room temperature, all volatiles were removed under reduced pressure. The resulting crude was then dissolved in ethyl acetate and washed with water (thrice) followed by brine. The combined organic layers were dried over $MgSO_4$, filtered and all volatiles were removed under reduced pressure. The crude was then loaded unto celite and purified via flash column chromatography using ethyl acetate/*cyclo*-hexane (30% → 100%) as eluent to give the title compound as an off-white solid (3.73 g, 84%).

^{1}H-NMR (500 MHz, DMSO-*d$_6$*): δ = 12.27 (s, 1H), 10.64 (s, 1H), 7.98 – 6.56 (m, 1H), 6.50 – 5.37 (m, 1H), 1.93 – 1.85 (m, 1H), 0.95 – 0.91 (m, 2H), 0.70 – 0.66 (m, 2H).

^{13}C-NMR (126 MHz, DMSO-*d$_6$*): δ = 161.2, 159.7, 158.3, 147.0, 146.6, 103.6, 102.6, 93.3, 92.2, 7.8, 6.7.

LCMS: R_t = 4.83 min (method E).

MS(ESI) [m/z]: calculated = 270.03 $[M+H]^+$; found = 270.0 $[M+H]^+$.

2-(4-((4-chloro-6-((5-cyclopropyl-1H-pyrazol-3-yl)amino)pyrimidin-2-yl)amino)phenyl)acetonitrile (11)

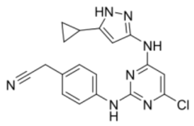

2,6-dichloro-N-(5-cyclopropyl-1H-pyrazol-3-yl)pyrimidin-4-amine (**9**) (3.70 g, 13.7 mmol, 1.00 eq.) and 2-(4-aminophenyl)acetonitrile (**10**) (1.81 g, 13.7 mmol, 1.00 eq.) were dissolved in 20 mL *n*-butanol. Conc. $HCl_{aqu.}$ (37%, 1.26 mL, 15.1 mmol, 1.10 eq.) was added and the resulting mixture was stirred at 100 °C overnight. The cooled reaction mixture was then filtered and the residue was thoroughly washed with 30 mL of cold *n*-butanol to give the title compound as beige solid (2.02 g, 40%).

^{1}H-NMR (500 MHz, DMSO-*d$_6$*): δ = 11.87 – 9.46 (m, 3H), 7.74 (d, ^3J = 8.3 Hz, 1H), 7.48 – 7.40 (m, 1H), 7.31 – 7.26 (m, 1H), 6.50 (s, 1H), 6.01 (s, 1H), 3.97 (s, 2H), 2.04 – 1.98 (m, 1H), 1.07 – 1.02 (m, 2H), 0.86 – 0.79 (m, 2H).

^{13}C-NMR (126 MHz, DMSO-d_6): δ = 159.4, 158.7, 158.4, 139.2, 131.34, 131.3, 129.4, 128.4, 124.4, 123.9, 119.4, 119.0, 96.2, 90.8, 22.0, 21.8, 8.8, 6.9.

LCMS: R_t = 4.86 min (method E).

MS(ESI) [m/z]: calculated = 366.12 $[M+H]^+$; found = 366.1 $[M+H]^+$.

***tert*-butyl 4-(2-((4-(cyanomethyl)phenyl)amino)-6-((5-cyclopropyl-1H-pyrazol-3-yl)amino)pyrimidin-4-yl)piperazine-1-carboxylate (4)**

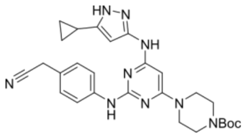

2-(4-((4-chloro-6-((5-cyclopropyl-1H-pyrazol-3-yl)amino)pyrimidin-2-yl)amino)phenyl)acetonitrile (**11**) (2.00 g, 5.47 mmol, 1.00 eq.) and *tert*-butyl piperazine-1-carboxylate (**12**) (5.09 g, 27.3 mmol, 5.00 eq.) were suspended in 5.4 mL dry 1,4-dioxane. DIPEA (1.90 mL, 10.9 mmol, 2.00 eq.) was added and the resulting reaction mixture was stirred at 125 °C overnight. After cooling to room temperature, the reaction mixture was diluted with ethyl acetate and washed with water (thrice) followed by brine. The combined organic layers were dried over $MgSO_4$, filtered and all volatiles were removed under reduced pressure. The obtained crude was then loaded unto celite and purified via flash column chromatography using methanol/dichloromethane (0% → 5%) as eluent to give the title compound as an off-white solid (2.04 g, 72%).

^{1}H-NMR (500 MHz, DMSO-d_6): δ = 12.47 – 11.71 (m, 1H), 9.92 – 8.34 (m, 2H), 7.81 – 7.54 (m, 2H), 7.29 – 7.13 (m, 2H), 6.30 – 5.13 (m, 2H), 3.93 (s, 2H), 3.52 – 3.38 (m, 8H), 1.89 – 1.79 (m, 1H), 1.43 (s, 9H), 0.98 – 0.80 (m, 2H), 0.72 – 0.59 (m, 2H).

^{13}C-NMR (126 MHz, DMSO-d_6): δ = 163.1, 160.8, 158.7, 153.9, 149.1, 145.3, 140.8, 128.1, 122.5, 119.6, 119.0, 92.6, 79.1, 77.3, 43.6, 42.4, 28.1, 21.8, 7.7, 6.8.

LCMS: R_t = 4.22 min (method E); purity = 96%.

MS(ESI) [m/z]: calculated = 516.28 $[M+H]^+$; found = 516.3 $[M+H]^+$.

HRMS: calculated = 516.2830 $[M+H]^+$; found = 516.2826 $[M+H]^+$.

2-(4-((4-((5-cyclopropyl-1H-pyrazol-3-yl)amino)-6-(4l4-piperazin-1-yl)pyrimidin-2-yl)amino)phenyl)acetonitrile (13)

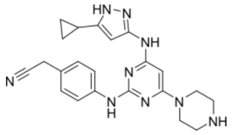

Tert-butyl 4-(2-((4-(cyanomethyl)phenyl)amino)-6-((5-cyclopropyl-1H-pyrazol-3-yl)amino)pyrimidin-4-yl)piperazine-1-carboxylate (**4**) (33 mg, 0.064 mmol, 1.0 eq.) was dissolved in 2 mL of a 25vol% solution trifluoroacetic acid/dichloromethane. The resulting mixture was stirred at room temperature for 1 h. The reaction mixture was then diluted with ethyl acetate, washed with sat. aqueous $NaHCO_3$ solution, followed by water and brine. The combined organic layers were dried over $MgSO_4$, filtered and all volatiles were removed under reduced pressure to give the title compound as the free base (quant.) and it was used without further purification in follow-up reactions.

LCMS: R_t = 3.30 (method E).

ESI [m/z]: calculated = 416.23 $[M+H]^+$; found = 416.3 $[M+H]^+$.

Tert-butyl (15-(4-(2-((4-(cyanomethyl)phenyl)amino)-6-((5-cyclopropyl-1H-pyrazol-3-yl)amino)pyrimidin-4-yl)piperazin-1-yl)-15-oxo-3,6,9,12-tetraoxapentadecyl)carbamate (4-L)

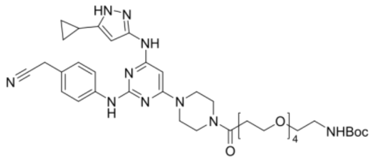

2,2-dimethyl-4-oxo-3,8,11,14,17-pentaoxa-5-azaicosan-20-oic acid (**14**) (35 mg, 0.096 mmol, 1.0 eq.), HATU (51 mg, 0.13 mmol, 1.4 eq.) and DIPEA (23 µL, 0.13 mmol, 1.4 eq.) were dissolved in 1 mL dry DMF and the resulting mixture was stirred for 20 minutes at room temperature. 2-(4-((4-((5-cyclopropyl-1H-pyrazol-3-yl)amino)-6-(4l4-piperazin-1-yl)pyrimidin-2-yl)amino)phenyl)acetonitrile (**13**) (44 mg, 0.11 mmol, 1.1 eq.) was dissolved in 1 mL dry DMF and added to the reaction mixture. The resulting mixture was stirred at room temperature overnight. The mixture was then diluted with ethyl acetate and washed with water followed by brine. The combined organic layers were dried over $MgSO_4$, filtered and all volatiles were removed under reduced pressure. The obtained crude was loaded unto celite and purified via reverse phase flash column chromatography using acetonitrile/water (5% → 100%) as eluent to give the title compound as pale yellow solid (38 mg, 52%).

^{1}H-NMR (500 MHz, DMSO-*d₆*): δ = 12.78 – 11.71 (m, 1H), 9.83 – 8.64 (m, 2H), 7.78 – 7.69 (m, 2H), 7.27 – 7.20 (m, 2H), 6.80 – 6.69 (m, 1H), 6.41 – 5.32 (m, 1H), 3.93 (s, 1H), 3.65 (t, ^3J = 6.3 Hz, 2H), 3.61 – 3.41 (m, 22H), 3.07 – 3.03 (m, 2H), 2.63 (t, ^3J = 6.1 Hz, 2H), 1.88 – 1.79 (m, 1H), 1.37 (s, 9H), 9.93 – 0.85 (m, 2H), 0.69 – 0.63 (m, 2H).

^{13}C-NMR (126 MHz, DMSO-*d₆*): δ = 169.1, 163.1, 160.5, 158.6, 155.6, 151.1, 140.8, 128.9, 128.1, 122.6, 120.7, 119.6, 118.9, 77.6, 77.0, 69.8, 69.72, 69.70, 69.5, 69.2, 66.8, 44.5, 43.9, 43.6, 40.6, 32.9, 28.2, 21.8, 7.7.

LCMS: R_t = 4.93 min (method B); purity = 96%.

MS(ESI) [m/z]: calculated = 763.43 $[M+H]^+$; found = 763.6 $[M+H]^+$.

Ethyl 2-chloro-6-((5-cyclopropyl-1H-pyrazol-3-yl)amino)pyrimidine-4-carboxylate (17)

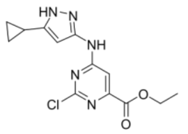

Methyl 2,6-dichloropyrimidine-4-carboxylate (**15**) (2.50 g, 12.1 mmol, 1.00 eq.) was suspended in 20 mL ethanol. 5-cyclopropyl-1H-pyrazol-3-amine (**16**) (1.49 g, 12.0 mmol, 1.00 eq.) was added followed by DIPEA (4.2 mL, 24 mmol, 2.0 eq.) and the resulting reaction mixture was stirred at 80 °C overnight. The cooled reaction mixture was then diluted with ethyl acetate and washed with water followed by brine. The combined organic layers were dried over $MgSO_4$, filtered and all volatiles were removed under reduced pressure. The resulting crude was loaded unto celite and purified via flash column chromatography using MeOH/DCM (0% → 5%) as eluent. The title compound was isolated as an off-white solid (2.60 g, 73%).

^{1}H-NMR (500 MHz, DMSO-*d$_6$*): δ = 12.41 – 12.17 (m, 1H), 10.84 – 10.57 (m, 1H), 8.45 – 7.22 (m, 1H), 6.48 – 5.56 (m, 1H), 4.33 (q, ^3J = 7.1 Hz, 2H), 1.99 – 1.84 (m, 1H), 1.31 (t, ^3J = 7.1 Hz, 3H), 0.96 – 0.91 (m, 2H), 0.71 – 0.67 (m, 2H).

^{13}C-NMR (126 MHz, DMSO-*d$_6$*): δ = 163.1, 162.1, 161.2, 159.9, 156.3, 153.9, 147.2, 146.6, 145.7, 107.0, 104.9, 93.5, 92.4, 61.9, 14.0, 7.8, 6.7.

TLC-MS(ESI) [m/z]: calculated = 330.07 $[M+Na]^+$; found = 330.2 $[M+Na]^+$.

Ethyl 2-((4-(cyanomethyl)phenyl)amino)-6-((5-cyclopropyl-1H-pyrazol-3-yl)amino)pyrimidine-4-carboxylate (5)

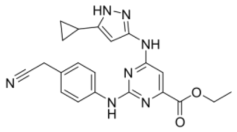

Ethyl 2-chloro-6-((5-cyclopropyl-1H-pyrazol-3-yl)amino)pyrimidine-4-carboxylate (**17**) (2.60 g, 8.48 mmol, 1.00 eq.) and 2-(4-aminophenyl)acetonitrile (**18**) (1.12 g, 8.48 mmol, 1.00 eq.) were suspended in 30 mL ethanol. Conc. $HCl_{aqu,}$ (37%, 1.13 mL, 13.6 mmol, 1.60 eq.) was added dropwise and the resulting reaction mixture was stirred at 85 °C for 72 h. The cooled reaction mixture was diluted with ethyl acetate and then carefully quenched with saturated aqueous $NaHCO_3$ solution. Layers were separated and the organic layer was washed with water followed by brine. The combined organic layers were dried over $MgSO_4$, filtered and all volatiles were removed under reduced pressure. The resulting crude was recrystallized from methanol to give the title compound as yellow powder (2.41 g, 71%).

^{1}H-NMR (500 MHz, DMSO-d_6): δ = 12.54 – 12.01 (m, 1H), 10.63 – 9.41 (m, 2H), 7.92 – 7.70 (m, 2H), 7.24 (d, ^{3}J = 8.5 Hz, 2H), 7.10 – 6.02 (m, 1H), 4.32 (q, ^{3}J = 7.1 Hz, 2H), 3.95 (s, 1H), 1.91 – 1.84 (m, 1H), 1.31 (t, ^{3}J = 7.1 Hz, 3H), 0.97 – 0.89 (m, 2H), 0.70 – 0.65 (m, 2H).

^{13}C-NMR (126 MHz, DMSO-d_6): δ = 164.3, 160.9, 159.9, 154.0, 147.8, 145.7, 140.1, 128.1, 123.6, 119.7, 119.5, 99.2, 93.3, 61.2, 21.8, 14.1, 7.8, 6.8.

LCMS: R_t = 6.26 min (method F); purity = 97%.

MS(ESI) [m/z]: calculated = 404.18 $[M+H]^+$; found = 404.2 $[M+H]^+$.

HRMS: calculated = 404.1829 $[M+H]^+$; found = 404.1826 $[M+H]^+$.

2-((4-(cyanomethyl)phenyl)amino)-6-((5-cyclopropyl-1H-pyrazol-3-yl)amino)pyrimidine-4-carboxylic acid (19)

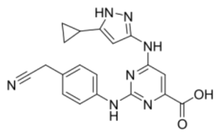

Ethyl 2-((4-(cyanomethyl)phenyl)amino)-6-((5-cyclopropyl-1H-pyrazol-3-yl)amino)pyrimidine-4-carboxylate (**5**) (0.20 g, 0.50 mmol, 1.00 eq.) and lithium hydroxide monohydrate (104 mg, 2.48 mmol, 5.00 eq.) were suspended in 5 mL of a methanol/water mixture (4:1) and the resulting mixture was stirred for 1 h at room temperature. The mixture was then carefully acidified using 5% $HCl_{aqu.}$ to adjust the pH to ~ 6. All volatiles were then removed under reduced pressure and the resulting crude was coevaporated with acetone twice. The title compound was obtained as a pale yellow solid (quant. yield) and used without further purification in follow-up reactions.

LCMS: R_t = 3.50 min (method E).

MS(ESI) [m/z]: calculated = 376.15 [M]; found = 376.2 $[M+H]^+$.

tert-butyl (1-(2-((4-(cyanomethyl)phenyl)amino)-6-((5-cyclopropyl-1H-pyrazol-3-yl)amino)pyrimidin-4-yl)-1-oxo-6,9,12-trioxa-2-azapentadecan-15-yl)carbamate (5-L)

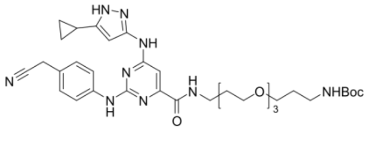

2-((4-(cyanomethyl)phenyl)amino)-6-((5-cyclopropyl-1H-pyrazol-3-yl)amino)pyrimidine-4-carboxylic acid (**19**) (189 mg, 49.6 mmol, 1.00 eq.), HATU (264 mg, 69.4 mmol, 1.40 eq.) and DIPEA (121 µL, 69.4 mmol, 1.40 eq.) were dissolved in 1 mL dry DMF and the resulting mixture was stirred for 20 minutes at room temperature. Tert-butyl (3-(2-(2-(3-aminopropoxy)ethoxy)ethoxy)propyl)carbamate (**20**) (191 mg, 59.5 mmol, 1.20 eq.) was

dissolved in 1 mL dry DMF and added to the reaction mixture. The resulting mixture was stirred at room temperature overnight. The mixture was then diluted with ethyl acetate and washed with water followed by brine. The combined organic layers were dried over $MgSO_4$, filtered and all volatiles were removed under reduced pressure. The obtained crude was loaded unto celite and purified via reverse phase flash column chromatography using acetonitrile/water (5% → 100%) as eluent to give the title compound as a pale yellow solid (222 mg, 66%).

^{1}H-NMR (500 MHz, DMSO-*d*$_6$): δ = 12.53 – 12.02 (m, 1H), 10.50 – 9.72 (m, 1H), 9.23 (s, 1H), 8.19 (t, ^3J = 5.6 Hz, 1H), 7.99 – 7.66 (m, 2H), 7.26 (d, ^3J = 8.2 Hz, 2H), 7.11 – 5.63 (m, 2H), 6.71 (t, ^3J = 5.1 Hz, 1H), 3.96 (s, 1H), 3.51 – 3.46 (m, 8H), 3.44 – 3.41 (m, 2H), 3.35 (t, ^3J = 6.4 Hz, 4H), 2.96 – 2.92 (m, 2H), 1.91 – 1.84 (m, 1H), 1.79 – 1.73 (m, 2H), 1.57 (quin, 2H), 1.36 (s, 9H), 1.00 – 0.88 (m, 2H), 0.71 – 0.65 (m, 2H).

^{13}C-NMR (126 MHz, DMSO-*d*$_6$): δ = 163.5, 161.1, 159.0, 156.3, 155.6, 147.8, 145.6, 140.0, 128.2, 123.7, 119.9, 119.5, 119.0, 96.4, 93.3, 77.4, 69.74, 69.71, 69.6, 69.5, 68.4, 68.1, 37.2, 36.4, 29.7, 29.2, 28.2, 21.8, 7.7, 6.8.

LCMS: R_t = 7.23 min (method F); purity = 95%.

MS(ESI) [m/z]: calculated = 678.37 $[M+H]^+$; found = 678.5 $[M+H]^+$.

2-((2,5-dichloropyrimidin-4-yl)amino)benzamide (23)

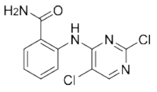

To a solution of 2,4,5-trichloropyrimidine (**22**) (7.00 g, 38.2 mmol, 1.0eq) in propan-2-ol (100 mL) were added 2-aminobenzamide (**21**) (6.24 g, 45.8 mmol, 1.2eq) and N-Ethyldiisopropylamine (7.98 mL, 45.8 mmol, 1.2eq) and the resulting mixture was refluxed for 18 hours. The suspension was filtered, the precipitate was washed with IPA (10 mL) and the solid was collected. The intermediate (**23**) was isolated as a pale yellow solid (10.2 g, 94%). The title compound was used without further steps for the next step.

tert-butyl 4-(4-((4-((2-carbamoylphenyl)amino)-5-chloropyrimidin-2-yl)amino)phenyl)piperazine-1-carboxylate (25)

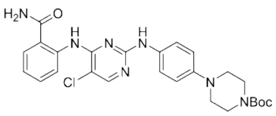

To a solution of 2-[(2,5-dichloropyrimidin-4-yl)amino]benzamide (**23**) (2.04 g, 7.21 mmol, 1.0eq) in DMF (30 mL) were added 4-(4-aminophenyl)piperazine-1-carboxylic acid tert-butyl ester (**24**) (2.00 g, 7.21 mmol, 1.0eq), DIPEA (2.51 mL, 14.4 mmol, 2.0eq) and the resulting mixture was stirred at 90°C for 33 hours and then at 95 °C for 27h and then at 110 °C for 24

h. The solvent was removed under reduced pressure and the residue was purified by reversed phase flash column chromatography (H_2O/ACN). The title compound was isolated as a white solid.

^{1}H NMR (500 MHz, DMSO-*d₆*): δ = 11.84 (s, 1H), 9.23 (s, 1H), 8.79 (s, 1H), 8.28 (s, 1H), 8.16 (s, 1H), 7.82 (dd, ^3J = 7.9, 1.3 Hz, 1H), 7.72 (s, 1H), 7.53 – 7.44 (m, 3H), 7.14 – 7.07 (m, 1H), 6.91 (d, ^3J = 9.0 Hz, 2H), 3.51 – 3.42 (m, 4H), 3.06 – 3.00 (m, 4H), 1.42 (s, 9H).
^{13}C NMR (126 MHz, DMSO-d_6): δ = 171.0, 158.0, 155.0, 154.6, 153.8, 146.4, 140.0, 132.8, 131.8, 128.6, 121.6, 121.2, 121.1, 119.8, 116.5, 104.4, 78.9, 49.3, 28.1.
LCMS: R_t = 4.12 min (method A).

MS(ESI) [m/z]: calculated = 524.22 $[M+Na+ACN]^+$; found = 524.3 $[M+Na+ACN]^+$.

2-((5-chloro-2-((4-(piperazin-1-yl)phenyl)amino)pyrimidin-4-yl)amino)benzamide (6)

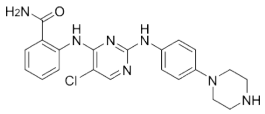

A solution of tert-butyl 4-(4-((4-((2-carbamoylphenyl)amino)-5-chloropyrimidin-2-yl)amino)phenyl)piperazine-1-carboxylate (**25**) (100 mg, 191 µM) in Dioxane/HCl (4 mL, 2 M) was stirred for 6 h. All volatiles were removed under reduced pressure, DCM and TEA (0.5 mL) were added and all volatiles were removed under reduced pressure. The title compound was isolated as colorless solid (58 mg, 72%) using reversed phase flash column chromatography twice (H_2O/ACN; amino-functionalized column).

^{1}H NMR (500 MHz, DMSO-d_6): δ = 11.87 (s, 1H), 9.28 (s, 1H), 8.88 – 8.72 (m, 2H), 8.29 (s, 1H), 8.17 (s, 1H), 7.82 (dd, ^3J = 7.9 Hz, ^4J = 1.5 Hz, 1H), 7.73 (s, 1H), 7.53 (d, ^3J = 8.9 Hz, 2H), 7.50 – 7.45 (m, 1H), 7.13 – 7.09 (m, 1H), 6.98 – 6.93 (m, 2H), 3.30 – 3.23 (m, 8H).
^{13}C NMR (126 MHz, DMSO-d_6): δ = 171.5, 158.4, 155.5, 155.1, 145.8, 140.5, 133.8, 132.3, 129.1, 122.1, 121.63, 121.56, 120.2, 117.0, 105.1, 46.9, 43.4.
LCMS: R_t = 2.98 min (method A); purity = 98%.

MS(ESI) [m/z]: calculated = 424.2 $[M+H]^+$; found = 424.1 $[M+H]^+$.

HRMS: calculated = 424.1647 $[M+H]^+$; found = 424.1638 $[M+H]^+$.

II. Promiscuous Kinase PROTACs

II.a Promiscuous Kinase PROTACs based on Kinase Parent Inhibitor 4

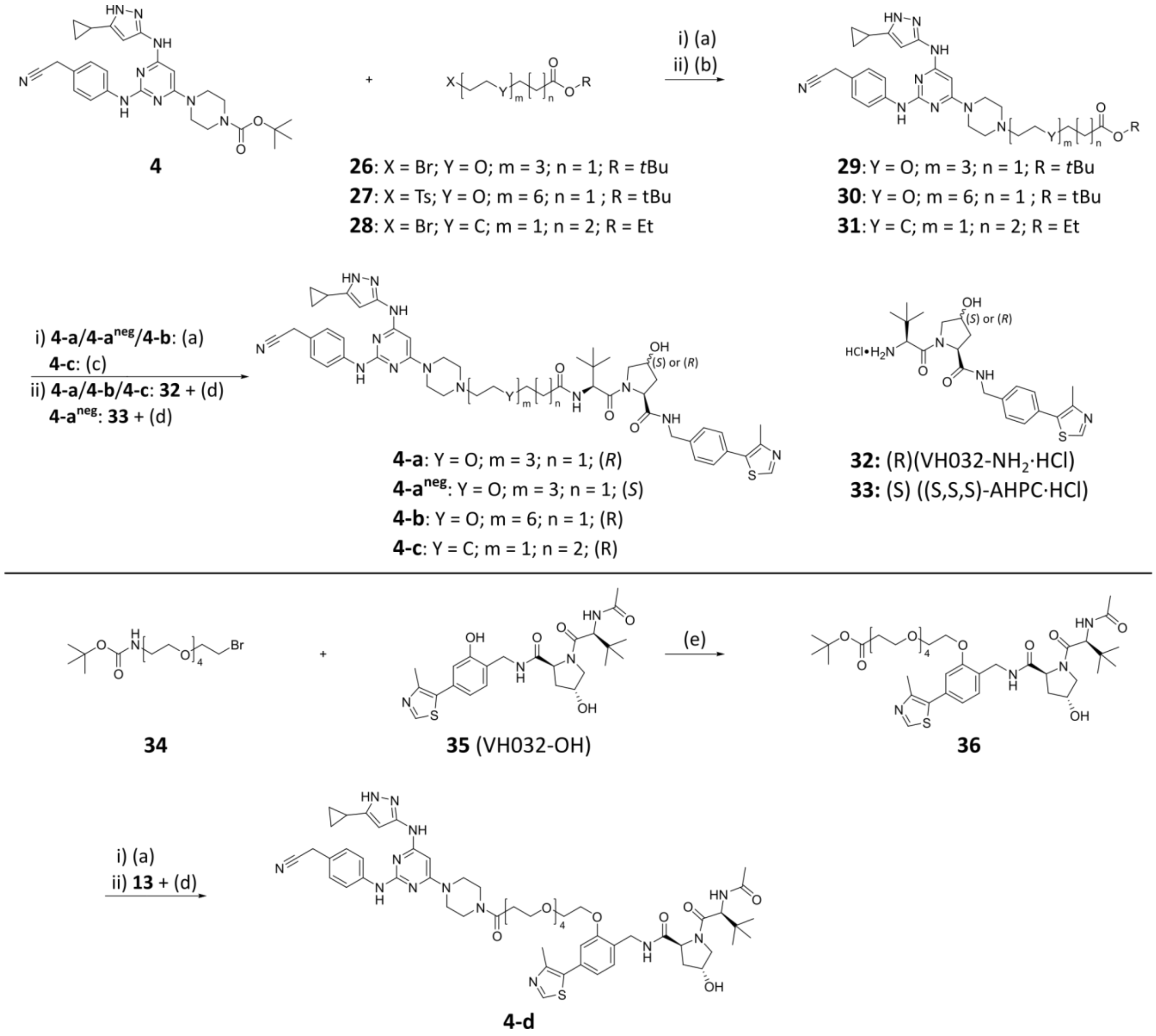

Based on Kinase Parent Warhead 4

Scheme S2: Synthesis routes of all PROTACs based on kinase parent inhibitor 4. Reagents and conditions: (a) 25vol% TFA/DCM, r.t., 20 min - 3 h; (b) K_2CO_3, MeCN, 80 °C, overnight; (c) $LiOH \cdot H_2O$, MeOH/H_2O (4:1), r.t.,1 - 1.5 h; (d) HATU, DIPEA, DMF, r.t., 1 h -overnight; (e) K_2CO_3, DMF, 50 °C, overnight.

Tert-butyl 3-(2-(2-(2-(4-(2-((4-(cyanomethyl)phenyl)amino)-6-((5-cyclopropyl-1H-pyrazol-3-yl)amino)pyrimidin-4-yl)piperazin-1-yl)ethoxy)ethoxy)ethoxy)propanoate (29)

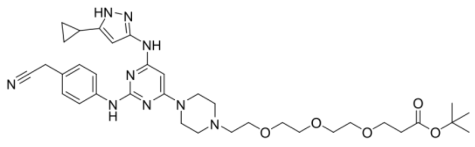

tert-butyl 4-(2-((4-(cyanomethyl)phenyl)amino)-6-((5-cyclopropyl-1H-pyrazol-3-yl)amino)pyrimidin-4-yl)piperazine-1-carboxylate (**4**) (50 mg, 0.94 mmol, 1.0 eq.) was dissolved in 1 mL of a 25vol% solution of trifluoroacetic acid/dichloromethane. The resulting mixture was stirred at room temperature for 30 min. All volatiles were removed under reduced pressure and the residue was coevaporated with dichloromethane twice to give the according trifluoroacetate salt. The trifluoroacetate salt, potassium carbonate (40 mg, 2.9 mmol, 3.0 eq.) and *tert*-butyl 3-(2-(2-(2-bromoethoxy)ethoxy)ethoxy)propanoate (**26**) (66 mg, 0.19 mmol, 2.0 eq.) were then suspended in 1 mL dry acetonitrile and the resulting mixture was stirred at 80 °C overnight. After cooling to room temperature, all volatiles were removed under reduced pressure. The crude was loaded unto celite and purified via reverse phase flash column chromatography using acetonitrile/water (5% → 100%) as eluent to give the title compound as a pale yellow resin (55 mg, 83%).

^{1}H-NMR (500 MHz, DMSO-*d$_6$*): δ = 12.48 – 11.70 (m, 1H), 9.79 – 8.43 (m, 2H), 7.80 – 7.56 (m, 2H), 7.26 – 7.05 (m, 2H), 3.92 (s, 2H), 3.60 – 3.44 (m, 18H), 2.53 – 2.47 (m, 2H), 2.41 (t, ^3J = 6.2 Hz, 2H), 1.88 – 1.78 (m, 1H), 1.39 (s, 9H), 0.93 – 0.83 (m, 2H), 0.71 – 0.62 (m, 2H).

^{13}C-NMR (126 MHz, DMSO-*d$_6$*): δ = 170.4, 163.2, 160.6, 158.7, 140.9, 128.1, 122.4, 119.6, 118.8, 92.4, 80.0, 77.0, 70.4, 69.8, 69.70, 69.68, 69.7, 69.6, 68.3, 66.2, 57.3, 52.8, 50.0, 43.9, 35.9, 32.2, 28.5, 27.7, 21.7, 7.7.

LCMS: R_t = 4.11 min (method E).

MS (ESI) [m/z]: calculated = 676.39 $[M+H]^+$; found = 676.5 $[M+H]^+$.

(2S,4R)-1-((S)-14-(tert-butyl)-1-(4-(2-((4-(cyanomethyl)phenyl)amino)-6-((5-cyclopropyl-1H-pyrazol-3-yl)amino)pyrimidin-4-yl)piperazin-1-yl)-12-oxo-3,6,9-trioxa-13-azapentadecan-15-oyl)-4-hydroxy-N-(4-(4-methylthiazol-5-yl)benzyl)pyrrolidine-2-carboxamide (4-a)

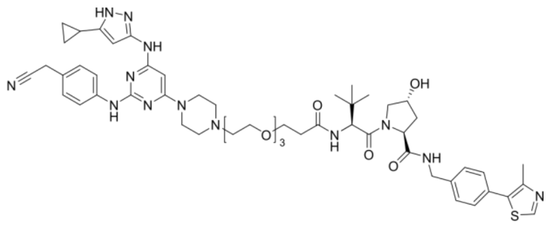

Tert-butyl-3-(2-(2-(2-(4-(2-((4-(cyanomethyl)phenyl)amino)-6-((5-cyclopropyl-1H-pyrazol-3-yl)amino)pyrimidin-4-yl)piperazin-1-yl)ethoxy)ethoxy)ethoxy)propanoate (**29**) (30 mg, 0.44 mmol, 1.0 eq.) was dissolved in 2 mL of a 25vol% solution trifluoroacetic acid/dichloromethane. The resulting mixture was stirred at room temperature for 30 min. It was then diluted with ethyl acetate and carefully washed with sat. aqueous $NaHCO_3$ solution. The combined organic layers were dried over $MgSO_4$, filtered and all volatiles were removed under reduced pressure to give the free acid as a trifluoroacetate salt. The trifluoroacetate salt was then dissolved in 2 mL dry DMF and DIPEA was added (33 µL, 1.9 mmol, 3.4 eq.) followed by HATU (24 mg, 0.62 mmol, 1.4 eq.) and the resulting mixture was stirred at room temperature for 25 min. A solution of (2S,4R)-1-((S)-2-(chloro-l5-azaneyl)-3,3-dimethylbutanoyl)-4-hydroxy-N-(4-(4-methylthiazol-5-yl)benzyl)pyrrolidine-2-carboxamide (**32**, VH032-NH_2·HCl) (25 mg, 0.53 mmol, 1.2 eq.) and DIPEA (8 µL, 0.4 mmol, 1 eq.) in 0.5 mL dry DMF was prepared and added to the reaction mixture. The mixture was stirred at room temperature overnight. The mixture was then diluted with ethyl acetate and washed with water (twice). The combined organic layers were dried over $MgSO_4$, filtered and all volatiles were removed under reduced pressure. The obtained crude was loaded unto celite and purified via reverse phase flash column chromatography using acetonitrile/water (5% → 100%) as eluent first followed by a second purification step via flash column chromatography using methanol/dichloromethane (0% → 10%) as eluent to give the title compound as pale yellow solid (7 mg, 15%).

^{1}H-NMR (500 MHz, DMSO-*d$_6$*): δ = 12.44 – 11.55 (m, 1H), 9.67 – 8.65 (m, 2H), 8.97 (s, 1H), 8.55 (t, ^{3}J = 5.9 Hz, 1H), 7.90 (d, ^{3}J = 9.4 Hz, 1H), 7.83 (d, ^{3}J = 11.7 Hz, 1H), 7.80 – 7.76 (m, 2H), 7.43 – 7.36 (m, 4H), 7.20 (d, ^{3}J = 8.2 Hz, 2H), 6.30 – 5.51 (m, 2H), 5.12 (s, 1H), 4.55 (d, ^{3}J = 9.4 Hz, 1H), 4.46 – 4.40 (m, 2H), 4.37 – 4.33 (m, 1H), 4.22 (dd, ^{2}J = 15.9 Hz, ^{3}J =5.4 Hz, 1H), 3.95 – 3.78 (m, 2H), 3.70 – 3.41 (m, 22H), 2.57 – 2.53 (m, 1H), 2.44 (s, 3H), 2.38 – 2.33 (m, 1H), 2.06 – 2.00 (m, 1H), 1.93 – 1.87 (m, 1H), 1.86 – 1.80 (m, 1H), 0.93 (s, 9H), 0.90 – 0.86 (m, 2H), 0.68 – 0.64 (m, 2H).

^{13}C-NMR (126 MHz, DMSO-*d$_6$*): δ = 171.9, 169.9, 169.5, 163.2, 160.4, 158.6 151.4, 147.7, 140.8, 139.5, 131.1, 129.6, 128.6, 128.1, 127.4, 122.4, 119.6, 118.8, 69.74, 69.70, 69.65, 69.5, 68.9, 68.2, 66.9, 58.7, 57.2, 56.34, 56.28, 52.8, 43.9, 41.6, 37.9, 35.7, 35.4, 26.3, 21.7, 15.9, 7.6.

LCMS: R_t = 3.71 min (method G); purity = 96%.

MS(ESI) [m/z]: calculated = 1032.52 $[M+H]^+$; found = 1032.5 $[M+H]^+$.

HRMS: calculated = 1032.5236 $[M+H]^+$; found = 1032.5222 $[M+H]^+$.

(2S,4S)-1-((S)-14-(tert-butyl)-1-(4-(2-((4-(cyanomethyl)phenyl)amino)-6-((5-cyclopropyl-1H-pyrazol-3-yl)amino)pyrimidin-4-yl)piperazin-1-yl)-12-oxo-3,6,9-trioxa-13-azapentadecan-15-oyl)-4-hydroxy-N-(4-(4-methylthiazol-5-yl)benzyl)pyrrolidine-2-carboxamide (4-a^{neg})

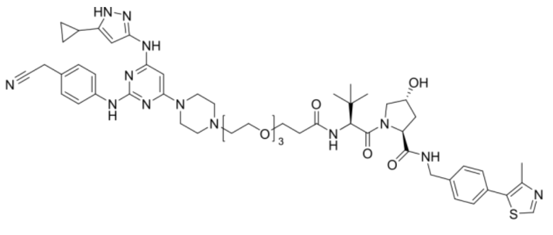

Tert-butyl 3-(2-(2-(2-(4-(2-((4-(cyanomethyl)phenyl)amino)-6-((5-cyclopropyl-1H-pyrazol-3-yl)amino)pyrimidin-4-yl)piperazin-1-yl)ethoxy)ethoxy)ethoxy)propanoate (**29**) (52 mg, 0.077 mmol, 1.0 eq.) was dissolved in 2 mL of a 25vol% solution trifluoroacetic acid/dichloromethane. The resulting mixture was stirred at room temperature for 2 h. All volatiles were removed under reduced pressure and the residue was coevaporated with dichloromethane twice to give the free acid as the trifluoroacetate salt. The trifluoroacetate salt was then dissolved in 1 mL dry DMF and DIPEA was added (50 µL, 0.29 mmol, 3.8 eq.) followed by HATU (35 mg, 0.092 mmol, 1.2 eq.) and the resulting mixture was stirred at room temperature for 5 min. A solution of (2S,4S)-1-((S)-2-(chloroamino)-3,3-dimethylbutanoyl)-4-hydroxy-N-(4-(4-methylthiazol-5-yl)benzyl)pyrrolidine-2-carboxamide (**33**, (S,S,S)-AHPC·HCl) (43 mg, 0.092 mmol, 1.2 eq.) and DIPEA (13 µL, 0.077 mmol, 1.0 eq.) in 0.5 mL dry DMF was prepared and added to the reaction mixture. The mixture was stirred at room temperature for 1 h. All volatiles were removed under reduced pressure. The obtained crude was loaded unto celite and purified via flash column chromatography using methanol/dichloromethane (0% → 10%). The isolated crude product was then purified via preparative HPLC using acetonitrile/water (5% → 100%) with 0.1% trifluoroacetic acid additive as eluent to give the title compound as the trifluoroacetic acetate salt. It was then loaded unto celite and immobilized on propylsulfonic acid functionalized silica using a 50:50 mixture of acetonitrile/water and subsequently flushed down from the silica using an ammonia/methanol solution. All volatiles were then removed under reduced pressure to give the title compound as an off-white solid (15 mg, 19%).

^{1}H-NMR (500 MHz, DMSO-*d$_6$*): δ = 12.51 – 11.40 (m, 1H), 9.29 – 8.76 (m, 2H), 8.97 (s, 1H), 8.73 – 8.57 (m, 1H), 7.99 – 7.86 (m, 1H), 7.81 – 7.61 (m, 2H), 7.44 – 7.36 (m, 4H), 7.26 – 7.13 (m, 2H), 6.12 – 5.61 (m, 2H), 5.49 – 5.29 (m, 1H), 4.48 (d, ^{3}J = 8.9 Hz, 1H), 4.44 (dd, ^{2}J = 6.4 Hz, ^{3}J = 15.9 Hz, 1H), 4.40 – 4.32 (m, 1H), 4.26 (dd, ^{2}J = 5.5 Hz, ^{3}J = 15.8 Hz, 1H), 4.23 – 4.18 (m, 1H), 3.92 (s, 2H), 3.70 – 3.39 (m, 20H), 2.56 – 2.52 (m, 1H), 2.44 (s, 3H), 2.38 – 2.29 (m, 2H), 1.87 – 1.80 (m, 1H), 1.77 – 1.71 (m, 1H), 0.95 (s, 9H), 0.90 – 0.85 (m, 2H), 0.69 – 0.61 (m, 2H).

^{13}C-NMR (126 MHz, DMSO-d_6): δ = 172.4, 170.2, 169.8, 163.2, 160.5, 158.7, 151.4, 147.7, 140.9, 139.2, 131.1, 129.7, 128.7, 128.1, 127.4, 122.5, 119.6, 118.8, 69.8, 69.71, 69.66, 69.5, 69.1, 68.2, 66.9, 58.5, 57.2, 56.6, 55.6, 52.8, 43.9, 41.8, 36.9, 35.6, 34.8, 26.3, 21.7, 15.9, 7.7.

LCMS: R_t = 3.66 min (method E); purity = 96%.

MS(ESI) [m/z]: calculated = 1032.52 $[M+H]^+$; found = 1032.5 $[M+H]^+$.

HRMS: calculated = 1032.5236 $[M+H]^+$; found = 1032.5221 $[M+H]^+$.

***Tert*-butyl 1-(tosyloxy)-3,6,9,12,15,18-hexaoxahenicosan-21-oate (27)**

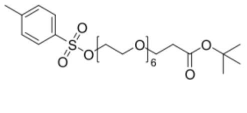

Tert-butyl 1-hydroxy-3,6,9,12,15,18-hexaoxahenicosan-21-oate (50 mg, 0.12 mmol, 1.0 eq.) was dissolved in 0.5 mL dichloromethane and cooled to 0 °C. 4-methylbenzenesulfonyl chloride (46 mg, 0.24 mmol, 2.0 eq.) was added followed by TEA (34 µL, 0.24 mmol, 2.0 eq.) and DMAP (1 mg, 0.009 mmol, 0.07 eq.). The resulting reaction mixture was allowed to warm up to room temperature under stirring overnight. The mixture was then diluted with dichloromethane and washed with sat. aqueous NH_4Cl solution (twice) followed by brine. The combined organic layers were dried over $MgSO_4$, filtered and all volatiles were removed under reduced pressure. The obtained crude was loaded unto celite and purified via flash column chromatography using methanol/dichloromethane (0% → 10%) as eluent to give the title compound as colorless oil (20 mg, 29%).

^{1}H-NMR (500 MHz, DMSO-d_6): δ = 7.80 – 7.77 (m, 2H), 7.49 – 7.47 (m, 2H), 4.12 – 4.10 (m, 2H), 3.59 – 3.56 (m, 4H), 3.50 – 3.47 (m, 16H), 3.44 (s, 4H), 2.42 (s, 3H), 2.40 (t, ^3J = 6.3 Hz, 2H), 1.39 (s, 9H).

^{13}C-NMR (126 MHz, DMSO-d_6): δ = 170.4, 144.9, 132.4, 130.1, 127.6, 79.7, 70.0, 69.8, 69.74, 69.68, 69.6, 67.9, 66.2, 35.8, 27.7, 21.1.

LCMS: R_t = 5.50 min (method E).

MS(ESI) [m/z]: calculated = 587.25 $[M+Na]^+$; found = 587.3 $[M+Na]^+$.

***Tert*-butyl 1-(4-(2-((4-(cyanomethyl)phenyl)amino)-6-((5-cyclopropyl-1H-pyrazol-3-yl)amino)pyrimidin-4-yl)piperazin-1-yl)-3,6,9,12,15,18-hexaoxahenicosan-21-oate (30)**

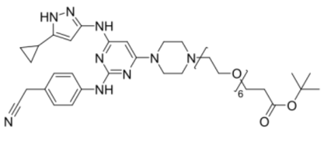

tert-butyl 4-(2-((4-(cyanomethyl)phenyl)amino)-6-((5-cyclopropyl-1H-pyrazol-3-yl)amino)pyrimidin-4-yl)piperazine-1-carboxylate (**4**) (20 mg, 0.35 mmol, 1.0 eq.) was

dissolved in 1 mL of a 25vol% solution of trifluoroacetic acid/dichloromethane. The resulting mixture was stirred at room temperature for 3 h. All volatiles were removed under reduced pressure and the residue was coevaporated with dichloromethane twice to give the according trifluoroacetate salt. The trifluoroacetate salt and potassium carbonate (15 mg, 1.1 mmol, 3.0 eq.) were then suspended in 0.25 mL dry acetonitrile. A solution of *tert*-butyl 1-(tosyloxy)-3,6,9,12,15,18-hexaoxahenicosan-21-oate (**27**) (37 mg, 0.71 mmol, 2.0 eq.) in 0.25 mL dry acetonitrile was added and the resulting mixture was stirred at 80 °C overnight. After cooling to room temperature, the reaction mixture was diluted with ethyl acetate and washed with water (twice) followed by brine. The combined organic layers were dried over $MgSO_4$, filtered and all volatiles were removed under reduced pressure. The obtained crude was loaded unto celite and purified via reverse phase flash column chromatography using acetonitrile/water (5% → 100%) as eluent to give the title compound as a colorless resin (10 mg, 35%).

^{1}H-NMR (500 MHz, DMSO-*d$_6$*): δ = 12.15 – 11.65 (m, 1H), 9.65 – 8.46 (m, 2H), 7.74 – 7.58 (m, 2H), 7.22 -7.08 (m, 2H), 6.30 – 5.51 (m, 2H), 3.92 (s, 2H), 3.62 – 3.40 (m, 32H), 2.54 – 2.51 (m, 2H), 2.40 (t, ^3J = 6.2 Hz, 2H), 1.86 – 1.80 (m, 1H), 1.38 (s, 9H), 0.91 – 0.85 (m, 2H), 0.68 – 0.63 (m, 2H).

^{13}C-NMR (126 MHz, DMSO-*d$_6$*): δ = 170.4, 163.2, 160.5, 158.7, 140.9, 128.7, 128.1, 122.4, 119.6, 118.8, 79.7, 77.0, 69.78, 69.76, 69., 69.6, 68.2, 66.2, 57.2, 52.8, 50., 43.9, 42.4, 35.8, 28.5, 27.74, 21.72, 7.6.

LCMS: R_t = 3.83 min (method E).

MS(ESI) [m/z]: calculated = 808.47 $[M+H]^+$; found = 808.5 $[M+H]^+$.

(2S,4R)-1-((S)-23-(tert-butyl)-1-(4-(2-((4-(cyanomethyl)phenyl)amino)-6-((5-cyclopropyl-1H-pyrazol-3-yl)amino)pyrimidin-4-yl)piperazin-1-yl)-21-oxo-3,6,9,12,15,18-hexaoxa-22-azatetracosan-24-oyl)-4-hydroxy-N-(4-(4-methylthiazol-5-yl)benzyl)pyrrolidine-2-carboxamide (4-b)

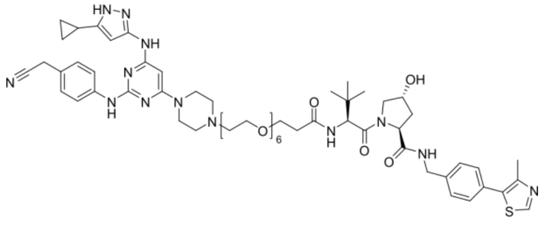

Tert-butyl 1-(4-(2-((4-(cyanomethyl)phenyl)amino)-6-((5-cyclopropyl-1H-pyrazol-3-yl)amino)pyrimidin-4-yl)piperazin-1-yl)-3,6,9,12,15,18-hexaoxahenicosan-21-oate (**30**) (10 mg, 0.12 mmol, 1.0 eq.) was dissolved in 1 mL of a 25vol% solution trifluoroacetic acid/dichloromethane. The resulting mixture was stirred at room temperature for 45 min. All volatiles were removed under reduced pressure and the residue was coevaporated with

dichloromethane twice to give the free acid as the trifluoroacetate salt. The trifluoroacetate salt was then dissolved in 0.25 mL dry DMF and DIPEA was added (13 µL, 0.74 mmol, 6.0 eq.) followed by HATU (5.7 mg, 0.15 mmol, 1.2 eq.) and the resulting mixture was stirred at room temperature for 5 min. A solution of (2S,4R)-1-((S)-2-(chloroamino)-3,3-dimethylbutanoyl)-4-hydroxy-N-(4-(4-methylthiazol-5-yl)benzyl)pyrrolidine-2-carboxamide (**32**, VH032-NH_2·HCl) (7.5 mg, 0.16 mmol, 1.3 eq.) and DIPEA (2.2 µL, 0.12 mmol, 1.0 eq.) in 0.25 mL dry DMF was prepared and added to the reaction mixture. The mixture was stirred at room temperature for 1.5 h. All volatiles were removed under reduced pressure. The obtained crude was loaded unto celite and purified via reverse phase flash column chromatography using acetonitrile/water with 0.1% trifluoroacetic acid additive (5% → 100%) as eluent to give the title compound as pale yellow solid.

^{1}H-NMR (500 MHz, DMSO-*d$_6$*): δ = 10.04 – 9.37 (m, 3H), 8.98 (s, 1H), 8.55 (t, ^3J = 6.0 Hz, 1H), 7.90 (d, ^3J = 9.4 Hz, 1H), 7.65 (d, ^3J = 8.5 Hz, 2H), 7.44 – 7.36 (m, 4H), 7.27 (d, ^3J = 8.5 Hz, 2H), 5.97 – 5.77 (m, 2H), 4.55 (d, ^3J = 9.4 Hz, 1H), 4.45 – 4.40 (m, 2H), 4.36 – 4.33 (m, 1H), 4.22 (dd, ^2J = 5.5 Hz, ^3J = 15.9 Hz, 1H), 3.97 (s, 2H), 3.80 – 3.77 (m, 2H), 3.68 – 3.46 (m, 28H), 3.38 – 3.34 (m, 2H), 33.4 – 3.25 (m, 2H), 3.20 – 3.05 (m, 2H), 2.58 – 2.53 (m, 1H), 2.44 (s, 3H), 2.37 – 2.31 (m, 1H), 2.08 – 1.99 (m, 1H), 1.95 – 1.83 (m, 2H), 0.96 – 0.90 (m, 2H), 0.93 (s, 9H), 0.73 – 0.68 (m, 2H).

^{13}C-NMR (126 MHz, DMSO-*d$_6$*): δ = 171.9, 169.9, 169.5, 162.2, 158.2, 151.4, 147.7, 139.5, 131.2, 129.6, 128.6, 128.4, 127.4, 119.8, 119.5, 76.9, 69.78, 69.74, 69.70, 69.66, 69.6, 69.4, 68.8, 66.9, 63.9, 58.7, 56.3, 56.3, 54.9, 50.6, 41.6, 41.0, 37.9, 35.6, 35.4, 26.3, 21.8, 15.9, 8.0.

LCMS: R_t = 3.66 min (method E); purity = 96%.

MS(ESI) [m/z]: calculated = 1164.60 $[M+H]^+$; found = 1164.6 $[M+H]^+$.

HRMS: calculated = 1164.6023 $[M+H]^+$; found = 1164.60097 $[M+H]^+$.

Ethyl 7-(4-(2-((4-(cyanomethyl)phenyl)amino)-6-((5-cyclopropyl-1H-pyrazol-3-yl)amino)pyrimidin-4-yl)piperazin-1-yl)heptanoate (31)

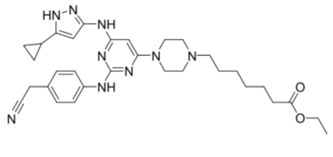

tert-butyl 4-(2-((4-(cyanomethyl)phenyl)amino)-6-((5-cyclopropyl-1H-pyrazol-3-yl)amino)pyrimidin-4-yl)piperazine-1-carboxylate (**4**) (50 mg, 0.097 mmol, 1.0 eq.) was dissolved in 1 mL of a 25vol% solution of trifluoroacetic acid/dichloromethane. The resulting mixture was stirred at room temperature for 20 min. All volatiles were removed under reduced pressure and the residue was coevaporated with dichloromethane twice to give the according

trifluoroacetate salt. The trifluoroacetate salt and potassium carbonate (40 mg, 0.29 mmol, 3.0 eq.) were then suspended in 0.5 mL dry acetonitrile. A solution of ethyl 7-bromoheptanoate (**28**) (46 mg, 0.19 mmol, 2.0 eq.) in 0.5 mL dry acetonitrile was added and the resulting mixture was stirred at 80 °C overnight. After cooling to room temperature, all volatiles were removed under reduced pressure. The obtained crude was loaded unto celite and purified via flash column chromatography using methanol/dichloromethane (0% → 10%) as eluent to give the title compound as a colorless solid (31 mg, 56%).

^{1}H-NMR (500 MHz, DMSO-*d$_6$*): δ = 12.65 – 11.55 (m, 1H), 9.83 – 8.44 (m, 2H), 7.72 (d, ^3J = 8.1 Hz, 2H), 7.21 (d, ^3J = 8.5 Hz, 2H), 6.58 – 5.21 (m, 2H), 4.04 (q, ^3J = 7.1 Hz, 2H), 3.92 (s, 2H), 3.50 – 3.43 (m, 4H), 2.47 – 2.37 (m, 4H), 2.31 – 2.24 (m, 2H), 2.27 (t, ^3J = 7.4 Hz, 2H), 1.86 – 1.80 (m, 1H), 1.55 – 1.49 (m, 2H), 1.47 – 1.40 (m, 2H), 1.30 – 1.25 (m, 4H), 1.17 (t, ^3J = 7.1 Hz, 3H), 0.91 – 0.85 (m, 2H), 0.70 – 0.63 (m, 2H).

^{13}C-NMR (126 MHz, DMSO-*d$_6$*): δ = 172.9, 163.3, 160.5, 158.7, 148.7, 145.7, 140.9, 128.1, 122.4, 119.6, 118.8, 92.2, 77.0, 60.6, 59.6, 57.8, 52.5, 43.9, 33.5, 32.3, 28.4, 28.4, 26.6, 26.0, 25.2, 24.5, 24.4, 21.7, 14.1, 7.67.

LCMS: R_t = 3.72 min (method E).

MS(ESI) [m/z]: calculated = 572.35 $[M+H]^+$; found = 572.4 $[M+H]^+$.

(2S,4R)-1-((S)-2-(7-(4-(2-((4-(cyanomethyl)phenyl)amino)-6-((5-cyclopropyl-1H-pyrazol-3-yl)amino)pyrimidin-4-yl)piperazin-1-yl)heptanamido)-3,3-dimethylbutanoyl)-4-hydroxy-N-(4-(4-methylthiazol-5-yl)benzyl)pyrrolidine-2-carboxamide (4-c)

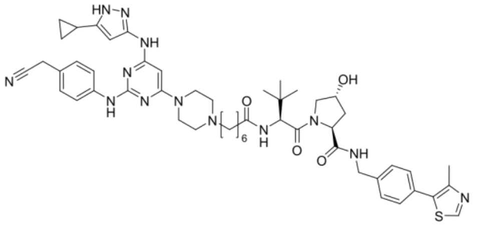

Ethyl 7-(4-(2-((4-(cyanomethyl)phenyl)amino)-6-((5-cyclopropyl-1H-pyrazol-3-yl)amino)pyrimidin-4-yl)piperazin-1-yl)heptanoate (**31**) (40 mg, 0.070 mmol, 1.0 eq.) and lithium hydroxide (15 mg, 0.35 mmol, 5.0 eq.) were suspended in 1 mL of a methanol/water mixture (4:1) and the resulting mixture was stirred at room temperature overnight. The mixture was then carefully acidified using 10% $HCl_{aqu.}$ to adjust the pH to ~ 6. All volatiles were then removed under reduced pressure and the resulting crude was coevaporated with acetone twice. The free acid was obtained as a pale yellow solid (quant. yield) and used without further purification in the following step. 7-(4-(2-((4-(cyanomethyl)phenyl)amino)-6-((5-cyclopropyl-1H-pyrazol-3-yl)amino)pyrimidin-4-yl)piperazin-1-yl)heptanoic acid (39 mg, 0.069 mmol, 1.0 eq.) was suspended in 0.5 mL dry DMF. DIPEA (17 µL, 0.098 mmol, 1.4 eq.) was added

followed by HATU (32 mg, 0.084 mmol, 1.2 eq.) and the resulting mixture was stirred at room temperature for 5 min. A solution of (2S,4R)-1-((S)-2-(chloroamino)-3,3-dimethylbutanoyl)-4-hydroxy-N-(4-(4-methylthiazol-5-yl)benzyl)pyrrolidine-2-carboxamide (**32**, VH032-NH_2·HCl) (39 mg, 0.084 mmol, 1.2 eq.) in 0.5 dry DMF was added and the mixture was stirred at room temperature for 1 h. All volatiles were removed under reduced pressure. The obtained crude was loaded unto celite and purified via reverse phase flash column chromatography to give the title compound as pale yellow solid (25 mg, 37%).

^{1}H-NMR (500 MHz, DMSO-*d₆*): δ = 12.63 – 11.68 (m, 1H), 9.79 – 8.64 (m, 2H), 8.97 (s, 1H), 8.55 (t, ^3J = 6.0 Hz, 1H), 7.84 (d, ^3J = 9.4 Hz, 1H), 7.80 – 7.62 (m, 2H), 7.43 – 7.37 (m, 4H), 7.29 – 7.09 (m, 2H), 6.22 – 5.29 (m, 2H), 5.12 (d, ^3J = 3.0 Hz, 1H), 4.55 (d, ^3J = 9.4 Hz, 1H), 4.46 – 4.39 (m, 2H), 4.38 – 4.33 (m, 1H), 4.22 (dd, ^2J = 5.5 Hz, ^3J = 15.9 Hz, 1H), 3.92 (s, 2H), 3.71 – 3.62 (m, 2H), 3.49 – 3.42 (m, 4H), 2.44 (s, 3H), 2.46 – 2.39 (m, 4H), 2.31 – 2.22 (m, 3H), 2.15 – 2.09 (m, 1H), 2.06 – 2.01 (m, 1H), 1.94 – 1.88 (m, 1H), 1.86 – 1.79 (m, 1H), 1.55 – 1.41 (m, 4H), 1.30 – 1.24 (m, 4H), 0.94 (s, 9H), 0.91 – 0.85 (m, 2H), 0.69 – 0.61 (m, 2H).

^{13}C-NMR (126 MHz, DMSO-*d₆*): δ = 172.1, 171.9, 169.7, 163.3, 160.6, 158.7, 151.4, 147.7, 140.9, 139.5, 131.2, 129.6, 128.6, 128.1, 127.4, 122.4, 119.6, 118.8, 92.4, 77.1, 68.9, 58.7, 57.9, 56.3, 56.3, 52.5, 43.9, 41.7, 38.0, 35.2, 34.9, 28.6, 26.7, 26.4, 26.2, 25.4, 21.7, 15.9, 7.7.

LCMS: R_t = 3.71 min (method A); purity = 99%.

MS(ESI) [m/z]: calculated = 956.51 $[M+H]^+$; found = 956.5 $[M+H]^+$.

HRMS: calculated = 956.5076 $[M+H]^+$; found = 956.5066 $[M+H]^+$.

Tert-butyl 1-(2-(((2S,4R)-1-((S)-2-acetamido-3,3-dimethylbutanoyl)-4-hydroxypyrrolidine-2-carboxamido)methyl)-5-(4-methylthiazol-5-yl)phenoxy)-3,6,9,12-tetraoxapentadecan-15-oate (36)

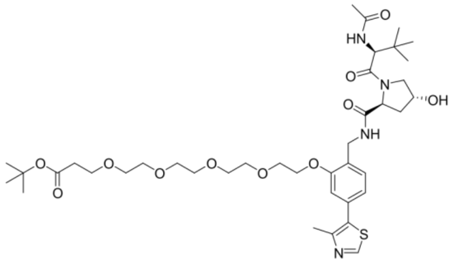

(2S,4R)-1-((S)-2-acetamido-3,3-dimethylbutanoyl)-4-hydroxy-N-(2-hydroxy-4-(4-methylthiazol-5-yl)benzyl)pyrrolidine-2-carboxamide (**35**, VH032-OH) (19 mg, 0.039 mmol, 1.0 eq.) and potassium carbonate (8 mg, 0.06 mmol, 2 eq.) were suspended in 0.35 mL dry DMF and the resulting reaction mixture was stirred at room temperature for 15 min. Tert-butyl 1-bromo-3,6,9,12-tetraoxapentadecan-15-oate (**34**) (17 mg, 0.043 mmol, 1.1 eq.) was added and the mixture was stirred at 50 °C overnight. After cooling to room temperature, all volatiles

were removed under reduced pressure. The obtained crude was loaded unto celite and purified via reverse phase flash column chromatography using acetonitrile/water (5% → 100%) as eluent to give the title compound as an off-white solid (29 mg, 34%).

^{1}H-NMR (500 MHz, DMSO-*d$_6$*): δ = 8.98 (s, 1H), 8.43 (t, ^3J = 6.0 Hz, 1H), 7.95 (d, ^3J = 9.4 Hz, 1H), 7.47 (d, ^3J = 7.8 Hz, 1H), 7.03 (d, ^4J = 1.5 Hz, 1H), 6.92 (dd, ^3J = 7.8 Hz, ^4J = 1.5 Hz, 1H), 4.54 (d, ^3J = 9.4 Hz, 1H), 4.47 (t, ^3J = 8.0 Hz, 1H), 4.36 – 4.31 (m, 2H), 4.19 – 4.12 (m, 3H), 3.80 – 3.78 (m, 2H), 3.67 – 3.64 (m, 2H), 3.63 – 3.60 (m, 3H), 3.58 – 3.53 (m, 5H), 3.52 – 3.44 (m, 6H), 2.46 (s, 3H), 2.39 (t, ^3J = 6.2 Hz, 2H), 2.07 – 2.01 (m, 1H), 1.95 – 1.90 (m, 1H), 1.89 (s, 3H), 1.38 (s, 9H), 0.92 (s, 9H).

^{13}C-NMR (126 MHz, DMSO-*d$_6$*): δ = 172.1, 170.4, 169.7, 169.1, 155.8, 151.4, 147.8, 131.3, 130.7, 127.7, 127.3, 120.9, 112.0, 79.7, 70.1, 69.84, 69.82, 69.78, 69.69, 69.65, 69.0, 68.9, 67.9, 66.2, 58.7, 56.4, 37.9, 37.2, 35.8, 35.2, 27.7, 26.3, 22.3, 16.0.

LCMS: R_t = 4.86 min (method E).

MS(ESI) [m/z]: calculated = 793.41 $[M+H]^+$; found = 793.5 $[M+H]^+$.

(2S,4R)-1-((S)-2-acetamido-3,3-dimethylbutanoyl)-N-(2-((15-(4-(2-((4-(cyanomethyl)phenyl)amino)-6-((5-cyclopropyl-1H-pyrazol-3-yl)amino)pyrimidin-4-yl)piperazin-1-yl)-15-oxo-3,6,9,12-tetraoxapentadecyl)oxy)-4-(4-methylthiazol-5-yl)benzyl)-4-hydroxypyrrolidine-2-carboxamide (4-d)

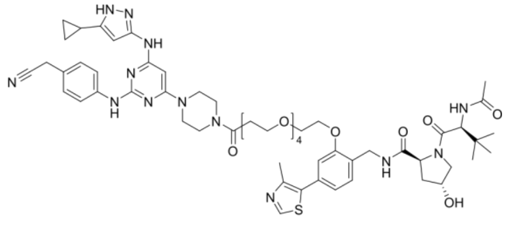

Tert-butyl 1-(2-(((2S,4R)-1-((S)-2-acetamido-3,3-dimethylbutanoyl)-4-hydroxypyrrolidine-2-carboxamido)methyl)-5-(4-methylthiazol-5-yl)phenoxy)-3,6,9,12-tetraoxapentadecan-15-oate (**36**) (24 mg, 0.046 mmol, 1.0 eq.) was dissolved in 2 mL of a 25vol% solution trifluoroacetic acid/dichloromethane. The resulting mixture was stirred at room temperature for 1 h. All volatiles were removed under reduced pressure and the residue was coevaporated with dichloromethane twice to give the free acid as the trifluoroacetate salt. The trifluoroacetate salt was then dissolved in 0.5 mL dry DMF and DIPEA was added (10 µL, 0.058 mmol, 1.4 eq.) followed by HATU (22 mg, 0.058 mmol, 1.4 eq.) and the resulting mixture was stirred at room temperature for 20 min. A solution of 2-(4-((4-((5-cyclopropyl-1H-pyrazol-3-yl)amino)-6-(piperazin-1-yl)pyrimidin-2-yl)amino)phenyl)acetonitrile (**13**) (27 mg, 0.064 mmol, 1.0 eq.) in 1 mL dry DMF was prepared and added to the reaction mixture. The mixture was stirred at room temperature overnight. The reaction mixture was then diluted with ethyl acetate and washed

with water followed by brine. The combined organic layers were dried over $MgSO_4$, filtered and all volatiles were removed under reduced pressure. The obtained crude was loaded unto celite and purified via reverse phase flash column chromatography using acetonitrile/water (5% → 100%) to give the title compound as an off-white solid (37 mg, 78%).

^{1}H-NMR (500 MHz, DMSO-*d₆*): δ = 12.69 – 11.40 (m, 1H), 9.81 – 8.99 (m, 2H), 8.97 (s, 1H), 8.43 (t, ^{3}J = 6.0 Hz, 1H), 7.95 (d, ^{3}J = 9.4 Hz, 1H), 7.70 (d, ^{3}J = 8.2 Hz, 2H), 7.47 (d, ^{3}J = 7.8 Hz, 1H), 7.23 (d, ^{3}J = 8.4 Hz, 2H), 7.02 (d, ^{4}J = 1.5 Hz, 1H), 6.92 (dd, ^{3}J = 7.8 Hz, ^{4}J = 1.5 Hz, 1H), 6.18 – 5.59 (m, 2H), 5.12 (bs, 1H), 4.54 (d, ^{3}J = 9.4 Hz, 1H), 4.47 (t, ^{3}J = 8.0 Hz, 1H), 4.37 – 4.31 (m, 2H), 4.18 – 4.12 (m, 3H), 3.94 (s, 3H), 3.78 – 3.75 (m, 2H), 3.67 – 3.51 (m, 16H), 3.50 – 3.46 (m, 8H), 2.62 (t, ^{3}J = 6.7 Hz, 2H), 2.46 (s, 3H), 2.07 – 2.02 (m, 1H), 1.95 – 1.90 (m, 1H), 1.89 (s, 3H), 1.87 – 1.82 (m, 1H), 0.92 (s, 9H), 0.91 – 0.88 (m, 1H), 0.69 – 0.65 (m, 1H).

^{13}C-NMR (126 MHz, DMSO-*d₆*): δ = 172.1, 169.7, 169.1, 169.1, 162.8, 155.7, 151.4, 147.8, 140.3, 131.3, 130.7, 128.2, 127.7, 127.3, 123.1, 120.9, 119.5, 119.2, 112.0, 76.78, 70.0, 69.80, 69.75, 69.7, 69.0, 68.9, 67.9, 66.8, 58.7, 56.4, 44.4, 44.0, 43.6, 40.5, 38.0, 37.2, 35.2, 32.9, 26.3, 22.3, 21.8, 16.0, 7.8.

LCMS: R_t = 5.195 min (method B); purity = 97%.

MS(ESI) [m/z]: calculated = 1134.56 $[M+H]^+$; found = 1134.6 $[M+H]^+$.

HRMS: calculated = 1134.5553 $[M+H]^+$; found = 1134.5545 $[M+H]^+$.

II.b Promiscuous Kinase PROTACs based on Kinase Parent Inhibitor 5

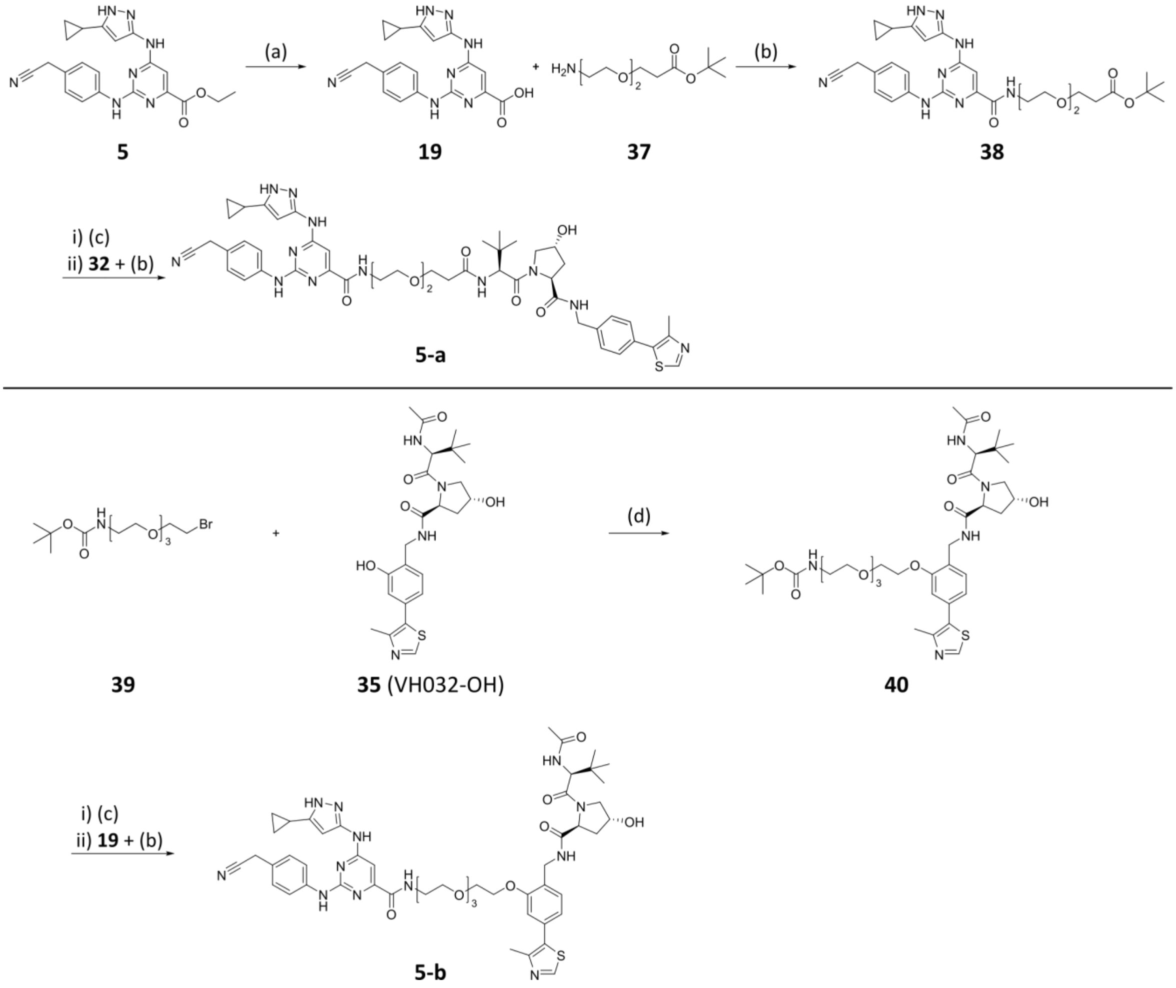

Scheme 3: Synthesis routes of all PROTACs based on kinase parent inhibitor 5. Reagents and conditions: (a) LiOH·H_2O, MeOH/H_2O (4:1), r.t.,1 h; (b) HATU, DIPEA, DMF, r.t., 1 h - overnight; (c) 25vol% TFA/DCM, r.t., 30 min - 1 h; (d) K_2CO_3, MeCN, 50 °C, overnight.

***Tert*-butyl 3-(2-(2-(2-((4-(cyanomethyl)phenyl)amino)-6-((5-cyclopropyl-1H-pyrazol-3-yl)amino)pyrimidine-4-carboxamido)ethoxy)ethoxy)propanoate (38)**

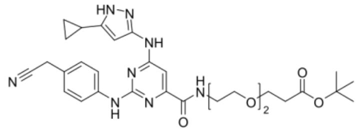

2-((4-(cyanomethyl)phenyl)amino)-6-((5-cyclopropyl-1H-pyrazol-3-yl)amino)pyrimidine-4-carboxylic acid (**19**) (51 mg, 0.14 mmol, 1.0 eq.) was dissolved in 1.5 mL dry DMF. DIPEA (33 µL, 0.19 mmol, 1.4 eq.) was added followed by HATU (72 mg, 0.19 mmol, 1.4 eq.) and the resulting mixture was stirred at room temperature for 20 min. A solution of *tert*-butyl 3-(2-(2-aminoethoxy)ethoxy)propanoate (**37**) (38 mg, 0.16 mmol, 1.2 eq.) in 0.5 dry DMF was added and the mixture was stirred at room temperature overnight. The reaction mixture was then diluted with ethyl acetate and washed with water followed by brine. The combined organic layers were dried over $MgSO_4$, filtered and all volatiles were removed under reduced pressure. The obtained crude was loaded unto celite and purified via reverse phase flash column chromatography using acetonitrile/water (5% → 100%) to give the title compound as a colorless resin (quant. yield).

^{1}H-NMR (500 MHz, DMSO-*d$_6$*): δ = 12.51 – 12.05 (m, 1H), 10.68 – 9.53 (m, 1H), 9.29 (s, 1H), 8.16 (t, ^3J = 5.7 Hz, 1H), 7.81 – 7.62 (m, 2H), 7.27 (d, ^3J = 8.0 Hz, 2H), 7.10 – 6.09 (m, 1H), 3.97 (s, 2H), 3.58 – 3.54 (m, 6H), 3.52 – 3.50 (m, 2H), 3.49 – 3.44 (m, 2H), 2.38 (t, ^3J = 6.2 Hz, 2H), 1.91 – 1.84 (m, 1H), 1.36 (s, 9H), 0.99 – 0.87 (m, 2H), 0.72 – 0.66 (m, 2H).

^{13}C-NMR (126 MHz, DMSO-*d$_6$*): δ = 170.4, 163.4, 161.1, 159.0, 155.7, 147.8, 145.6, 140.0, 128.2, 123.7, 119.9, 119.4, 96.4, 93.3, 79.7, 69.7, 69.5, 68.9, 66.2, 38.7, 35.8, 27.7, 21.8, 7.7, 6.8.

LCMS: R_t = 5.43 min (method E).
MS(ESI) [m/z]: calculated = 591.30 $[M+H]^+$; found = 591.4 $[M+H]^+$.

TLC-ESI [m/z]: calculated = 591.30 $[M+H]^+$; found = 591.2 $[M+H]^+$.

2-((4-(cyanomethyl)phenyl)amino)-6-((5-cyclopropyl-1H-pyrazol-3-yl)amino)-N-(2-(2-(3-(((S)-1-((2S,4R)-4-hydroxy-2-((4-(4-methylthiazol-5-yl)benzyl)carbamoyl)pyrrolidin-1-yl)-3,3-dimethyl-1-oxobutan-2-yl)amino)-3-oxopropoxy)ethoxy)ethyl)pyrimidine-4-carboxamide (5-a)

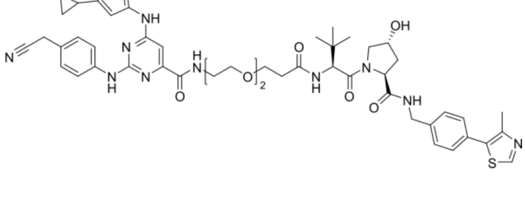

tert-butyl 3-(2-(2-(2-((4-(cyanomethyl)phenyl)amino)-6-((5-cyclopropyl-1H-pyrazol-3-yl)amino)pyrimidine-4-carboxamido)ethoxy)ethoxy)propanoate (**38**) (35 mg, 0.059 mmol, 1.0 eq.) was dissolved in 2 mL of a 25vol% solution trifluoroacetic acid/dichloromethane. The resulting mixture was stirred at room temperature for 30 min. The mixture was then diluted with ethyl acetate and carefully washed with sat. aqueous $NaHCO_3$ solution. The layers were slightly acidified using 10% $HCl_{aqu.}$ and then separated. The combined organic layers were dried over $MgSO_4$, filtered and all volatiles were removed under reduced pressure. The obtained hydrochloride salt was then dissolved in 1 mL dry DMF and DIPEA was added (15 µL, 0.083 mmol, 1.4 eq.) followed by HATU (32 mg, 0.083 mmol, 1.4 eq.) and the resulting mixture was stirred at room temperature for 20 min. A solution of (2S,4R)-1-((S)-2-(chloroamino)-3,3-dimethylbutanoyl)-4-hydroxy-N-(4-(4-methylthiazol-5-yl)benzyl)pyrrolidine-2-carboxamide (**32**, VH032-NH_2·HCl) (33 mg, 0.071 mmol, 1.2 eq.) and DIPEA (10 µL, 0.059 mmol, 1.0 eq.) in 0.5 mL dry DMF was prepared and added to the reaction mixture. The mixture was stirred at room temperature overnight. The reaction mixture was then diluted with ethyl acetate and washed with water followed by brine. The combined organic layers were dried over $MgSO_4$, filtered and all volatiles were removed under reduced pressure. The obtained crude was loaded unto celite and purified via reverse phase flash column chromatography using acetonitrile/water (5% → 100%). The isolated crude product was then purified via preparative HPLC using acetonitrile/water (5% → 100%) with 0.1% trifluoroacetic acid additive as eluent. The isolated trifluoroacetate salt was then dissolved in ethyl acetate and washed with sat. aqueous $NaHCO_3$ solution followed by brine. The combined organic layers were dried over $MgSO_4$, filtered and all volatiles were removed under reduced pressure to give the title compound as a light brown solid (8 mg, 15%).

^{1}H-NMR (500 MHz, DMSO-*d$_6$*): δ = 12.39 – 11.82 (m, 1H), 10.47 – 9.64 (m, 1H), 9.41 – 9.25 (m, 1H), 8.97 (s, 1H), 8.55 (t, ^3J = 6.0 Hz, 1H), 8.16 (t, ^3J = 5.8 Hz, 1H), 7.90 (d, ^3J = 9.4 Hz, 2H), 7.76 – 7.66 (m, 2H), 7.42 – 7.36 (m, 4H), 7.26 (d, ^3J = 8.5 Hz, 2H), 7.06 – 5.82 (m, 2H), 5.12 (d, ^3J = 3.6 Hz, 1H), 4.55 (d, ^3J = 9.4 Hz, 1H), 4.45 – 4.40 (m, 2H), 4.36 – 4.33 (m, 1H), 4.22 (dd, ^2J = 5.6 Hz, ^3J = 15.9 Hz, 1H), 3.97 (s, 2H), 3.68 – 3.44 (m, 12H), 2.53 – 2.51 (m, 1H), 2.44 (s, 3H), 2.38 – 2.32 (m, 1H), 2.06 – 2.01 (m, 1H), 1.92 – 1.84 (m, 2H), 0.94 – 0.90 (m, 2H), 0.92 (s, 9H), 0.69 – 0.66 (m, 2H).

^{13}C-NMR (126 MHz, DMSO-*d$_6$*): δ 171.9, 169.9, 169.5, 163.4, 159.0, 151.4, 147.7, 139.9, 139.5, 131.1, 129.6, 128.6, 128.2, 127.4, 123.7, 119.8, 119.5, 69.5, 7.49, 68.90, 68.85, 66.9, 58.7, 56.35, 56.30, 41.7, 38.7, 37.9, 35.6, 35.3, 26.3, 21.8, 15.9, 7.7, 7.

LCMS: R_t = 4.43 min (method E); purity = 95%.

MS(ESI) [m/z]: calculated = 947.43 $[M+H]^+$; found = 947.5 $[M+H]^+$.

HRMS: calculated = 947.4345 $[M+H]^+$; found = 947.4335 $[M+H]^+$.

Tert-butyl (2-(2-(2-(2-(2-(((2S,4R)-1-((S)-2-acetamido-3,3-dimethylbutanoyl)-4-hydroxypyrrolidine-2-carboxamido)methyl)-5-(4-methylthiazol-5-yl)phenoxy)ethoxy)ethoxy)ethoxy)ethyl)carbamate (40)

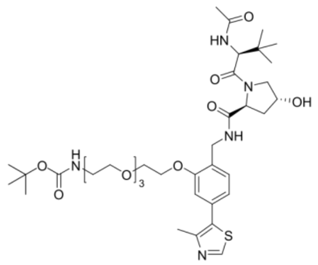

(2S,4R)-1-((S)-2-acetamido-3,3-dimethylbutanoyl)-4-hydroxy-N-(2-hydroxy-4-(4-methylthiazol-5-yl)benzyl)pyrrolidine-2-carboxamide (**35**, VH032-OH) (25 mg, 0.052 mmol, 1.0 eq.) and potassium carbonate (11 mg, 0.077 mmol, 1.5 eq.) were suspended in 0.5 mL dry acetonitrile and the resulting reaction mixture was stirred at room temperature for 10 min. Tert-butyl (2-(2-(2-(2-bromoethoxy)ethoxy)ethoxy)ethyl)carbamate (**39**) (18 mg, 0.052 mmol, 1.0 eq.) was added and the mixture was stirred at 50 °C overnight. After cooling to room temperature, all volatiles were removed under reduced pressure. The obtained crude was loaded unto celite and purified via reverse phase flash column chromatography using acetonitrile/water (5% → 100%) as eluent to give the title compound as an off-white solid (37 mg, 95%).

^{1}H-NMR (500 MHz, DMSO-d_6): δ = 8.98 (s, 1H), 8.43 (t, ^3J = 6.0 Hz, 1H), 7.95 (d, ^3J = 9.4 Hz, 1H), 7.47 (d, ^3J = 7.8 Hz, 1H), 7.03 (d, ^4J = 1.5 Hz, 1H), 6.92 (dd, ^3J = 7.8 Hz, ^4J = 1.5 Hz, 1H), 6.72 (t, ^3J = 5.2 Hz, 1H), 4.54 (d, ^3J = 9.4 Hz, 1H), 4.47 (t, ^3J = 8.0 Hz, 1H), 4.37 – 4.31 (m, 2H), 4.19 – 4.13 (m, 3H), 3.80 – 3.77 (m, 2H), 3.67 – 3.64 (m, 2H), 3.64 – 3.61 (m, 2H), 3.56 – 3.53 (m, 2H), 3.52 – 3.46 (m, 4H), 3.36 (t, ^3J = 6.1 Hz, 2H), 3.057 – 3.03 (m, 2H), 2.46 (s, 3H), 2.07 – 2.02 (m, 1H), 1.95 – 1.90 (m, 1H), 1.89 (s, 3H), 1.36 (s, 9H), 0.92 (s, 9H).

^{13}C-NMR (126 MHz, DMSO-d_6): δ = 172.1, 169.7, 169.1, 155.8, 155.6, 151.4, 147.8, 131.3, 130.8, 127.7, 127.3, 120.9, 112.0, 77.6, 70.1, 69.84, 69.76, 69.5, 69.2, 69.0, 68.9, 67.9, 58.7, 56.4, 37.9, 37.2, 35.2, 28.2, 26.4, 26.3, 22.3, 16.0.

LCMS: R_t = 9.82 min (method F).

MS(ESI) [m/z]: calculated = 764.39 $[M+H]^+$; found = 764.6 $[M+H]^+$.

N-(2-(2-(2-(2-(((2S,4R)-1-((S)-2-acetamido-3,3-dimethylbutanoyl)-4-hydroxypyrrolidine-2-carboxamido)methyl)-5-(4-methylthiazol-5-yl)phenoxy)ethoxy)ethoxy)ethoxy)ethyl)-2-((4-(cyanomethyl)phenyl)amino)-6-((5-cyclopropyl-1H-pyrazol-3-yl)amino)pyrimidine-4-carboxamide (5-b)

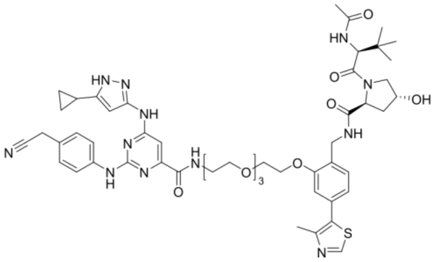

Tert-butyl (2-(2-(2-(2-(((2S,4R)-1-((S)-2-acetamido-3,3-dimethylbutanoyl)-4-hydroxypyrrolidine-2-carboxamido)methyl)-5-(4-methylthiazol-5-yl)phenoxy)ethoxy)ethoxy)ethoxy)ethyl)carbamate (**40**) (45 mg, 0.059 mmol, 1.1 eq.) was dissolved in 2 mL of a 25vol% solution trifluoroacetic acid/dichloromethane. The resulting mixture was stirred at room temperature for 1 h. All volatiles were then removed under reduced pressure and the residue was coevaporated with dichloromethane twice to give the according trifluoroacetate salt. 2-((4-(cyanomethyl)phenyl)amino)-6-((5-cyclopropyl-1H-pyrazol-3-yl)amino)pyrimidine-4-carboxylic acid (**19**) (20 mg, 0.053 mmol, 1.0 eq.) was dissolved in 0.5 mL dry DMF and DIPEA was added (13 µL, 0.075 mmol, 1.4 eq.) followed by HATU (28 mg, 0.075 mmol, 1.4 eq.) and the resulting mixture was stirred at room temperature for 20 min. A solution of the previously obtained amine salt in 1 mL dry DMF was prepared and added to the reaction mixture. The mixture was stirred at room temperature overnight. The reaction mixture was then diluted with ethyl acetate and washed with water followed by brine. The combined organic layers were dried over $MgSO_4$, filtered and all volatiles were removed under reduced pressure. The obtained crude was loaded unto celite and purified via reverse phase flash column chromatography using acetonitrile/water (5% → 100%) to give the title compound as a pale-yellow powder (15 mg, 28%).

^{1}H-NMR (500 MHz, DMSO-*d*$_6$): δ = 12.38 – 11.79 (m, 1H), 10.38 – 9.78 (m, 1H), 9.54 – 9.16 (m, 1H), 8.96 (s, 1H), 8.43 (t, ^{3}J = 6.1 Hz, 1H), 8.17 (t, ^{3}J = 5.6 Hz, 1H), 7.96 (d, ^{3}J = 9.4 Hz, 1H), 7.73 – 7.66 (m, 2H), 7.46 (d, ^{3}J = 7.8 Hz, 1H), 7.25 (d, ^{3}J = 8.5 Hz, 2H), 7.01 (d, ^{4}J = 1.5 Hz, 1H), 6.90 (dd, ^{3}J = 7.8 Hz, ^{4}J = 1.5 Hz, 1H), 6.70 – 5.87 (m, 1H), 5.36 – 4.82 (m, 1H), 4.54 (d, ^{3}J = 9.5 Hz, 1H), 4.46 (t, ^{3}J = 8.1 Hz, 1H), 4.36 – 4.30 (m, 2H), 4.16 – 4.11 (m, 3H), 3.96 (s, 2H), 3.76 – 3.74 (m, 2H), 3.66 – 3.64 (m, 2H), 3.60 – 3.58 (m, 2H), 3.57 – 3.52 (m, 8H), 3.48 – 3.44 (m, 2H), 2.44 (s, 3H), 2.07 – 2.02 (m, 1H), 1.94 – 1.90 (m, 1H), 1.88 (s, 3H), 1.87 – 1.83 (m, 1H), 0.93 – 0.90 (m, 2H), 0.91 (s, 9H), 0.69 – 0.66 (m, 2H).

^{13}C-NMR (126 MHz, DMSO-*d₆*): δ = 172.1, 169.8, 169.1, 163.3, 158.9, 155.7, 151.4, 147.8, 139.8, 131.3, 130.7, 128.2, 127.6, 127.2, 123.7, 120.8, 119.8, 119.4, 111.9, 70.0, 69.82, 69.76, 69.6, 69.0, 68.9, 68.8, 67.8, 58.7, 56.4, 38.7, 37.9, 37.2, 35.2, 26.3, 22.3, 21.8, 16.0, 7.7.

LCMS: R_t = 4.38 min (method E); purity = 95%.

MS(ESI) [m/z]: calculated = 1021.47 $[M+H]^+$; found = 1021.5 $[M+H]^+$.

HRMS: calculated = 1021.4715 $[M+H]^+$; found = 1021.4719 $[M+H]^+$.

II.c Promiscuous Kinase PROTACs based on Kinase Parent Inhibitor 6

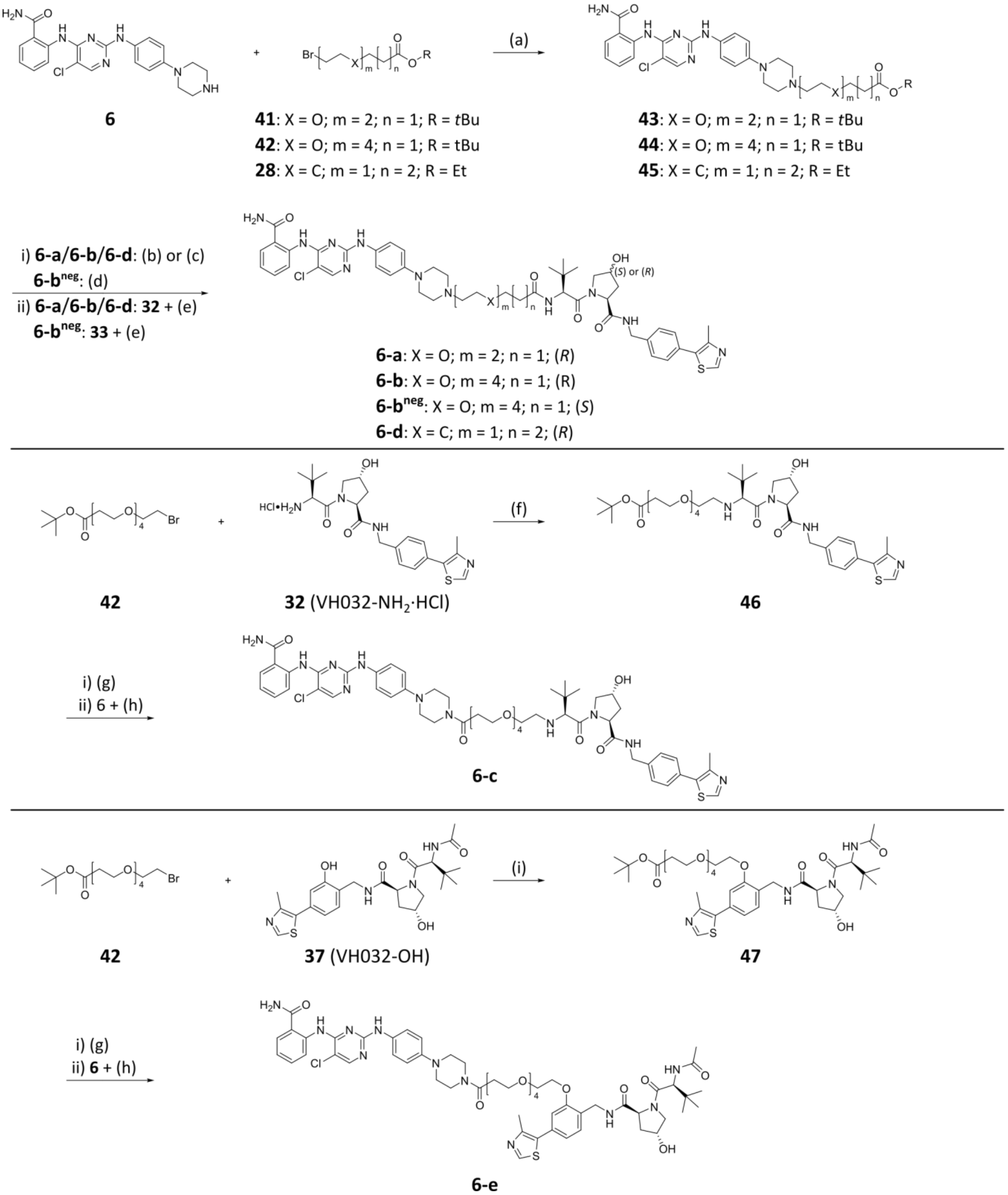

Scheme S4: Scheme 5: Synthesis routes of all PROTACs based on kinase parent inhibitor 6. Reagents and conditions: (a) K_2CO_3, MeCN, 70 - 80 °C, overnight; (b) 50vol% TFA/DCM, r.t., 5 h; (c) 4M HCl in 1,4-dioxane, r.t., 1 – 2 h; (d) LiOH·H_2O, MeOH/H_2O 4:1, r.t., 2 d; (e) HATU, DIPEA, DMF, r.t., overnight; (f) TEA, TBAI, MeCN, 40 °C, overnight ➔ 60 °C, 8 h; (g) 4M HCl in 1,4-dioxane, r.t., overnight; (h) *N*-methylmorpholine, HOBt; EDC·HCl, DMF, r.t., overnight; (i) Cs_2CO_3, MeCN, 50 °C, overnight.

tert-butyl 3-(2-(2-(4-(4-((4-((2-carbamoylphenyl)amino)-5-chloropyrimidin-2-yl)amino)phenyl)piperazin-1-yl)ethoxy)ethoxy)propanoate (43)

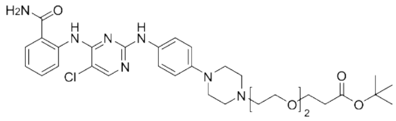

A mixture of 2-((5-chloro-2-((4-(piperazin-1-yl)phenyl)amino)pyrimidin-4-yl)amino)benzamide (**6**) (150 mg, 0.354 mmol, 1.0eq), tert-butyl 3-(2-(2-bromoethoxy)ethoxy)propanoate (**41**) (137 mg, 0.460 mmol, 1.3eq) and K_2CO_3 (147 mg, 1.06 mmol, 3.0eq) in DMF (5 mL) was stirred at 80 °C overnight. DCM and brine were added and the layers were separated. The aqueous layer was extracted with DCM (3x) and the combined organic layers were dried with $MgSO_4$. The solvent was removed under reduced pressure and the crude material was purified using reversed phase flash column chromatography (H_2O/ACN). The title compound was isolated as a beige solid (131 mg, 58%).

^{1}H NMR (500 MHz, DMSO-*d$_6$*): δ = 11.84 (s, 1H), 9.19 (s, 1H), 8.81 (s, 1H), 8.28 (s, 1H), 8.15 (s, 1H), 7.81 (d, ^3J = 7.9 Hz, 1H), 7.72 (s, 1H), 7.49 – 7.42 (m, 3H), 7.10 (td, ^3J = 7.63 Hz, ^4J = 0.90 Hz, 1H), 6.92 – 6.84 (m, 2H), 3.60 (t, ^3J = 6.2 Hz, 2H), 3.54 (t, ^3J = 5.9 Hz, 2H), 3.50 (s, 4H), 3.10 – 3.02 (m, 4H), 2.59 – 2.55 (m, 4H), 2.53 – 2.50 (m, 2H), 2.42 (t, ^3J = 6.2 Hz, 2H), 1.40 (s, 9H).

^{13}C NMR (126 MHz, DMSO-*d$_6$*): δ = 171.0, 170.4, 158.1, 154.9, 154.6, 146.6, 140.0, 132.1, 131.8, 128.5, 121.5, 121.4, 121.1, 119.7, 115.7, 104.3, 79.7, 69.64, 69.62, 68.4, 66.2, 57.3, 53.2, 48.98, 35.9, 27.8.

LCMS: R_t = 3.41 min (method A).

MS(ESI) [m/z]: calculated = 640.3 $[M+H]^+$; found = 640.3 $[M+H]^+$.

(2S,4R)-1-((S)-2-(3-(2-(2-(4-(4-((4-((2-carbamoylphenyl)amino)-5-chloropyrimidin-2-yl)amino)phenyl)piperazin-1-yl)ethoxy)ethoxy)propanamido)-3,3-dimethylbutanoyl)-4-hydroxy-N-(4-(4-methylthiazol-5-yl)benzyl)pyrrolidine-2-carboxamide (6-a)

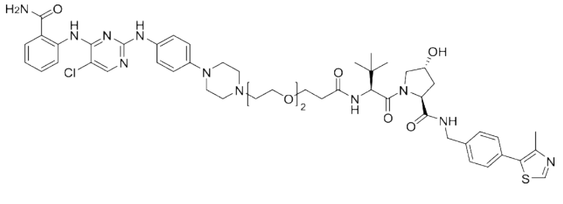

A solution of tert-butyl 3-(2-(2-(4-(4-((4-((2-carbamoylphenyl)amino)-5-chloropyrimidin-2-yl)amino)phenyl)piperazin-1-yl)ethoxy)ethoxy)propanoate (**43**) (15 mg, 23 µmol, 1.0 eq) in DCM/TFA (4 mL, 1/1) was stirred for 5 h and evaporated under reduced pressure. The residue was suspended in DCM and evaporated two times. HATU (11 mg, 28 µmol, 1.2eq) was added to a solution of the acid, VHL-amine-hydrochloride (**32**, VH032-NH_2·HCl) (12 mg, 26 µmol,

1.1eq) and DIPEA (12 µL, 70 µmol, 3.0eq) in DMF (2 mL). The solution was stirred overnight. DCM and brine were added and the layers were separated. The aqueous layer was extracted with DCM (3x) and the combined organic layers were dried with $MgSO_4$. The solvent was removed under reduced pressure and the crude material was purified using reversed phase flash column chromatography (H_2O/ACN). The title compound was isolated as a yellow oil (11 mg, 47%).

^{1}H NMR (500 MHz, DMSO-*d₆*): δ = 11.83 (s, 1H), 9.19 (s, 1H), 8.97 (s, 1H), 8.79 (s, 1H), 8.55 (t, ^3J = 6.1 Hz, 1H), 8.28 (s, 1H), 8.14 (s, 1H), 7.91 (d, ^3J = 9.4 Hz, 1H), 7.81 (dd, ^3J = 7.9 Hz, ^4J = 1.2 Hz, 1H), 7.72 (s, 1H), 7.50-7.35 (m, 7H), 7.12 – 7.08 (m, 1H), 6.89 – 6.85 (m, 2H), 5.13 (d, ^3J = 3.1 Hz, 1H), 4.56 (d, ^3J = 9.4 Hz, 1H), 4.46 – 4.39 (m, 2H), 4.35 (s, 1H), 4.22 (dd, ^2J = 15.9 Hz, ^3J = 5.5 Hz, 1H), 3.70 – 3.46 (m, 10H), 3.10 – 3.02 (m, 4H), 2.59 – 2.51 (m, 7H), 2.44 (s, 3H), 2.40 – 2.33 (m, 1H), 2.06 – 1.99 (m, 1H), 1.94 – 1.87 (m, 1H), 0.94 (s, 9H).
^{13}C NMR (126 MHz, DMSO-*d₆*): δ = 171.9, 171.0, 169.9, 169.5, 158.1, 154.9, 154.6, 151.4, 147.7, 146.6, 140.0, 139.5, 132.1, 131.8, 131.2, 129.6, 128.6, 128.5, 127.4, 121.6, 121.4, 121.1, 119.7, 115.7, 104.3, 69.6, 69.5, 68.9, 68.4, 66.9, 58.7, 57.3, 56.4, 56.3, 53.2, 48.9, 41.7, 37.9, 35.7, 35.4, 26.4, 15.9.
LCMS: R_t = 3.25 min (method A); purity = >99%.

MS(ESI) [m/z]: calculated = 498.7 $[M/2+H]^{2+}$; found = 498.8 $[M/2+H]^{2+}$.

HRMS: calculated = 996.4316 $[M+H]^+$; found = 996.4312 $[M+H]^+$.

tert-butyl 1-(4-(4-((4-((2-carbamoylphenyl)amino)-5-chloropyrimidin-2-yl)amino)phenyl)piperazin-1-yl)-3,6,9,12-tetraoxapentadecan-15-oate (44)

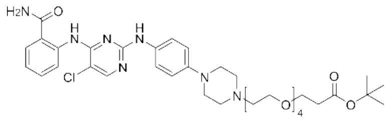

A solution of tert-butyl 4-(4-((4-((2-carbamoylphenyl)amino)-5-chloropyrimidin-2-yl)amino)phenyl)piperazine-1-carboxylate (**6**) (52 mg, 99 µmol, 1eq) in Dioxane/HCl (4 M, 3 mL) was stirred for 5 h and evaporated under reduced pressure. The residue was suspended in DCM and evaporated two times. A mixture of the amine, tert-butyl 1-bromo-3,6,9,12-tetraoxapentadecan-15-oate (**42**) (46 mg, 119 µmol, 1.2eq) and K_2CO_3 (41 mg, 298 µmol, 3.0eq) in DMF (4 mL) was stirred at 70 °C overnight. DCM and brine were added and the layers were separated. The aqueous layer was extracted with DCM (3x) and the combined organic layers were dried with $MgSO_4$. The solvent was removed under reduced pressure and the crude material was purified using reversed phase flash column chromatography (H_2O/ACN). The crude title compound was isolated as a beige solid and used without further purification (60 mg, 83%).

^{1}H NMR (500 MHz, DCM-*d$_2$*): δ = 11.27 (s, 1H), 8.74 (d, ^3J = 8.5 Hz, 1H), 8.04 (s, 1H), 7.64-7.54 (m, 1H), 7.44 (t, ^3J = 7.9 Hz, 1H), 7.40 (d, ^3J = 8.9 Hz, 2H), 7.12 (s, 1H), 7.08 (t, ^3J = 7.5 Hz, 1H), 6.89 (d, ^3J = 8.9 Hz, 2H), 6.38 (s, 1H), 6.00 (s, 1H), 3.69 – 3.65 (m, 2H), 3.63 – 3.55 (m, 12H), 3.17 – 3.13 (m, 4H), 2.67 – 2.64 (m, 4H), 2.62 (t, ^3J = 5.7 Hz, 2H), 2.46 (t, ^3J = 6.5 Hz, 2H), 1.43 (s, 9H).

^{13}C NMR (101 MHz, DCM-*d$_2$*): δ = 171.82, 171.32, 158.99, 156.28, 155.17, 148.43, 141.07, 132.94, 132.26, 128.19, 122.80, 122.67, 122.37, 120.09, 116.88, 106.52, 80.81, 73.17, 71.10, 71.04, 71.00, 70.96, 70.90, 70.81, 70.79, 69.38, 67.40, 67.37, 62.04, 58.46, 54.00, 50.25, 36.87, 28.38.

LCMS: R_t = 3.40 min (method A).

MS(ESI) [m/z]: calculated = 728.4 $[M+H]^+$; found = 728.4 $[M+H]^+$.

(2S,4R)-1-((S)-17-(tert-butyl)-1-(4-(4-((4-((2-carbamoylphenyl)amino)-5-chloropyrimidin-2-yl)amino)phenyl)piperazin-1-yl)-15-oxo-3,6,9,12-tetraoxa-16-azaoctadecan-18-oyl)-4-hydroxy-N-(4-(4-methylthiazol-5-yl)benzyl)pyrrolidine-2-carboxamide (6-b)

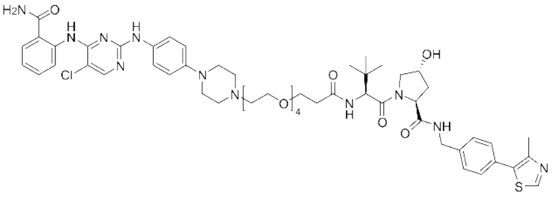

To a solution of tert-butyl 1-(4-(4-((4-((2-carbamoylphenyl)amino)-5-chloropyrimidin-2-yl)amino)phenyl)piperazin-1-yl)-3,6,9,12-tetraoxapentadecan-15-oate (**44**) (152 mg, 209 µmol, 1.0eq) in 1,4-dioxane/water (5.0 mL + 0.5 mL) was added a solution of HCl in dioxane (1.0 mL, 4 M, 19eq) and the resulting mixture was stirred for 1.5 h at r.t followed by 5.0 h at 40 °C. All volatiles were removed under reduced pressure. The crude material was dissolved in DMF (6 mL), treated with TEA (0.5 mL) and all volatiles were removed under reduced pressure. The crude product was used without further purification for the next steps.

To a solution of (2S,4R)-1-((S)-2-amino-3,3-dimethylbutanoyl)-4-hydroxy-N-(4-(4-methylthiazol-5-yl)benzyl)pyrrolidine-2-carboxamide hydrochloride (**32**, VH032-NH_2·HCl) (19 mg, 41 µmol, 1.0eq) in DMF (2 mL) were added the crude acid (TEA salt, 32 mg, 41 µmol, 1.0eq), 1-hydroxybenzotriazole (0.5 mg, 4 µmol, 0.1eq), 4-methylmorpholine (23 µL, 205 µmol, 5.0 eq) and EDC HCl (9 mg, 49 µmol, 1.2eq). The resulting mixture was stirred for 15 h. The solvent was evaporated and the residue was purified using flash column chromatography (DCM/MeOH, then DCM EtOH) twice followed by reversed phase flash column chromatography (H_2O/ACN). The title compound was isolated as colorless wax (9 mg, 20%).

^{1}H NMR (500 MHz, $CDCl_3$): δ = 11.25 (s, 1H), 8.64 (s, 1H), 8.60 (d, ^3J = 8.4 Hz, 1H), 8.01 (s, 1H), 7.92 (s, 1H), 7.66 (s, 1H), 7.58 (d, ^3J = 7.9 Hz, 1H), 7.41 (d, ^3J = 8.6 Hz, 2H), 7.37 – 7.28

(m, 5H), 7.04 – 7.00 (m, 2H), 6.82 (d, ^3J = 8.7 Hz, 2H), 6.74 (s, 1H), 6.15 (s, 1H), 4.75 (t, ^3J = 8.1 Hz, 1H), 4.57 (dd, ^2J = 15.0 Hz, ^3J = 6.6 Hz, 1H), 4.52 (s, 1H), 4.49 (d, ^3J = 8.5 Hz, 1H), 4.29 (dd, ^2J = 15.1 Hz, ^3J = 5.1 Hz, 1H), 4.06 (d, ^3J = 11.1 Hz, 1H), 3.94 (s, 2H), 3.66 – 3.53 (m, 18H), 3.45 – 3.07 (m, 9H), 2.45 (s, 3H), 2.38 (ddd, ^2J = 13.0 Hz, ^3J = 8.7 Hz, ^3J = 4.5 Hz, 2H), 2.33 (t, ^3J = 5.7 Hz, 2H), 2.21 (dd, ^2J = 13.2 Hz, ^3J = 7.9 Hz, 1H), 0.97 (s, 9H).

^{13}C NMR (126 MHz, $CDCl_3$): δ = 171.88, 171.59, 171.55, 171.51, 157.86, 155.83, 154.03, 150.38, 148.49, 145.90, 140.33, 138.49, 133.57, 132.46, 131.83, 130.82, 129.44, 128.22, 128.00, 122.41, 122.27, 119.85, 117.73, 106.22, 70.74, 70.60, 70.52, 70.47, 70.43, 70.39, 70.33, 67.28, 59.06, 57.94, 57.10, 56.72, 52.86, 47.97, 43.27, 36.97, 36.69, 35.33, 29.84, 26.61, 16.20.

LCMS: R_t = 3.37 min (method A); purity = 95%.

MS(ESI) [m/z]: calculated = 1084.5 $[M+H]^+$; found = 1084.5 $[M+H]^+$.

HRMS: calculated = 542.7456 $[M/2+H]^{2+}$; found = 542.7467 $[M/2+H]^{2+}$.

(2S,4S)-1-((S)-17-(tert-butyl)-1-(4-(4-((4-((2-carbamoylphenyl)amino)-5-chloropyrimidin-2-yl)amino)phenyl)piperazin-1-yl)-15-oxo-3,6,9,12-tetraoxa-16-azaoctadecan-18-oyl)-4-hydroxy-N-(4-(4-methylthiazol-5-yl)benzyl)pyrrolidine-2-carboxamide (6-b^{neg})

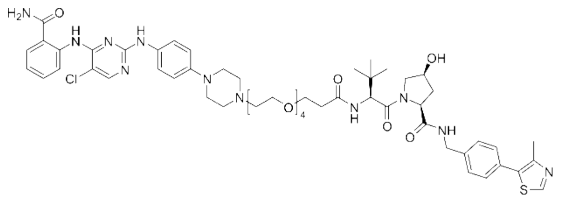

A solution of tert-butyl 1-(4-(4-((4-((2-carbamoylphenyl)amino)-5-chloropyrimidin-2-yl)amino)phenyl)piperazin-1-yl)-3,6,9,12-tetraoxapentadecan-15-oate (**44**) (24 mg, 33 µmol, 1.0eq) in DCM/TFA (4 mL, 1/1) was stirred for 2 h and evaporated under reduced pressure. The residue was suspended in DCM and evaporated two times. HATU (15 mg, 39.5 µmol, 1.2eq) was added to a solution of the acid, nc-VHL-amine (**33**, (S,S,S)-AHPC·HCl) (16 mg, 36 µmol, 1.1eq) and DIPEA (15 µL, 99 µmol, 3.0eq) in DMF (2 mL). The solution was stirred overnight. DCM and brine were added and the layers were separated. The aqueous layer was extracted with DCM (3x) and the combined organic layers were dried with $MgSO_4$. The solvent was removed under reduced pressure and the crude material was purified using reversed phase flash column chromatography (H_2O/ACN). The title compound was isolated as a yellow solid (9 mg, 25%).

^{1}H NMR (500 MHz, DCM-d_2): δ = 11.26 (s, 1H), 8.71 (d, ^3J = 8.2 Hz, 1H), 8.65 (s, 1H), 8.11 (s, 1H), 7.98 (s, 1H), 7.59 (dd, ^3J = 7.9 Hz, ^4J = 1.5 Hz, 1H), 7.44-7.34 (m, 8H), 7.09 – 7.04 (m, 1H), 6.89 (t, ^3J = 6.2 Hz, 2H), 6.73 (d, ^3J = 8.9 Hz, 1H), 6.50 (s, 1H), 5.91 (s, 1H), 5.60 (s, 1H), 4.69 (dd, ^3J = 7.9 Hz, ^4J = 2.2 Hz, 1H), 4.64 (dd, ^2J = 15.1 Hz, ^3J = 7.1 Hz, 1H), 4.51 (d, J = 9.0

Hz, 1H), 4.37 (t, J = 4.1 Hz, 1H), 4.27 (dd, ^2J = 15.1 Hz, ^3J = 5.0 Hz, 1H), 3.84 – 3.76 (m, 2H), 3.69 – 3.56 (m, 16H), 3.18 – 3.11 (m, 4H), 2.69 – 2.64 (m, 4H), 2.61 (t, ^3J = 5.4 Hz, 2H), 2.46 (s, 3H), 2.40 – 2.29 (m, 2H), 2.22 – 2.13 (m, 2H), 0.94 (s, 9H).

^{13}C NMR (126 MHz, DCM-*d₂*): δ = 173.7, 172.4, 171.8, 171.5, 158.9, 156.3, 155.0, 150.7, 149.2, 148.5, 140.9, 138.4, 132.9, 132.3, 132.0, 131.6, 129.9, 128.6, 128.2, 123.1, 122.7, 122.4, 120.1, 116.9, 106.3, 71.7, 71.10, 71.06, 71.00, 70.95, 70.90, 69.4, 67.8, 60.5, 59.1, 58.4, 57.4, 54.1, 50.2, 43.9, 37.3, 36.1, 35.7, 26.7, 16.5.

LCMS: R_t = 5.39 min (method C); purity = >99%.

MS(ESI) [m/z]: calculated = 1084.5 $[M+H]^+$; found = 1084.6 $[M+H]^+$.

HRMS: calculated = 1084.4840 $[M+H]^+$; found = 1084.4841 $[M+H]^+$.

tert-butyl (S)-3-((2S,4R)-4-hydroxy-2-((4-(4-methylthiazol-5-yl)benzyl)carbamoyl)pyrrolidine-1-carbonyl)-2,2-dimethyl-7,10,13,16-tetraoxa-4-azanonadecan-19-oate (TFA salt) (46)

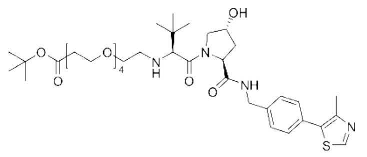

To a solution of (2S,4R)-1-[(2S)-2-amino-3,3-dimethylbutanoyl]-4-hydroxy-N-[[4-(4-methyl-1,3-thiazol-5-yl)phenyl]methyl]pyrrolidine-2-carboxamide;hydrochloride (**32,** VH032-NH_2·HCl) (102 mg, 0.218 mmol, 1.0eq) in ACN (2.0 mL) was added tert-butyl 3-[2-[2-[2-(2-bromoethoxy)ethoxy]ethoxy]ethoxy]propanoate (**42**) (88 mg, 0.23 mmol, 1.1eq), TEA (152 µL, 1.09 mmol, 5.0eq). The resulting mixture was stirred for 3 h and then at 40 °C for 15 h, then at 60 °C for 8 h. Then tetrabutylazanium iodide (15 mg, 0.041 mmol, 0.2eq) was added and the resulting mixture was stirred at 60 °C for 63 h. The solvent was removed under reduced pressure and the residue was purified by flash column chromatography (DCM/EtOH), then by a second flash column chromatography (DCM/MeOH). The crude title compound was isolated as a yellow wax (25 mg, 16%) and used without further purification.

^{1}H NMR (500 MHz, DCM-*d₂*): δ = 8.69 (s, 1H), 8.21 (t, ^3J = 5.7 Hz, 1H), 7.33 (s, 4H), 4.84 (t, ^3J = 8.3 Hz, 1H), 4.52 (s, 1H), 4.48 – 4.34 (m, 3H), 4.00 (d, ^3J = 11.0 Hz, 2H), 3.73 – 3.52 (m, 18H), 3.37 (dd, ^2J = 13.5 Hz, ^3J = 3.4 Hz, 1H), 3.03 (dd, ^2J = 12.0 Hz, ^3J = 9.0 Hz, 1H), 2.50 (s, 3H), 2.48 (t, ^3J = 6.5 Hz, 2H), 2.32 – 2.23 (m, 2H), 1.43 (s, 9H), 1.09 (s, 9H).

^{13}C NMR (126 MHz, DCM-*d₂*): δ = 171.3, 171.1, 167.0, 150.5, 148.3, 138.6, 131.9, 130.7, 129.5, 128.2, 80.9, 70.6, 70.51, 70.49, 70.45, 70.37, 70.2, 70.1, 67.0, 66.9, 59.6, 57.4, 47.1, 43.2, 36.8, 36.3, 35.5, 28.2, 26.6, 16.1.

LCMS: R_t = 3.20 min (method A).

MS(ESI) [m/z]: calculated = 735.4 $[M+H]^+$; found = 735.4 $[M+H]^+$.

(2R,4S)-1-((S)-2-(tert-butyl)-18-(4-(4-((4-((2-carbamoylphenyl)amino)-5-chloropyrimidin-2-yl)amino)phenyl)piperazin-1-yl)-18-oxo-6,9,12,15-tetraoxa-3-azaoctadecanoyl)-4-hydroxy-N-(4-(4-methylthiazol-5-yl)benzyl)pyrrolidine-2-carboxamide (6-c)

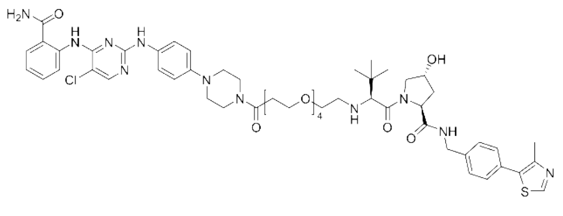

To a solution of tert-butyl (S)-3-((2S,4R)-4-hydroxy-2-((4-(4-methylthiazol-5-yl)benzyl)carbamoyl)pyrrolidine-1-carbonyl)-2,2-dimethyl-7,10,13,16-tetraoxa-4-azanonadecan-19-oate (**46**) (20 mg, 27 µmol, 1.0eq) in 1,4-dioxane (2.0 mL) was added a solution of HCl in dioxane (0.2 mL, 4 M, 29eq) and the resulting mixture was stirred for 16 h at r.t Then, additional HCl in dioxane (0.2 mL, 4M, 29eq) was added and the mixture was stirred for 5 h at 40 °C. All volatiles were removed under reduced pressure. The crude material was dissolved in ACN (1 mL), treated with TEA (0.5 mL) and all volatiles were removed under reduced pressure. The crude product was used without further purification for the next steps.

To a solution of 2-((5-chloro-2-((4-(piperazin-1-yl)phenyl)amino)pyrimidin-4-yl)amino)benzamide (**6**) (12 mg, 27 µmol, 1.0eq) in DMF (2 mL) were added the crude acid (TEA salt, 21 mg, 27 µmol, 1.0eq), 1-hydroxybenzotriazole (0.4 mg, 3 µmol, 0.1eq), 4-methylmorpholine (15 µL, 136 µmol, 5.0 eq) and EDC HCl (6 mg, 33 µmol, 1.2eq). The resulting mixture was stirred for 20 h. The solvent was evaporated and the residue was purified using flash column chromatography (DCM/MeOH, then DCM EtOH) twice followed by reversed phase flash column chromatography (H_2O/ACN). The crude title compound was isolated as colorless wax (7 mg, 24%).

^{1}H NMR (500 MHz, $CDCl_3$): δ = 11.37 (s, 1H), 9.12 (s, 1H), 8.64 (d, ^3J = 8.4 Hz, 1H), 8.63 (s, 1H), 8.02 (s, 1H), 7.64 (d, ^3J = 7.2 Hz, 1H), 7.53 (s, 1H), 7.43 – 7.35 (m, 5H), 7.29 (d, ^3J = 8.2 Hz, 2H), 7.05 (t, ^3J = 7.5 Hz, 1H), 6.86 (d, ^3J = 8.8 Hz, 2H), 6.04 (s, 1H), 5.03 (t, ^3J = 8.0 Hz, 1H), 4.53 – 4.47 (m, 1H), 4.46 – 4.35 (m, 3H), 4.33 – 4.21 (m, 1H), 3.98 (d, ^2J = 11.2 Hz, 1H), 3.79 (t, ^3J = 6.6 Hz, 2H), 3.78 – 3.75 (m, 2H), 3.68 – 3.49 (m, 17H), 3.35 (d, ^2J = 14.0 Hz, 1H), 3.13 – 3.04 (m, 4H), 2.85 – 2.77 (m, 1H), 2.66 (t, ^3J = 6.4 Hz, 2H), 2.47 (s, 3H), 2.25 (t, ^3J = 8.5 Hz, 2H), 1.11 (s, 9H).

^{13}C NMR (126 MHz, $CDCl_3$): δ = 171.46, 171.46, 169.78, 157.54, 155.98, 150.32, 148.46, 147.30, 140.26, 138.80, 132.99, 132.46, 131.92, 130.60, 129.40, 128.26, 128.16, 122.50, 122.42, 119.94, 117.37, 106.37, 70.71, 70.58, 70.52, 70.45, 70.32, 70.13, 67.70, 67.18, 59.64, 57.39, 50.64, 50.07, 46.92, 45.91, 43.03, 41.75, 36.93, 35.70, 33.67, 29.85, 26.89, 16.21.

LCMS: R_t = 7.11 min (method D); purity = 88%.

MS(ESI) [m/z]: calculated = 1084.5 $[M+H]^+$; found = 1084.4 $[M+H]^+$.

HRMS: calculated = 542.7456 $[M/2+H]^{2+}$; found = 542.7452 $[M/2+H]^{2+}$.

ethyl 7-(4-(4-((4-((2-carbamoylphenyl)amino)-5-chloropyrimidin-2-yl)amino)phenyl)piperazin-1-yl)heptanoate (45)

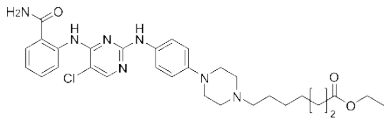

A solution of tert-butyl 4-(4-((4-((2-carbamoylphenyl)amino)-5-chloropyrimidin-2-yl)amino)phenyl)piperazine-1-carboxylate (**6**) (164 mg, 0.313 mmol, 1eq) in DCM/TFA (4 mL, 1/1) was stirred for 6 h and evaporated under reduced pressure. The residue was suspended in DCM and evaporated two times. A mixture of the amine, Ethyl-7-bromoheptanoate (**28**) (111 mg, 0.469 mmol, 1.5eq) and K_2CO_3 (130 mg, 0.939 mmol, 3.0eq) in DMF (5 mL) was stirred at 80 °C overnight. DCM and brine were added and the layers were separated. The aqueous layer was extracted with DCM (3x) and the combined organic layers were dried with $MgSO_4$. The solvent was removed under reduced pressure and the crude material was purified using reversed phase flash column chromatography (H_2O/ACN). The title compound was isolated as a colorless solid (76 mg, 42%).

^{1}H NMR (500 MHz, DMSO-*d6*): δ = 11.84 (s, 1H), 9.19 (s, 1H), 8.80 (s, 1H), 8.28 (s, 1H), 8.15 (s, 1H), 7.81 (dd, ^3J = 7.9 Hz, ^4J = 1.4 Hz, 1H), 7.72 (s, 1H), 7.51 – 7.43 (m, 3H), 7.15 – 7.08 (m, 1H), 6.90 – 6.85 (m, 2H), 4.05 (q, ^3J = 7.1 Hz, 2H), 3.13 – 3.00 (m, 4H), 2.53 – 2.50 (m, 4H), 2.30 (t, ^3J = 7.30 Hz, 2H), 2.27 (t, ^3J = 7.47 Hz, 2H), 1.57 – 1.49 (m, 2H), 1.48 – 1.41 (m, 2H), 1.34 – 1.26 (m, 4H), 1.17 (t, ^3J = 7.1 Hz, 3H).
^{13}C NMR (126 MHz, DMSO-*d6*): δ = 172.9, 171.0, 158.0, 154.9, 154.6, 146.6, 140.0, 132.1, 131.8, 128.5, 121.6, 121.4, 121.1, 119.7, 115.7, 104.3, 59.6, 57.8, 52.8, 48.97, 33.5, 28.4, 26.6, 26.1, 24.4, 14.1.

LCMS: R_t = 3.36 min (method A).

MS(ESI) [m/z]: calculated = 580.3 $[M+H]^+$; found = 580.3 $[M+H]^+$.

(2S,4R)-1-((S)-2-(7-(4-(4-((4-((2-carbamoylphenyl)amino)-5-chloropyrimidin-2-yl)amino)phenyl)piperazin-1-yl)heptanamido)-3,3-dimethylbutanoyl)-4-hydroxy-N-(4-(4-methylthiazol-5-yl)benzyl)pyrrolidine-2-carboxamide (6-d)

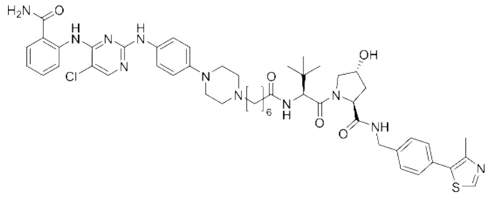

A solution of ethyl 7-(4-(4-((4-((2-carbamoylphenyl)amino)-5-chloropyrimidin-2-yl)amino)phenyl)piperazin-1-yl)heptanoate (**45**) (15 mg, 26 µmol, 1.0eq) and LiOH-H_2O (11 mg, 258 µmol, 10.0eq) in MeOH/H_2O (2 mL, 4/1) was stirred over the weekend. The solution was acidified with HCl (1M) and all volatiles were removed under reduced pressure. HATU (12 mg, 31 µmol, 1.2eq) was added to a solution of the acid, VHL-amine-hydrochloride (**32**, VH032-NH_2·HCl) (13 mg, 28 µmol, 1.1eq) and DIPEA (14 µL, 78 µmol, 3.0eq) in DMF (2 mL). The solution was stirred overnight. DCM and brine were added and the layers were separated. The aqueous layer was extracted with DCM (3x) and the combined organic layers were dried with $MgSO_4$. The solvent was removed under reduced pressure and the crude material was purified using reversed phase flash column chromatography (H_2O/ACN) followed by prep HPLC. The residue was dissolved in DCM and washed with an aqueous solution of Na_2CO_3 twice followed by water once. The title compound was isolated as a beige solid (8 mg, 32%).

^{1}H NMR (500 MHz, DMSO-*d$_6$*): δ = 11.83 (s, 1H), 9.19 (s, 1H), 8.97 (s, 1H), 8.80 (s, 1H), 8.55 (t, ^3J = 6.1 Hz, 1H), 8.28 (s, 1H), 8.15 (s, 1H), 7.84 (d, ^3J = 9.4 Hz, 1H), 7.81 (dd, ^3J = 7.9 Hz, ^4J = 1.4 Hz, 1H), 7.72 (s, 1H), 7.49 – 7.36 (m, 7H), 7.13 – 7.08 (m, 1H), 6.87 (d, ^3J = 9.1 Hz, 2H), 5.12 (d, ^3J = 3.3 Hz, 1H), 4.55 (d, ^3J = 9.4 Hz, 1H), 4.46 – 4.40 (m, 2H), 4.35 (s, 1H), 4.22 (dd, ^2J = 15.9 Hz, ^3J = 5.5 Hz, 1H), 3.70 – 3.62 (m, 2H), 3.10 – 3.04 (m, 4H), 2.49 – 2.47 (m, 4H), 2.44 (s, 3H), 2.32 – 2.23 (m, 3H), 2.15 – 2.09 (m, 1H), 2.06 – 2.00 (m, 1H), 1.91 (ddd, ^2J = 12.9 Hz, ^3J = 8.6 Hz, ^3J = 4.6 Hz, 1H), 1.57 – 1.40 (m, 4H), 1.32 – 1.24 (m, 4H), 0.94 (s, 9H).

^{13}C NMR (126 MHz, DMSO-*d$_6$*): δ = 172.1, 171.9, 171.0, 169.7, 158.1, 154.9, 154.6, 151.4, 147.7, 146.6, 140.0, 139.5, 132.1, 131.8, 131.1, 129.6, 128.6, 128.5, 127.4, 121.6, 121.4, 121.1, 119.7, 115.7, 104.3, 68.9, 58.7, 57.9, 56.33, 56.28, 52.9, 48.9, 41.6, 37.9, 35.2, 34.9, 28.6, 26.7, 26.4, 26.2, 25.4, 15.9.

LCMS: R_t = 3.31 min (method A); purity = >99%.

MS(ESI) [m/z]: calculated = 964.4 $[M+H]^+$; found = 964.5 $[M+H]^+$.

HRMS: calculated = 482.7245 $[M/2+H]^{2+}$; found = 482.7239 $[M/2+H]^{2+}$.

tert-butyl 1-(2-(((2S,4R)-1-((S)-2-acetamido-3,3-dimethylbutanoyl)-4-hydroxypyrrolidine-2-carboxamido)methyl)-5-(4-methylthiazol-5-yl)phenoxy)-3,6,9,12-tetraoxapentadecan-15-oate (47)

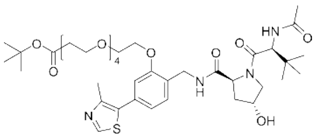

To a solution of (2S,4R)-1-[(2S)-2-acetamido-3,3-dimethylbutanoyl]-4-hydroxy-N-[[2-hydroxy-4-(4-methyl-1,3-thiazol-5-yl)phenyl]methyl]pyrrolidine-2-carboxamide (**37**, VH032-OH) (32 mg, 66 µmol, 1.0eq) in ACN (2.0 mL) was added tert-butyl 3-[2-[2-[2-(2-bromoethoxy)ethoxy]ethoxy]ethoxy]propanoate (**42**) (28 mg, 72 µmol, 1.1eq), cesium;carbonate (32 mg, 98 µmol, 1.5eq) and the resulting mixture was stirred at 50 °C for 13 h. The solvent was removed under reduced pressure and the residue was purified by flash column chromatography (DCM/MeOH). The title compound was isolated as a colorless wax (39 mg, 75%).

^{1}H NMR (500 MHz, DCM-d_2): δ = 8.99 (s, 1H), 8.43 (t, ^3J = 6.0 Hz, 1H), 7.95 (d, ^3J = 9.4 Hz, 1H), 7.47 (d, ^3J = 7.8 Hz, 1H), 7.03 (d, ^4J = 1.5 Hz, 1H), 6.92 (dd, ^3J = 7.8 Hz, ^4J = 1.5 Hz, 1H), 4.53 (d, ^3J = 9.4 Hz, 1H), 4.46 (t, ^3J = 8.0 Hz, 1H), 4.37 – 4.30 (m, 2H), 4.20 – 4.12 (m, 3H), 3.80 – 3.76 (m, 2H), 3.66 – 3.45 (m, 19H), 2.46 (s, 3H), 2.39 (t, ^3J = 6.2 Hz, 2H), 1.89 (s, 3H), 1.38 (s, 9H), 0.92 (s, 9H).
^{13}C NMR (126 MHz, DCM-d_2): δ = 172.1, 170.4, 169.7, 169.1, 155.8, 151.5, 147.7, 131.4, 130.7, 127.7, 127.3, 120.9, 111.9, 79.7, 70.1, 69.9, 69.82, 69.78, 69.70, 69.66, 69.0, 68.9, 67.9, 66.2, 58.7, 56.4, 37.9, 37.2, 35.9, 35.2, 31.3, 27.8, 26.3, 22.3, 15.9.
LCMS: R_t = 3.76 min (method A).

MS(ESI) [m/z]: calculated = 793.4 $[M+H]^+$; found = 793.5 $[M+H]^+$.

(2S,4R)-1-((S)-2-acetamido-3,3-dimethylbutanoyl)-N-(2-((15-(4-(4-((4-((2-carbamoylphenyl)amino)-5-chloropyrimidin-2-yl)amino)phenyl)piperazin-1-yl)-15-oxo-3,6,9,12-tetraoxapentadecyl)oxy)-4-(4-methylthiazol-5-yl)benzyl)-4-hydroxypyrrolidine-2-carboxamide (6-e)

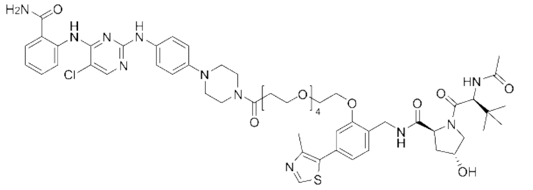

To a solution of tert-butyl 1-(2-(((2S,4R)-1-((S)-2-acetamido-3,3-dimethylbutanoyl)-4-hydroxypyrrolidine-2-carboxamido)methyl)-5-(4-methylthiazol-5-yl)phenoxy)-3,6,9,12-tetraoxapentadecan-15-oate (**47**) (16 mg, 38 µmol, 1.0eq) in 1,4-dioxane (2.0 mL) was added

a solution of HCl in dioxane (0.2 mL, 4 M, 29eq) and the resulting mixture was stirred for 16 h at r.t Then, water (0.2 mL) and additional HCl in dioxane (0.2 mL, 4M, 29eq) was added and the mixture was stirred for 5 h at 40 °C. All volatiles were removed under reduced pressure. The crude material was dissolved in ACN (1 mL), treated with TEA (0.1 mL) and all volatiles were removed under reduced pressure. The crude product was used without further purification for the next steps.

To a solution of 2-((5-chloro-2-((4-(piperazin-1-yl)phenyl)amino)pyrimidin-4-yl)amino)benzamide (**6**) (16 mg, 38 µmol, 1.0eq) in DMF (2 mL) were added the crude acid (TEA salt, 32 mg, 38 µmol, 1.0eq), 1-hydroxybenzotriazole (0.5 mg, 4 µmol, 0.1eq), 4-methylmorpholine (21 µL, 189 µmol, 5.0 eq) and EDC HCl (9 mg, 45 µmol, 1.2eq). The resulting mixture was stirred for 20 h. The solvent was evaporated and the residue was purified using flash column chromatography (DCM/MeOH) followed by reversed phase flash column chromatography (H_2O/ACN). The crude title compound was isolated as colorless wax (10 mg, 23%). Triethylamine hydrochloride was observed as an impurity.

^{1}H NMR (500 MHz, $CDCl_3$): δ = 12.10 (s, 1H), 12.06 (s, 1H), 9.13 (s, 1H), 8.64 (s, 1H), 8.51 (d, ^3J = 8.3 Hz, 1H), 7.88 (s, 1H), 7.70 (d, ^3J = 7.2 Hz, 1H), 7.43 – 7.36 (m, 3H), 7.36 – 7.29 (m, 2H), 7.14 (t, ^3J = 7.3 Hz, 1H), 6.96 – 6.88 (m, 3H), 6.84 (s, 1H), 6.66 (d, ^3J = 8.9 Hz, 1H), 6.37 (s, 1H), 4.65 (t, ^3J = 7.9 Hz, 1H), 4.55 – 4.37 (m, 4H), 4.20 – 4.08 (m, 2H), 4.01 (d, ^3J = 11.2 Hz, 1H), 3.86 (dd, ^3J = 9.9 Hz, ^3J = 5.7 Hz, 2H) 3.82 – 3.76 (m, 4H), 3.75 – 3.54 (m, 14H), 3.18 – 3.06 (m, 6H), 2.66 (t, ^3J = 6.2 Hz, 2H), 2.47 (s, 3H), 2.32 (ddd, ^2J = 12.9 Hz, ^3J = 8.1 Hz, ^3J = 4.4 Hz, 1H), 2.15 (dd, ^2J = 13.1 Hz, ^3J = 8.5 Hz, 1H), 1.96 (s, 3H), 0.91 (s, 9H).
^{13}C NMR (126 MHz, $CDCl_3$): δ = 171.58, 171.27, 171.08, 170.80, 169.96, 156.80, 156.44, 154.34, 150.46, 148.54, 139.05, 132.54, 132.28, 131.85, 129.86, 129.77, 129.08, 128.34, 127.00, 123.96, 123.77, 122.74, 122.06, 120.68, 120.45, 117.15, 112.81, 106.25, 77.16, 70.90, 70.62, 70.61, 70.59, 70.57, 70.56, 70.24, 69.77, 68.05, 67.71, 58.89, 57.81, 56.90, 41.50, 39.06, 36.80, 35.30, 33.74, 29.83, 26.55, 23.21, 16.29.
LCMS: R_t = 7.27 min (method D); purity = >99%.

MS(ESI) [m/z]: calculated = 1164.5 $[M+Na]^+$; found = 1164.3 $[M+Na]^+$.

HRMS: calculated = 1142.4895 $[M+H]^+$; found = 1142.4897 $[M+H]^+$.

References

(1) Klaeger, S.; Heinzlmeir, S.; Wilhelm, M.; Polzer, H.; Vick, B.; Koenig, P.-A.; Reinecke, M.; Ruprecht, B.; Petzoldt, S.; Meng, C.; Zecha, J.; Reiter, K.; Qiao, H.; Helm, D.; Koch, H.; Schoof, M.; Canevari, G.; Casale, E.; Depaolini, S. R.; Feuchtinger, A.; Wu, Z.; Schmidt, T.; Rueckert, L.; Becker, W.; Huenges, J.; Garz, A.-K.; Gohlke, B.-O.; Zolg, D. P.; Kayser, G.; Vooder, T.; Preissner, R.; Hahne, H.; Tõnisson, N.; Kramer, K.; Götze, K.; Bassermann, F.; Schlegl, J.; Ehrlich, H.-C.; Aiche, S.; Walch, A.; Greif, P. A.; Schneider, S.; Felder, E. R.; Ruland, J.; Médard, G.; Jeremias, I.; Spiekermann, K.; Kuster, B. The Target Landscape of Clinical Kinase Drugs. *Science* **2017**, *358* (6367), eaan4368. https://doi.org/10.1126/science.aan4368.

(2) Médard, G.; Pachl, F.; Ruprecht, B.; Klaeger, S.; Heinzlmeir, S.; Helm, D.; Qiao, H.; Ku, X.; Wilhelm, M.; Kuehne, T.; Wu, Z.; Dittmann, A.; Hopf, C.; Kramer, K.; Kuster, B. Optimized Chemical Proteomics Assay for Kinase Inhibitor Profiling. *J. Proteome Res.* **2015**, *14* (3), 1574–1586. https://doi.org/10.1021/pr5012608.

(3) Reinecke, M.; Ruprecht, B.; Poser, S.; Wiechmann, S.; Wilhelm, M.; Heinzlmeir, S.; Kuster, B.; Médard, G. Chemoproteomic Selectivity Profiling of PIKK and PI3K Kinase Inhibitors. *ACS Chem. Biol.* **2019**, *14* (4), 655–664. https://doi.org/10.1021/acschembio.8b01020.

(4) Hahne, H.; Pachl, F.; Ruprecht, B.; Maier, S. K.; Klaeger, S.; Helm, D.; Médard, G.; Wilm, M.; Lemeer, S.; Kuster, B. DMSO Enhances Electrospray Response, Boosting Sensitivity of Proteomic Experiments. *Nat Methods* **2013**, *10* (10), 989–991. https://doi.org/10.1038/nmeth.2610.

(5) Cox, J.; Mann, M. MaxQuant Enables High Peptide Identification Rates, Individualized p.p.b.-Range Mass Accuracies and Proteome-Wide Protein Quantification. *Nat Biotechnol* **2008**, *26* (12), 1367–1372. https://doi.org/10.1038/nbt.1511.

(6) Cox, J.; Neuhauser, N.; Michalski, A.; Scheltema, R. A.; Olsen, J. V.; Mann, M. Andromeda: A Peptide Search Engine Integrated into the MaxQuant Environment. *J. Proteome Res.* **2011**, *10* (4), 1794–1805. https://doi.org/10.1021/pr101065j.

(7) Cox, J.; Hein, M. Y.; Luber, C. A.; Paron, I.; Nagaraj, N.; Mann, M. Accurate Proteome-Wide Label-Free Quantification by Delayed Normalization and Maximal Peptide Ratio Extraction, Termed MaxLFQ. *Molecular & Cellular Proteomics* **2014**, *13* (9), 2513–2526. https://doi.org/10.1074/mcp.M113.031591.

(8) Hughes, C. S.; Moggridge, S.; Müller, T.; Sorensen, P. H.; Morin, G. B.; Krijgsveld, J. Single-Pot, Solid-Phase-Enhanced Sample Preparation for Proteomics Experiments. *Nat Protoc* **2019**, *14* (1), 68–85. https://doi.org/10.1038/s41596-018-0082-x.

(9) Bian, Y.; Zheng, R.; Bayer, F. P.; Wong, C.; Chang, Y.-C.; Meng, C.; Zolg, D. P.; Reinecke, M.; Zecha, J.; Wiechmann, S.; Heinzlmeir, S.; Scherr, J.; Hemmer, B.; Baynham, M.; Gingras, A.-C.; Boychenko, O.; Kuster, B. Robust, Reproducible and Quantitative Analysis of Thousands of Proteomes by Micro-Flow LC–MS/MS. *Nat Commun* **2020**, *11* (1), 157. https://doi.org/10.1038/s41467-019-13973-x.

(10) Bian, Y.; Bayer, F. P.; Chang, Y.-C.; Meng, C.; Hoefer, S.; Deng, N.; Zheng, R.; Boychenko, O.; Kuster, B. Robust Microflow LC-MS/MS for Proteome Analysis: 38 000 Runs and Counting. *Anal. Chem.* **2021**, *93* (8), 3686–3690. https://doi.org/10.1021/acs.analchem.1c00257.

(11) McAlister, G. C.; Nusinow, D. P.; Jedrychowski, M. P.; Wühr, M.; Huttlin, E. L.; Erickson, B. K.; Rad, R.; Haas, W.; Gygi, S. P. MultiNotch MS3 Enables Accurate, Sensitive, and Multiplexed Detection of Differential Expression across Cancer Cell Line Proteomes. *Anal. Chem.* **2014**, *86* (14), 7150–7158. https://doi.org/10.1021/ac502040v.

(12) Elias, J. E.; Gygi, S. P. Target-Decoy Search Strategy for Increased Confidence in Large-Scale Protein Identifications by Mass Spectrometry. *Nat Methods* **2007**, *4* (3), 207–214. https://doi.org/10.1038/nmeth1019.

(13) Ritchie, M. E.; Phipson, B.; Wu, D.; Hu, Y.; Law, C. W.; Shi, W.; Smyth, G. K. Limma Powers Differential Expression Analyses for RNA-Sequencing and Microarray Studies. *Nucleic Acids Research* **2015**, *43* (7), e47–e47. https://doi.org/10.1093/nar/gkv007.

(14) Adhikari, B.; Bozilovic, J.; Diebold, M.; Schwarz, J. D.; Hofstetter, J.; Schröder, M.; Wanior, M.; Narain, A.; Vogt, M.; Dudvarski Stankovic, N.; Baluapuri, A.; Schönemann, L.; Eing, L.; Bhandare, P.; Kuster, B.; Schlosser, A.; Heinzlmeir, S.; Sotriffer, C.; Knapp, S.; Wolf, E. PROTAC-Mediated Degradation Reveals a Non-Catalytic Function of AURORA-A Kinase. *Nat Chem Biol* **2020**, *16* (11), 1179–1188. https://doi.org/10.1038/s41589-020-00652-y.

(15) Amrhein, J. A.; Berger, L. M.; Tjaden, A.; Krämer, A.; Elson, L.; Tolvanen, T.; Martinez-Molina, D.; Kaiser, A.; Schubert-Zsilavecz, M.; Müller, S.; Knapp, S.; Hanke, T. Discovery of 3-Amino-1H-Pyrazole-Based Kinase Inhibitors to Illuminate the Understudied PCTAIRE Family. *IJMS* **2022**, *23* (23), 14834. https://doi.org/10.3390/ijms232314834.

(16) Krämer, A.; Kurz, C. G.; Berger, B.-T.; Celik, I. E.; Tjaden, A.; Greco, F. A.; Knapp, S.; Hanke, T. Optimization of Pyrazolo[1,5-a]Pyrimidines Lead to the Identification of a Highly Selective Casein Kinase 2 Inhibitor. *European Journal of Medicinal Chemistry* **2020**, *208*, 112770. https://doi.org/10.1016/j.ejmech.2020.112770.

(17) Amrhein, J. A.; Wang, G.; Berger, B.-T.; Berger, L. M.; Kalampaliki, A. D.; Krämer, A.; Knapp, S.; Hanke, T. Design and Synthesis of Pyrazole-Based Macrocyclic Kinase Inhibitors Targeting BMPR2. *ACS Med. Chem. Lett.* **2023**, *14* (6), 833–840. https://doi.org/10.1021/acsmedchemlett.3c00127.

(18) Karlsson, M.; Zhang, C.; Méar, L.; Zhong, W.; Digre, A.; Katona, B.; Sjöstedt, E.; Butler, L.; Odeberg, J.; Dusart, P.; Edfors, F.; Oksvold, P.; Von Feilitzen, K.; Zwahlen, M.; Arif, M.; Altay, O.; Li, X.; Ozcan, M.; Mardinoglu, A.; Fagerberg, L.; Mulder, J.; Luo, Y.; Ponten, F.; Uhlén, M.; Lindskog, C. A Single–Cell Type Transcriptomics Map of Human Tissues. *Sci. Adv.* **2021**, *7* (31), eabh2169. https://doi.org/10.1126/sciadv.abh2169.

Compound Characterization Data

I. Kinase Parent Inhibitors and Linker Conjugates

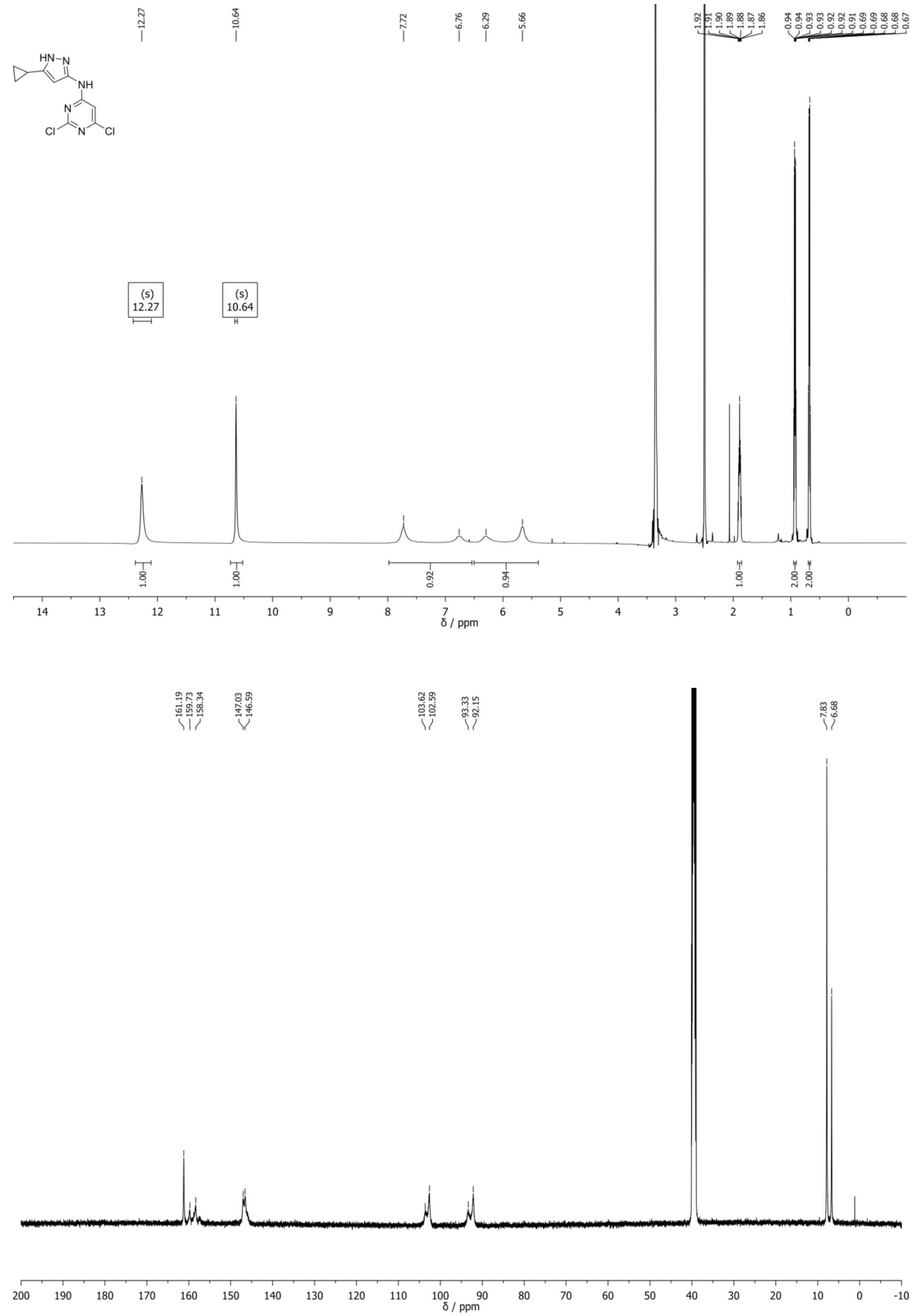

Figure S22: ^{1}H- (top) and ^{13}C-NMR (bottom) spectra (500 MHz and 126 MHz, 298 K, DMSO-d_6) and chemical structure of compound **9**.

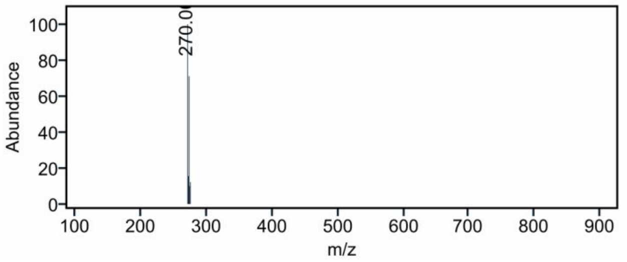

Figure S23: ESI-MS spectrum of compound **9** with $[M+H]^+_{calc.}$ = 270.03 m/z.

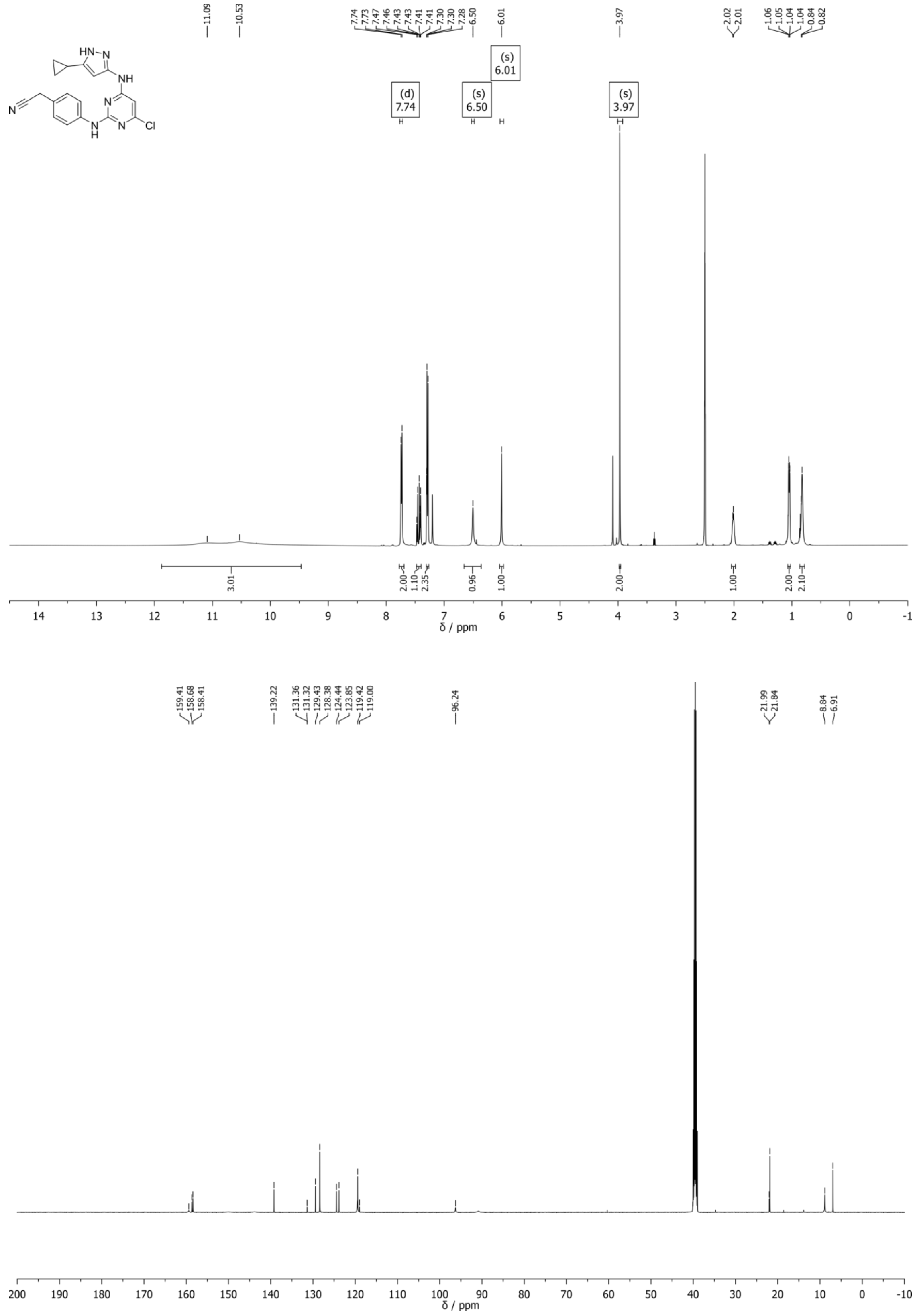

Figure S24: ^{1}H- (top) and ^{13}C-NMR (bottom) spectra (500 MHz and 126 MHz, 298 K, DMSO-d_6) and chemical structure of compound **11**.

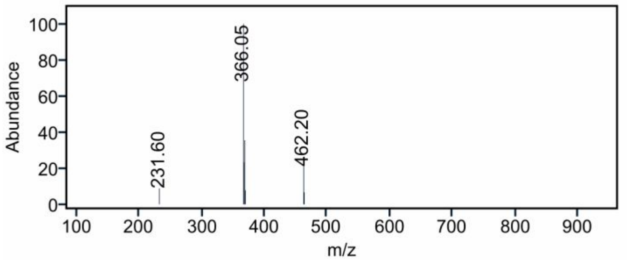

Figure S25: ESI-MS spectrum of compound **11** with $[M+H]^+_{calc.}$ = 366.12 m/z.

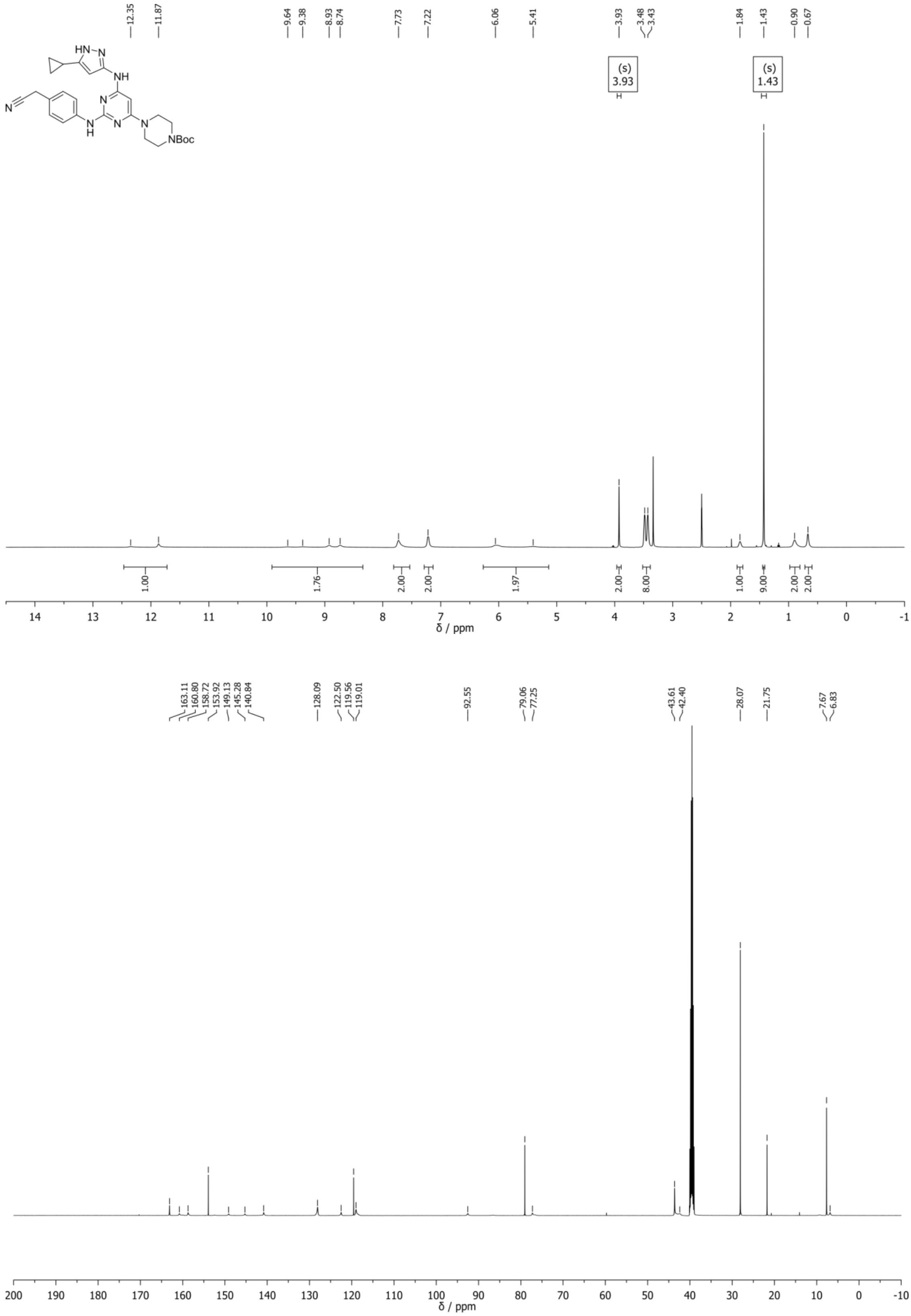

Figure S26: ^{1}H- (top) and ^{13}C-NMR (bottom) spectra (500 MHz and 126 MHz, 298 K, DMSO-d_6) and chemical structure of compound **4.**

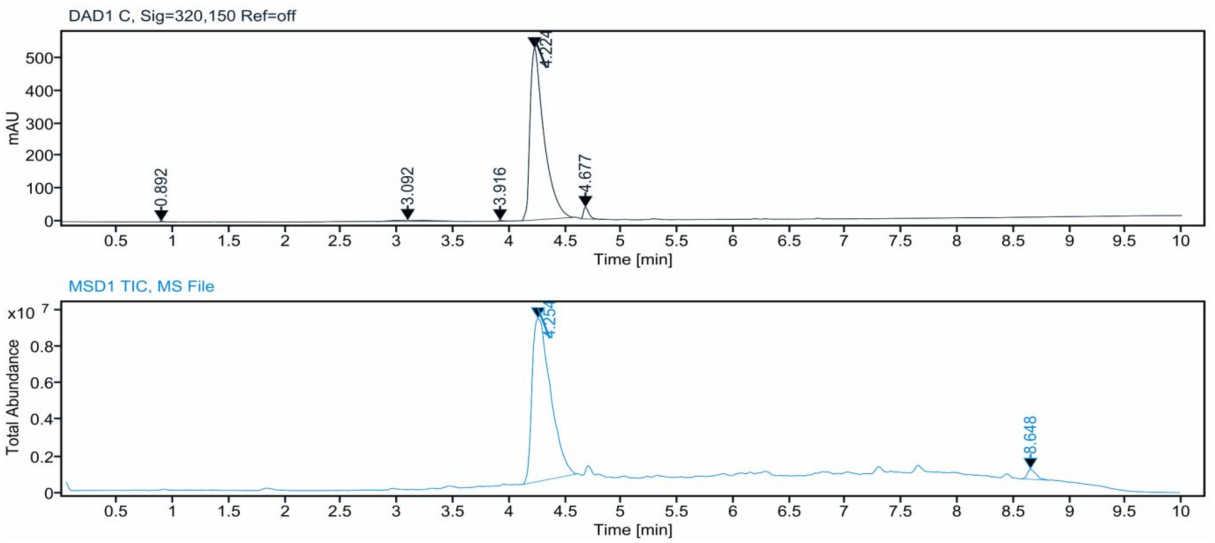

Sample Purity

Signal Description DAD1 C, Sig=320,150 Ref=off

Sample Name	Name	RT	Width	Area	Area%	Height
NM213_M		0.892	0.048	5.0336	0.11	1.4908
NM213_M		3.092	0.398	86.6474	1.81	3.5878
NM213_M		3.916	0.050	7.0121	0.15	2.3487
NM213_M		4.224	0.128	4563.5039	95.45	525.7070
NM213_M		4.677	0.050	118.7272	2.48	35.8593

Max Area% 95.452

UV Signal Purity>95% Pass

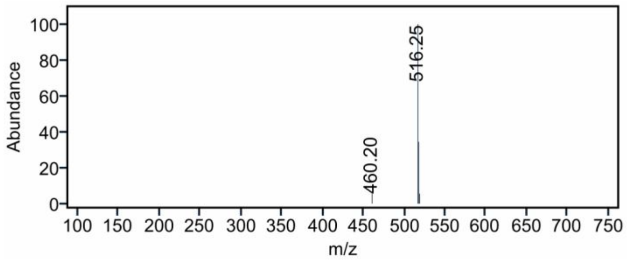

Figure S27: LC/MS spectra of purified compound **4** at 320 nm wavelength with $[M+H]^+_{calc.}$ = 516.28 m/z.

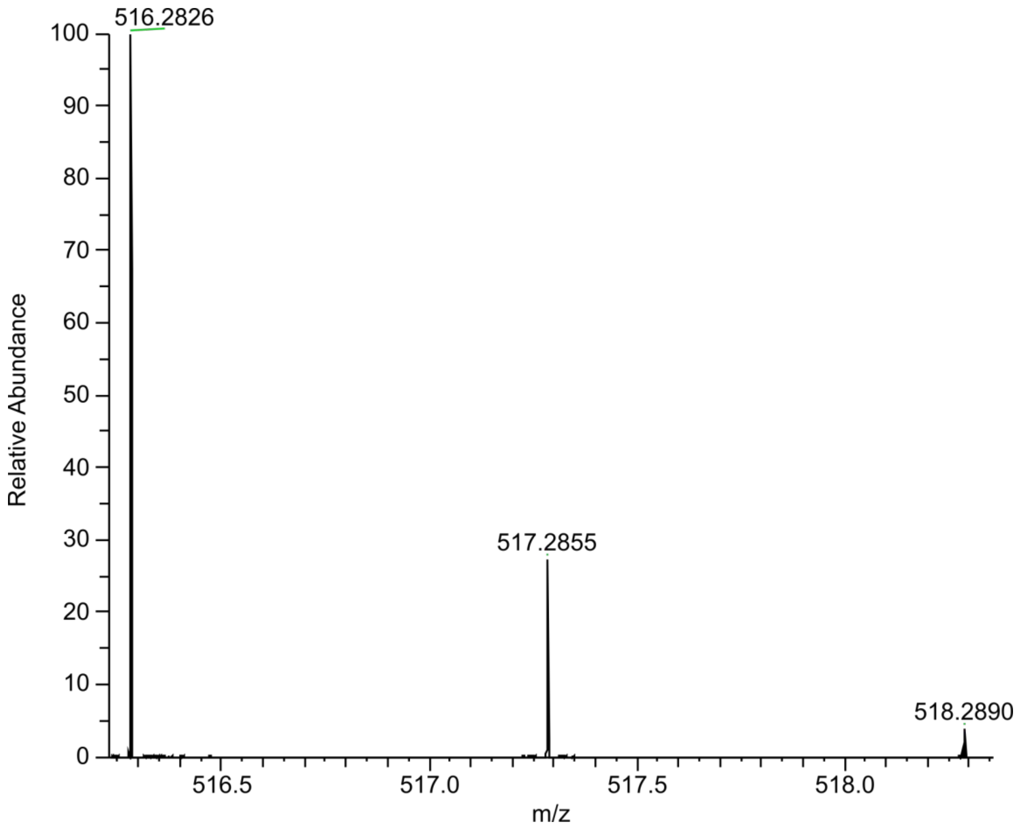

Figure S28: High-resolution mass spectrum of compound **4** with $[M+H]^{+}_{calc.}$ = 516.2830 m/z.

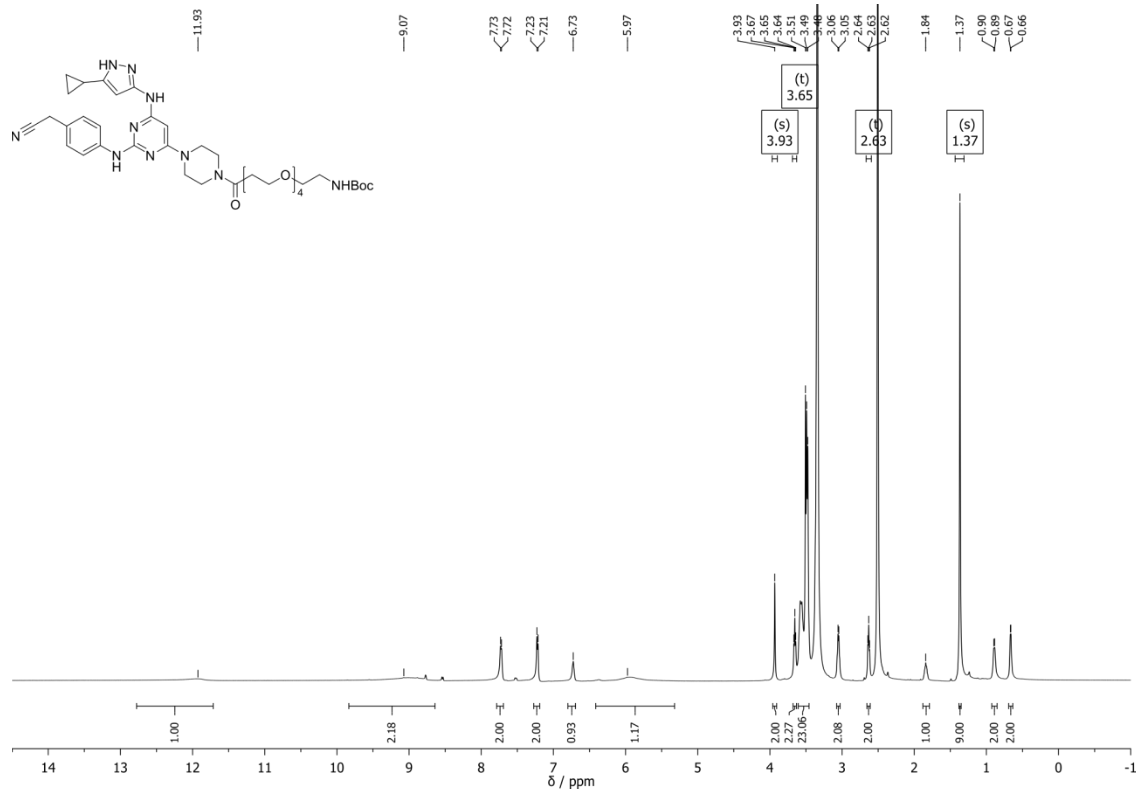

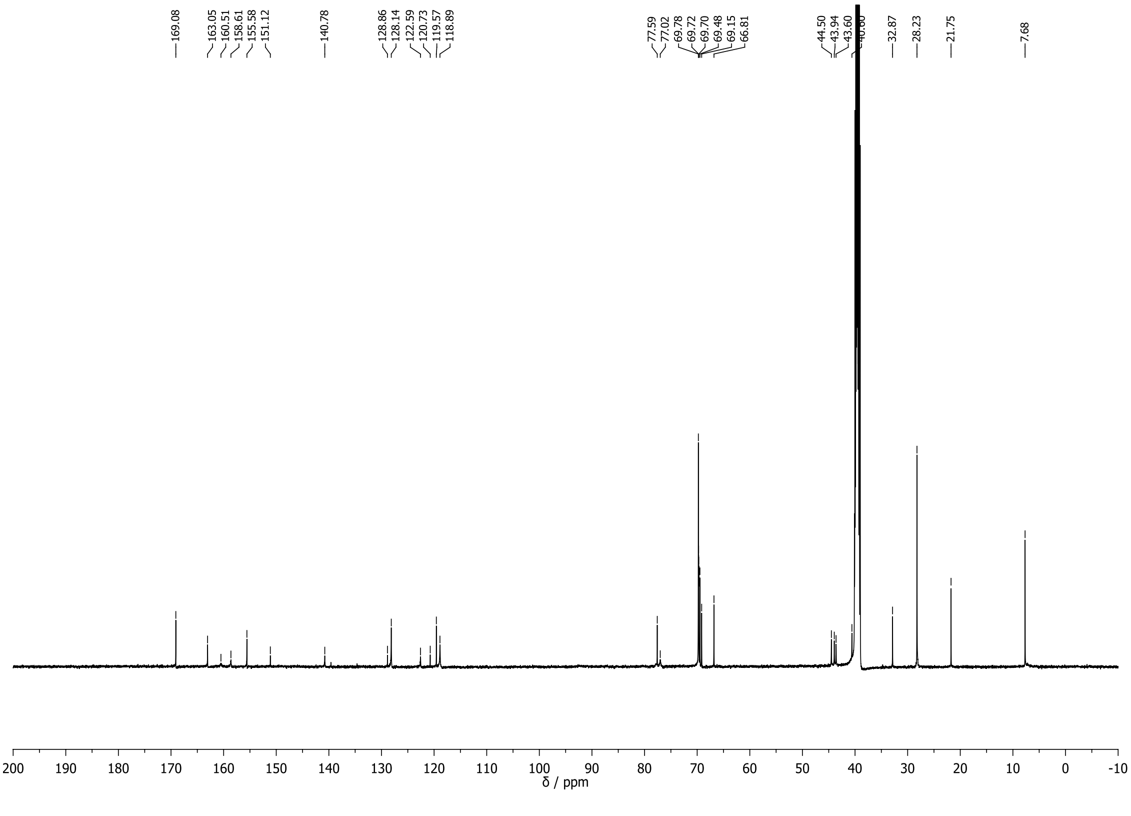

Figure S29: ^{1}H- (top) and ^{13}C-NMR (bottom) spectra (500 MHz and 126 MHz, 298 K, DMSO-d_6) and chemical structure of compound **4-L**.

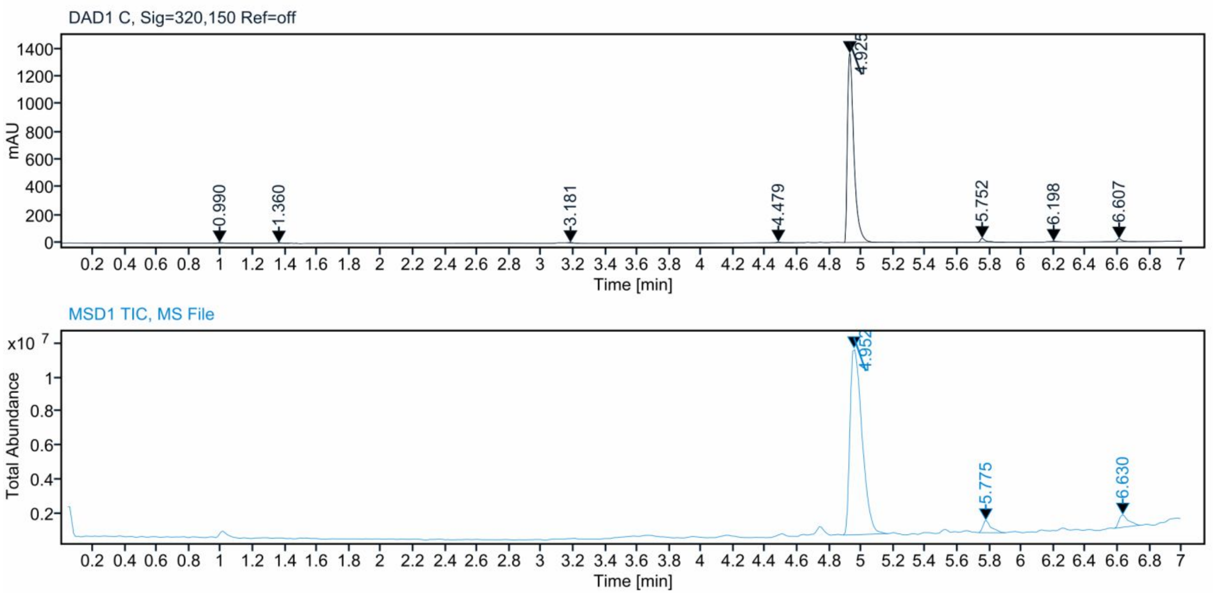

Sample Purity

Signal Description DAD1 C, Sig=320,150 Ref=off

Sample Name	Name	RT	Width	Area	Area%	Height
NM80_check_conc		0.990	0.040	13.1849	0.33	4.0775
NM80_check_conc		1.360	0.033	8.8236	0.22	3.5697
NM80_check_conc		3.181	0.029	8.4404	0.21	4.3849
NM80_check_conc		4.479	0.024	10.7938	0.27	6.6658
NM80_check_conc		4.925	0.041	3804.2854	95.94	1363.3745
NM80_check_conc		5.752	0.029	62.9401	1.59	28.1725
NM80_check_conc		6.198	0.029	8.9570	0.23	4.5831
NM80_check_conc		6.607	0.031	47.7782	1.20	21.2868

Max Area% 95.942

UV Signal Purity>95% Pass

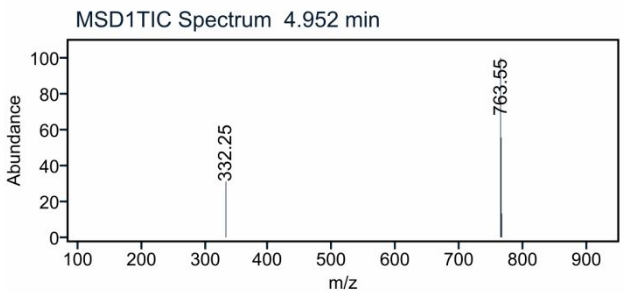

Figure S30: LC/MS spectra of purified compound **4-L** at 320 nm with $[M+H]^+_{calc.}$ = 762.42 m/z.

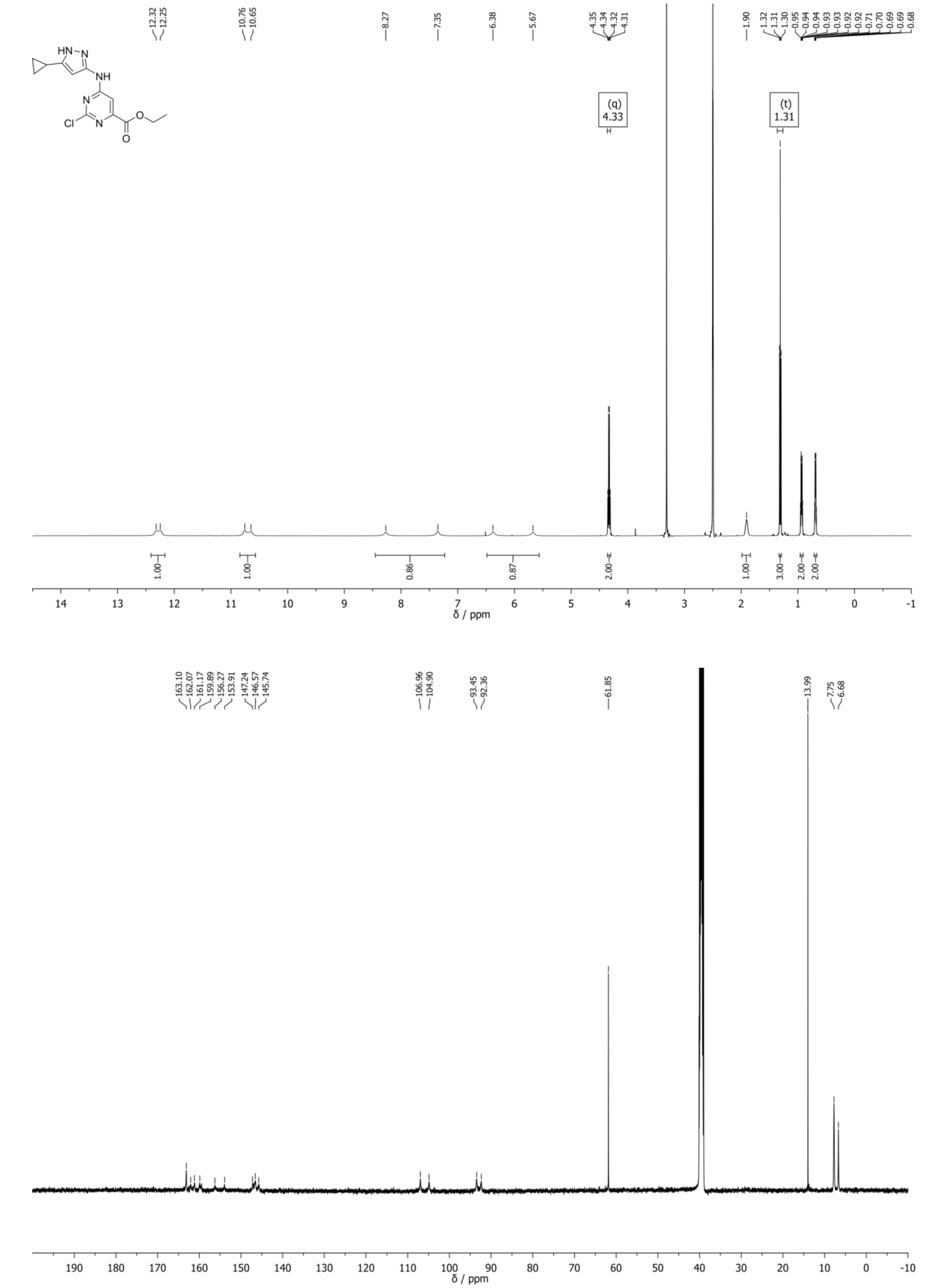

Figure S31: ^{1}H- (top) and ^{13}C-NMR (bottom) spectra (500 MHz and 126 MHz, 298 K, DMSO-d_6) and chemical structure of compound **17**.

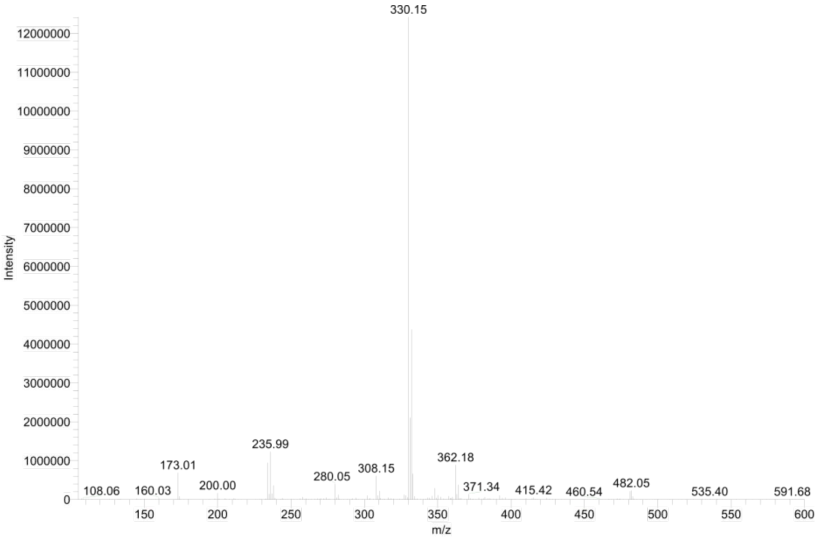

Figure S32: ESI-TLC-MS spectrum of compound **17** with $[M+Na]^{+}_{calc.}$ = 330.07 m/z.

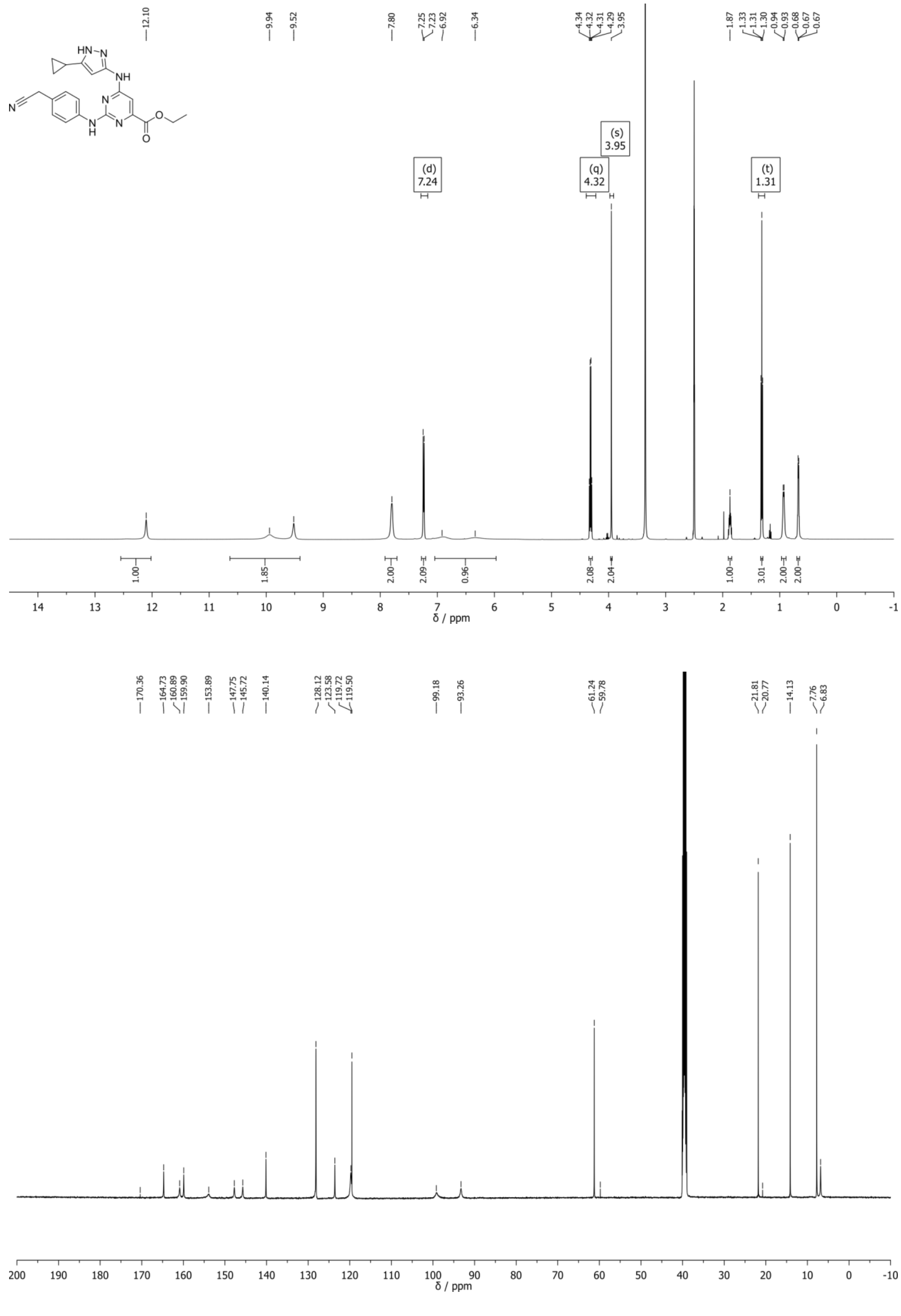

Figure S33: ^{1}H- (top) and ^{13}C-NMR (bottom) spectra (500 MHz and 126 MHz, 298 K, DMSO-d_6) and chemical structure of compound **5**.

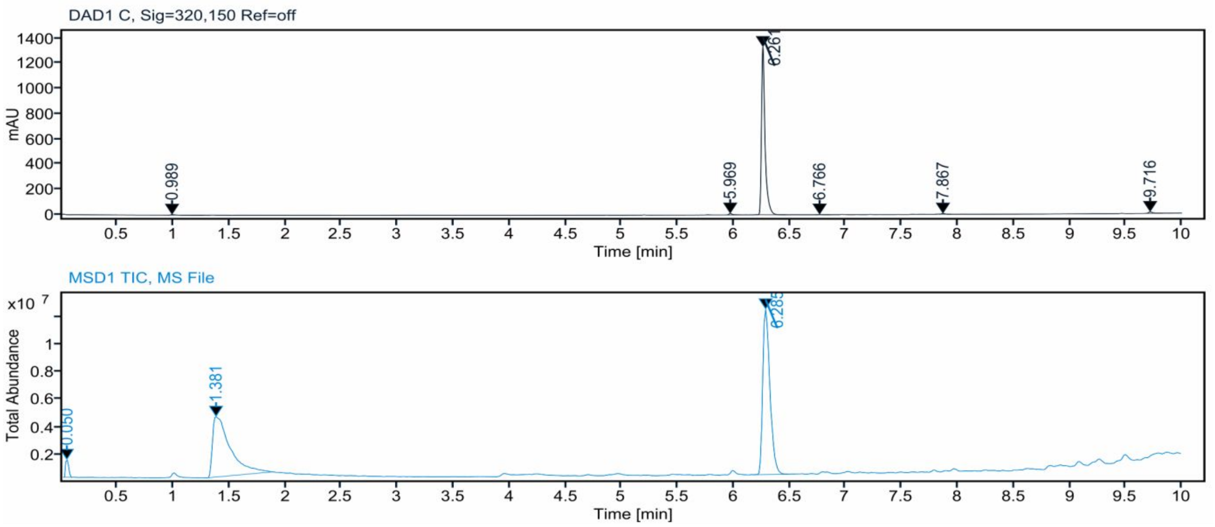

Sample Purity

Signal Description DAD1 C, Sig=320,150 Ref=off

Sample Name	Name	RT	Width	Area	Area%	Height
NM59_check		0.989	0.038	11.3997	0.38	3.9222
NM59_check		5.969	0.029	24.8659	0.82	11.8327
NM59_check		6.261	0.030	2931.5378	97.18	1330.7179
NM59_check		6.766	0.033	5.7674	0.19	2.3229
NM59_check		7.867	0.035	12.4042	0.41	5.2728
NM59_check		9.716	0.037	30.7663	1.02	11.7819

Max Area% 97.176

UV Signal Purity>95% Pass

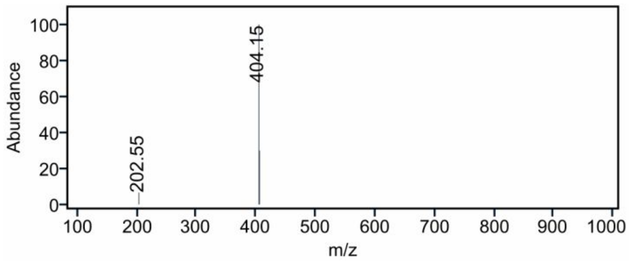

Figure S34: LC/MS spectra of purified compound **5** at 320 nm wavelength with $[M+H]^+_{calc.}$ = 404.18 m/z.

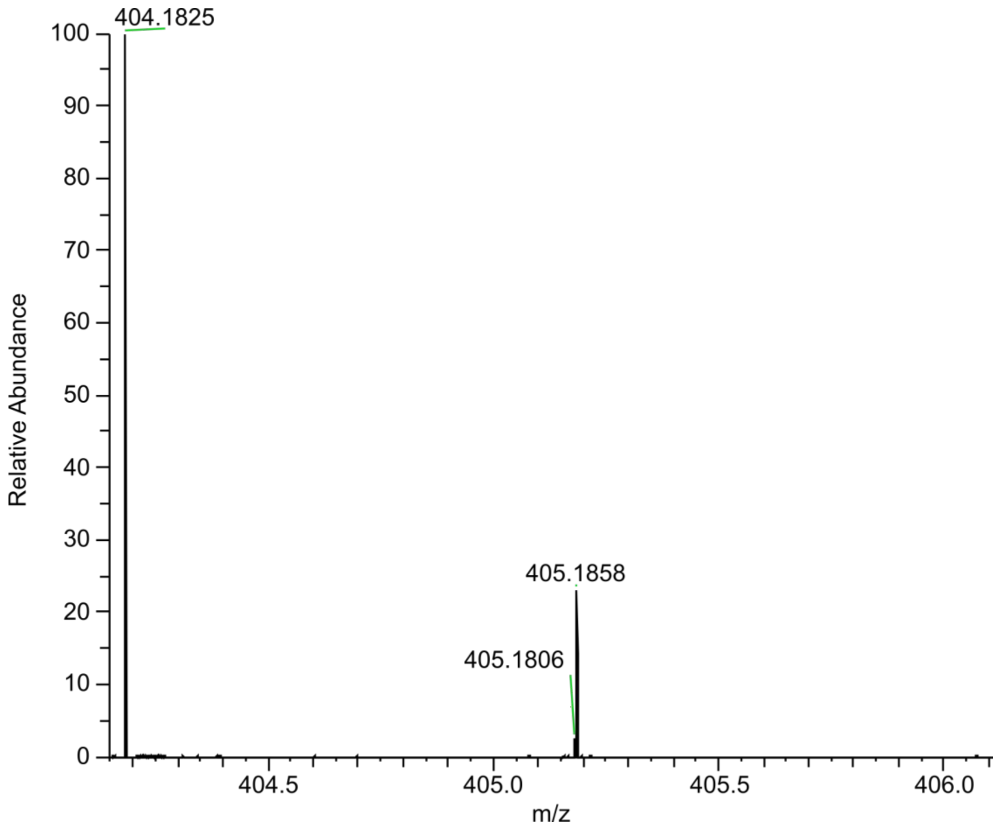

Figure S35: High-resolution mass spectrum of compound **5** with $[M+H]^{+}_{calc.}$ = 404.1829 m/z.

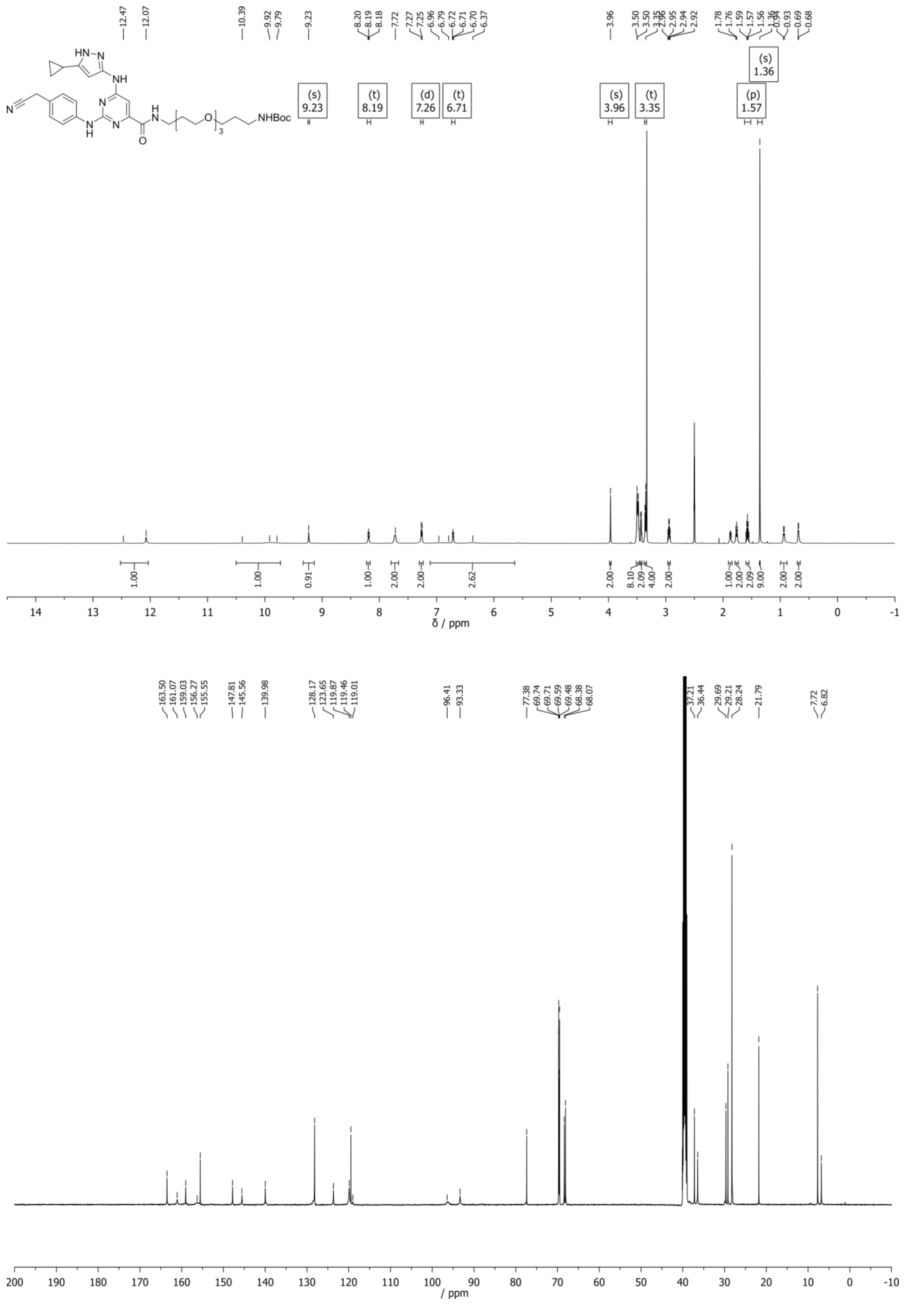

Figure S36: ^{1}H- (top) and ^{13}C-NMR (bottom) spectra (500 MHz and 126 MHz, 298 K, DMSO-d_6) and chemical structure of compound **5-L**.

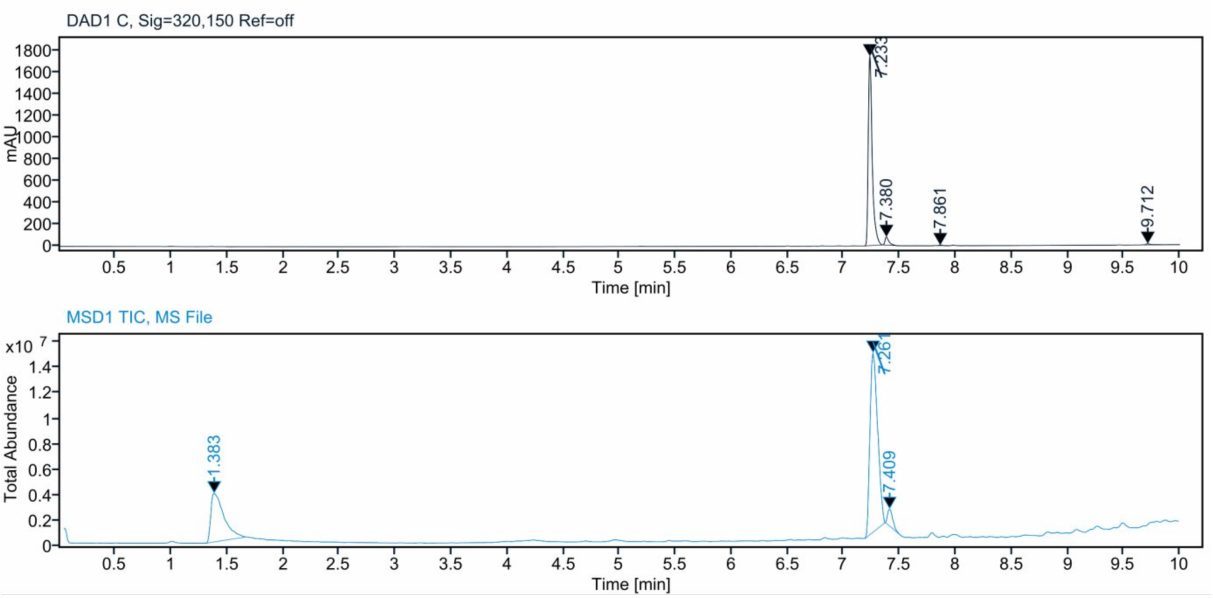

Sample Purity

Signal Description	DAD1 C, Sig=320,150 Ref=off

Sample Name	Name	RT	Width	Area	Area%	Height
NM68_check		7.233	0.035	4280.0996	95.07	1746.6417
NM68_check		7.380	0.034	183.3193	4.07	75.9752
NM68_check		7.861	0.035	10.7809	0.24	4.6785
NM68_check		9.712	0.037	27.7475	0.62	10.4604

Max Area%	95.072
UV Signal Purity>95%	Pass

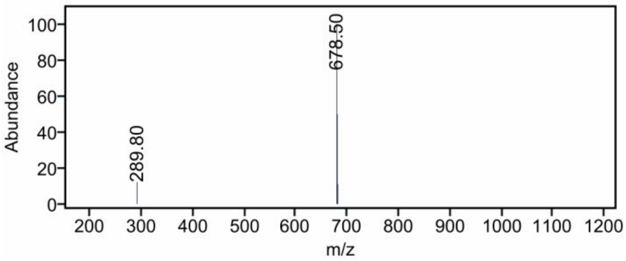

Figure S37: LC/MS spectra of purified compound **5-L** at 320 nm wavelength with $[M+H]^+_{calc.}$ = 678.37 m/z.

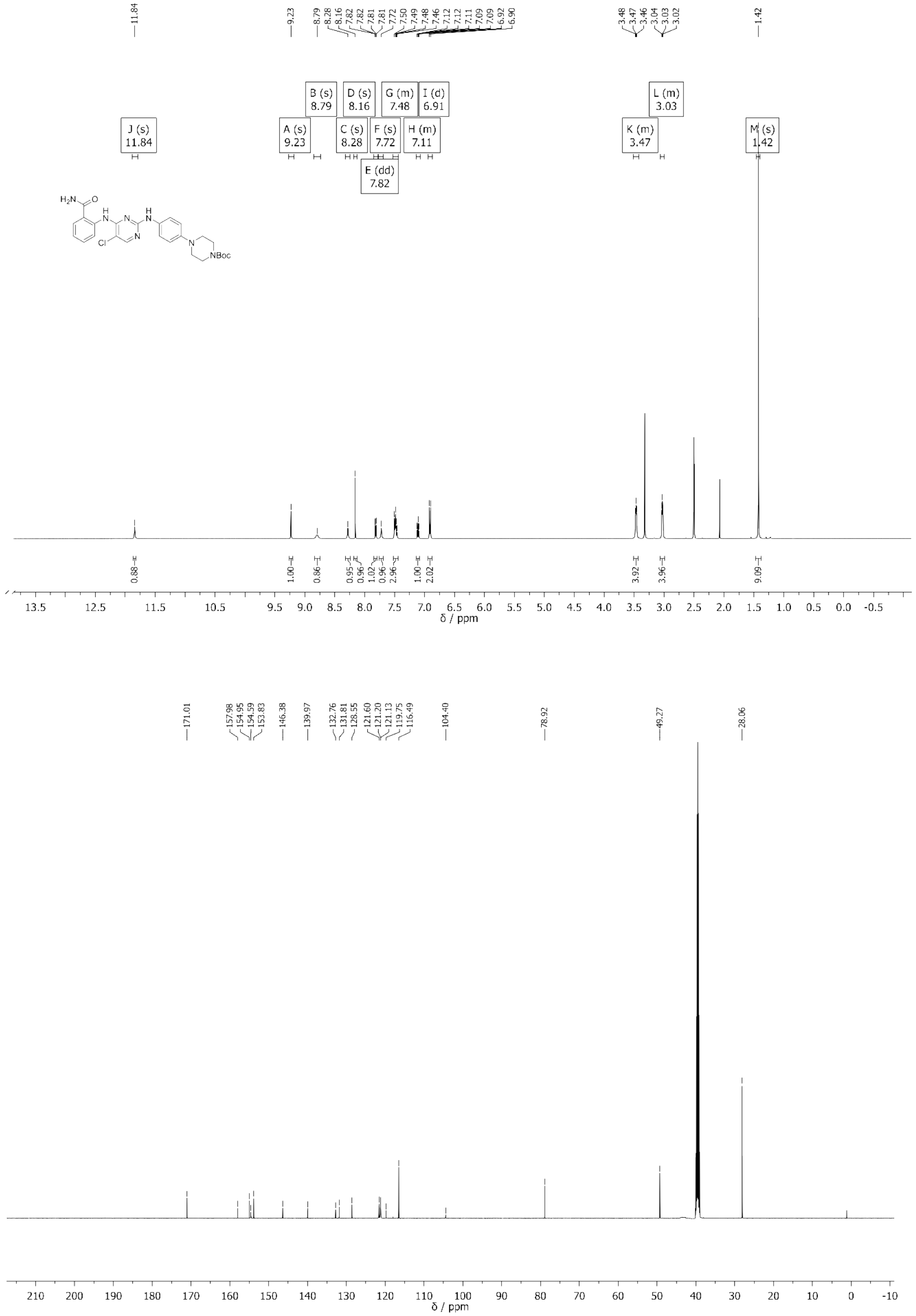

Figure S38: ^{1}H- (top) and 13C-NMR (bottom) spectra (500 MHz and 126 MHz, 298 K, DMSO-d_6) and chemical structure of compound **25.**

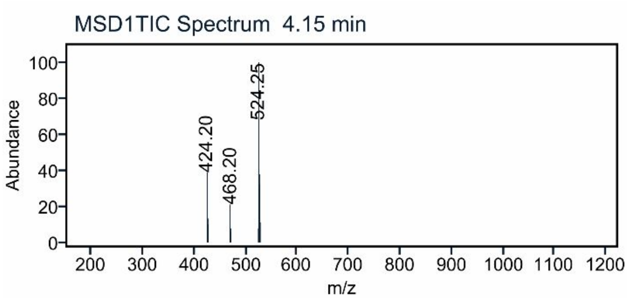

Figure S39: LC/MS spectra of purified compound **25** at 320 nm with $[M+Na+ACN]^{+}_{calc.}$ = 524.2 m/z.

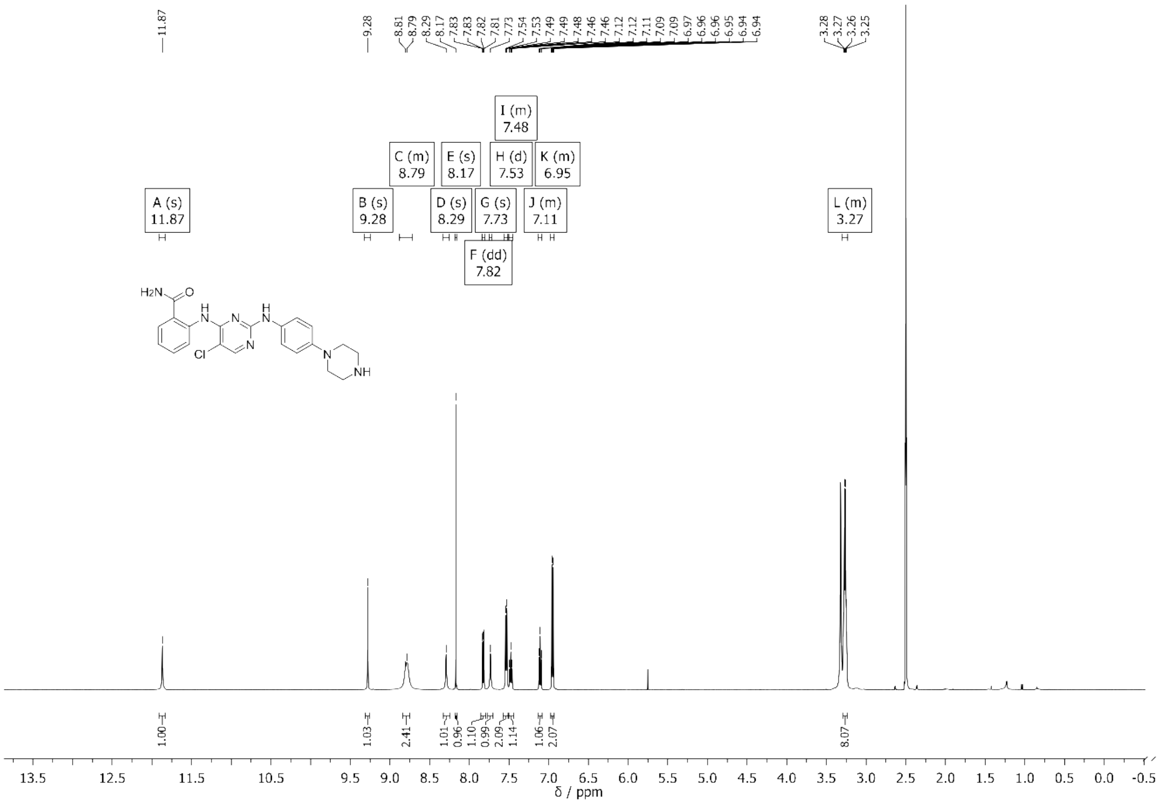

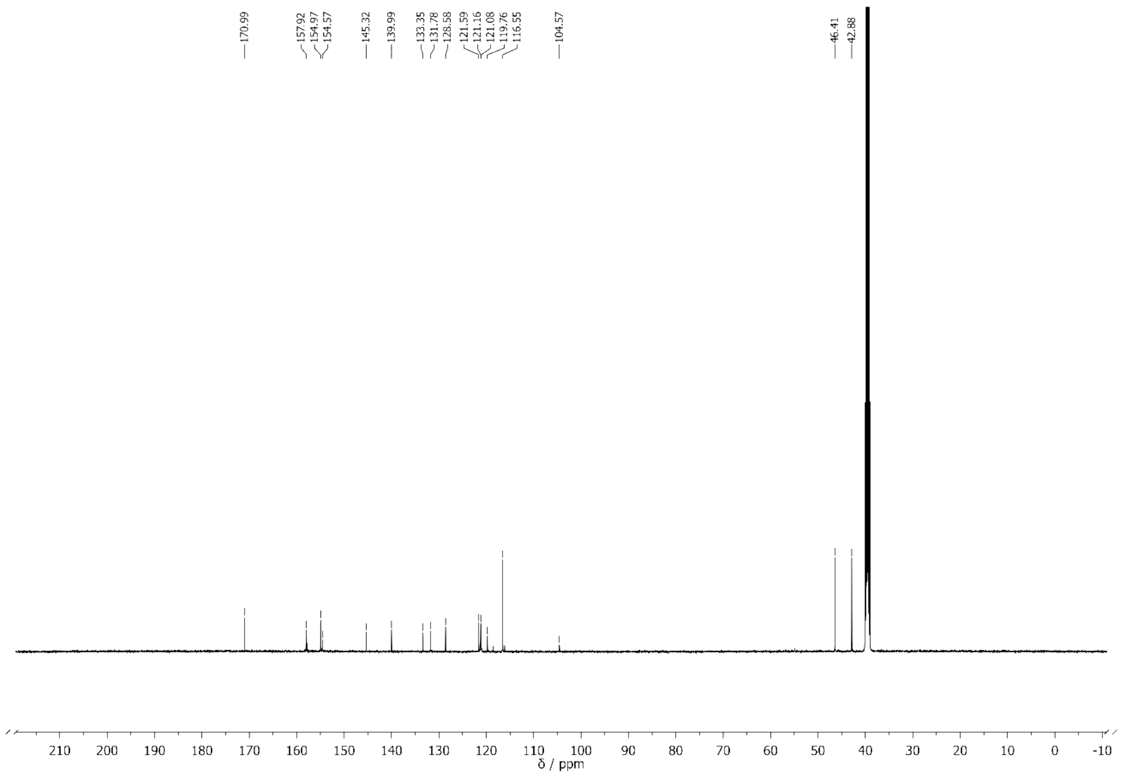

Figure S40: ^{1}H- (top) and 13C-NMR (bottom) spectra (500 MHz and 126 MHz, 298 K, DMSO-d_6) and chemical structure of compound **6**.

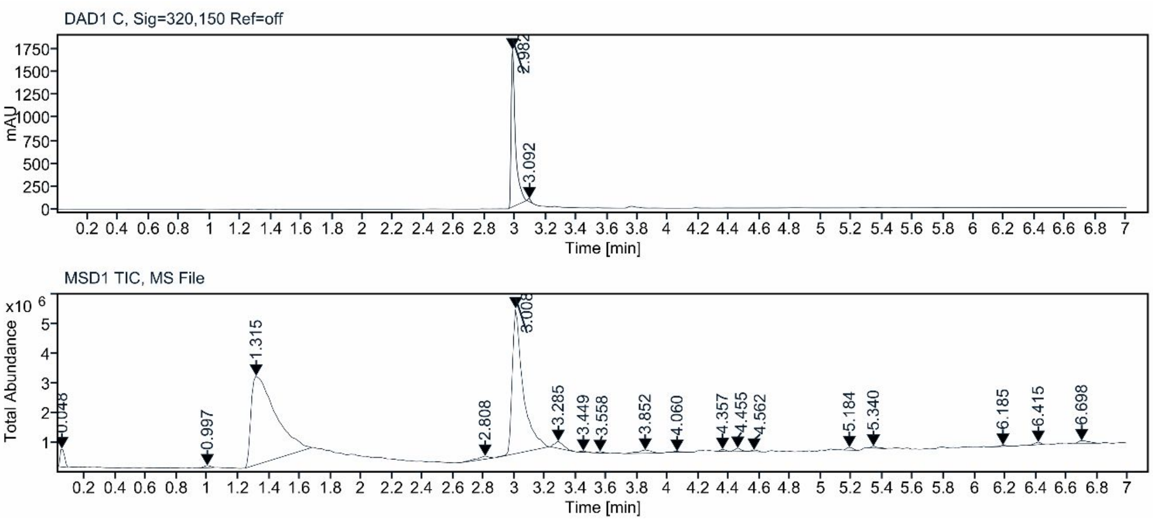

Signal Description DAD1 C, Sig=320,150 Ref=off

Sample Name	Name	RT	Width	Area	Area%	Height
jw-462		2.982	0.026	3378.9204	98.71	1730.0533
jw-462		3.092	0.020	44.1442	1.29	34.9334

Max Area% 98.710

UV Signal Purity>95% Pass

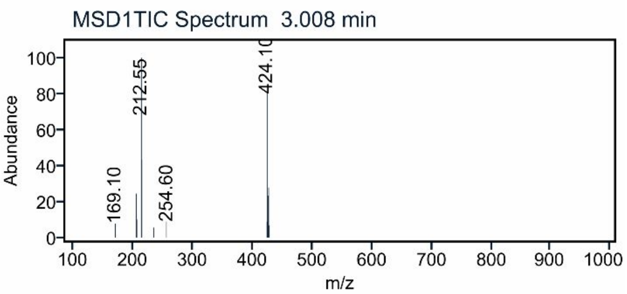

Figure S41: LC/MS spectra of purified compound **6** at 320 nm wavelength with $[M+H]^{+}_{calc.}$ = 424.2 m/z.

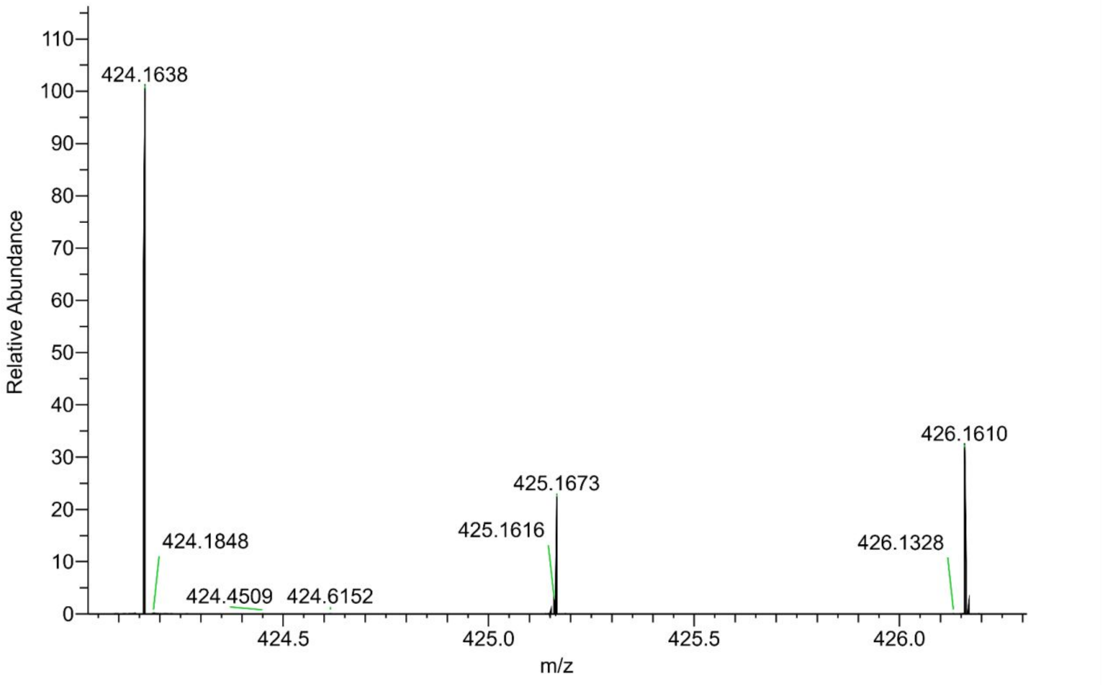

Figure 42: High-resolution mass spectrum of compound **6** with $[M+H]^+_{calc.}$ = 424.1647 m/z.

II. Promiscuous Kinase PROTACs

II.a Promiscuous Kinase PROTACs based on Kinase Parent Inhibitor 4

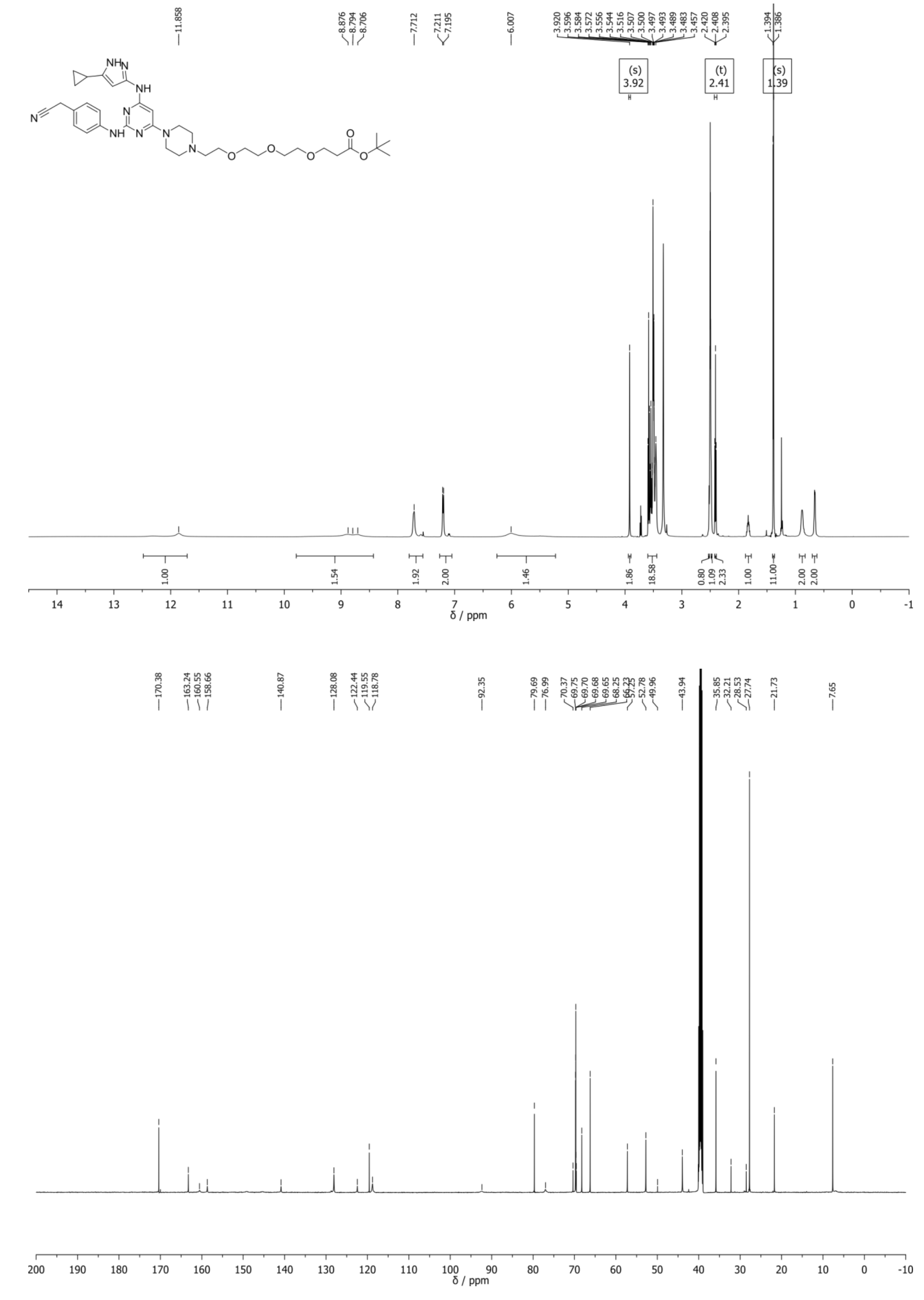

Figure S43: ^{1}H- (top) and ^{13}C-NMR (bottom) spectra (500 MHz and 126 MHz, 298 K, DMSO-d_6) and chemical structure of compound **29**

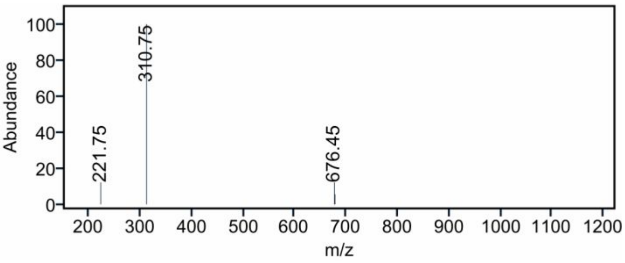

Figure S44: ESI-MS spectrum of compound **29** with $[M+H]^{+}_{calc.}$ = 676.38 m/z.

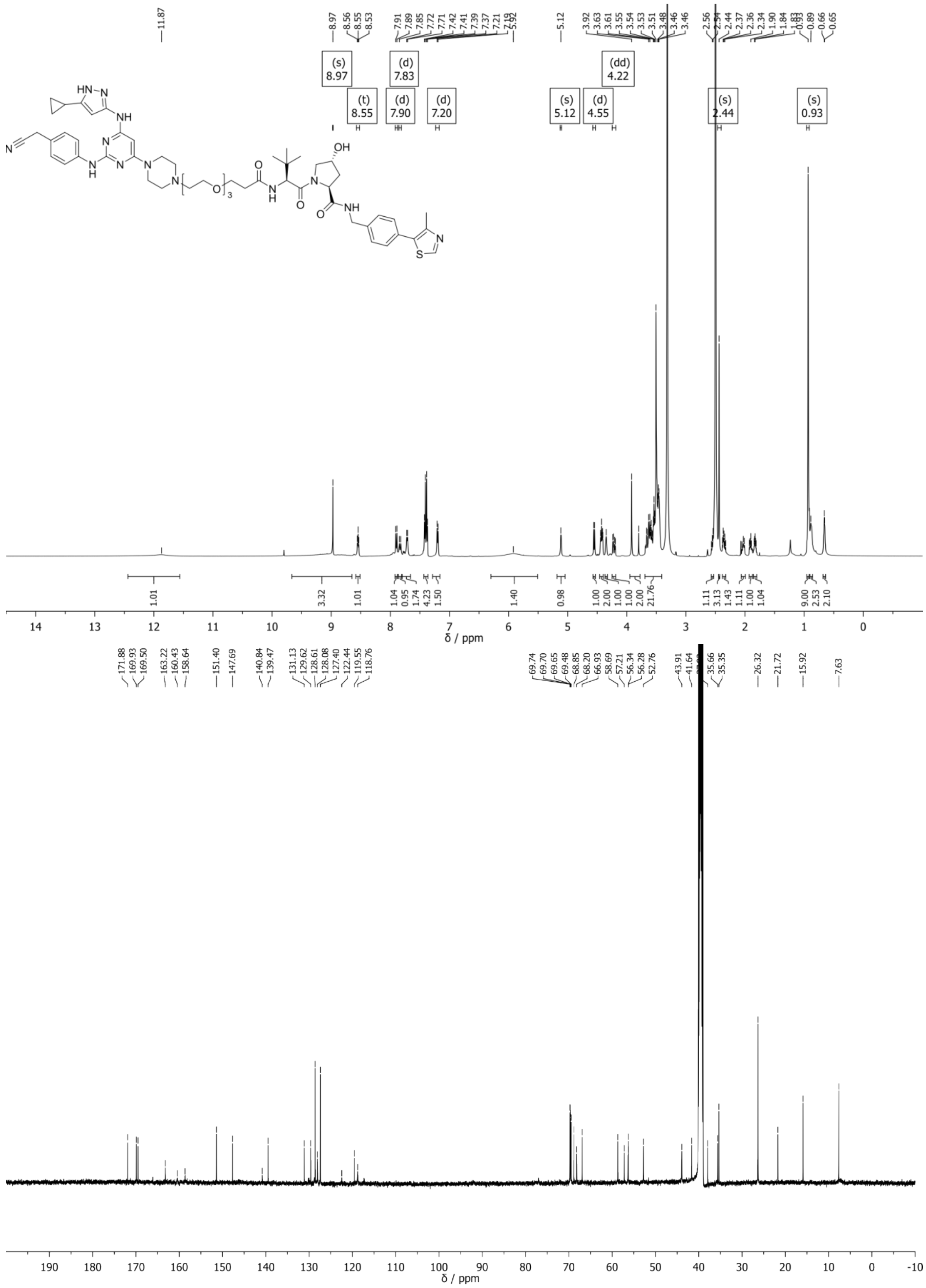

Figure S45: ^{1}H- (top) and ^{13}C-NMR (bottom) spectra (500 MHz and 126 MHz, 298 K, DMSO-d_6) and chemical structure of compound **4-a**.

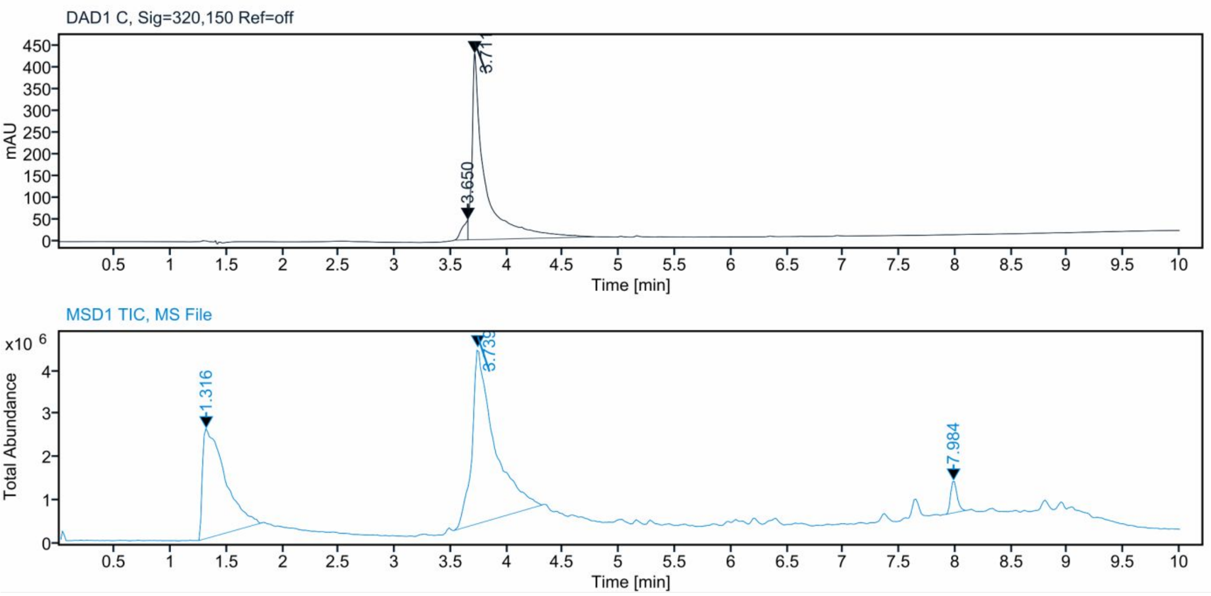

Sample Purity

Signal Description DAD1 C, Sig=320,150 Ref=off

Sample Name	Name	RT	Width	Area	Area%	Height
NM288desalt		3.650	0.058	159.0585	4.40	45.9444
NM288desalt		3.711	0.073	3458.4221	95.60	429.3995

Max Area% 95.603

UV Signal Purity>95% Pass

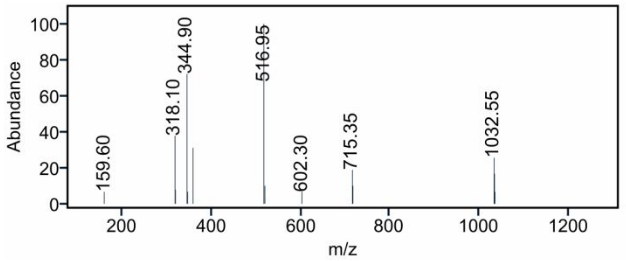

Figure S 46: LC/MS spectra of purified compound **4-a** at 320 nm with $[M+H]^{+}_{calc.}$ = 1032.52 m/z.

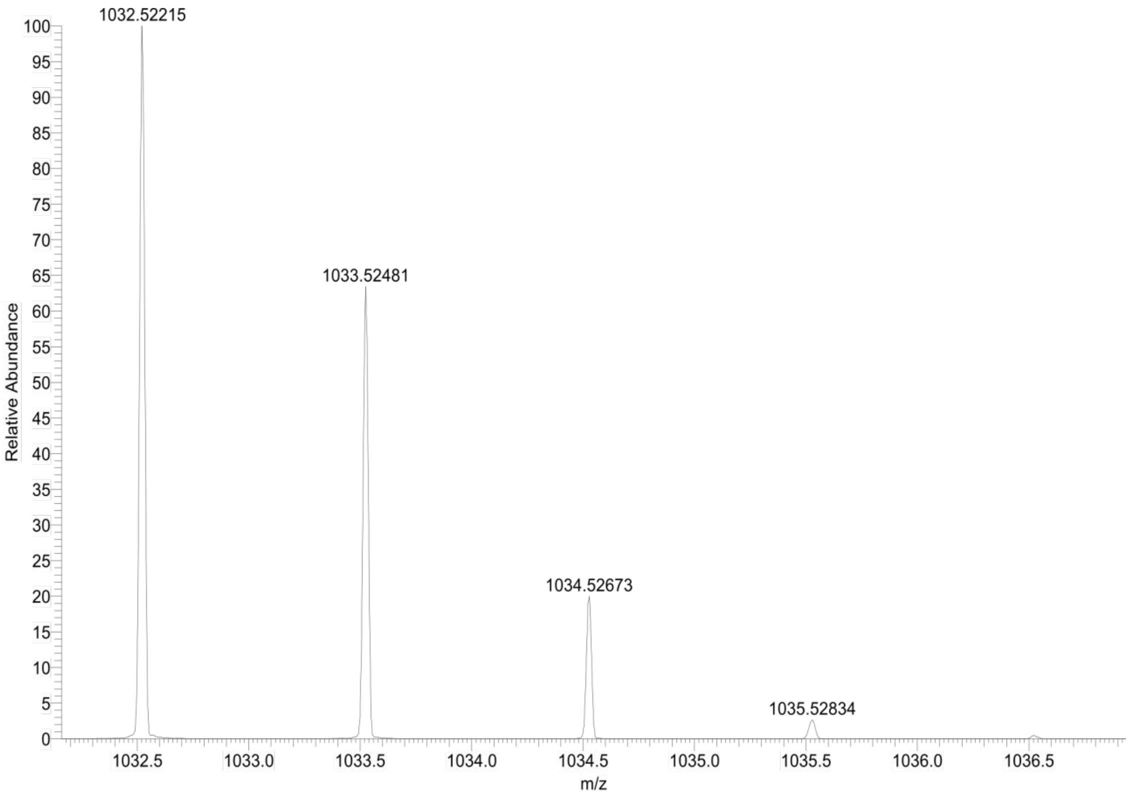

Figure S47: High-resolution mass spectrum of compound **4-a** with $[M+H]^+_{calc.}$ = 1032.52364 m/z.

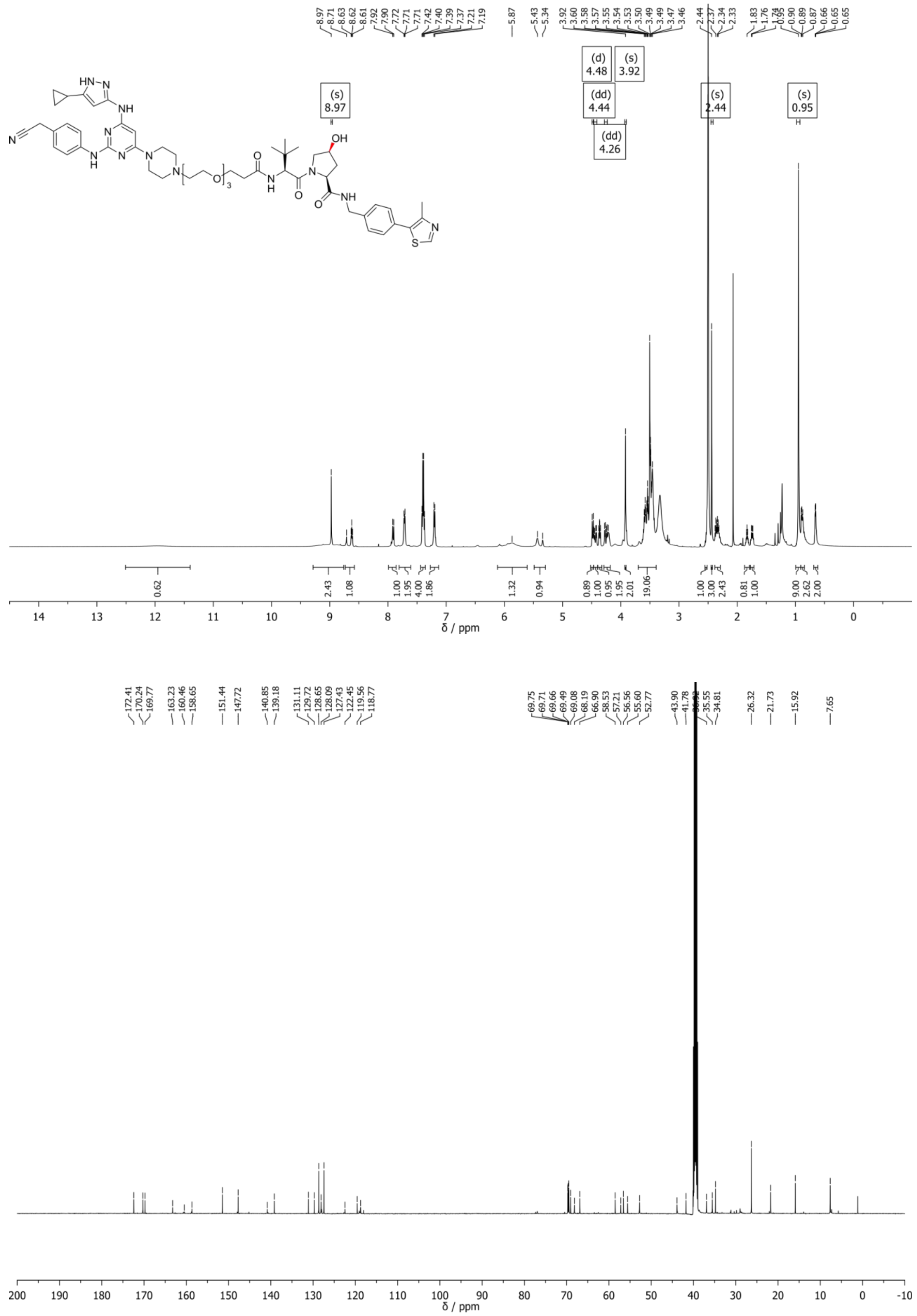

Figure S48: ^{1}H- (top) and ^{13}C-NMR (bottom) spectra (500 MHz and 126 MHz, 298 K, DMSO-d_6) and chemical structure of compound **4-a^{neg}**.

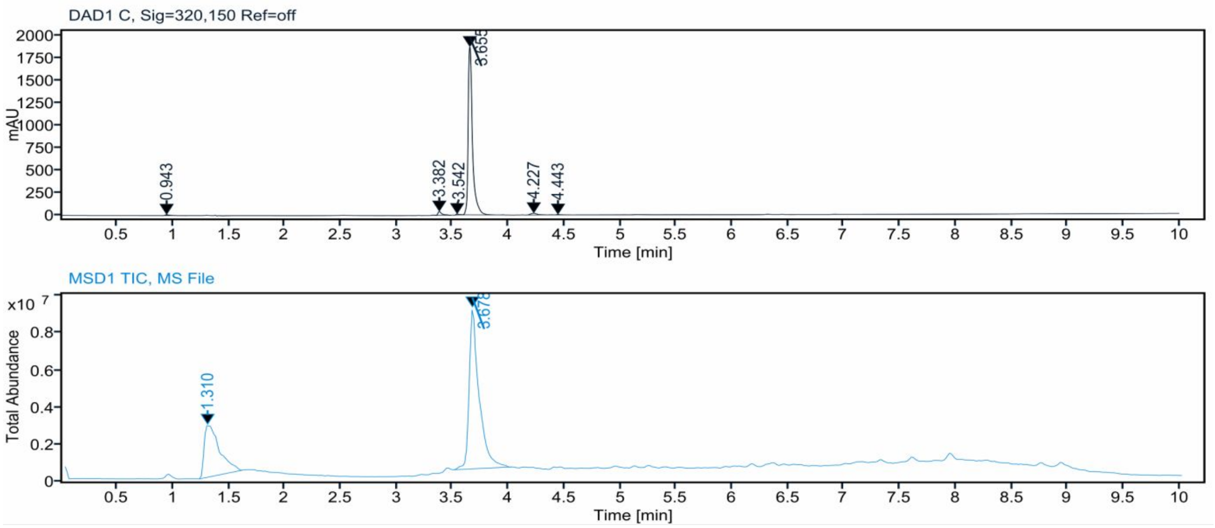

Sample Purity

Signal Description DAD1 C, Sig=320,150 Ref=off

Sample Name	Name	RT	Width	Area	Area%	Height
NM444_2_purity_x		0.943	0.034	27.2528	0.52	9.8179
NM444_2_purity_x		3.382	0.031	97.4979	1.85	41.9235
NM444_2_purity_x		3.542	0.034	19.6766	0.37	10.9844
NM444_2_purity_x		3.655	0.037	5030.8701	95.66	1861.4760
NM444_2_purity_x		4.227	0.059	75.2694	1.43	19.9413
NM444_2_purity_x		4.443	0.025	8.2873	0.16	4.4741

Max Area% 95.665

UV Signal Purity>95% Pass

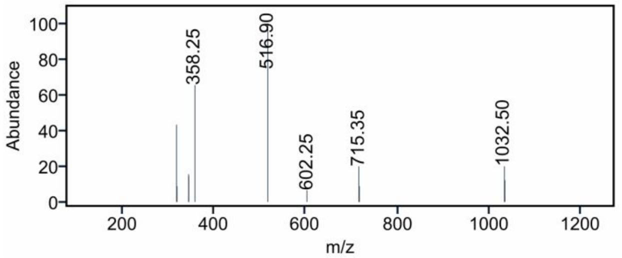

Figure S49: LC/MS spectra of purified compound **4-a^{neg}** at 320 nm wavelength with $[M+H]^+_{calc.}$ = 1032.52 m/z.

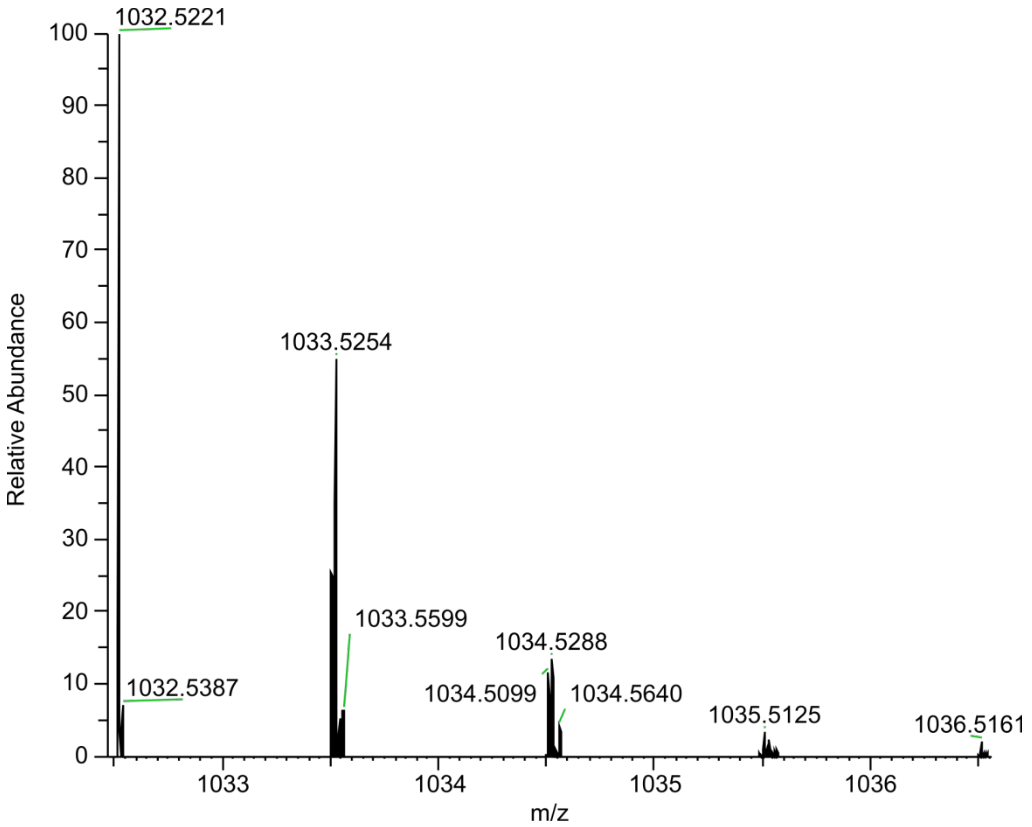

Figure S50: High-resolution mass spectrum of compound **4-a**[neg] with $[M+H]^+_{calc.}$ = 1032.5236 m/z.

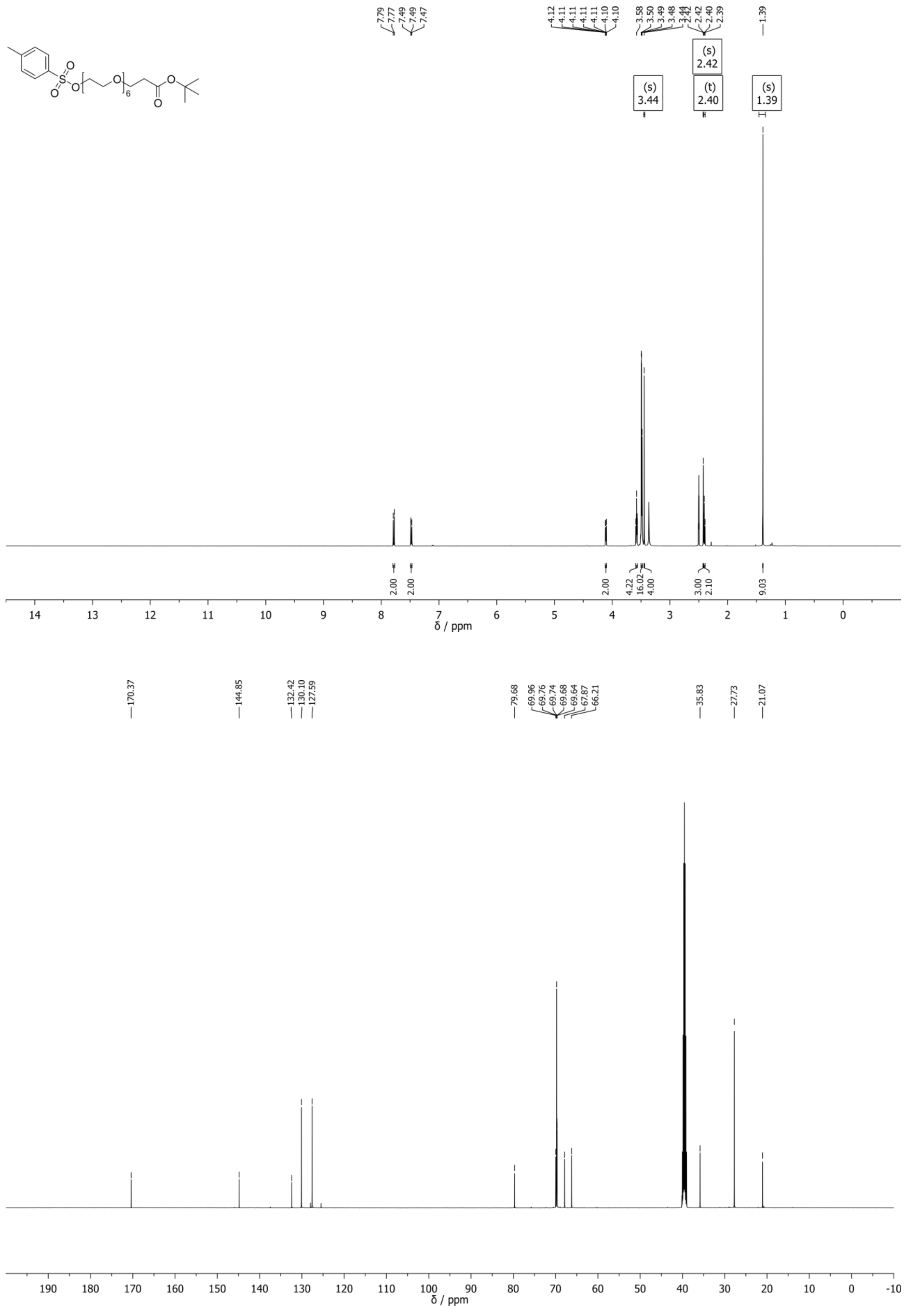

Figure S51: ^{1}H- (top) and ^{13}C-NMR (bottom) spectra (500 MHz and 126 MHz, 298 K, DMSO-d_6) and chemical structure of compound **27**.

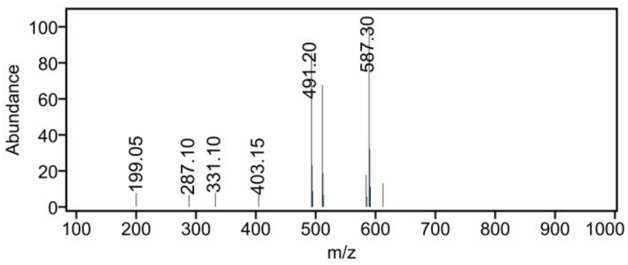

Figure 52: ESI-MS spectrum of compound **27** with $[M+H]^{+}_{calc.}$ = 587.28 m/z.

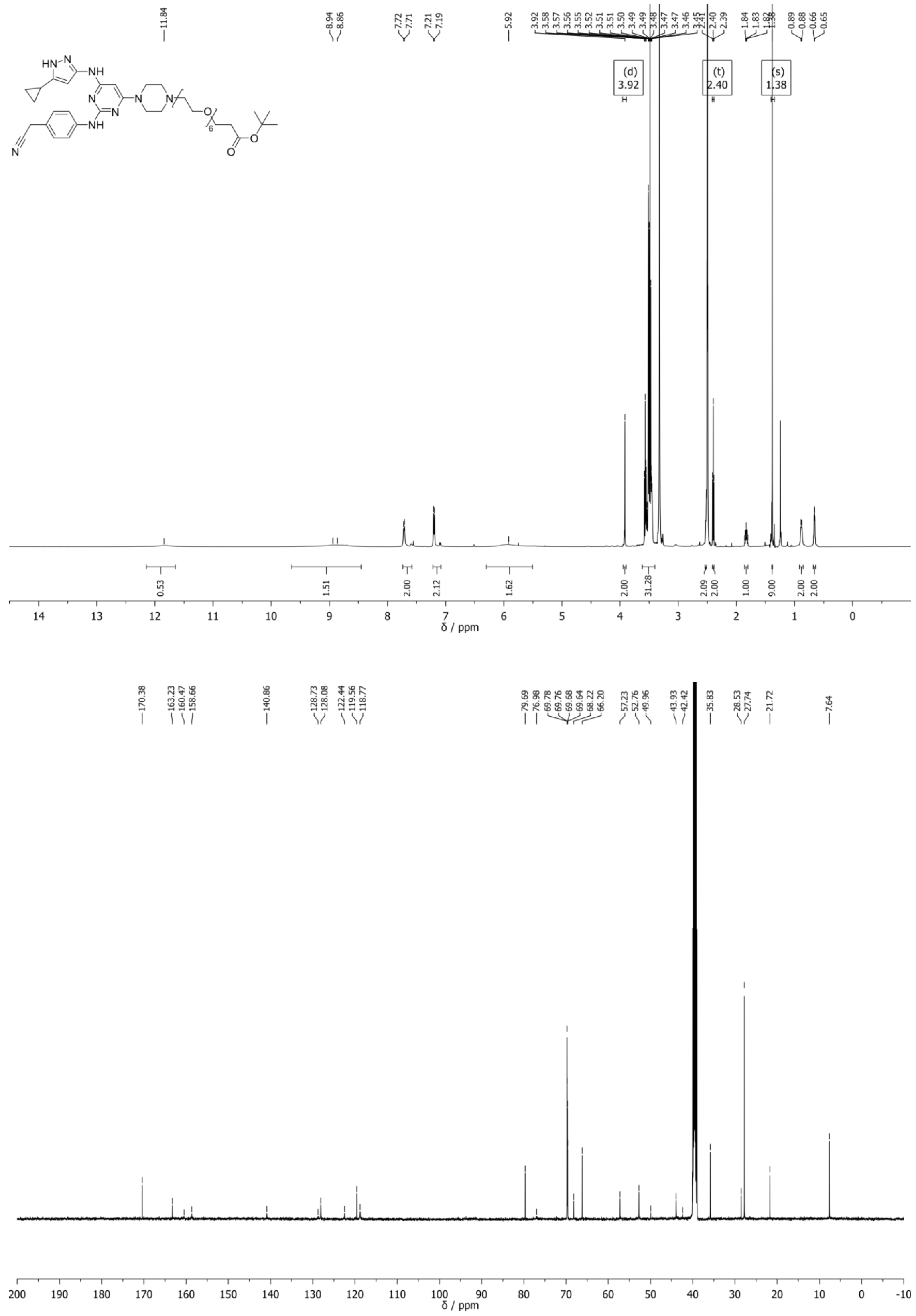

Figure S53: ^{1}H- (top) and ^{13}C-NMR (bottom) spectra (500 MHz and 126 MHz, 298 K, DMSO-d_6) and chemical structure of compound **30**.

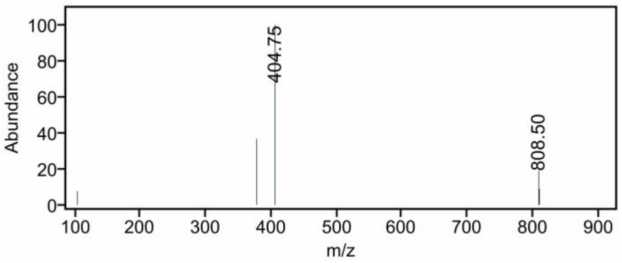

Figure 54: ESI-MS spectrum of compound **30** with $[M+H]^{+}_{calc.}$ = 808.47 m/z.

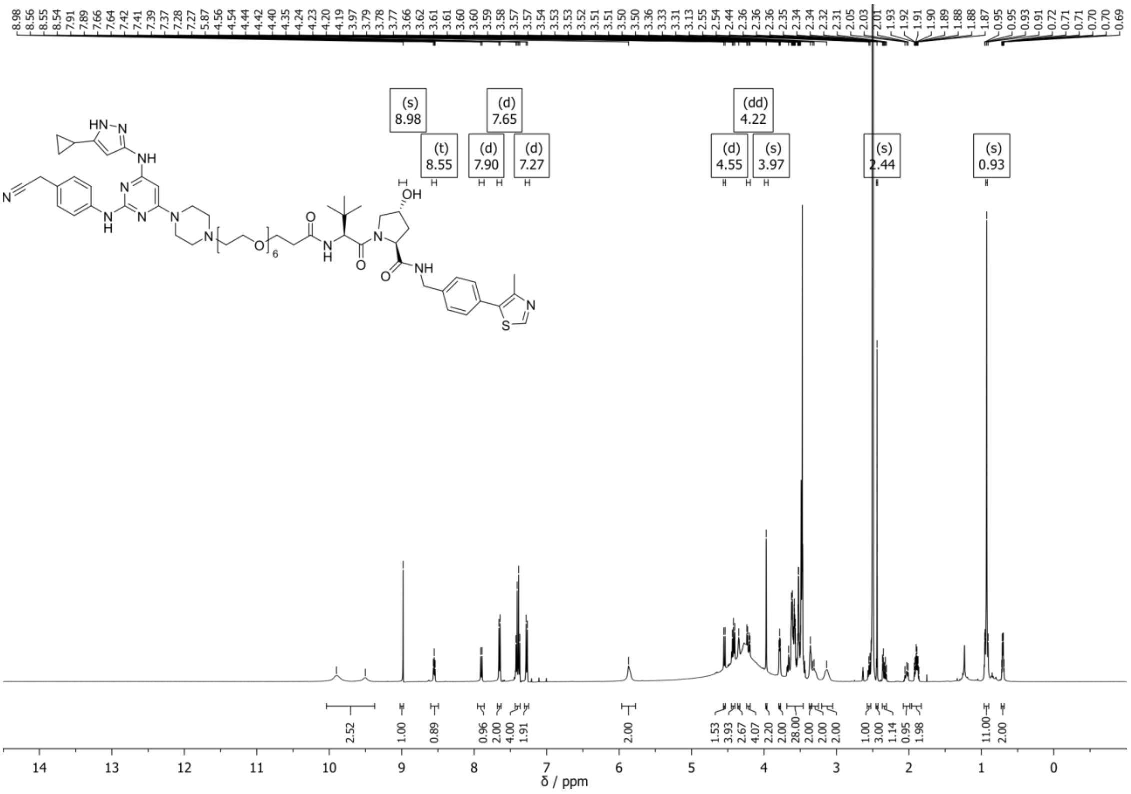

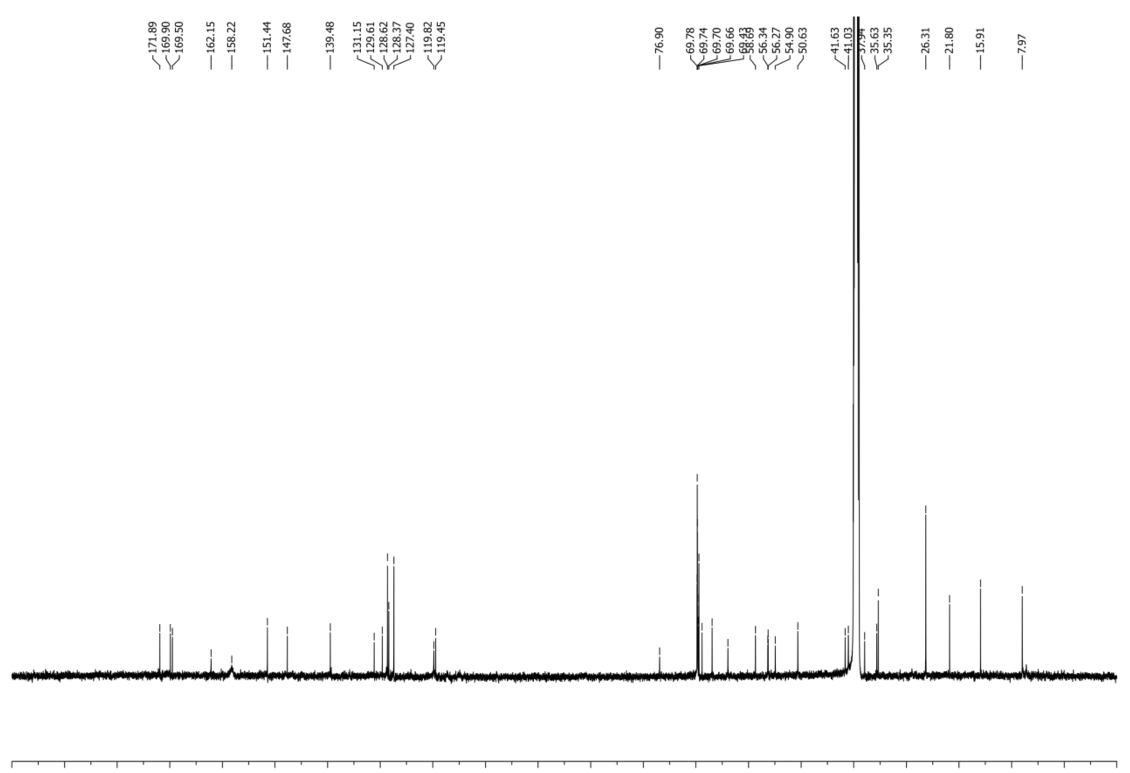

Figure S55: ^{1}H- (top) and ^{13}C-NMR (bottom) spectra (500 MHz and 126 MHz, 298 K, DMSO-d_6) and chemical structure of compound **4-b**.

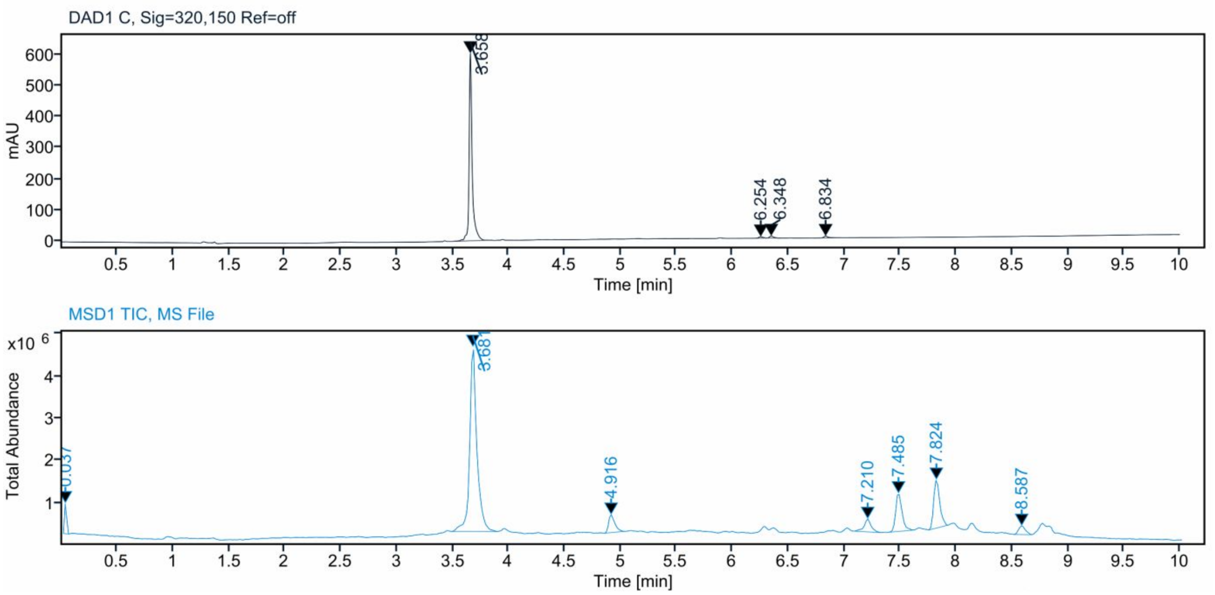

Sample Purity

Signal Description DAD1 C, Sig=320,150 Ref=off

Sample Name	Name	RT	Width	Area	Area%	Height
NM281_purity2		3.658	0.024	1149.6278	96.25	601.9084
NM281_purity2		6.254	0.039	11.9432	1.00	4.8556
NM281_purity2		6.348	0.037	16.1732	1.35	6.5426
NM281_purity2		6.834	0.038	16.6634	1.40	5.8961

Max Area% 96.251

UV Signal Purity>95% Pass

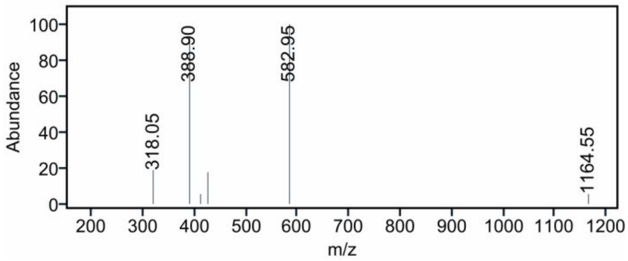

Figure S56: LC/MS spectra of purified compound **4-b** at 320 nm wavelength with $[M+H]^+_{calc.}$ = 1164.60 m/z.

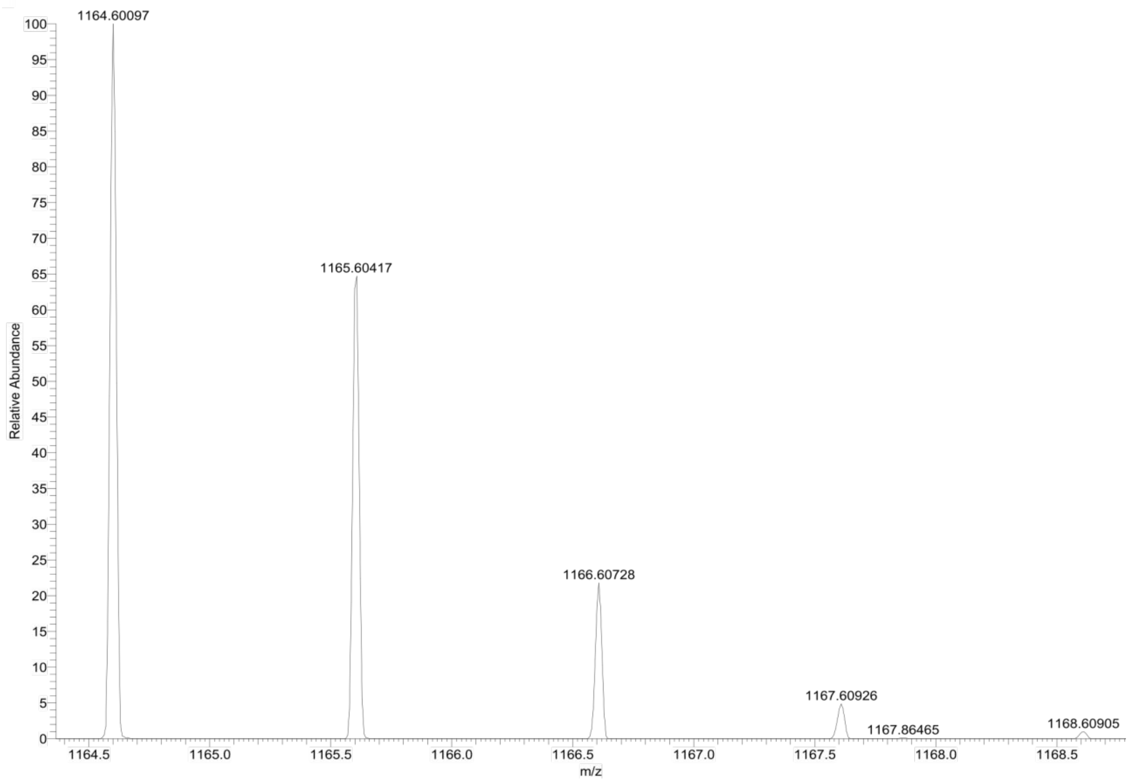

Figure S57: High-resolution mass spectrum of compound **4-b** with $[M+H]^+_{calc.}$ = 1164.60228 m/z.

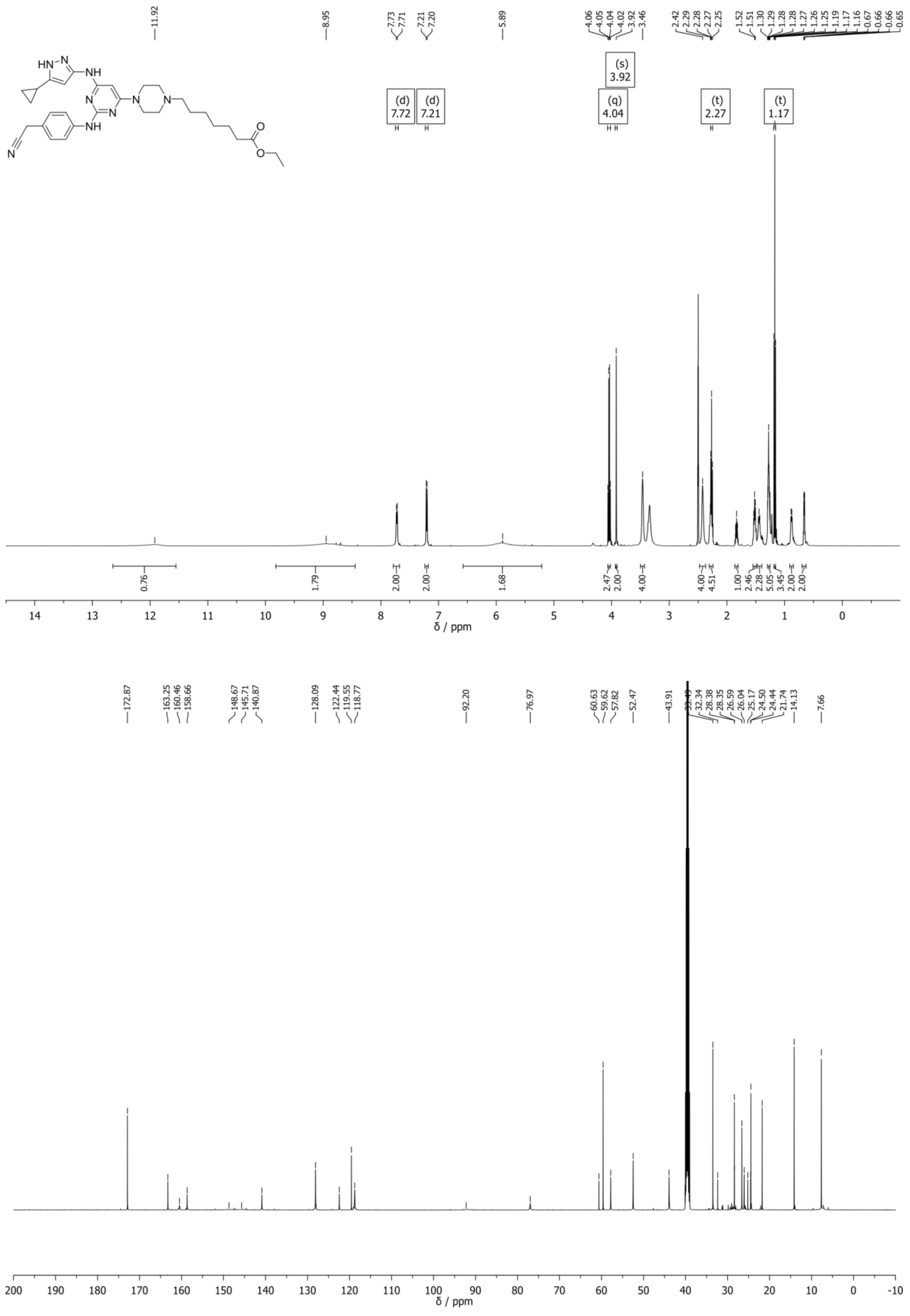

Figure S58: ^{1}H- (top) and ^{13}C-NMR (bottom) spectra (500 MHz and 126 MHz, 298 K, DMSO-d_6) and chemical structure of compound **31**.

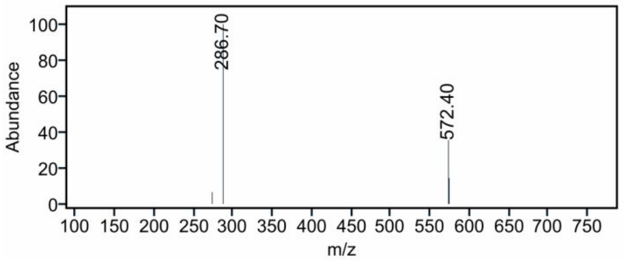

Figure S59: ESI-MS spectrum of compound **31** with $[M+H]^+_{calc.}$ = 572.35 m/z.

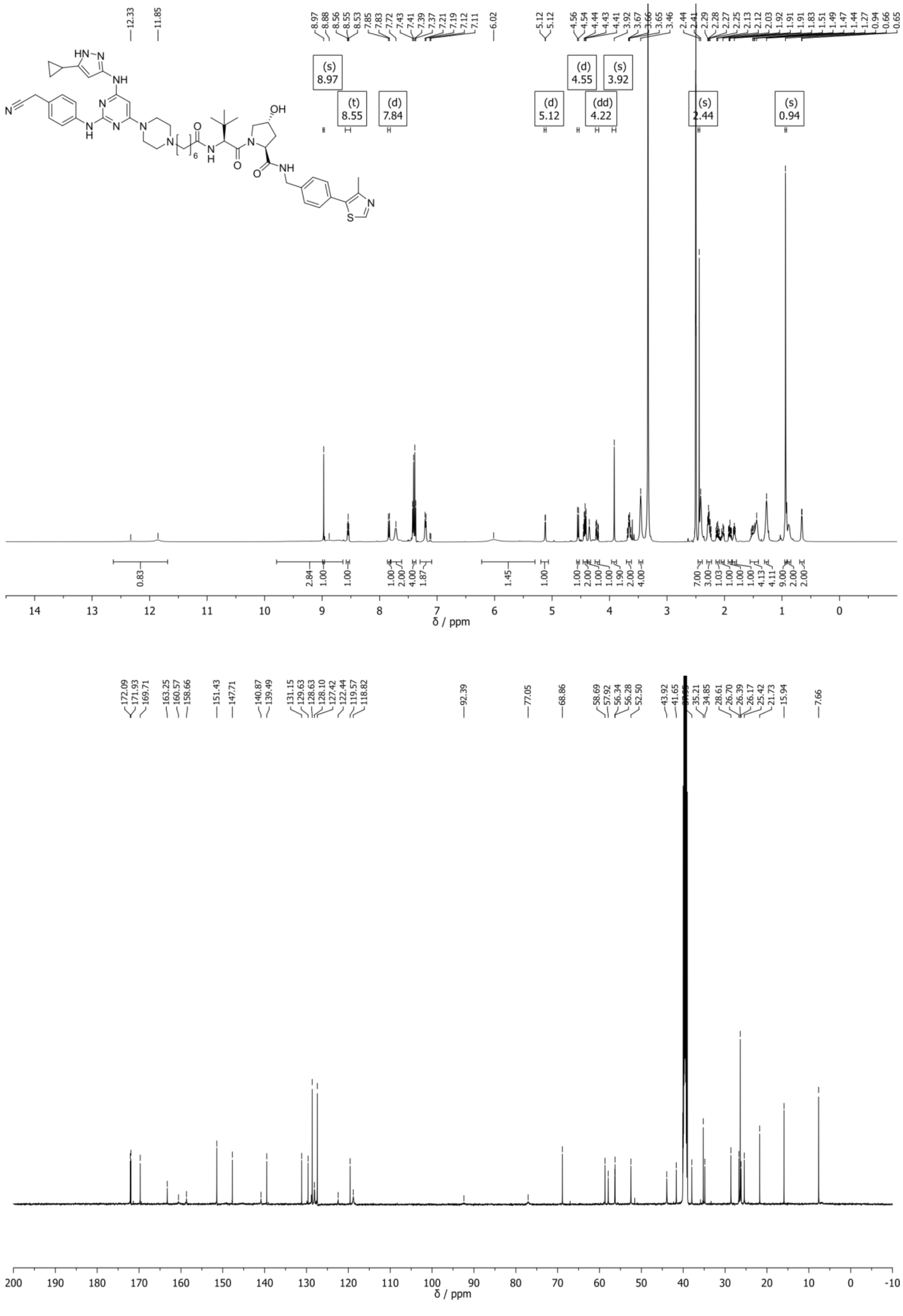

Figure S60: ^{1}H- (top) and ^{13}C-NMR (bottom) spectra (500 MHz and 126 MHz, 298 K, DMSO-d_6) and chemical structure of compound **4-c**.

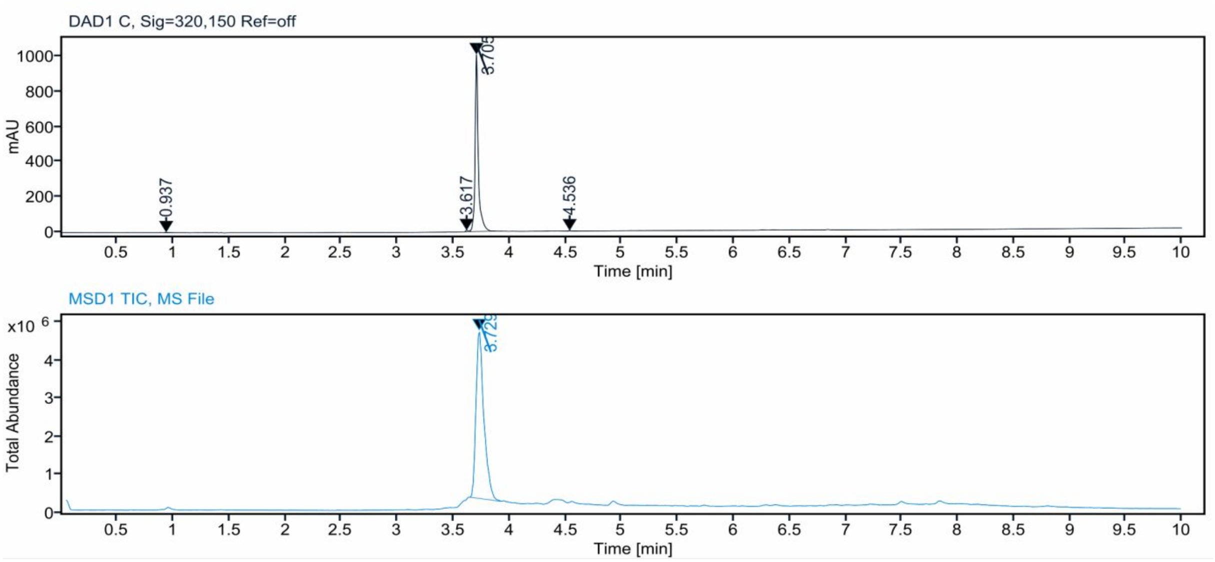

Sample Purity

Signal Description	DAD1 C, Sig=320,150 Ref=off

Sample Name	Name	RT	Width	Area	Area%	Height
NM277f11		0.937	0.030	6.2753	0.31	2.5227
NM277f11		3.617	0.043	18.9762	0.94	8.8553
NM277f11		3.705	0.025	1997.6278	98.50	1002.8005
NM277f11		4.536	0.025	5.0685	0.25	2.9324

Max Area%	98.505
UV Signal Purity>95%	Pass

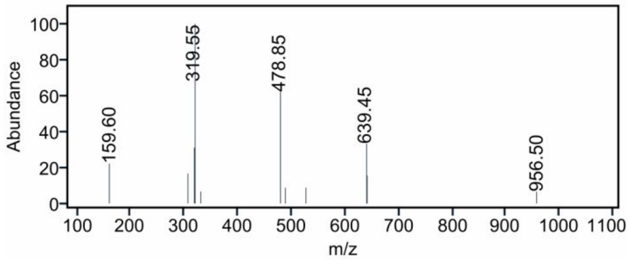

Figure S61: LC/MS spectra of purified compound **4-c** at 320 nm wavelength with $[M+H]^{+}_{calc.}$ = 956.51 m/z.

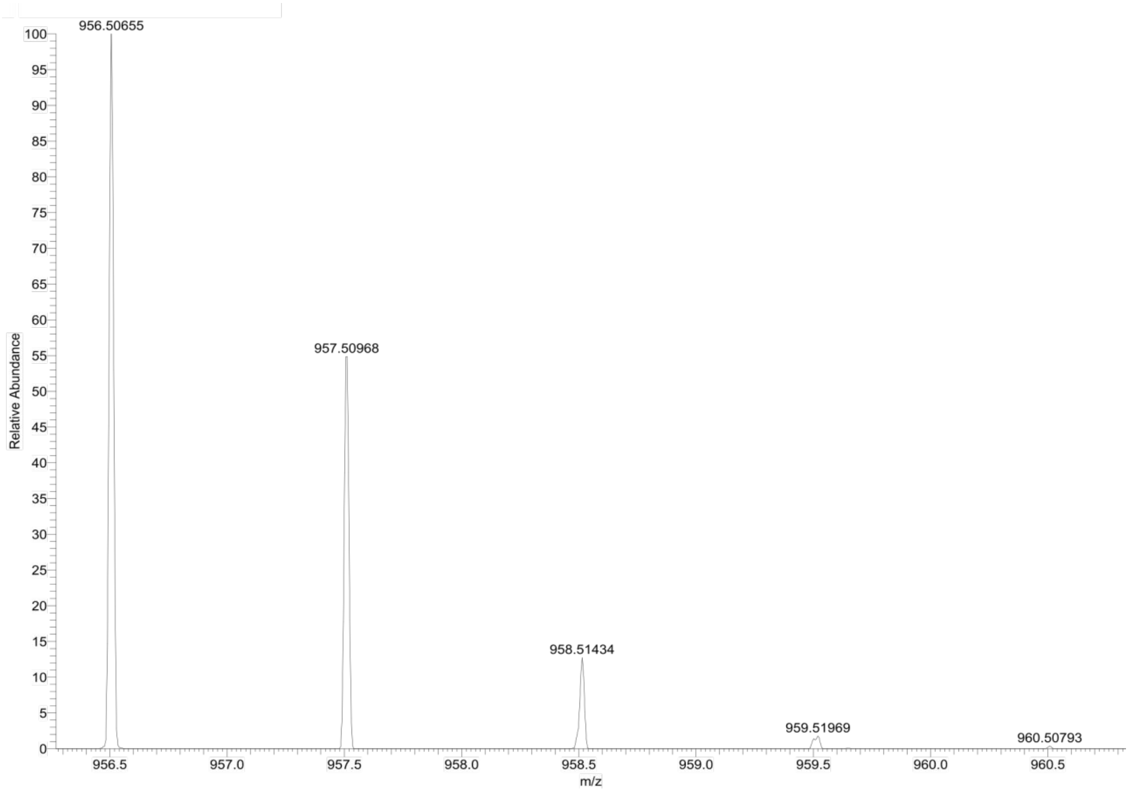

Figure S62: High-resolution mass spectrum of compound **4-c** with $[M+H]^{+}_{calc.}$ = 956.50760 m/z.

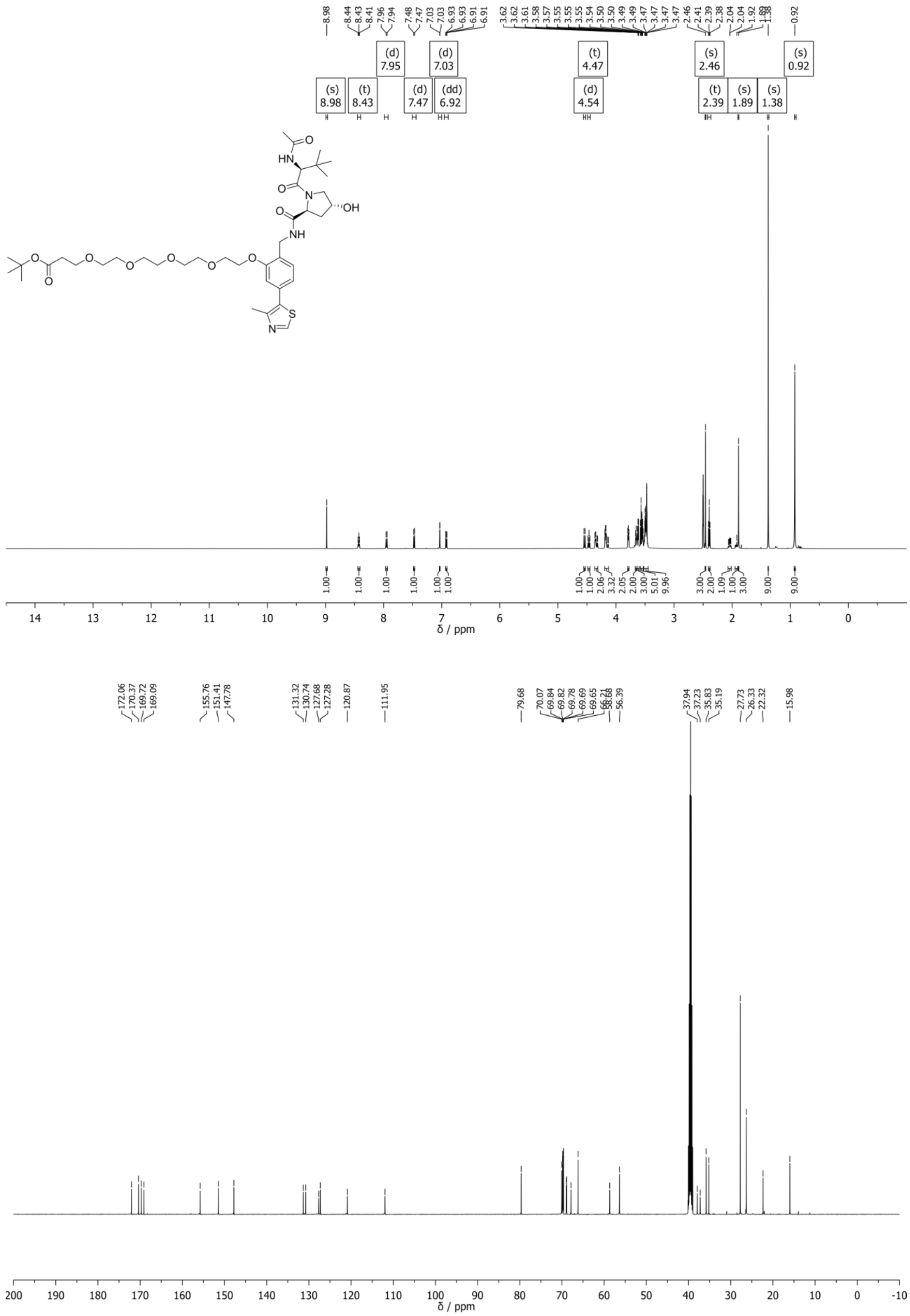

Figure S63: ^{1}H- (top) and ^{13}C-NMR (bottom) spectra (500 MHz and 126 MHz, 298 K, DMSO-d_6) and chemical structure of compound **36**.

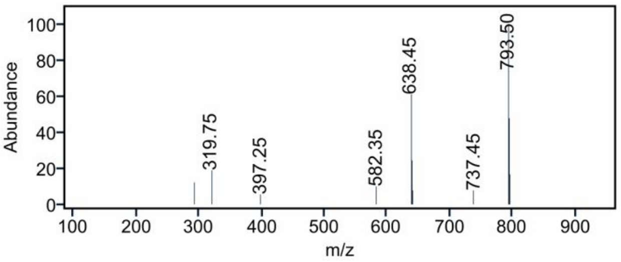

Figure S64: ESI-MS spectrum of compound **36** with $[M+H]^+_{calc.}$ = 793.40 m/z.

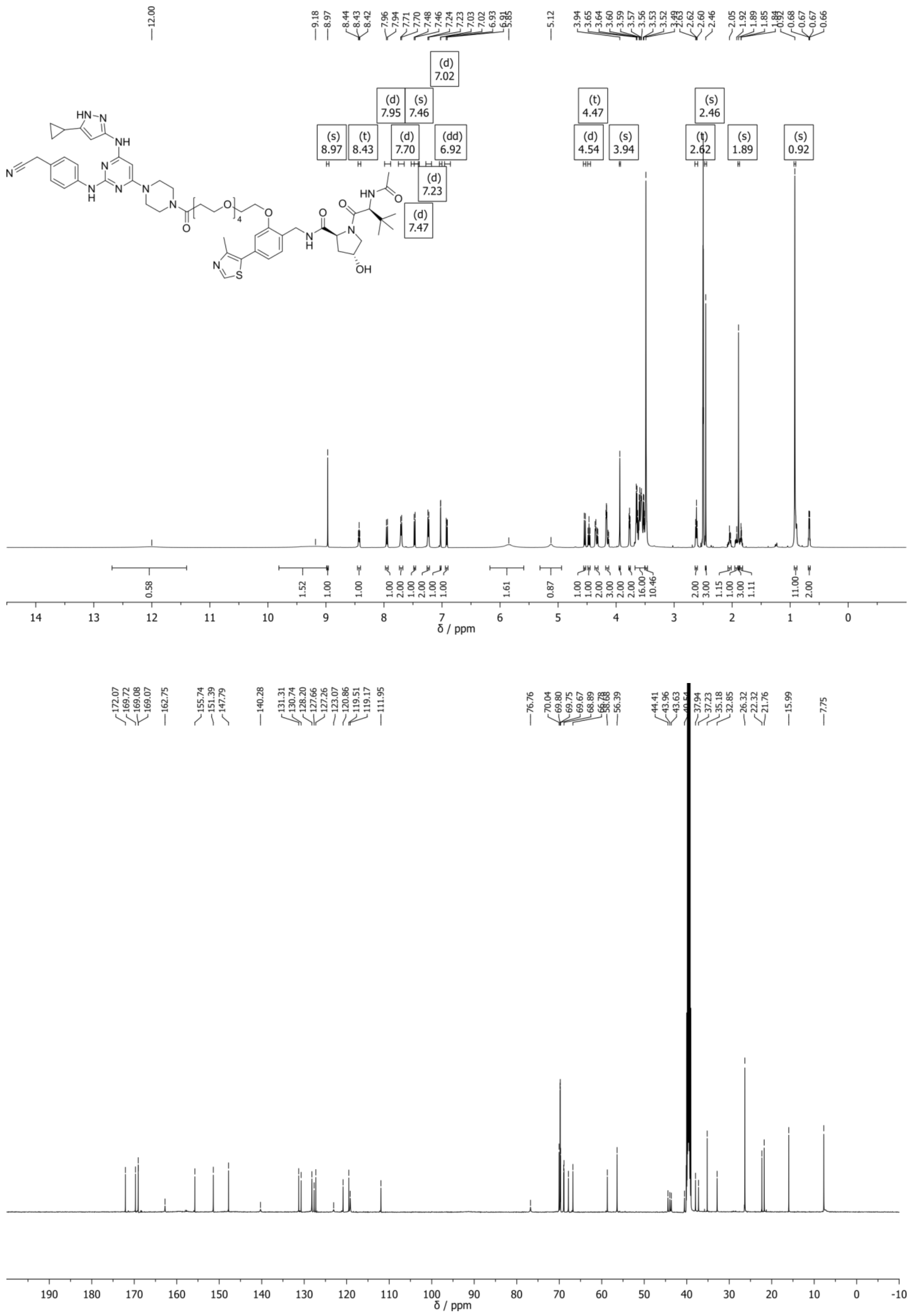

Figure S65: ^{1}H- (top) and ^{13}C-NMR (bottom) spectra (500 MHz and 126 MHz, 298 K, DMSO-d_6) and chemical structure of compound **4-d**.

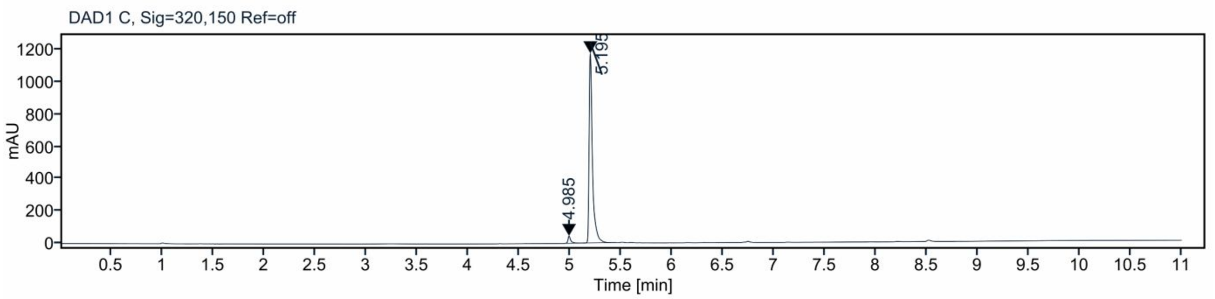

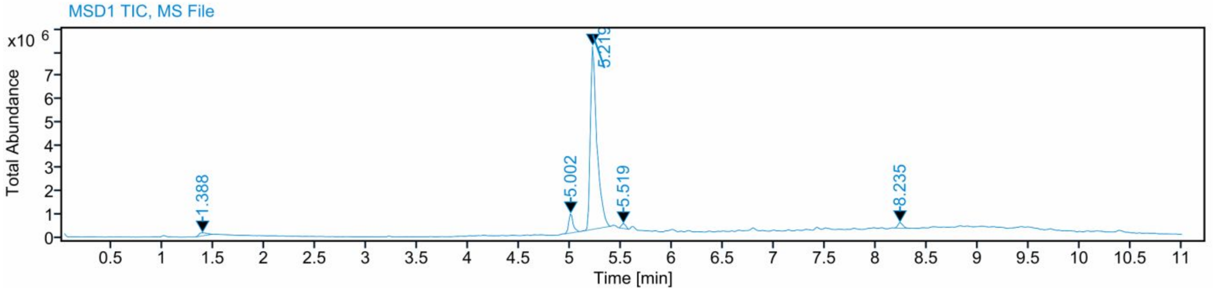

Sample Purity

Signal Description DAD1 C, Sig=320,150 Ref=off

Sample Name	Name	RT	Width	Area	Area%	Height
NM92_puritycheck		4.985	0.025	84.3465	3.06	44.9636
NM92_puritycheck		5.195	0.031	2669.2859	96.94	1175.8917

Max Area% 96.937

UV Signal Purity>95% Pass

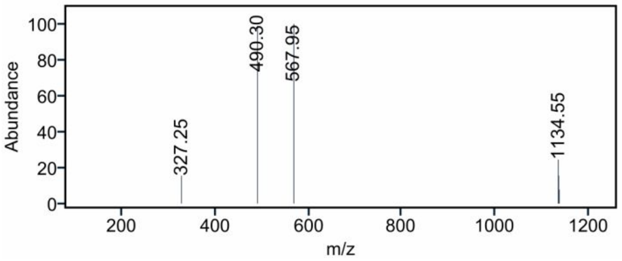

Figure S66: LC/MS spectra of purified compound **4-d** at 320 nm wavelength with $[M+H]^{+}_{calc.}$ = 1134.56 m/z.

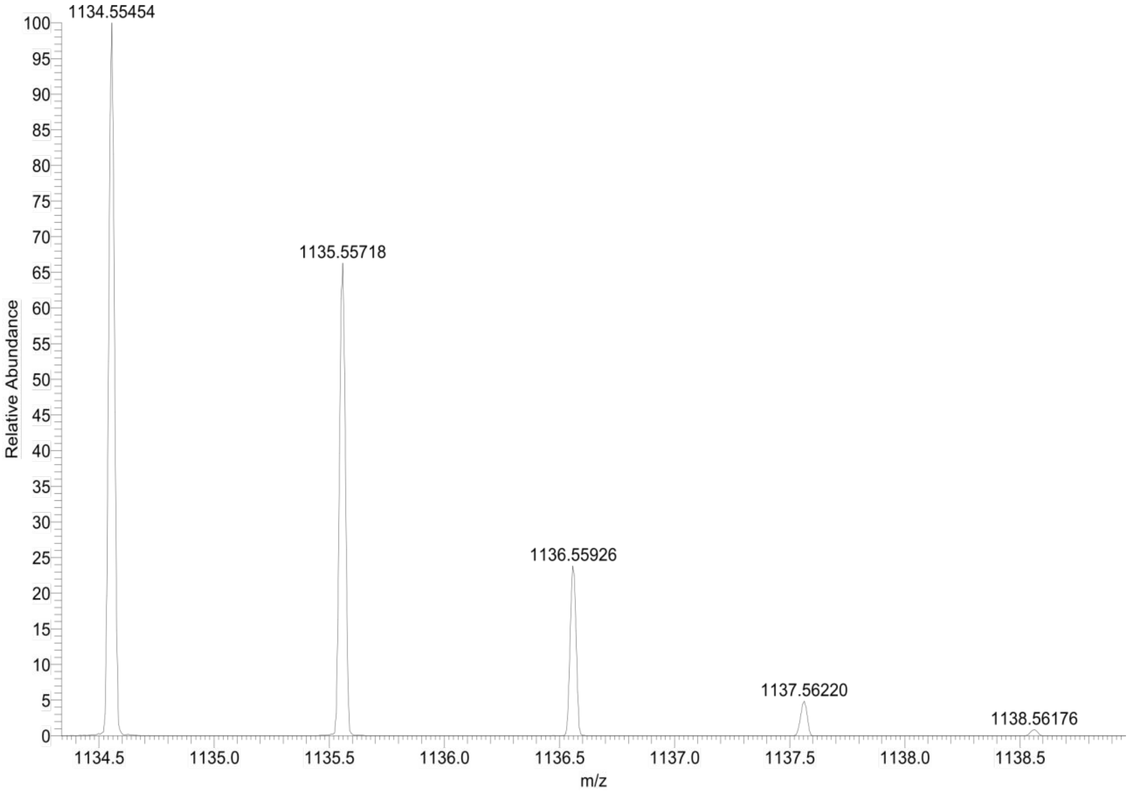

Figure S67: High-resolution mass spectrum of compound **4-d** with $[M+H]^+_{calc.}$ = 1134.55533 m/z.

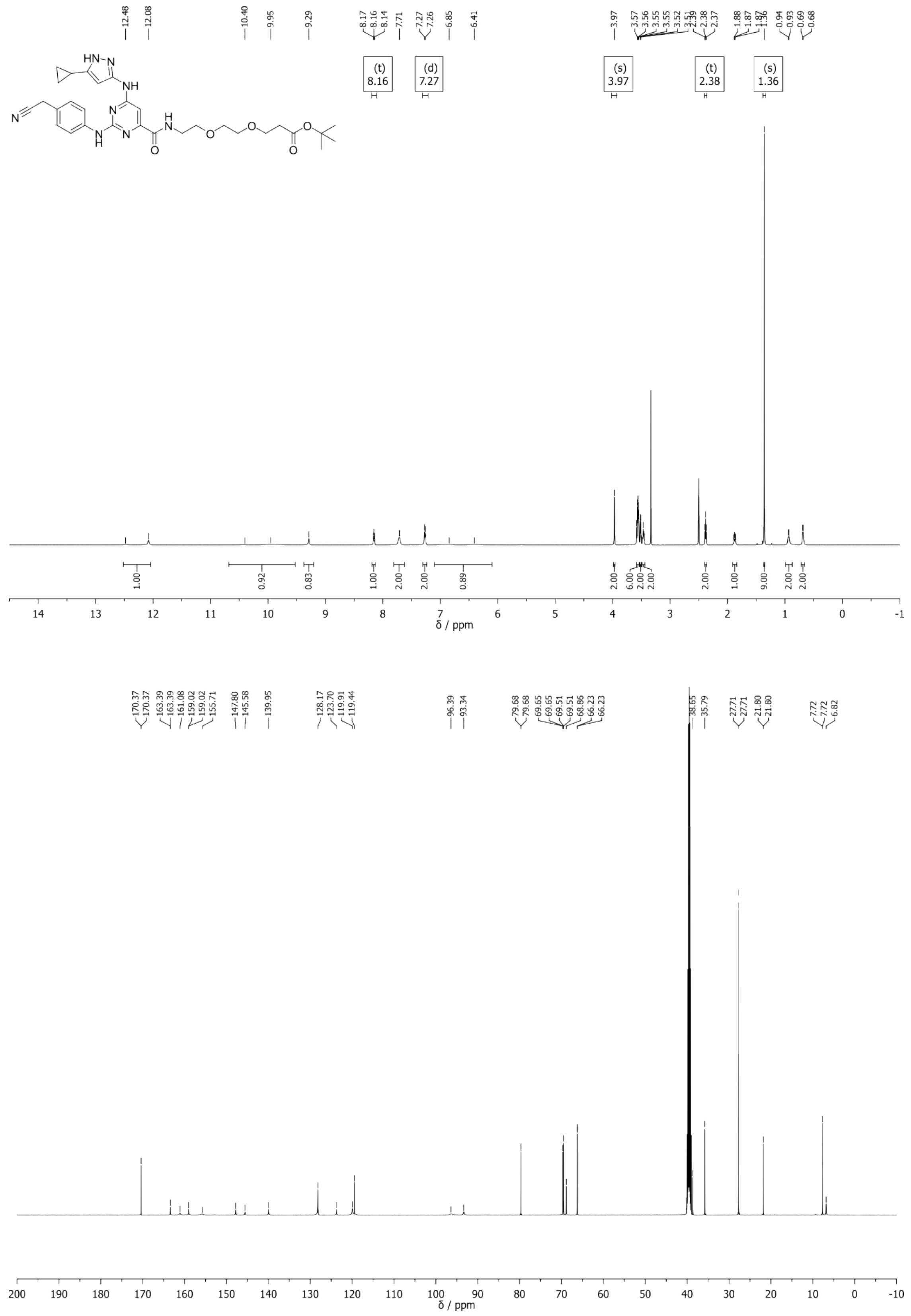

Figure S68: ^{1}H- (top) and ^{13}C-NMR (bottom) spectra (500 MHz and 126 MHz, 298 K, DMSO-d_6) and chemical structure of compound **38**.

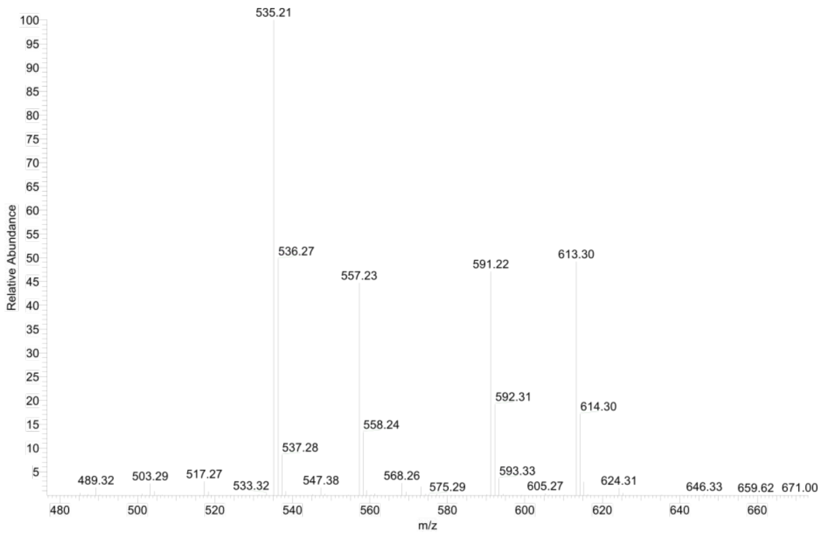

Figure S69: ESI-TLC-MS spectrum of compound **38** with $[M+H]^+_{calc.}$ = 591.30 m/z.

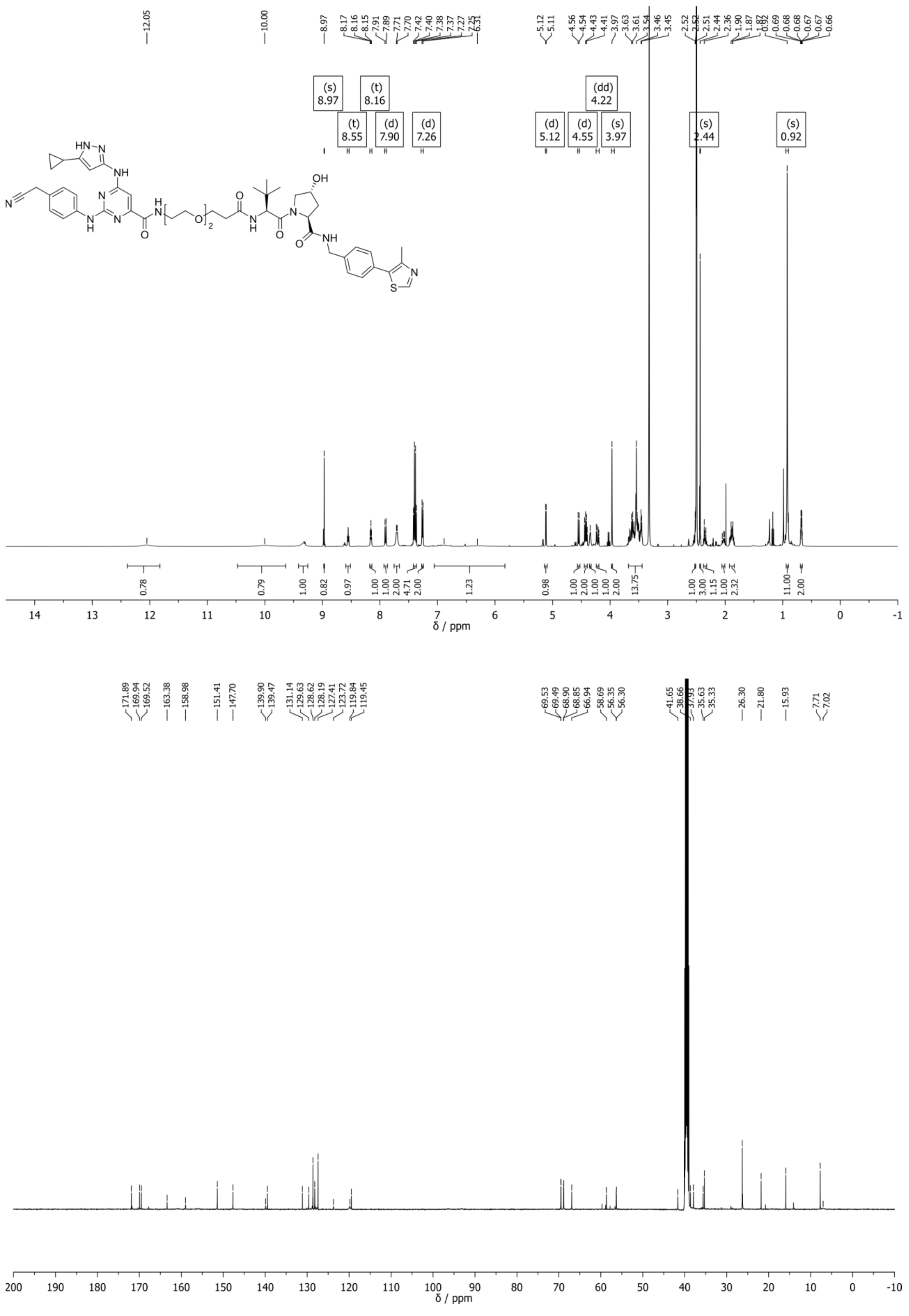

Figure S70: ^{1}H- (top) and ^{13}C-NMR (bottom) spectra (500 MHz and 126 MHz, 298 K, DMSO-d_6) and chemical structure of compound **5-a**.

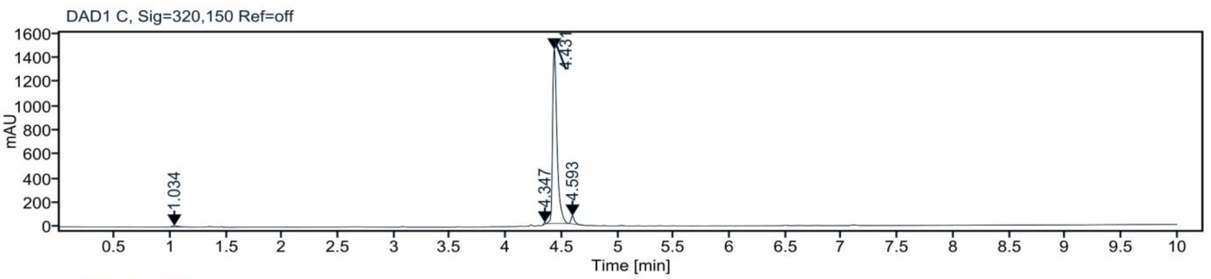

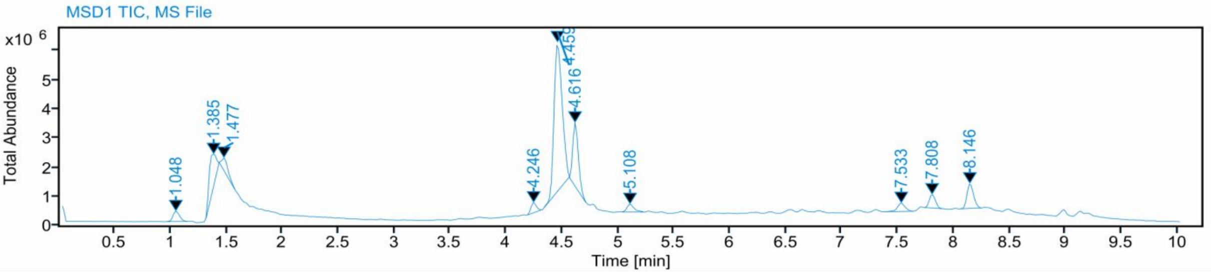

Sample Purity

Signal Description DAD1 C, Sig=320,150 Ref=off

Sample Name	Name	RT	Width	Area	Area%	Height
NM75pur_x		1.034	0.057	41.0937	0.98	10.0062
NM75pur_x		4.347	0.021	16.2760	0.39	13.4916
NM75pur_x		4.431	0.040	3990.6116	95.14	1442.8870
NM75pur_x		4.593	0.031	146.4632	3.49	67.2169

Max Area% 95.140

UV Signal Purity>95% Pass

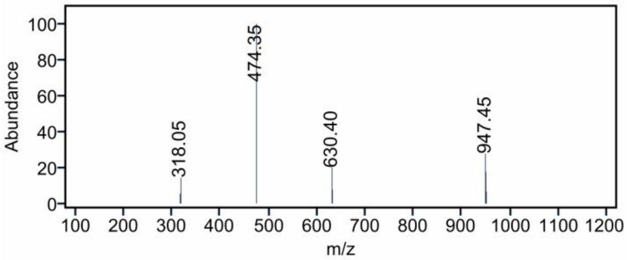

Figure S71: LC/MS spectra of purified compound **5-a** at 320 nm wavelength with $[M+H]^{+}_{calc.}$ = 947.43 m/z.

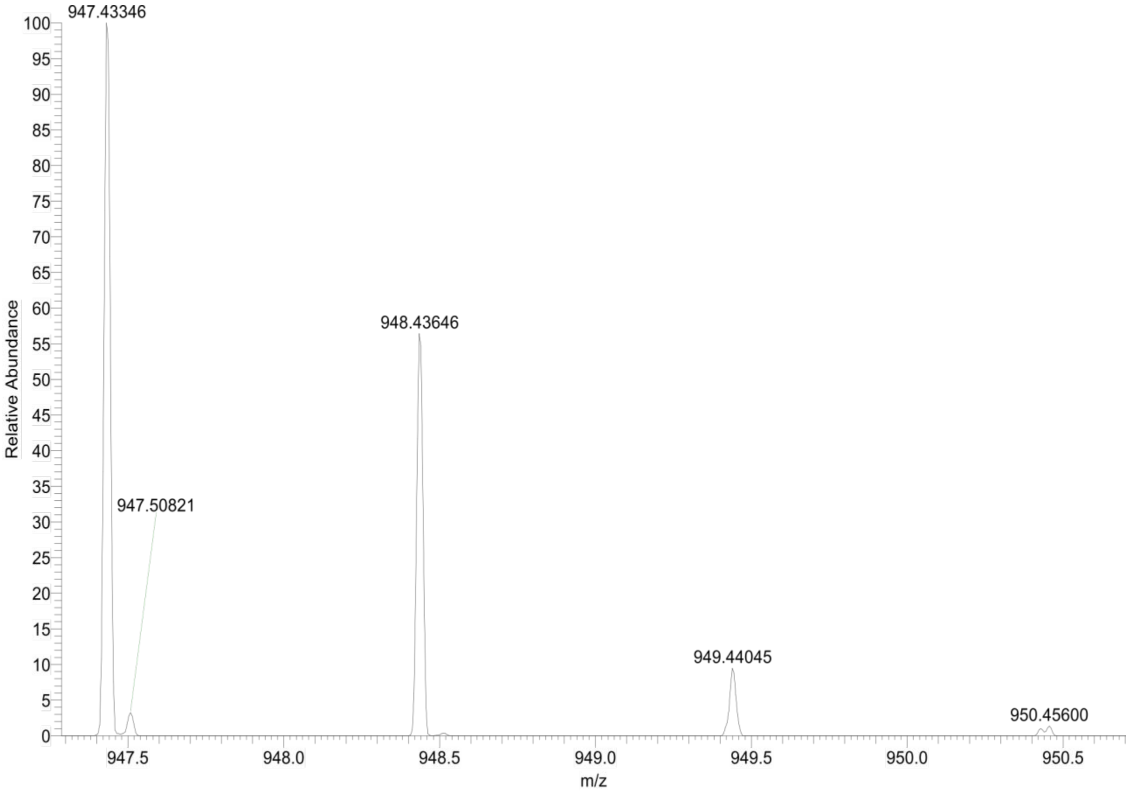

Figure S72: High-resolution mass spectrum of compound **5-a** with $[M+H]^+_{calc.}$ = 947.43449 m/z.

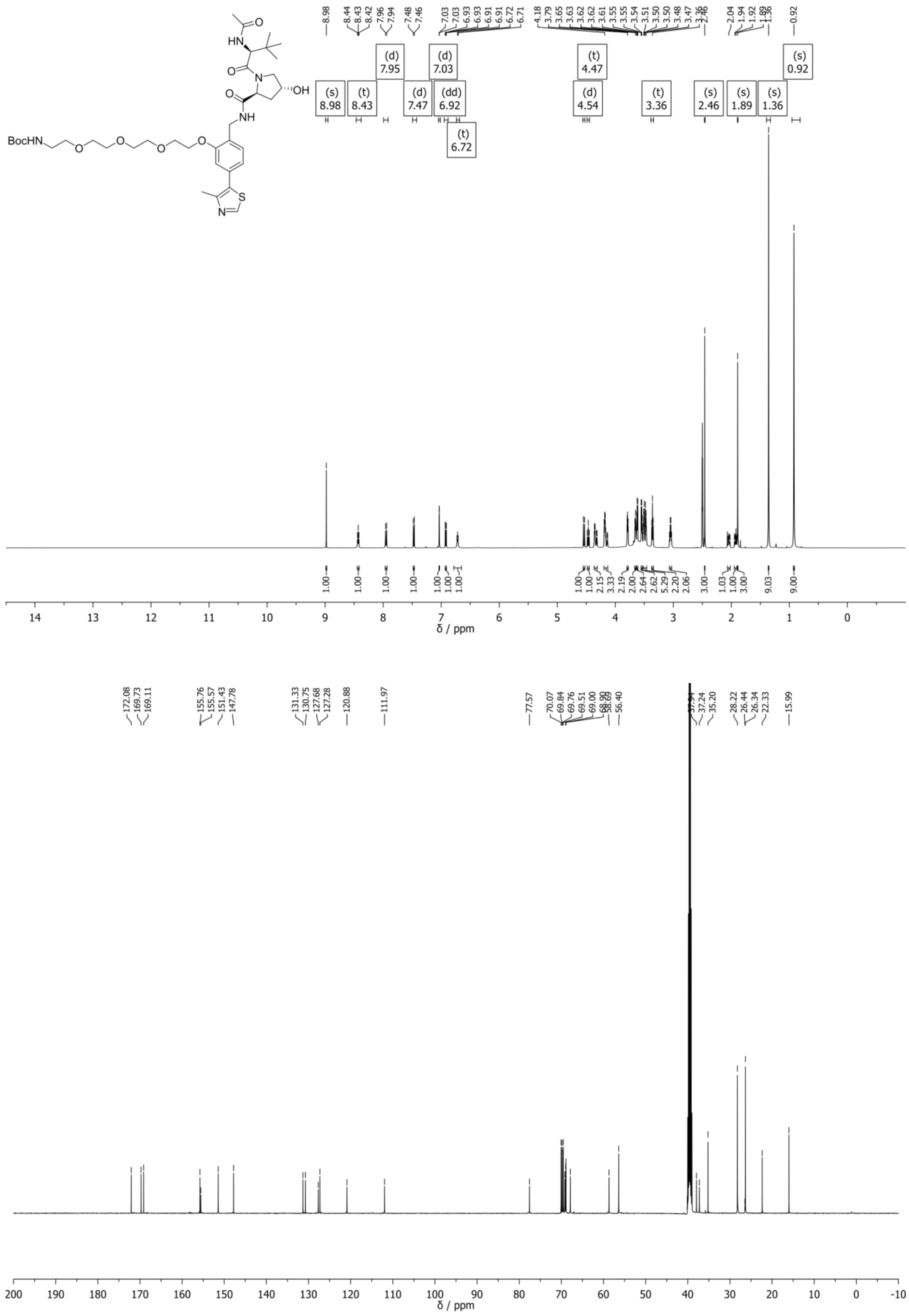

Figure S73: ^{1}H- (top) and ^{13}C-NMR (bottom) spectra (500 MHz and 126 MHz, 298 K, DMSO-d_6) and chemical structure of compound **40**.

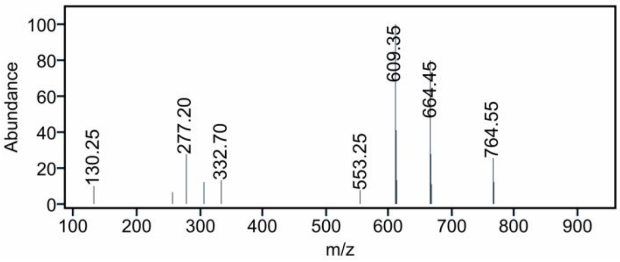

Figure S74: ESI-MS spectrum of compound **40** with $[M+H]^+_{calc.}$ = 764.40 m/z.

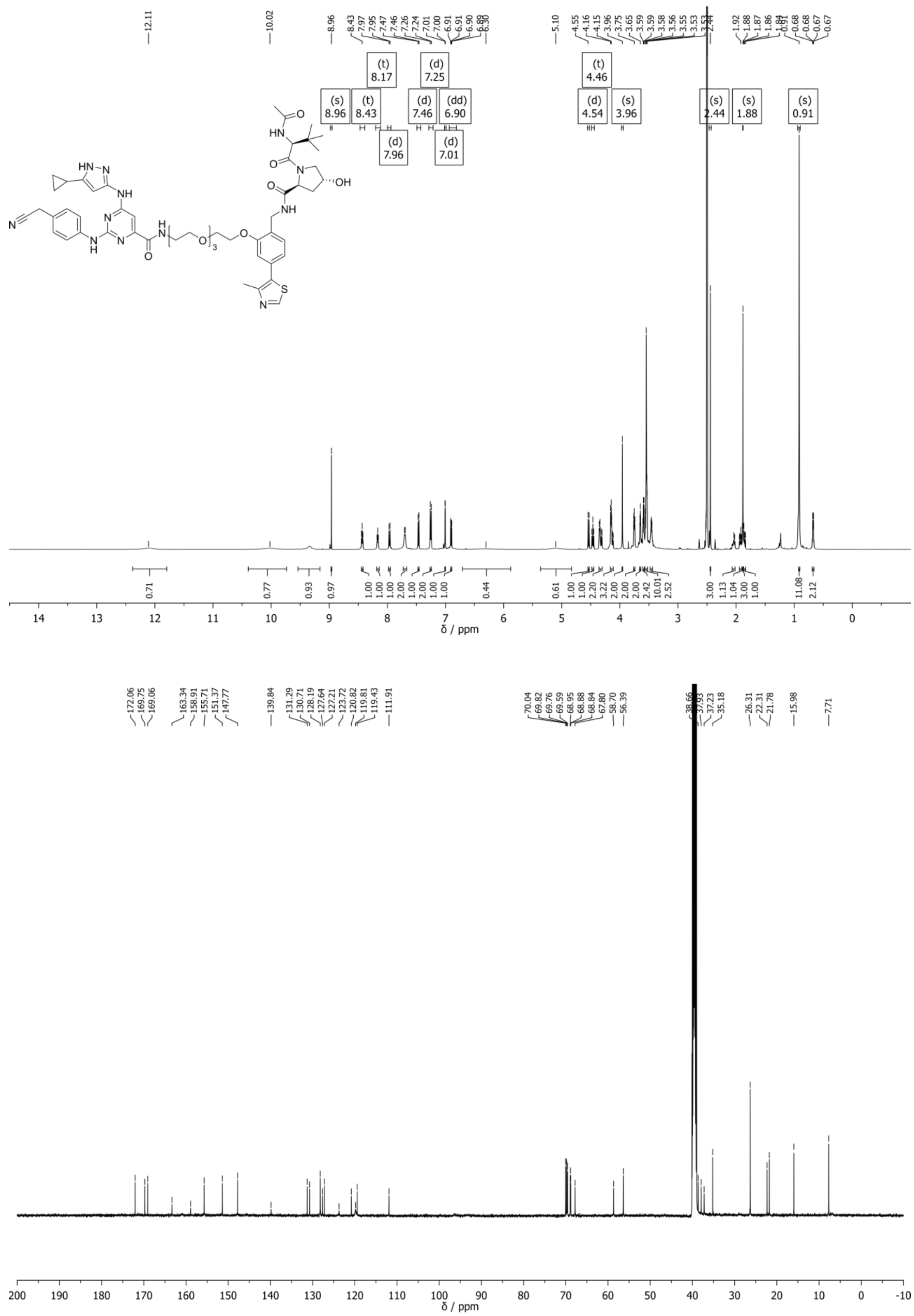

Figure S75: ^{1}H- (top) and ^{13}C-NMR (bottom) spectra (500 MHz and 126 MHz, 298 K, DMSO-d_6) and chemical structure of compound **5-b**.

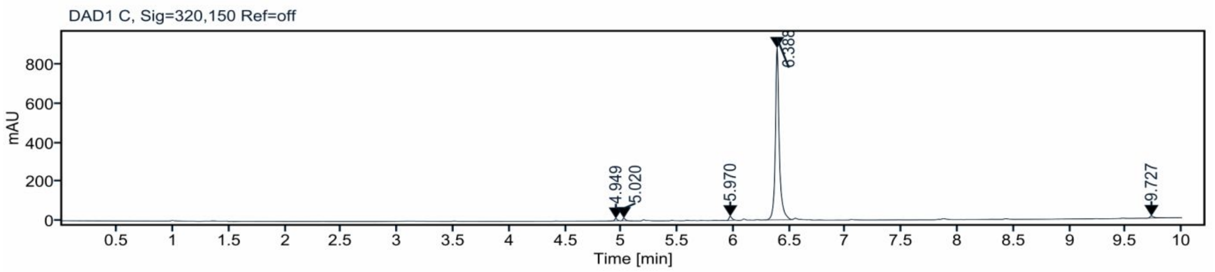

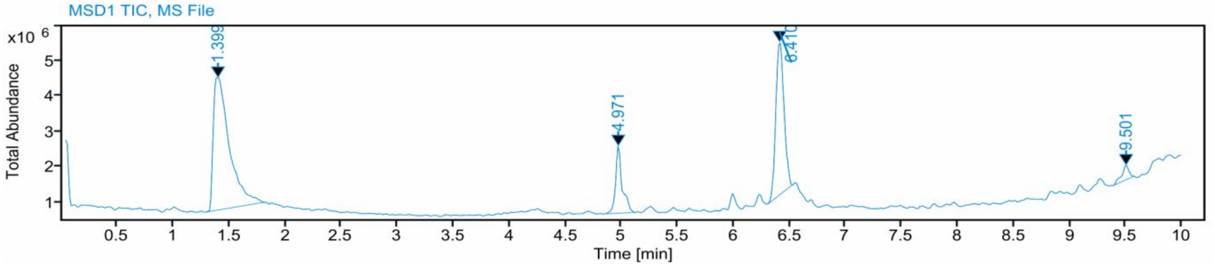

Sample Purity

Signal Description DAD1 C, Sig=320,150 Ref=off

Sample Name	Name	RT	Width	Area	Area%	Height
NM91_purity check		4.949	0.024	21.1355	0.93	12.1352
NM91_purity check		5.020	0.024	23.4608	1.03	13.8315
NM91_purity check		5.970	0.029	38.6316	1.70	21.1087
NM91_purity check		6.388	0.032	2165.8989	95.08	883.8633
NM91_purity check		9.727	0.038	28.9089	1.27	10.7718

Max Area% 95.077

UV Signal Purity>95% Pass

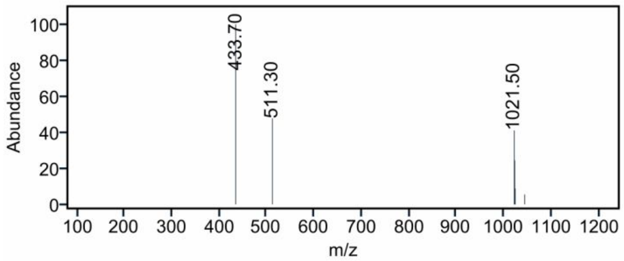

Figure S76: LC/MS spectra of purified compound **5-b** at 320 nm wavelength with $[M+H]^{+}_{calc.}$ = 1021.47 m/z.

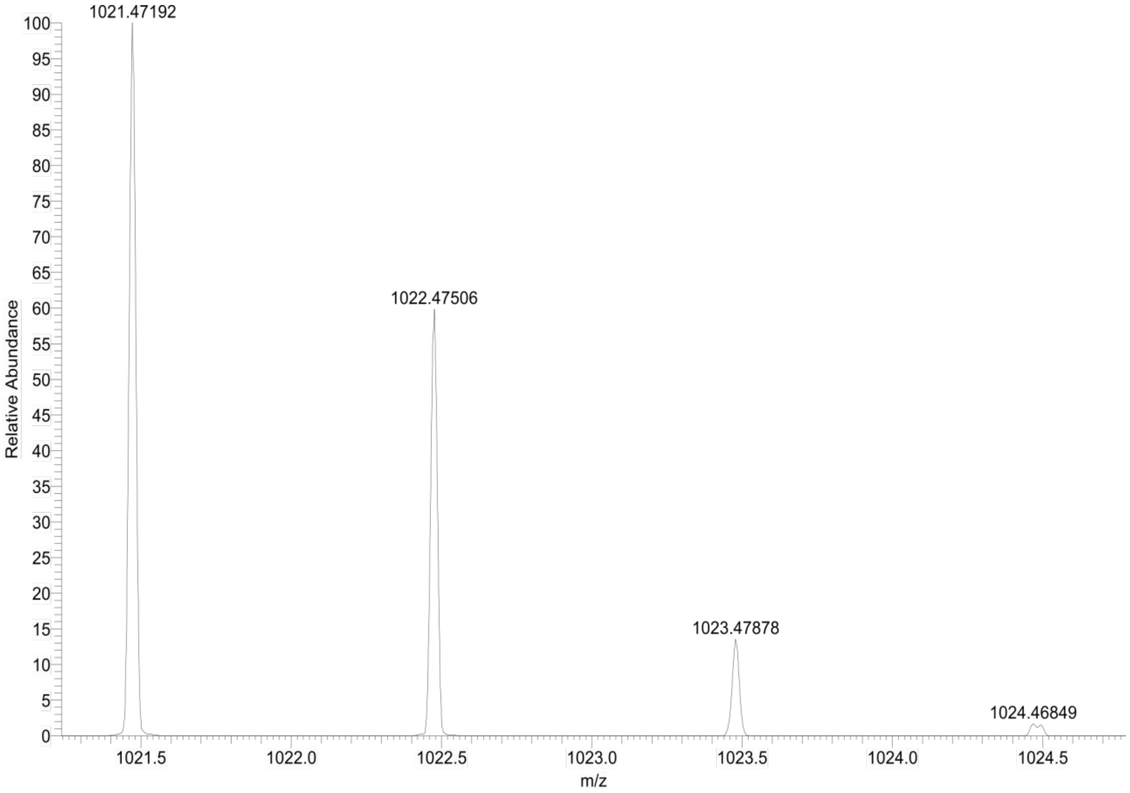

Figure S77: High-resolution mass spectrum of compound **5-b** with $[M+H]^{+}_{calc.}$ = 1021.47151 m/z.

II.c Promiscuous Kinase PROTACs based on Kinase Parent Inhibitor 6

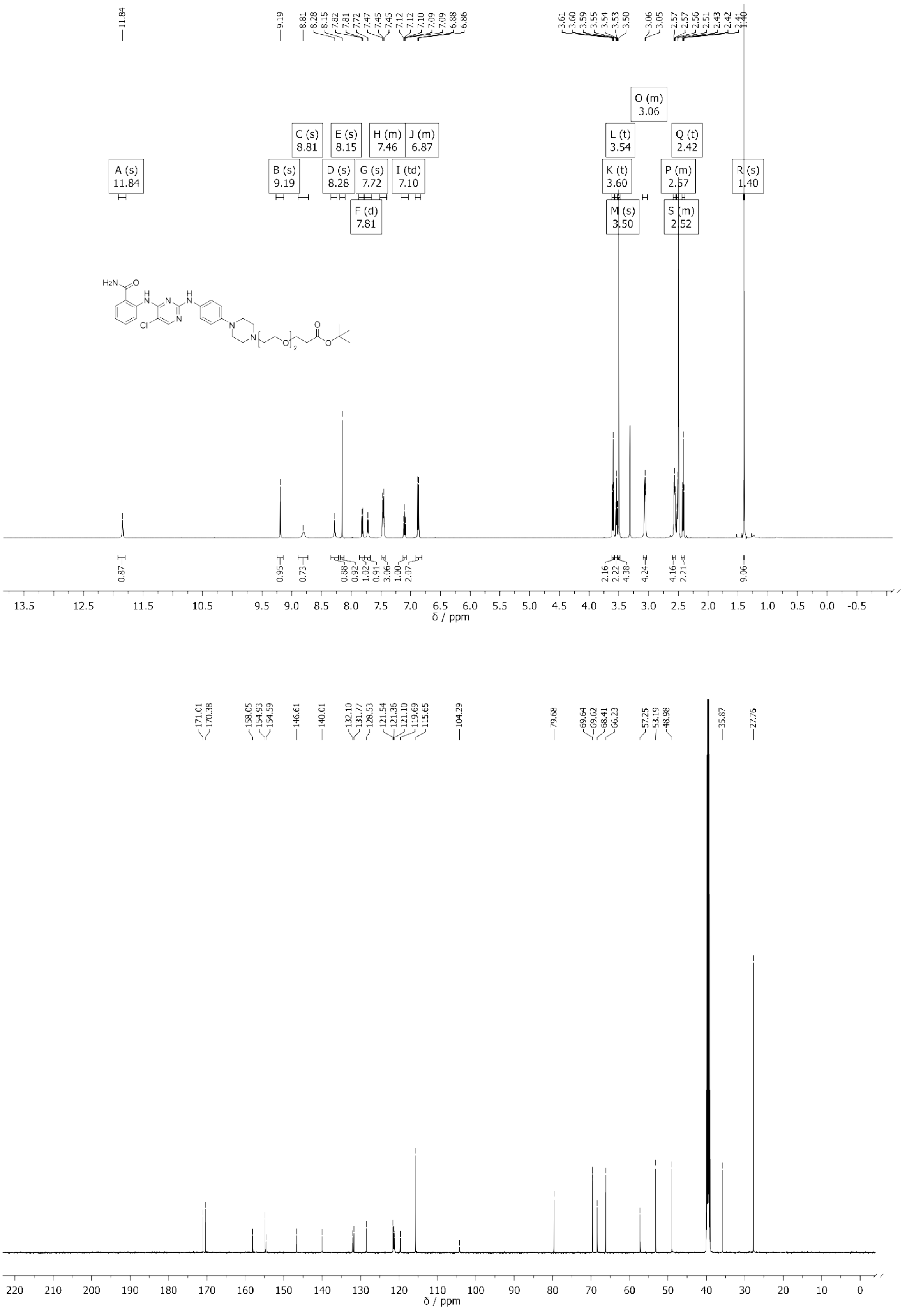

Figure S78: ^{1}H- (top) and 13C-NMR (bottom) spectra (500 MHz and 126 MHz, 298 K, DMSO-d_6) and chemical structure of compound **43**.

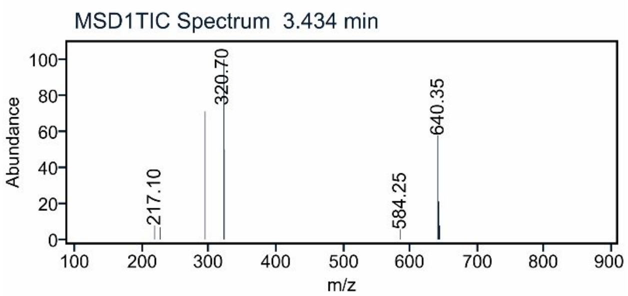

Figure S79: ESI-MS spectrum of compound **43** with $[M+H]^{+}_{calc.}$ = 640.3 m/z.

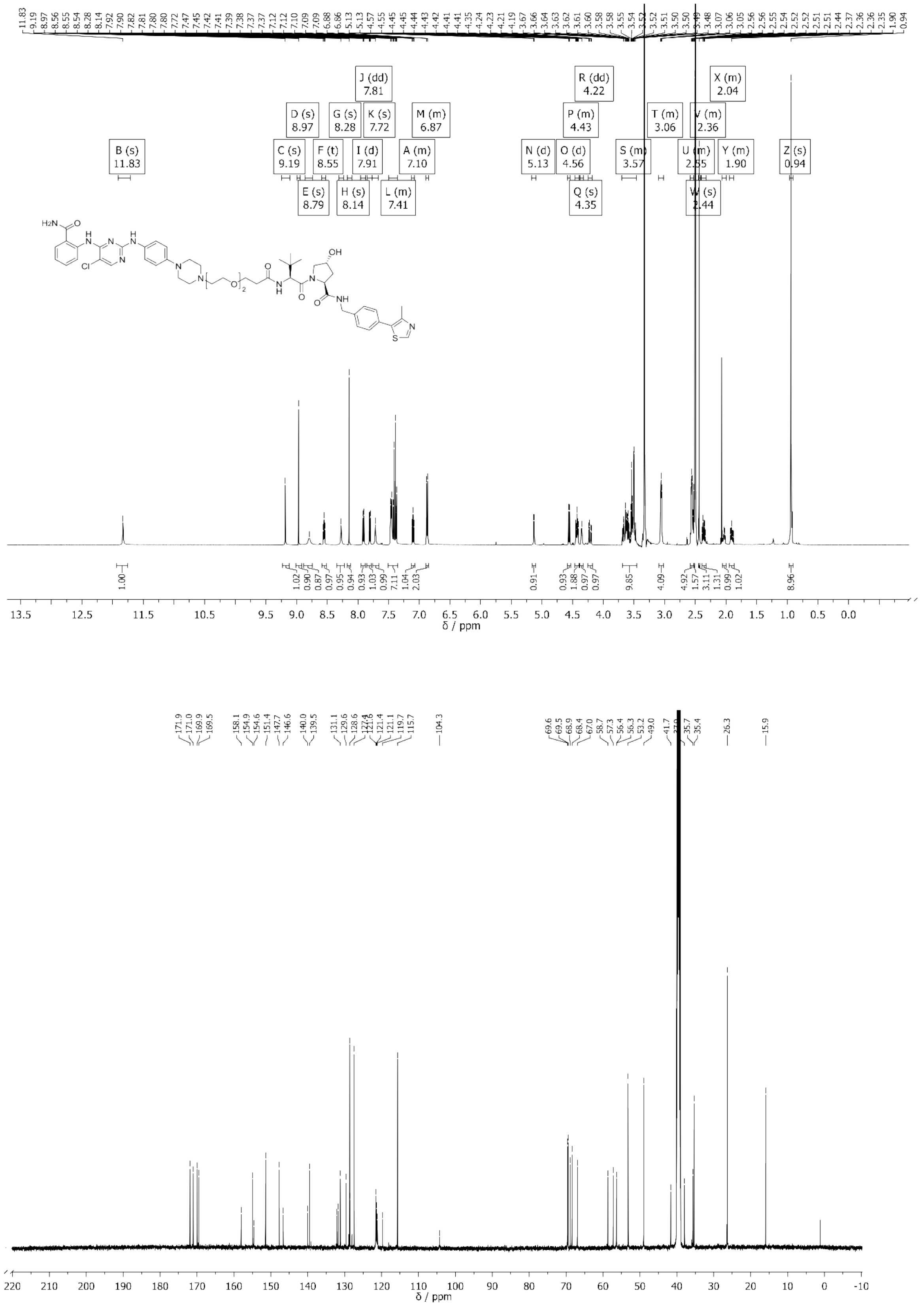

Figure S80: ^{1}H- (top) and 13C-NMR (bottom) spectra (500 MHz and 126 MHz, 298 K, DMSO-d_6) and chemical structure of compound **6-a**.

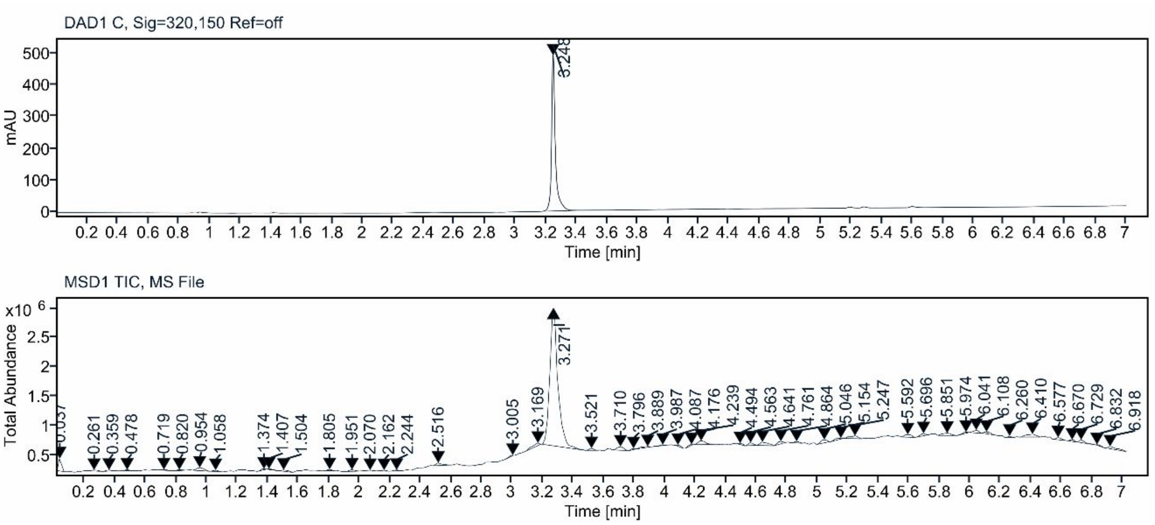

Signal Description DAD1 C, Sig=320,150 Ref=off

Sample Name	Name	RT	Width	Area	Area%	Height
jw-294 10		3.248	0.022	857.0103	100.00	489.7948

Max Area%	100.000
UV Signal Purity>95%	Pass

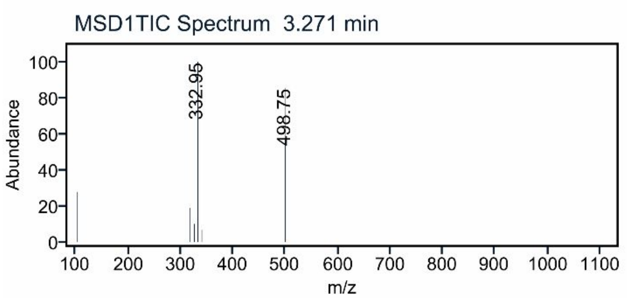

Figure S81: LC/MS spectra of purified compound **6-a** at 320 nm wavelength with $[M/2+H]^{2+}_{calc.}$ = 498.7 m/z.

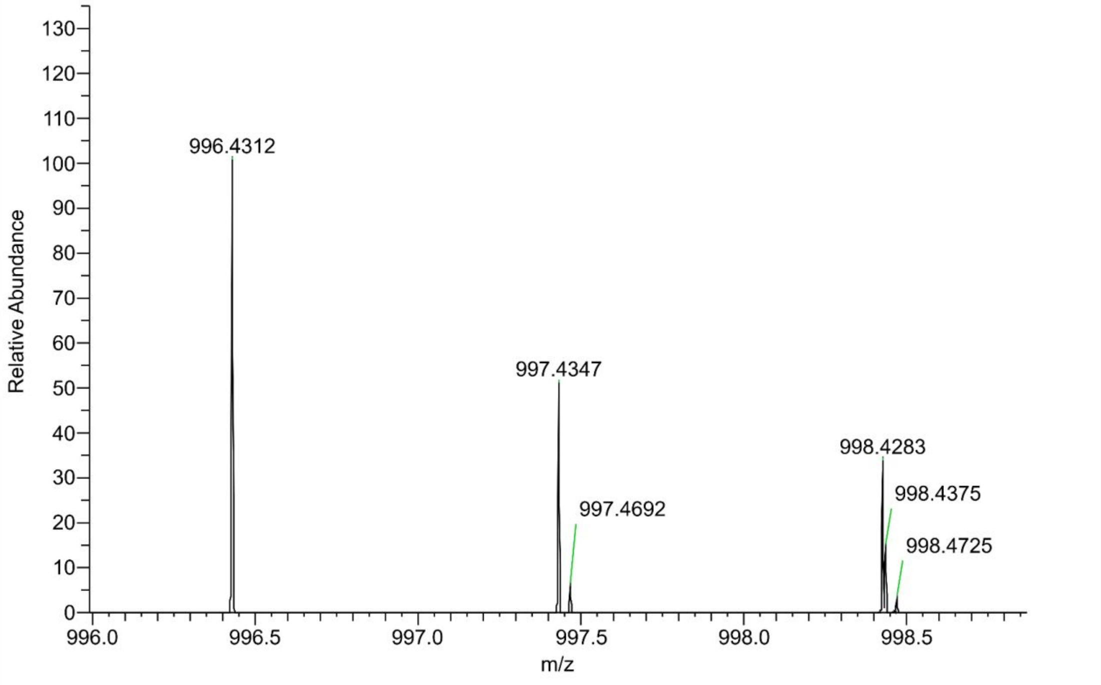

Figure S82: High-resolution mass spectrum of compound **6-a** with $[M+H]^{+}_{calc.}$ = 996.4316 m/z.

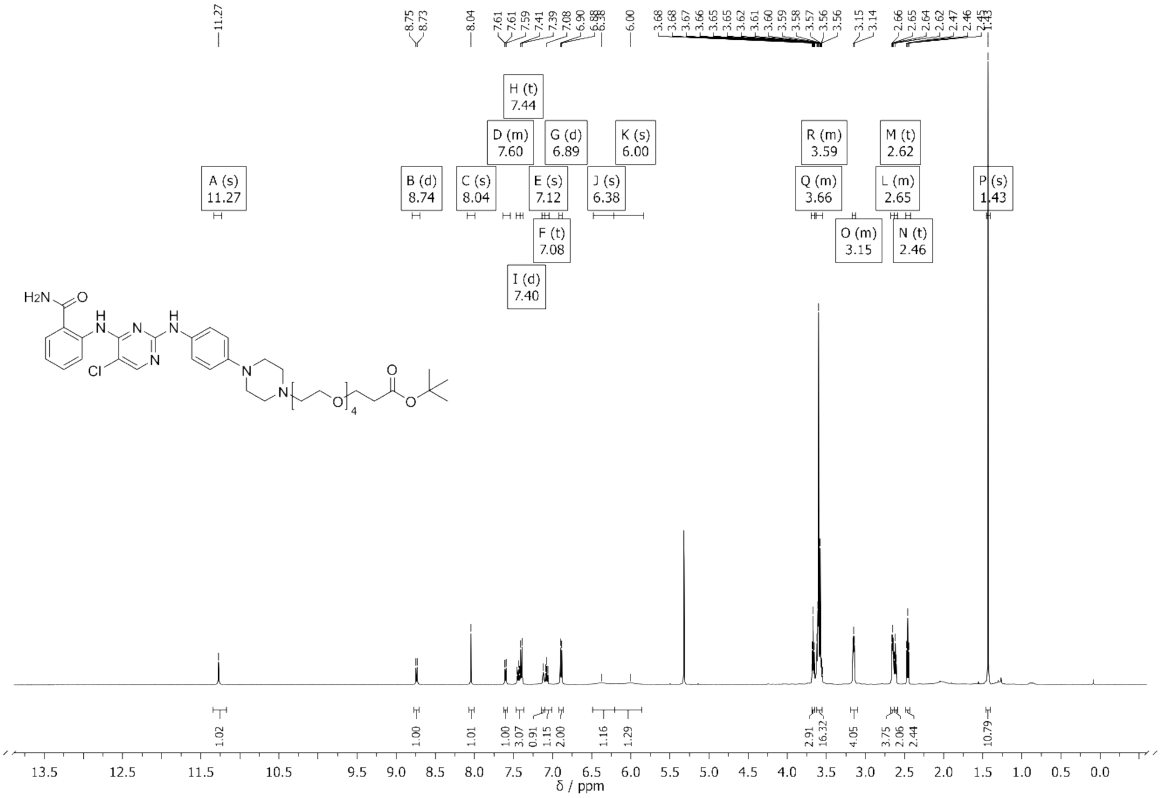

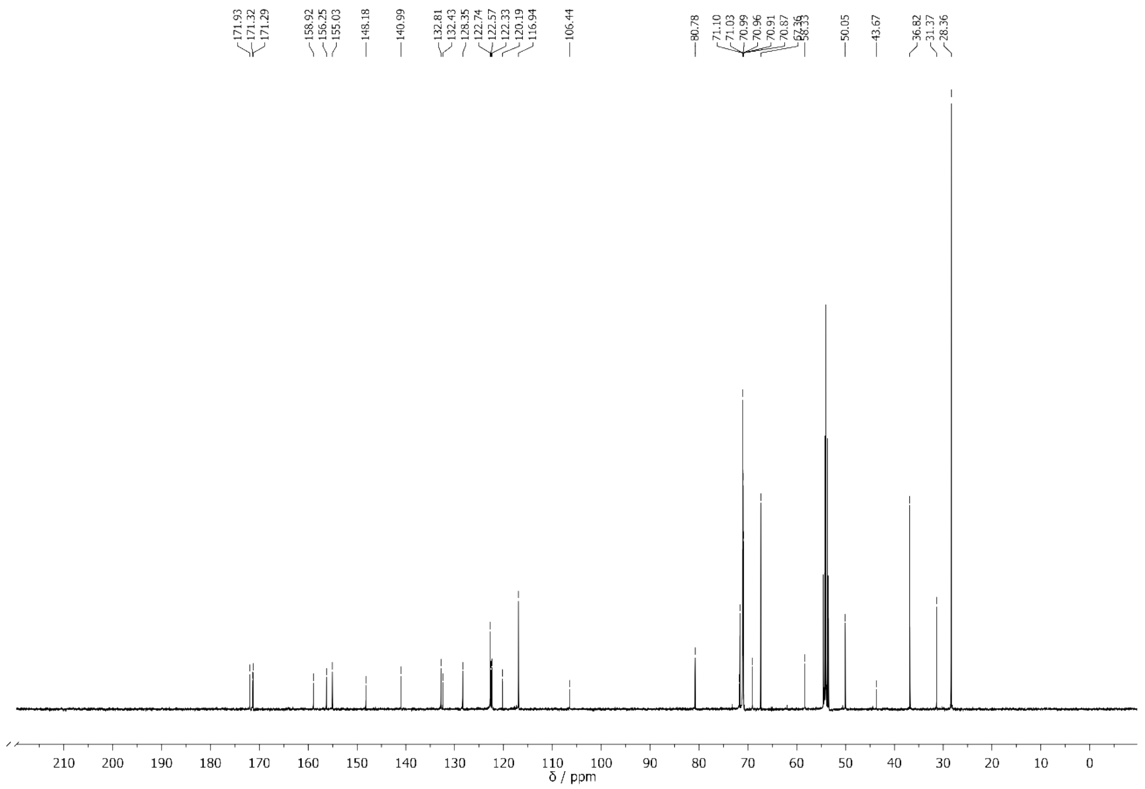

Figure S83: ^{1}H- (top) and 13C-NMR (bottom) spectra (500 MHz and 101 MHz, 298 K, DCM-d_2) and chemical structure of compound **44** .

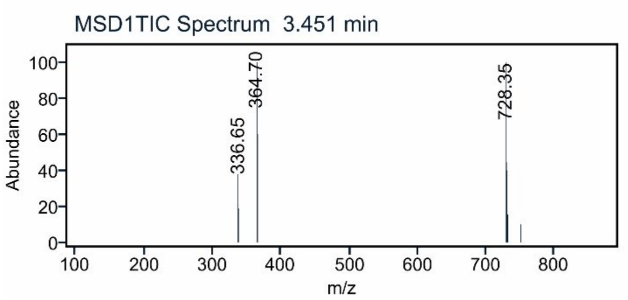

Figure S84: ESI-MS spectrum of compound **44** with $[M+H]^+_{calc.}$ = 728.4 m/z.

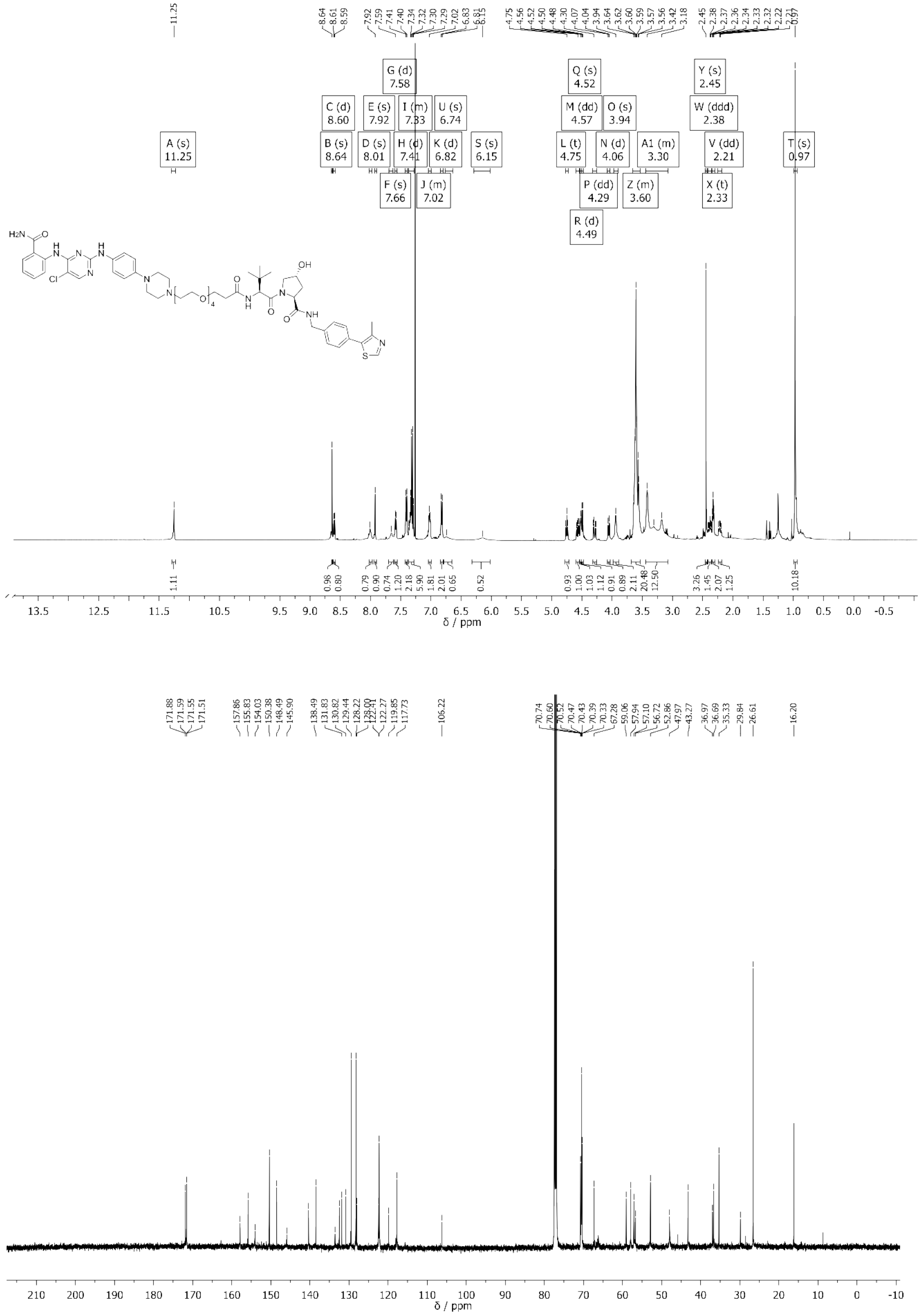

Figure S85: ^{1}H- (top) and 13C-NMR (bottom) spectra (500 MHz and 126 MHz, 298 K, $CDCl_3$) and chemical structure of compound **6-b**.

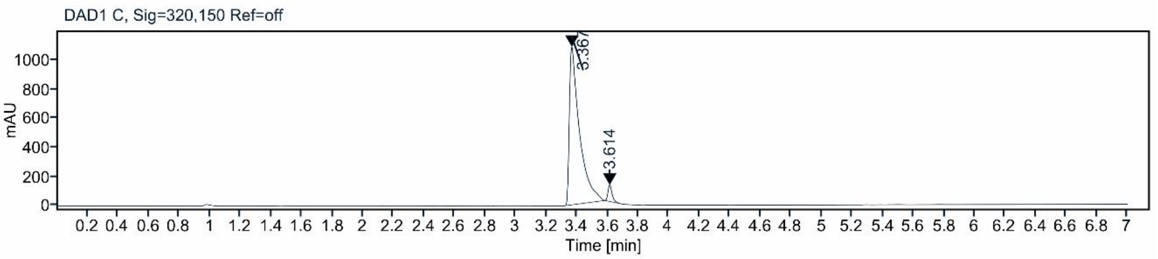

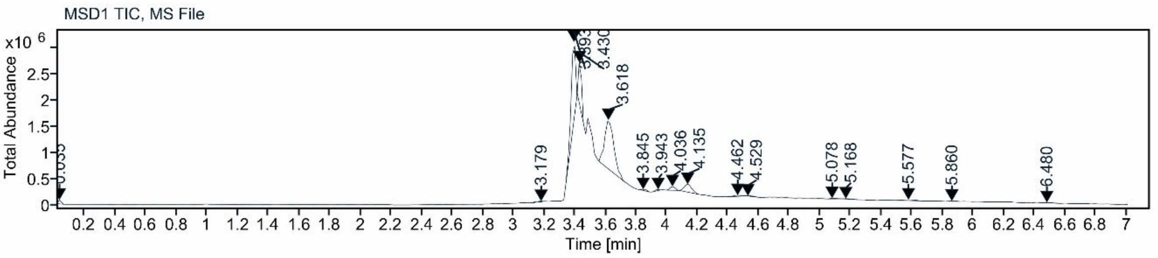

Signal Description DAD1 C, Sig=320,150 Ref=off

Sample Name	Name	RT	Width	Area	Area%	Height
LH147RP22		3.367	0.064	4987.6987	95.91	1084.3247
LH147RP22		3.614	0.027	212.6433	4.09	112.9978

Max Area%	95.911
UV Signal Purity>95%	Pass

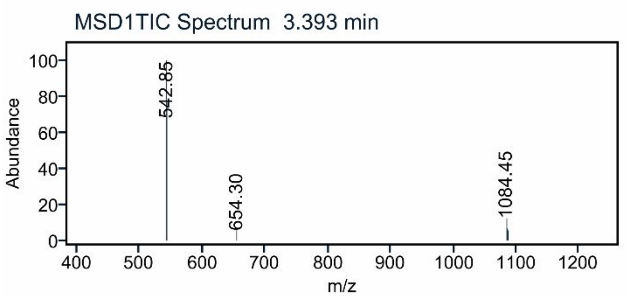

Figure S86: LC/MS spectra of purified compound **6-b** at 320 nm wavelength with $[M+H]^{+}_{calc.}$ = 1084.5 m/z.

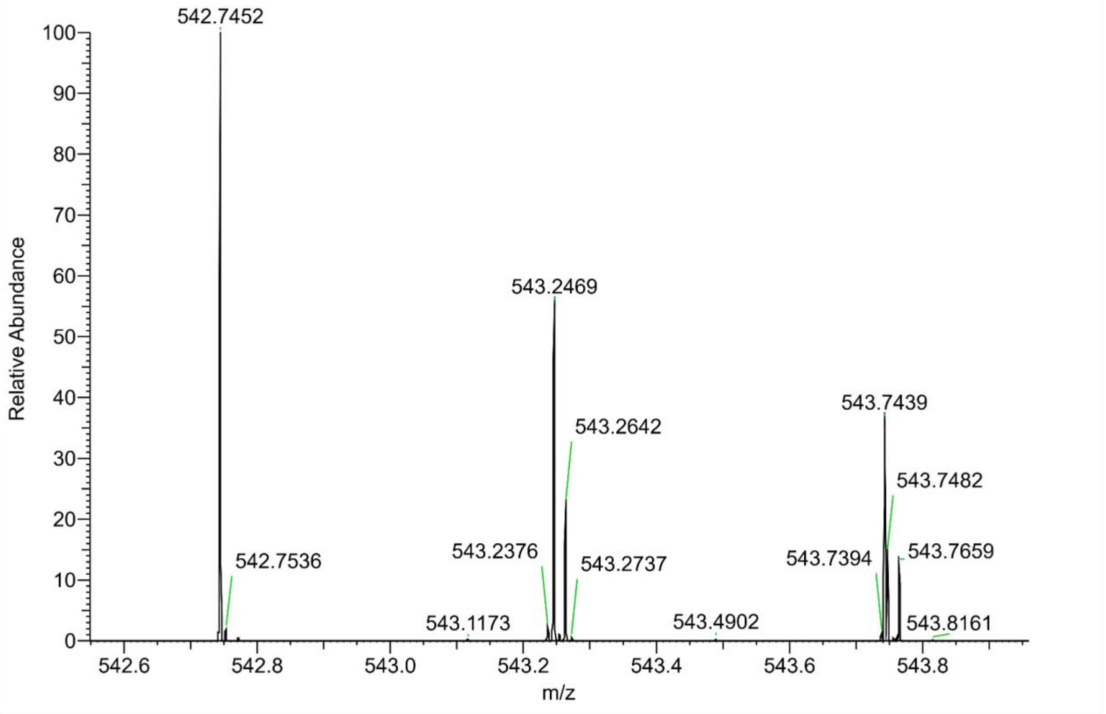

Figure S87: High-resolution mass spectrum of compound **6-b** with $[M/2+H]^{2+}_{calc.}$ = 542.7456 m/z.

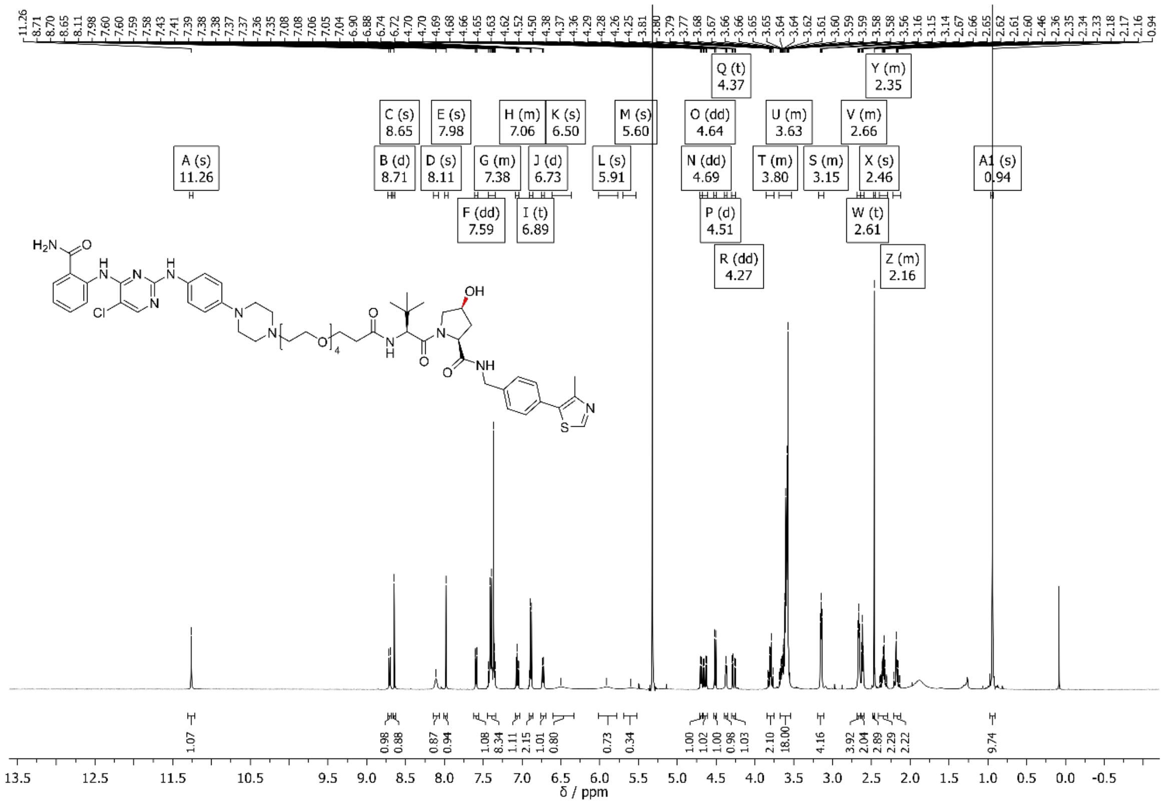

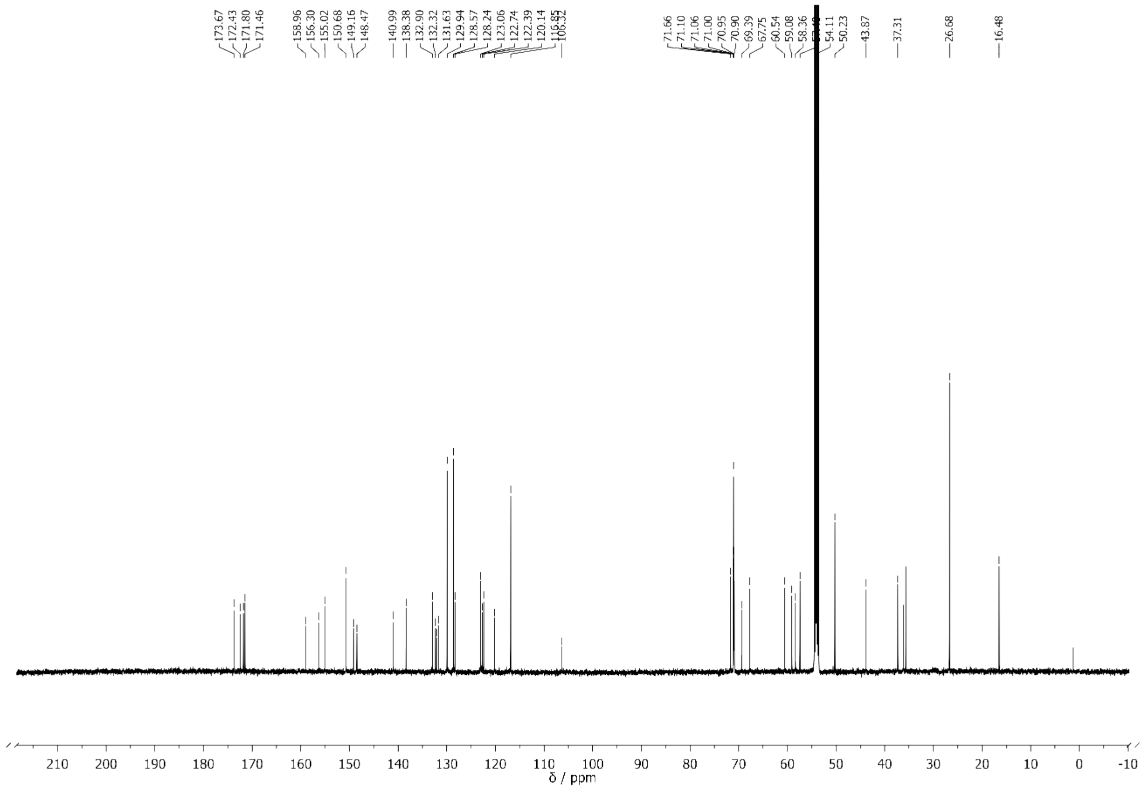

Figure S88: ^{1}H- (top) and 13C-NMR (bottom) spectra (500 MHz and 126 MHz, 298 K, DCM-d_2) and chemical structure of compound **6-b^{neg}**.

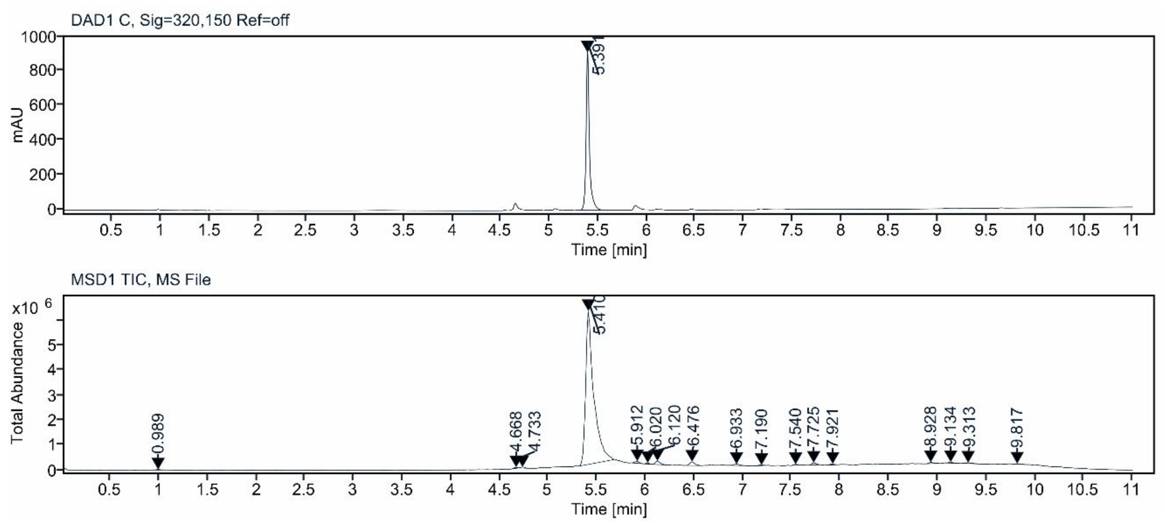

Signal Description DAD1 C, Sig=320,150 Ref=off

Sample Name	Name	RT	Width	Area	Area%	Height
jw-442 x		5.391	0.031	2172.9417	100.00	907.2358

Max Area% 100.000

UV Signal Purity>95% Pass

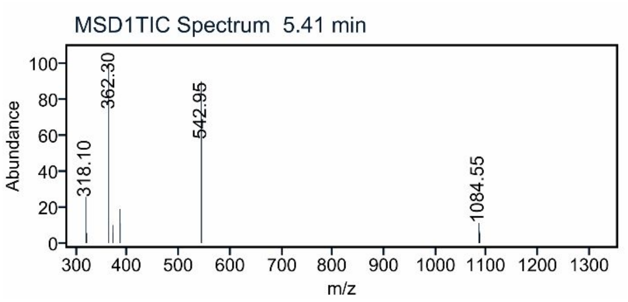

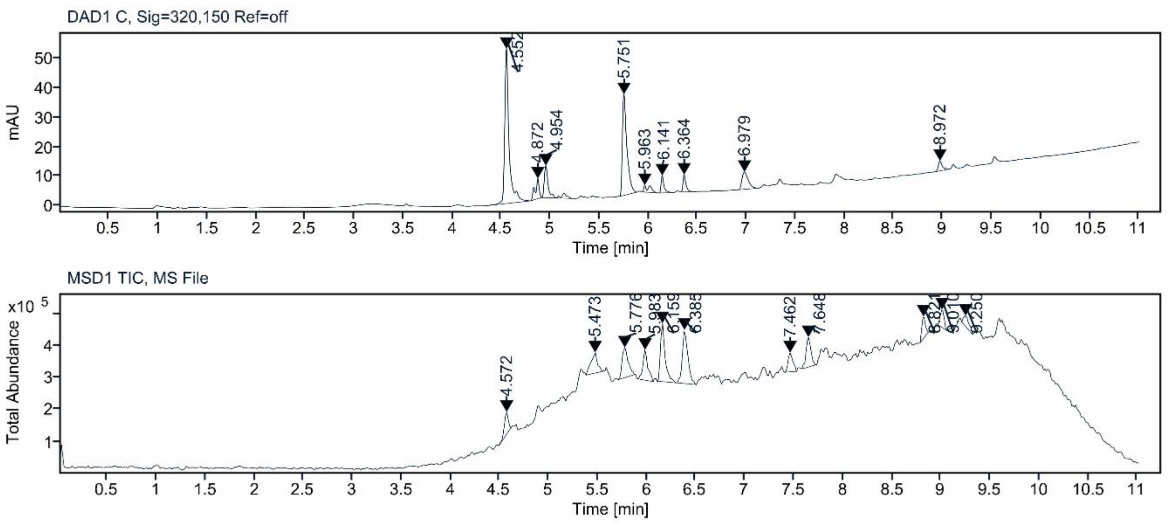

Figure S89: LC/MS spectra of purified compound **6-b^neg^** at 320 nm wavelength (top) with $[M+H]^{+}_{calc.}$ = 1084.5 m/z and blank spectrum without compound (bottom).

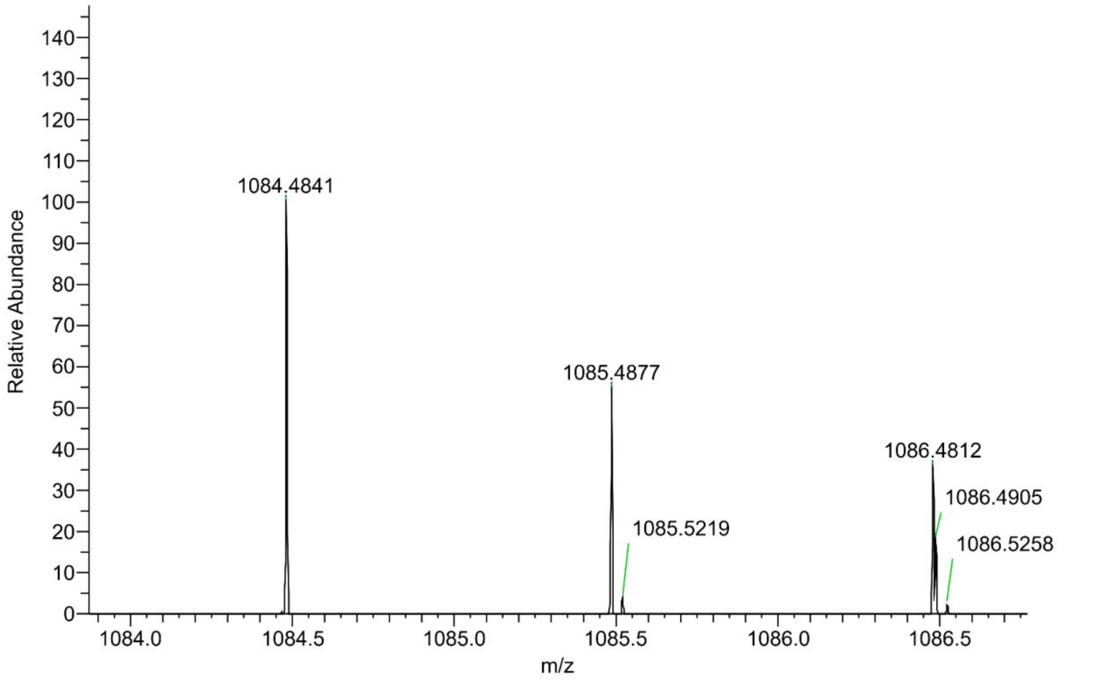

Figure S90: High-resolution mass spectrum of compound **6-b^{neg}** with $[M+H]^{+}_{calc.}$ = 1084.4840 m/z.

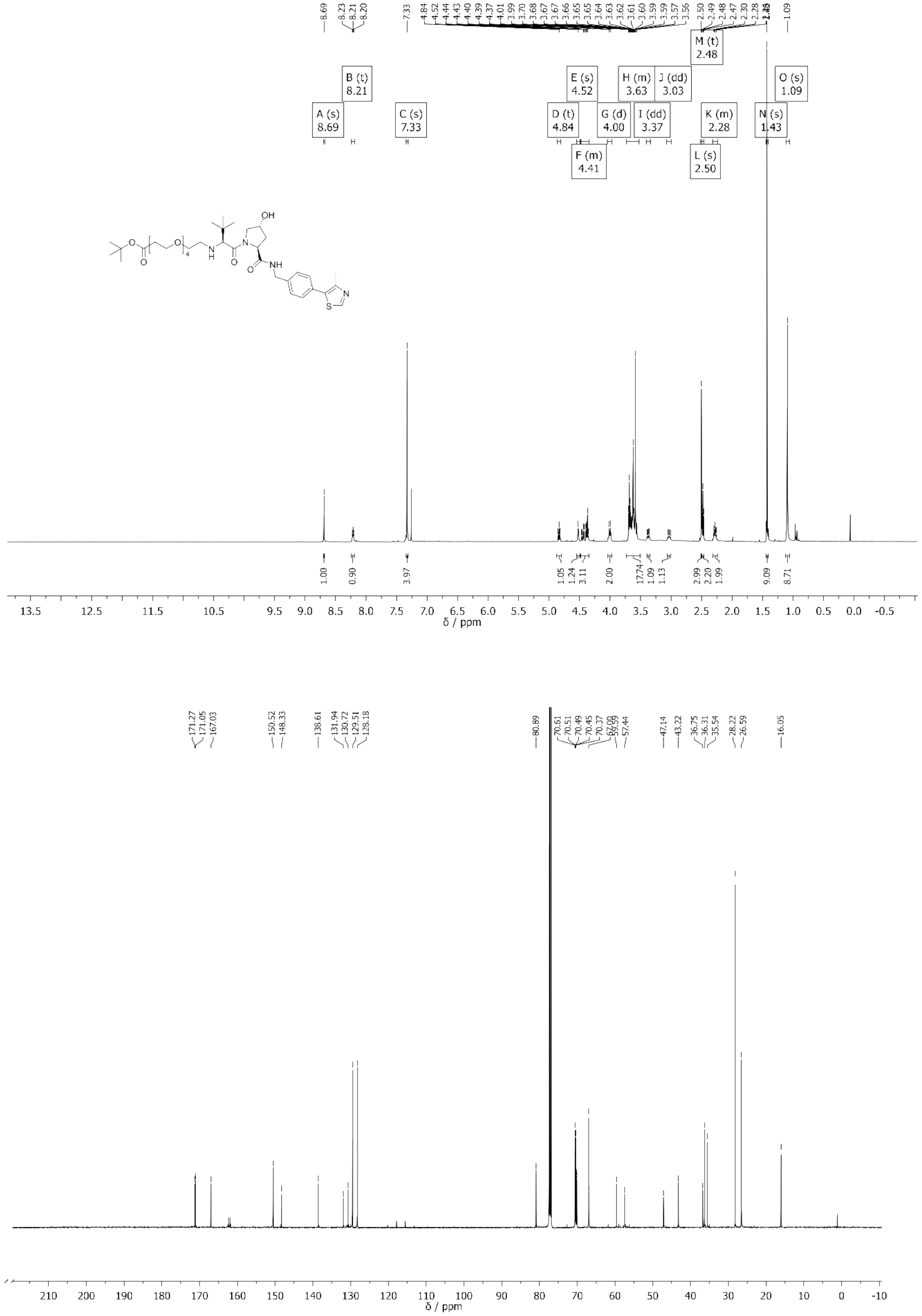

Figure S91: ^{1}H- (top) and 13C-NMR (bottom) spectra (500 MHz and 126 MHz, 298 K, DCM-d_2) and chemical structure of compound **46**.

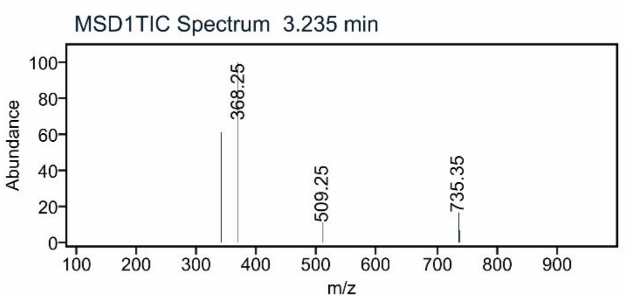

Figure S92: ESI-MS spectrum of compound **46** with $[M+H]^{+}_{calc.}$ = 735.4 m/z.

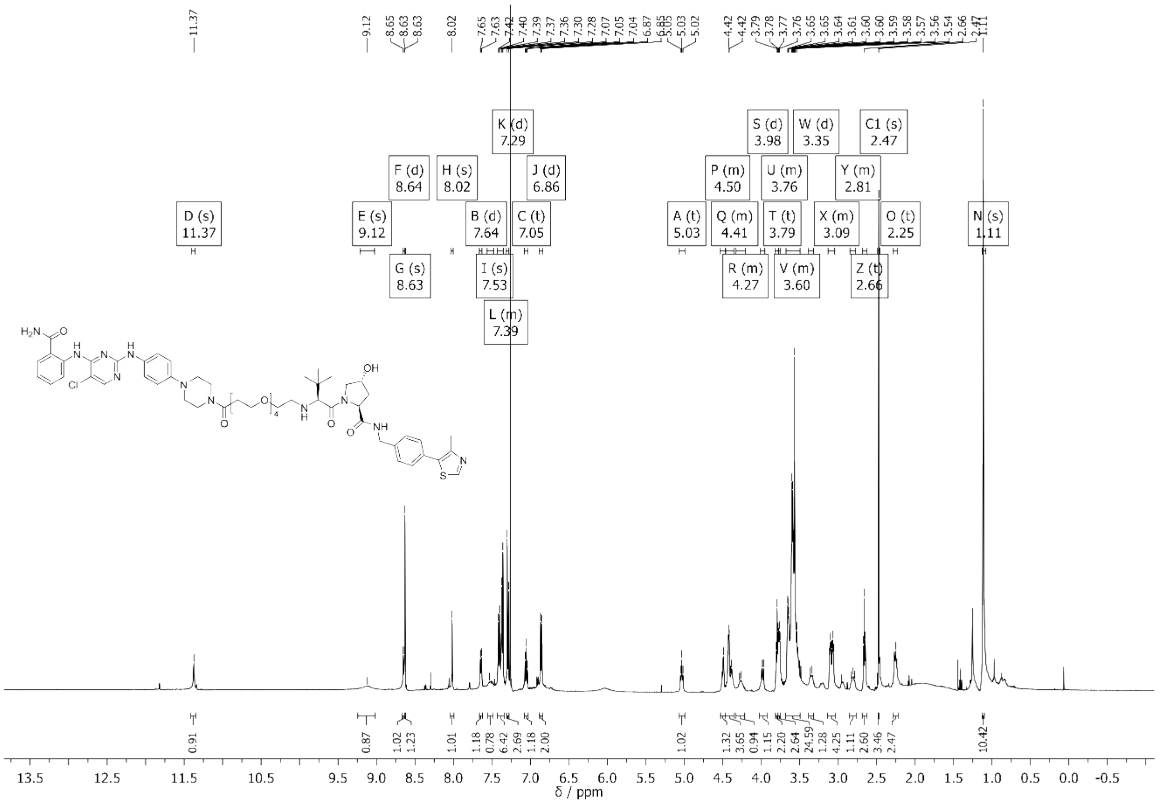

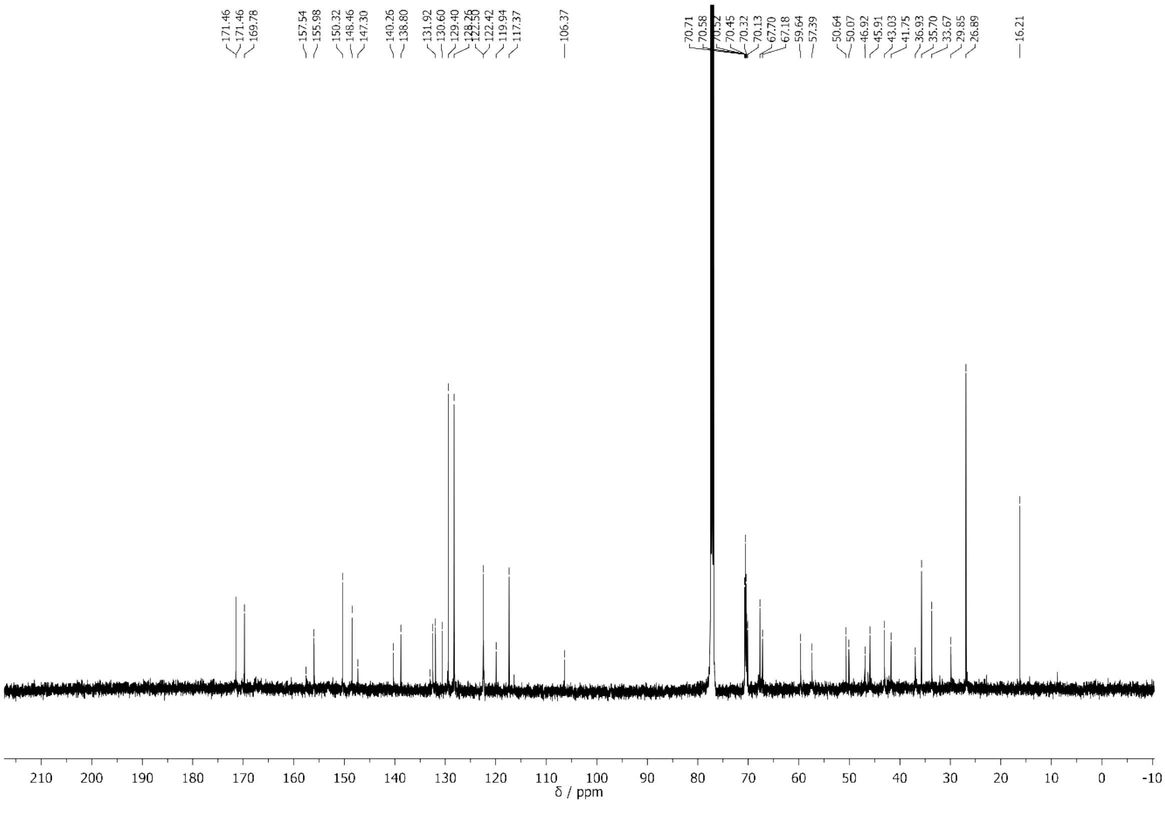

Figure S93: ^{1}H- (top) and 13C-NMR (bottom) spectra (500 MHz and 126 MHz, 298 K, $CDCl_3$) and chemical structure of compound **6-c**.

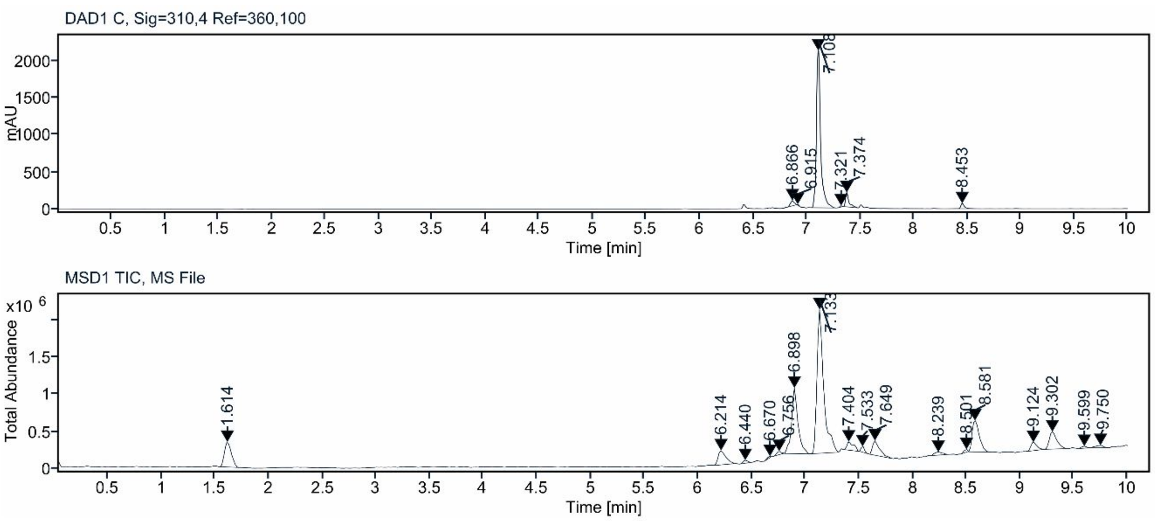

Signal Description DAD1 C, Sig=310,4 Ref=360,100

Sample Name	Name	RT	Width	Area	Area%	Height
VN2030		6.866	0.042	173.7320	2.60	71.3365
VN2030		6.915	0.017	14.6741	0.22	10.8103
VN2030		7.108	0.037	5886.5850	88.02	2137.1868
VN2030		7.321	0.022	20.3606	0.30	16.0648
VN2030		7.374	0.028	431.4770	6.45	193.7011
VN2030		8.453	0.032	161.2762	2.41	70.7449

Max Area% 88.016

UV Signal Purity>95% Fail

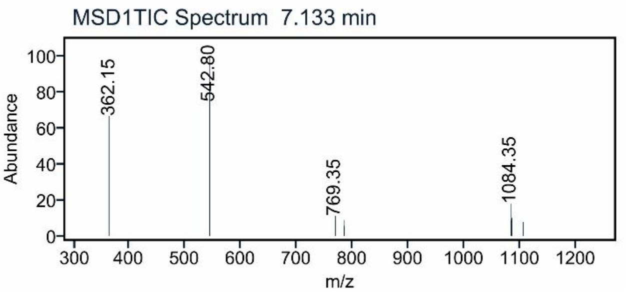

Figure S94: LC/MS spectra of purified compound **6-c** at 310 nm wavelength with $[M+H]^{+}_{calc.}$ = 1084.5 m/z.

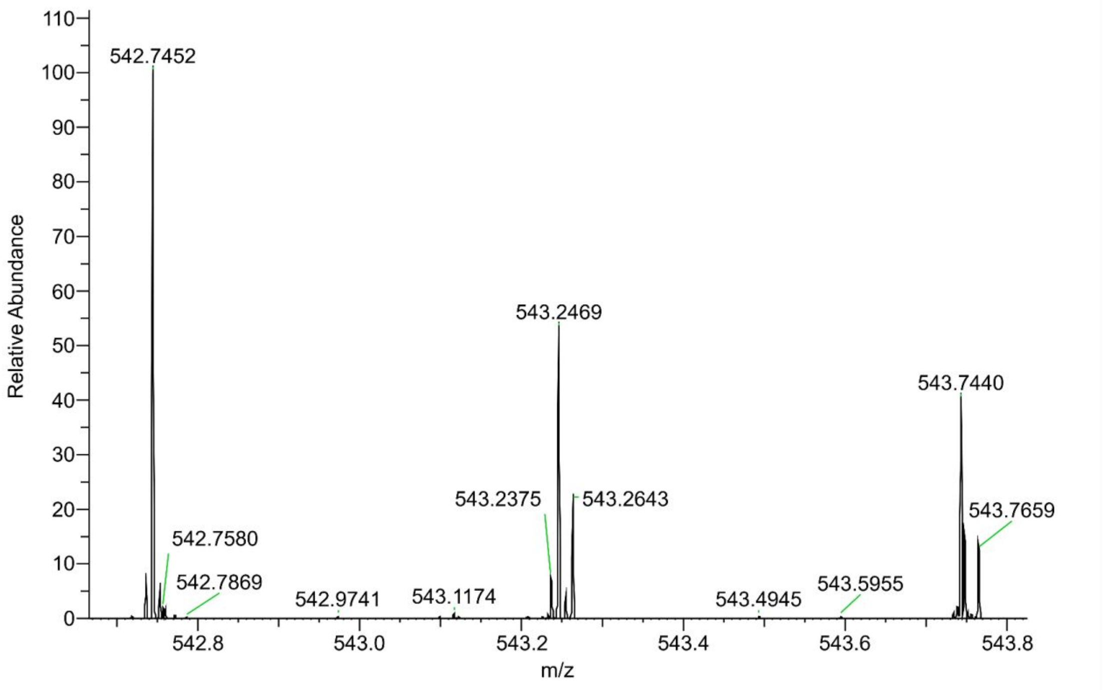

Figure S95: High-resolution mass spectrum of compound **6-c** with $[M/2+H]^{2+}_{calc.}$ = 542.7456 m/z.

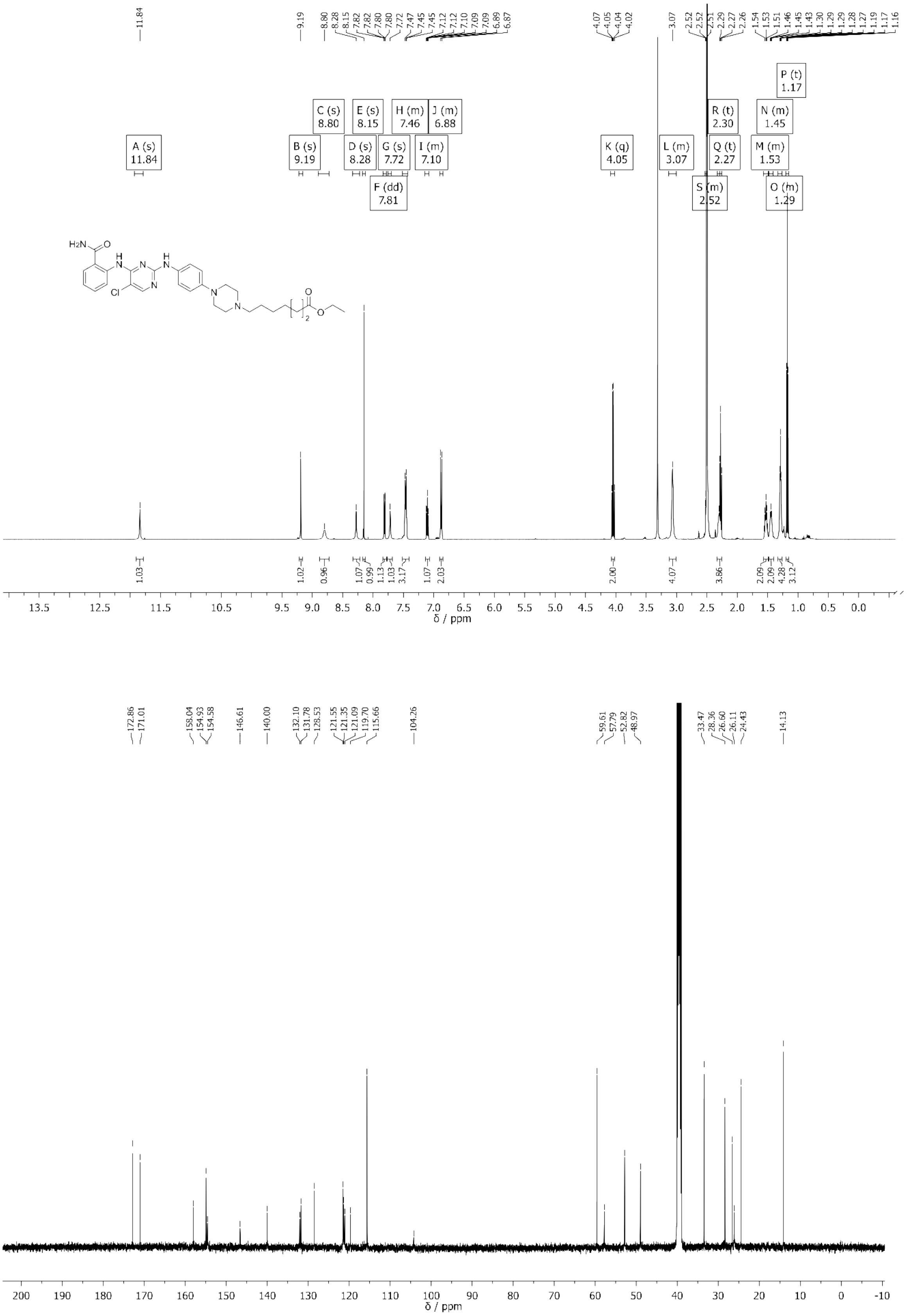

Figure S96: ^{1}H- (top) and 13C-NMR (bottom) spectra (500 MHz and 126 MHz, 298 K, DMSO-d_6) and chemical structure of compound **45**.

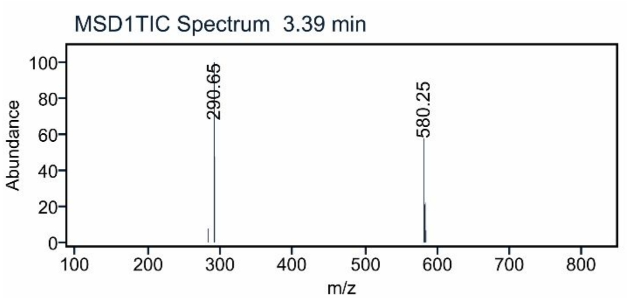

Figure S97: ESI-MS spectrum of compound **45** with $[M+H]^+_{calc.}$ = 580.3 m/z.

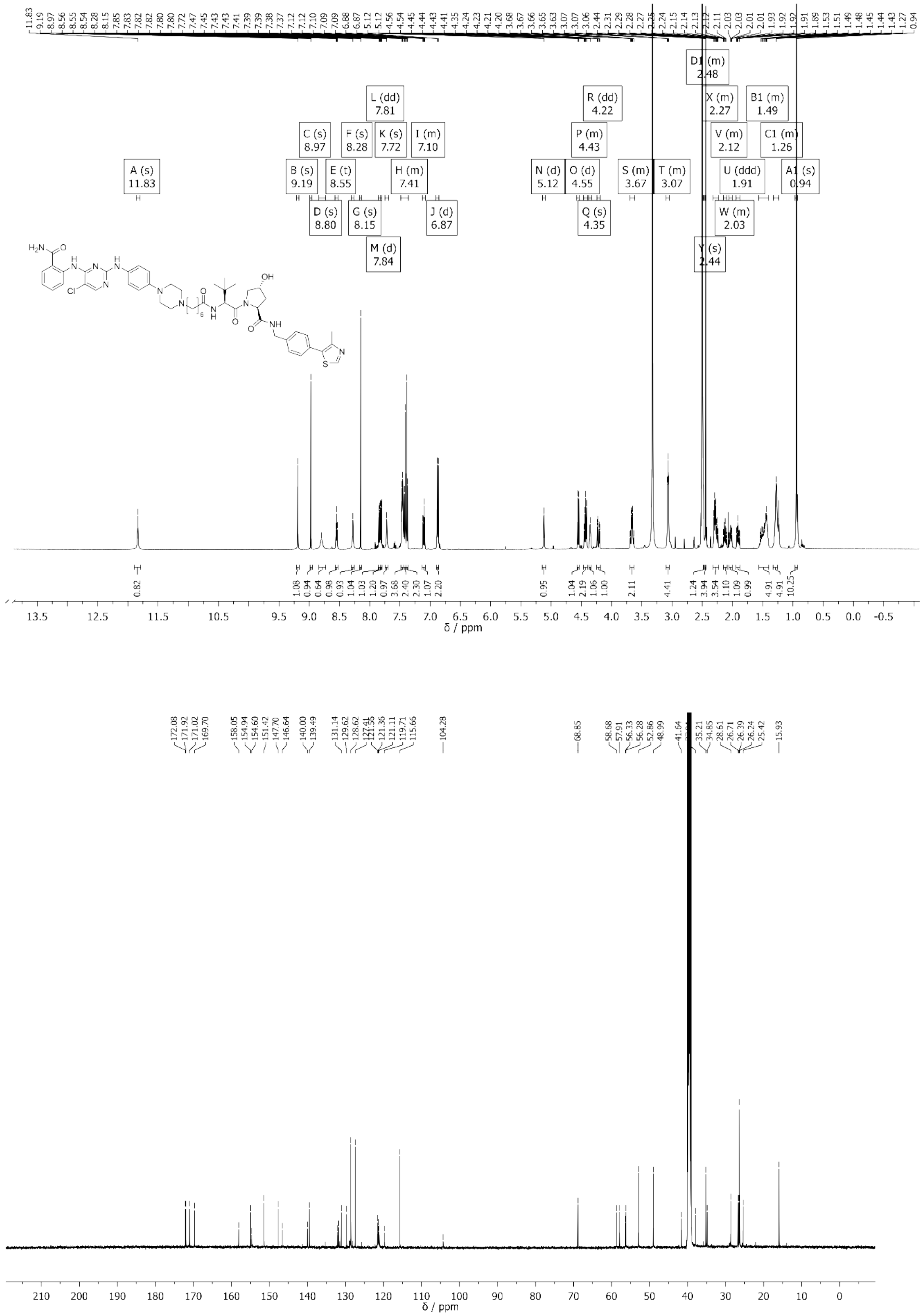

Figure S98: ^{1}H- (top) and 13C-NMR (bottom) spectra (500 MHz and 126 MHz, 298 K, DMSO-d_6) and chemical structure of compound **6-d**.

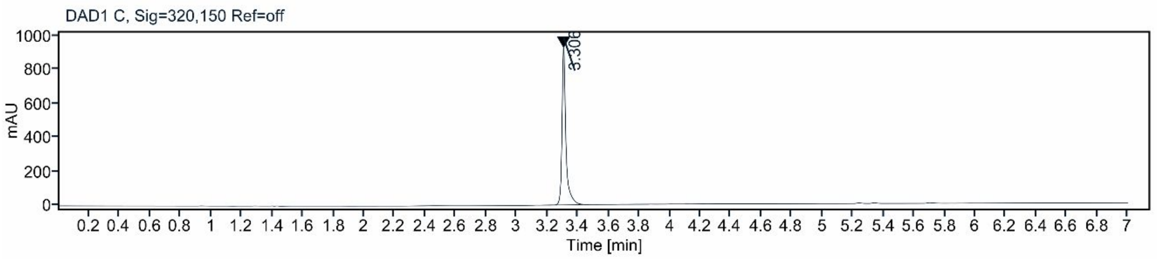

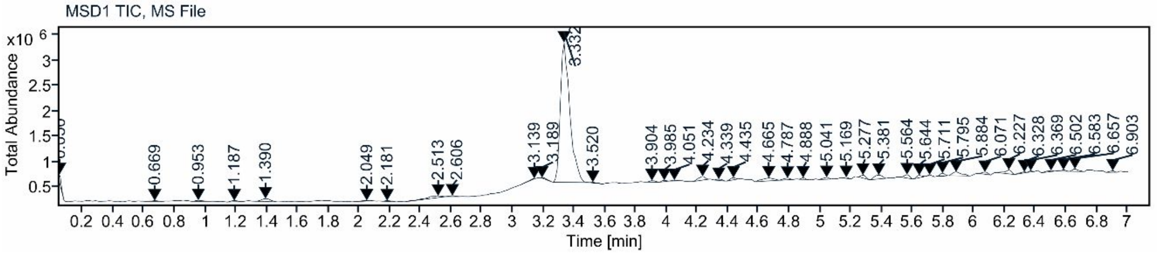

Signal Description	DAD1 C, Sig=320,150 Ref=off					
**Sample Name**	**Name**	**RT**	**Width**	**Area**	**Area%**	**Height**
jw-295 22		3.306	0.023	1654.9326	100.00	921.9699

Max Area%	100.000
UV Signal Purity>95%	Pass

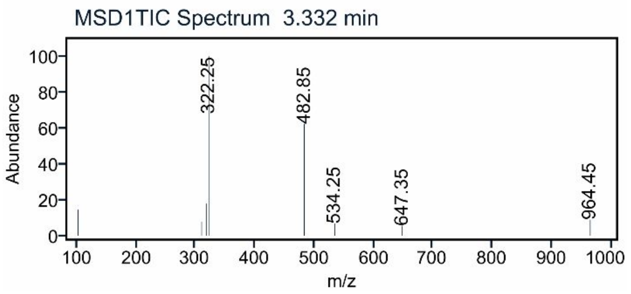

Figure S99: LC/MS spectra of purified compound **6-d** at 320 nm wavelength with $[M+H]^+_{calc.}$ = 964.4 m/z.

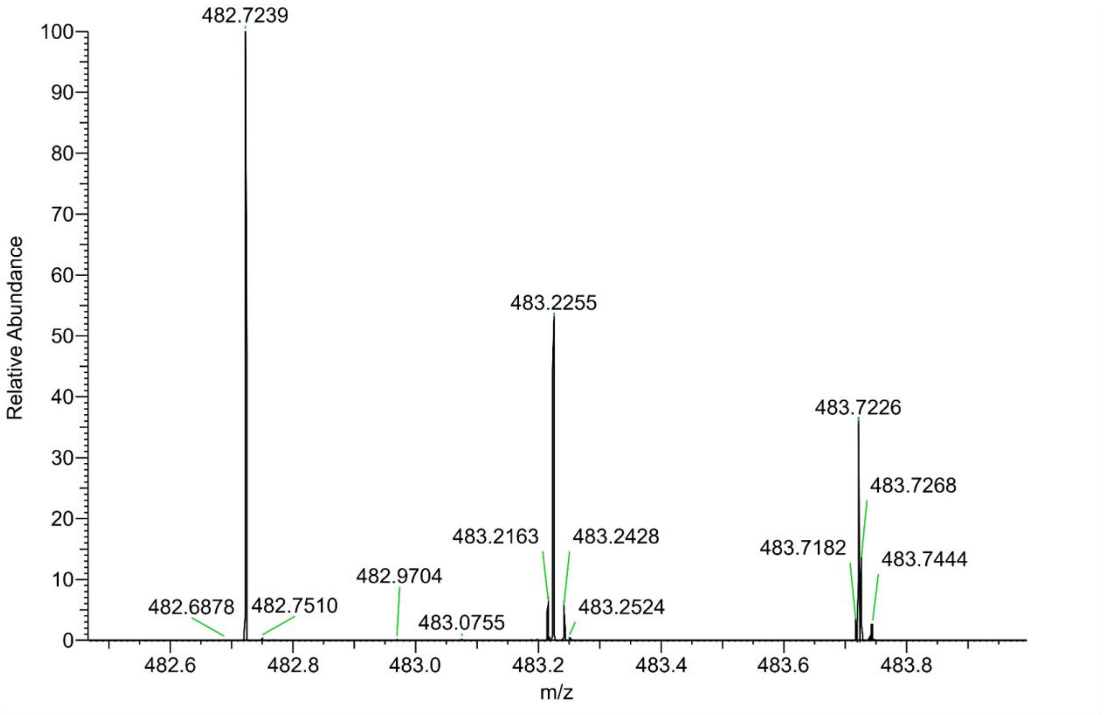

Figure S100: High-resolution mass spectrum of compound **6-d** with $[M/2+H]^{2+}_{calc.}$ = 482.7245 m/z.

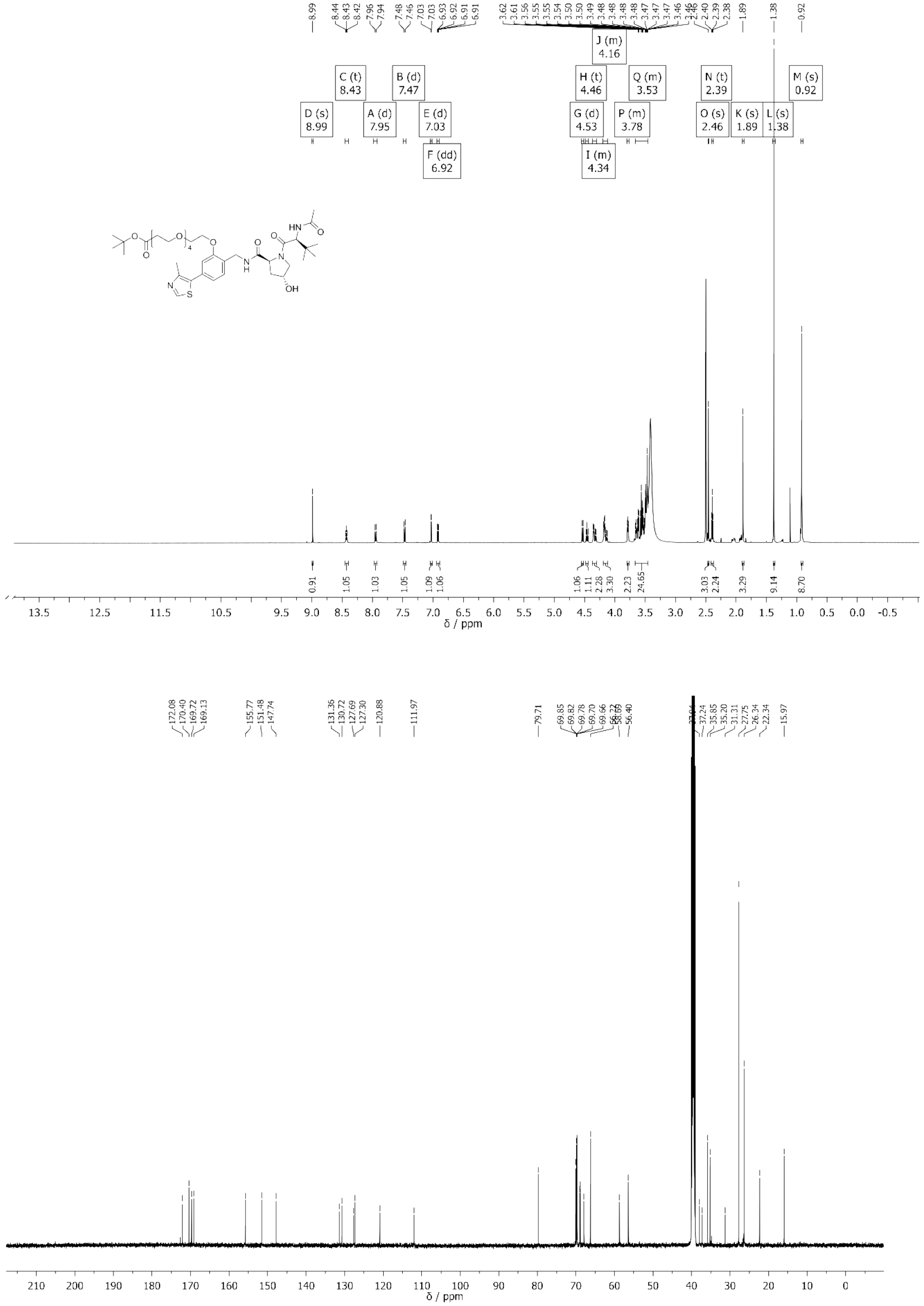

Figure S101: ^{1}H- (top) and 13C-NMR (bottom) spectra (500 MHz and 126 MHz, 298 K, DCM-d_2) and chemical structure of compound **47**.

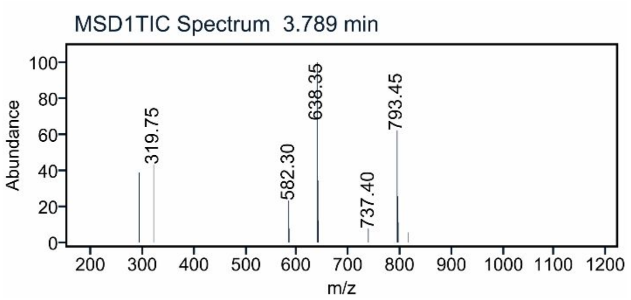

Figure S102: ESI-MS spectrum of compound **47** with $[M+H]^{+}_{calc.}$ = 793.4 m/z.

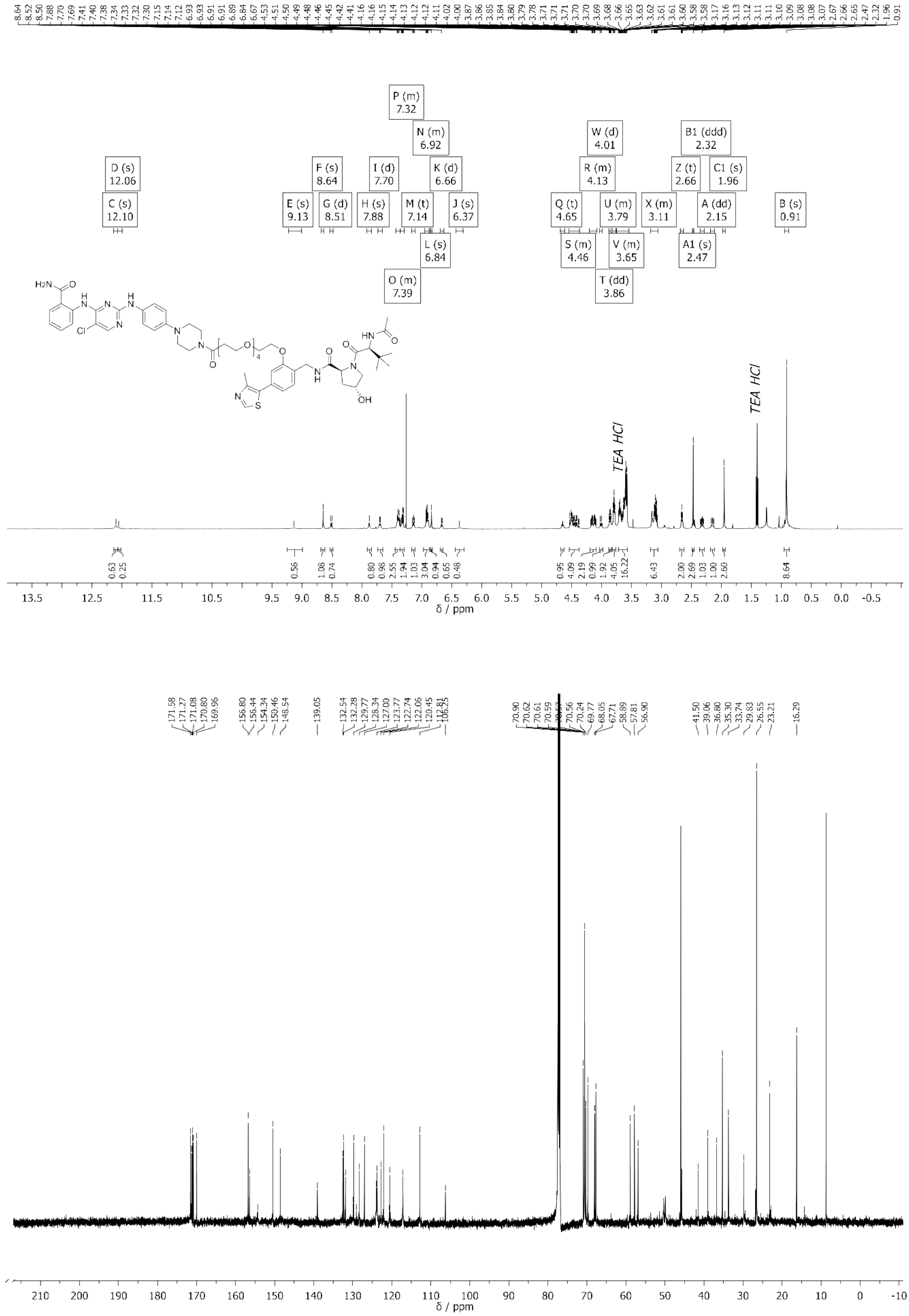

Figure S103: ^{1}H- (top) and 13C-NMR (bottom) spectra (500 MHz and 126 MHz, 298 K, $CDCl_3$) and chemical structure of compound **6-e**.

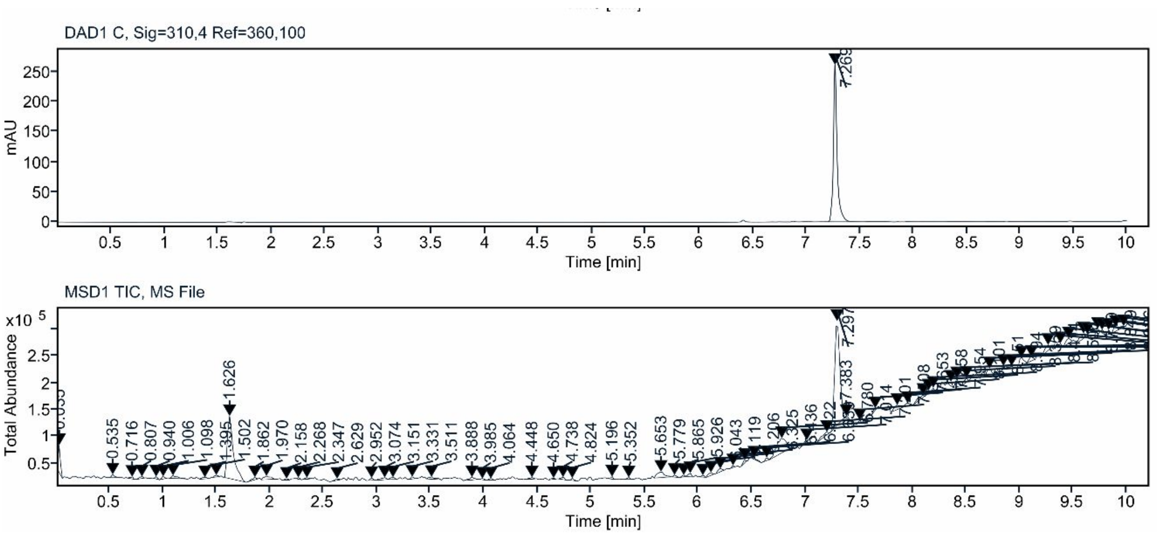

Signal Description DAD1 C, Sig=310,4 Ref=360,100

Sample Name	Name	RT	Width	Area	Area%	Height
VN2031-2		7.269	0.031	633.0694	100.00	261.6578

Max Area% 100.000

UV Signal Purity>95% Pass

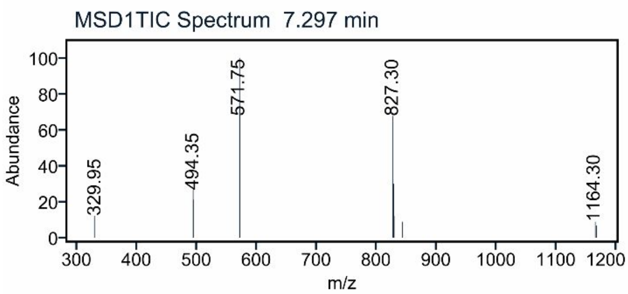

Figure S104: LC/MS spectra of purified compound **6-e** at 310 nm wavelength with $[M+Na]^{+}_{calc.}$ = 1164.5 m/z.

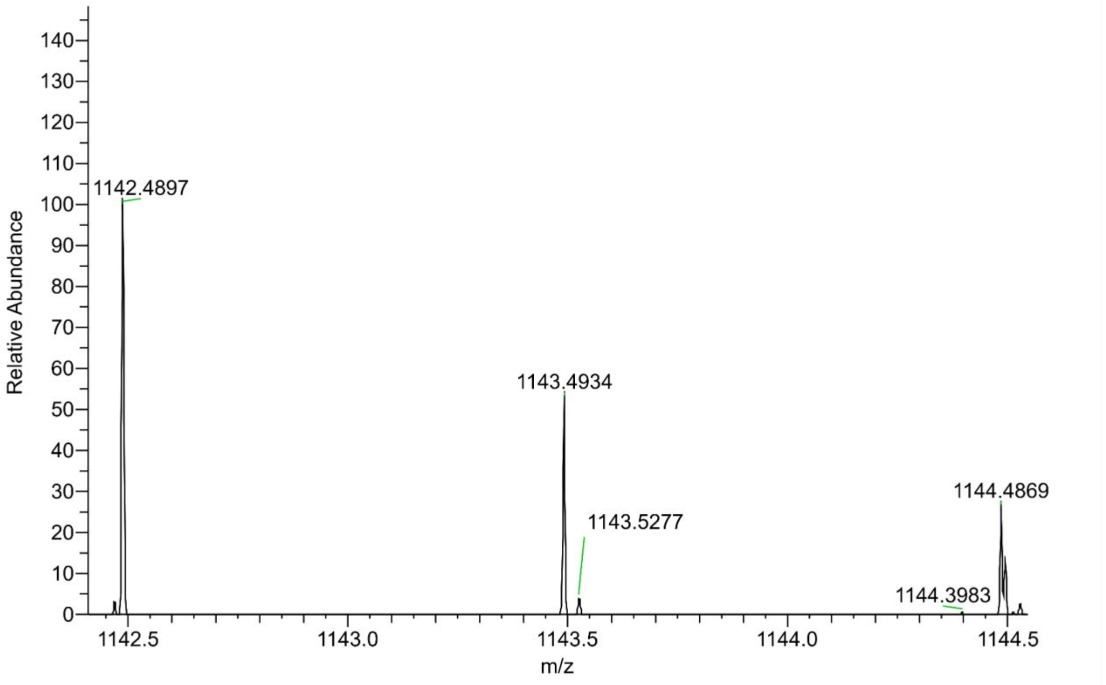

Figure S105: High-resolution mass spectrum of compound **6-e** with $[M+H]^+_{calc.}$ = 1142.4895 m/z.
